# Subcellular Localization and Mitotic Interactome Analyses Identify SIRT4 as a Centrosomally Localized and Microtubule Associated Protein

**DOI:** 10.3390/cells9091950

**Published:** 2020-08-24

**Authors:** Laura Bergmann, Alexander Lang, Christoph Bross, Simone Altinoluk-Hambüchen, Iris Fey, Nina Overbeck, Anja Stefanski, Constanze Wiek, Andreas Kefalas, Patrick Verhülsdonk, Christian Mielke, Dennis Sohn, Kai Stühler, Helmut Hanenberg, Reiner U. Jänicke, Jürgen Scheller, Andreas S. Reichert, Mohammad Reza Ahmadian, Roland P. Piekorz

**Affiliations:** 1 Institute of Biochemistry and Molecular Biology II, Medical Faculty, Heinrich Heine University Düsseldorf, 40225 Düsseldorf, Germany; laura.bergmann@hhu.de (L.B.); alexander.lang@hhu.de (A.L.); christoph.bross@hhu.de (C.B.); simonealtinoluk@gmail.com (S.A.-H.); Iris.fey@hhu.de (I.F.); andreas.kefalas@web.de (A.K.); Patrick.Verhuelsdonk@uni-duesseldorf.de (P.V.); jscheller@hhu.de (J.S.); reza.ahmadian@hhu.de (M.R.A.); 2 Molecular Proteomics Laboratory, Heinrich Heine University Düsseldorf, 40225 Düsseldorf, Germany; nina.overbeck@hhu.de (N.O.); anja.stefanski@hhu.de (A.S.); Kai.Stuehler@uni-duesseldorf.de (K.S.); 3 Department of Otolaryngology and Head/Neck Surgery, Medical Faculty, Heinrich Heine University Düsseldorf, 40225 Düsseldorf, Germany; Constanze.Wiek@med.uni-duesseldorf.de (C.W.); Helmut.Hanenberg@uni-duesseldorf.de (H.H.); 4 Institute of Clinical Chemistry and Laboratory Diagnostics, Medical Faculty, Heinrich Heine University Düsseldorf, 40225 Düsseldorf, Germany; Christian.Mielke@med.uni-duesseldorf.de; 5 Laboratory of Molecular Radiooncology, Clinic and Policlinic for Radiation Therapy and Radiooncology, Medical Faculty, Heinrich Heine University Düsseldorf, 40225 Düsseldorf, Germany; Dennis.Sohn@hhu.de (D.S.); janicke@uni-duesseldorf.de (R.U.J.); 6 Institute for Molecular Medicine I, Medical Faculty, Heinrich Heine University Düsseldorf, 40225 Düsseldorf, Germany; 7 Department of Pediatrics III, University Hospital Essen, University Duisburg-Essen, 45112 Essen, Germany; 8 Institute of Biochemistry and Molecular Biology I, Medical Faculty, Heinrich Heine University Düsseldorf, 40225 Düsseldorf, Germany; reichert@hhu.de

**Keywords:** sirtuin, SIRT4, interactome, centrosome, mitosis, HDAC6

## Abstract

The stress-inducible and senescence-associated tumor suppressor SIRT4, a member of the family of mitochondrial sirtuins (SIRT3, SIRT4, and SIRT5), regulates bioenergetics and metabolism via NAD^+^-dependent enzymatic activities. Next to the known mitochondrial location, we found that a fraction of endogenous or ectopically expressed SIRT4, but not SIRT3, is present in the cytosol and predominantly localizes to centrosomes. Confocal spinning disk microscopy revealed that SIRT4 is found during the cell cycle dynamically at centrosomes with an intensity peak in G_2_ and early mitosis. Moreover, SIRT4 precipitates with microtubules and interacts with structural (α,β-tubulin, γ-tubulin, TUBGCP2, TUBGCP3) and regulatory (HDAC6) microtubule components as detected by co-immunoprecipitation and mass spectrometric analyses of the mitotic SIRT4 interactome. Overexpression of SIRT4 resulted in a pronounced decrease of acetylated α-tubulin (K40) associated with altered microtubule dynamics in mitotic cells. SIRT4 or the N-terminally truncated variant SIRT4(ΔN28), which is unable to translocate into mitochondria, delayed mitotic progression and reduced cell proliferation. This study extends the functional roles of SIRT4 beyond mitochondrial metabolism and provides the first evidence that SIRT4 acts as a novel centrosomal/microtubule-associated protein in the regulation of cell cycle progression. Thus, stress-induced SIRT4 may exert its role as tumor suppressor through mitochondrial as well as extramitochondrial functions, the latter associated with its localization at the mitotic spindle apparatus.

## 1. Introduction

Mitotic cell division represents a complex and highly regulated process that allows the equal partitioning of duplicated DNA content from a mother cell into two daughter cells. The mitotic spindle apparatus is comprised of two centrosomes, one at each spindle pole, astral and spindle microtubules, and microtubule-associated protein (MAP) complexes [1,2,3]. Centrosomes are the main microtubule organizing centers in animal cells comprising a pair of centrioles that are surrounded by pericentriolar material (PCM) components with pericentrin as the major anchoring factor [4]. During the G_2_/M-phase of the cell cycle, cells undergo a massive microtubule rearrangement that functions as an important regulatory switch and consists of microtubule nucleation, elongation, polymerization, and depolymerization [5]. The length of microtubules and their “dynamic instability” depend on an equilibrium shift between “catastrophe” (microtubule shrinkage) and “rescue” (microtubule growth) that is primarily regulated by several MAPs [3]. The dynamic status of microtubules and their (in)stability are critically regulated by post-translational modifications, including (de)acetylation of α-tubulin (in particular lysine 40 [K40]) [6,7]. The sirtuin SIRT2 and histone deacetylase 6 (HDAC6) are known deacetylases that target K40-acetylated α-tubulin in a NAD^+^-dependent manner [8], thereby altering microtubule dynamics by decreasing microtubule stability [9,10].

The mammalian protein family of NAD^+^-dependent sirtuins (SIRT) comprises seven members which function in different cellular compartments mainly as deacetylases, deacylases, or ADP-ribosyltransferases. SIRT proteins are implicated in multiple pathways involved in epigenetic regulation and gene expression in the nucleus (SIRT1, 2, 6, and 7), proliferation/cell survival, aging, and life-span regulation (e.g., SIRT6) [11,12,13,14,15,16], as well as mitochondrial metabolism and bioenergetics (SIRT3, 4, 5) [15,16,17,18]. SIRT3, a major mitochondrial deacetylase, and SIRT5 promote mitochondrial energy production, whereas SIRT4 exerts the opposite effect [19,20,21,22]. In particular, the metabolic gatekeepers glutamate dehydrogenase (GDH) and pyruvate dehydrogenase (PDH) [23,24] are inhibited by SIRT4 by its ADP-ribosyltransferase and Lipoamidase activities, respectively [25]. Furthermore, SIRT4 displays target-specific deacetylase [26,27] and deacylase [25,28] enzymatic activities.

Possible extra-mitochondrial roles of SIRT3 and SIRT5 are most likely due to their not well understood nuclear and cytosolic localization and their corresponding protein targets [29,30,31]. SIRT3 regulates, in a direct or indirect (i.e., mitochondria-dependent) manner, microtubule dynamics and chromosomal alignment during mitosis by currently unknown mechanism(s) [32,33]. SIRT4 could also play an extramitochondrial role in microtubule dynamics, given that SIRT4 interacts with Leucine-rich protein 130 (LRP130) [24,34], a multi-domain and dual-function protein that interacts with the microtubule-associated protein MAP1S and integrates mitochondrial transport and the microtubule cytoskeleton in interphase [35]. Moreover, recent work visualized a partial localization of SIRT4 into the nucleus that is even increased upon mitochondrial stress [36].

Consistent with a role for SIRT3 and SIRT4 as tumor suppressor proteins, knock-out mouse lines for SIRT3 and SIRT4 develop mammary and lung tumors, respectively [37,38]. The tumorigenic phenotype of SIRT4 knock-out mice is associated with an increased chromosomal missegregation and aneuploidy/polyploidy that was also detected in primary SIRT4^−/−^ mouse embryonic fibroblasts [37]. Compared to wild-type cells, SIRT4^−/−^ cells show increased DNA damage and sensitivity toward chromosomal instability upon treatment with stressors like UV radiation [37]. It is unknown, whether the tumor phenotypes of mice lacking SIRT3 or SIRT4 are primarily based on mitochondria-dependent and/or -independent (i.e., mitotic/microtubule-associated) mechanisms. Moreover, SIRT4 was recently identified at the meiotic spindle apparatus during oocyte maturation. Oocytes from aged mice display higher SIRT4 levels leading to increased meiotic defects [39], which can be ameliorated by SIRT4 depletion. Consistent with its accumulation in aged oocytes, expression of SIRT4 is upregulated during replicative and stress-induced senescence, the latter triggered by different DNA-damaging stressors [37] as well as in vivo by UV radiation in photo-aged human skin [40].

Here, we performed subcellular localization and mitotic interactome analyses of SIRT4. Our findings indicate that besides its role in mitochondrial metabolism, SIRT4 functions also as a new centrosomally localized and microtubule-associated protein possibly involved in the regulation of mitotic cell cycle progression. In particular, ectopically expressed SIRT4 precipitates with microtubules, interacts with α,β-Tubulin and HDAC6 in co-immunoprecipitation experiments, and downregulates the levels of acetyl α-tubulin (K40) in G_2_-synchronized cells. Thus, both mitochondrial localized and extra-mitochondrial SIRT4 (presumably via metabolic inhibition/ROS generation [41] and alteration of mitotic regulation and/or microtubule dynamics, respectively) may trigger the anti-proliferative tumor suppressor function(s) of SIRT4 upon replicative/mitotic stress.

## 2. Materials and Methods

### 2.1. Cell Culture

HEK293, HT1080, and HeLa cell lines were cultured at 37 °C and 5% CO_2_ in DMEM (Dulbecco’s Modified Eagle Medium) containing high glucose (4.5 g/L; Thermo Fisher Scientific, Schwerte, Germany) with 10% fetal bovine serum (FBS) and penicillin (100 units/mL)/streptomycin (100 µg/mL). Cell lines were obtained from the German Collection of Microorganisms and Cell Cultures GmbH (DSMZ, Braunschweig, Germany) (HEK293: ACC 305; HeLa: ACC 57; HT1080: ACC 315).

### 2.2. Generation of SIRT4 Expressing Cell Lines

HEK293 cell lines stably expressing SIRT4-eGFP from the pcDNA3.1 vector and mutants [enzymatically inactive SIRT4(H161Y)-eGFP and SIRT4(ΔN28)-eGFP lacking the N-terminal mitochondrial targeting signal] have been described [42] and cultured in media containing 800 µg/mL Geneticin/G418 (Genaxxon bioscience GmbH, Ulm, Germany) as permanent selection agent. HEK293 and HeLa cell lines stably expressing SIRT4-eGFP from the retroviral vector puc2CL12IPwo were generated as described elsewhere [43] and further enriched by fluorescence-activated cell sorting (FACS). Expression of SIRT4-eGFP fusion constructs was validated by immunoblotting and flow cytometry.

### 2.3. Cell Proliferation Kinetics

HEK293 cells expressing eGFP, SIRT4-eGFP, or mutants thereof were seeded at 1.5 × 10^4^ cells/well in triplicates (6-well plates). Total numbers of viable cells were determined after 2 and 4 days using the TC10 cell counter (Bio-Rad, München, Germany).

### 2.4. Live Cell Imaging

HEK293 cells expressing eGFP or SIRT4-eGFP (3 × 10^5^) were seeded on µ-Dish 35 mm plates (ibidi GmbH, Martinsried, Planegg). For live cell imaging, cells were cultured in CO_2_-independent HEPES containing media (Life Technologies/Thermo Fisher Scientific, Schwerte, Germany) at 37 °C in an isolated incubation chamber essentially as described [44]. Cells were initially imaged at brightfield and 488 nm and thereafter only at brightfield every 12 min using a Nikon Eclipse TE2000-E microscope under control of the NIS Elements Advanced Research software (Nikon; version 4.20).

### 2.5. Preparation of Total Cell Lysates for Immunoblot Analysis

Cleared cell lysates were generated using lysis buffer containing either 0.3% CHAPS (3-[(3-Cholamidopropyl) dimethylammonio]-1-propanesulfonate) or 0.5% NP-40, 50 mM Tris-HCl (pH 7.4), 150 mM NaCl, 1 mM Na_3_VO_4_, 10 mM NaF, 1 mM EDTA, 1 mM EGTA, 2.5 mM Na_4_O_7_P_2_, 1 µM DTT, 1 × cOmplete^™^ protease inhibitor cocktail (Sigma-Aldrich). Lysates were cleared by centrifugation (11,000× *g* at 4 °C for 20 min). Protein concentration of the supernatants was determined using the Bradford assay (K015.1, Carl Roth GmbH, Karlsruhe, Germany). Cell lysates subjected to immunoblot analysis were obtained by lysing cells in lysis buffer containing 0.5% NP-40 (see above). Antibodies used for immunoblot analysis are listed in Table S2.

### 2.6. Immunoprecipitation of GFP Fusion Proteins Using the Anti-GFP Nanobody or Standard Immunoprecipitation Protocols

The single-domain-anti-GFP antibody (“nanobody”) method [45] was employed to immunoprecipitate SIRT4-eGFP fusion proteins essentially as described [42]. Co-immunoprecipitation of α-tubulin interacting proteins was performed from total cell lysates using α-Tubulin specific antibodies (rabbit anti-α-tubulin, ab52899, Abcam, Berlin, Germany) and Protein A/G Sepharose beads (Santa Cruz Biotechnology, Heidelberg, Germany). Cell lysates subjected to immunoprecipitation were obtained by lysing cells in lysis buffer containing 0.3% CHAPS (see above).

### 2.7. Subcellular Fractionation Analysis

Subcellular fractionation of total cell lysates was performed essentially as described [46] with additional centrifugation steps to obtain a cytosolic fraction together with a mitochondrially enriched particulate fraction. Cells were suspended in HEPES buffered solution [20 mM HEPES, pH 7.5; 220 mM mannitol; 70 mM sucrose; 1 mM EDTA; 1× protease inhibitor cocktail (Sigma-Aldrich, München, Germany)] and mechanically lysed by repeatedly passing through 20 G syringe needles. The total cell lysate was centrifuged (600× *g*, 10 min), and the resulting crude cytoplasmatic fraction without cellular debris was subjected to at least eight further centrifugation steps (600× *g*, 1000× *g*, 16,000× *g*) thereby collecting mitochondria enriched pellets and a pure cytosolic fraction. Mitochondria containing pellets were resuspended in HEPES buffered solution containing 10 mM MgCl_2_ and 250 mM sucrose, centrifuged (12,000× *g*, 10 min) twice through a sucrose cushion (HEPES buffered solution containing 0.5 mM MgCl_2_ and 880 mM sucrose). The resulting highly mitochondria enriched pellets were resuspended [40 mM Tris HCl, pH 7.5; 150 mM NaCl; 3% glycerol; 0.5 mM DTT; 1× protease inhibitor cocktail (Sigma-Aldrich, München, Germany)] and analyzed by SDS-PAGE. Antibodies used for immunoblot analysis of subcellular marker proteins are listed in Table S2.

### 2.8. Microtubule Pulldown Experiments

Pelleting of Taxol-stabilized microtubules from cytosolic fractions was performed essentially as described [47]. Asynchronously growing HEK293 cells expressing eGFP or SIRT4-eGFP were lysed in PHEM buffer [60 mM PIPES, 25 mM HEPES, 1 mM EGTA, 1 mM magnesium acetate, pH 6.8; 1 × cOmplete^™^ protease inhibitor cocktail (Sigma-Aldrich, München, Germany)] using a Dounce homogenizer. Following centrifugation (14,000× *g* for 30 min) of the total cell lysate, the supernatant (cytosolic fraction) was supplemented with GTP (1 mM) and Paclitaxel/Taxol (20 µM) (both from Sigma-Aldrich, München, Germany). Samples were incubated at room temperature for 30 min and subjected to centrifugation (14,000× *g* for 15 min) through a sucrose layer (15% sucrose in PHEM buffer) to obtain supernatant and the microtubules containing pellet fraction. The latter was washed one time in Taxol containing PHEM buffer, centrifuged, and sample fractions were analyzed by SDS-PAGE.

### 2.9. Ro3306 Mediated G_2_ Cell Cycle Arrest

Cells were treated for 14 h with the CDK1 inhibitor Ro3306 (10 µM; Selleckchem/BIOZOL, München, Germany) to achieve synchronization at G_2_. When indicated, cells were released into mitosis by one time washing and addition of fresh media, harvested 45 min later, and analyzed as indicated.

### 2.10. Mass Spectrometric Analysis of the Mitotic SIRT4 Interactome

Sample preparation for proteomic analysis, LC-MS analysis, computational mass spectrometric data analysis, and gene ontology/protein network analysis are specified in the Supplementary Materials and Methods section. Primary data obtained from mass spectrometric analysis of SIRT4-eGFP interacting proteins are listed in Table S1.

### 2.11. Confocal Laser Scanning Microscopy and Signal Quantification Using ImageJ Software

Cells were fixed in 4% paraformaldehyde for 20 min and permeabilized with 0.2% Triton X-100 for 20 min followed by a blocking step with 4% BSA/0.05% saponin for 30 min at room temperature. Alternatively, for spinning disk confocal analysis, cells were fixed in 4% paraformaldehyde for 20 min and permeabilized with 0.25% Triton X-100 for 5 min followed by a blocking step with 3% BSA in PBS (phosphate buffered saline) for two hours at room temperature. Cells were stained with primary antibodies in 1% BSA in PBS overnight at 4 °C. All primary and secondary antibodies used for confocal imaging analysis are listed in Table S3. DNA was detected by DAPI staining followed by mounting of coverslips with ProLong Gold antifade reagent (P36934; Invitrogen/Thermo Fisher Scientific, Germany). Analyses were performed with a LSM510-Meta confocal microscope (Carl Zeiss AG, Oberkochen, Germany) equipped with 40/1.3 immersion objectives and excitation wavelengths of 468 nm, 488 nm, 543 nm, and 633 nm. In addition, an UltraVIEW spinning disk confocal microscope (Perkin Elmer, Waltham, MA, USA) with excitation wavelengths of 405 nm, 488 nm, 561 nm, and 633 nm, a 60 ×/1.4 NA oil objective, and the Volocity 6.3 software (Perkin Elmer, Rodgau, Germany) was employed. To increase detection of SIRT4-eGFP fusion proteins, primary antibodies against GFP (GF090R; Nacalai Tesque, Inc./GERBU Biotechnik GmbH, Heidelberg, Germany; 1:1000) were employed in spinning disk confocal microscopy when indicated. Image processing and quantification of centrosomal SIRT4 and Pericentrin signal intensities were performed based on ImageJ software v1.49k.

### 2.12. Statistical Analysis

Data are presented as mean ± s.d. Multiple comparisons were analyzed by one-way analysis of variance (ANOVA) followed by Tukey’s post-hoc test to identify group differences in variance analysis using the GraphPad Prism software. Results with *p* ≤ 0.05 were considered significant.

## 3. Results

### 3.1. Endogenous SIRT4 and Ectopically Expressed SIRT4-eGFP Localize at Interphase and Mitotic Centrosomes

**Figure 1:**
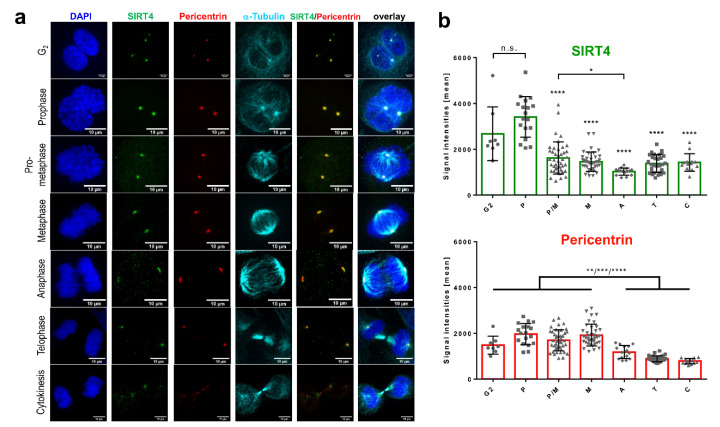
Centrosomal localization pattern of SIRT4 during G_2_/M progression. (**a**) Endogenous SIRT4 was detected in HeLa cells in G_2_ and subsequent mitotic stages using a polyclonal antibody against SIRT4 (sc-135053; Santa Cruz Biotechnology, Heidelberg, Germany) and spinning disk microscopy. Antibody staining against Pericentrin and α-tubulin was employed to visualize centrosomes and microtubules, respectively. DAPI was used to detect DNA. Bar: 10 µm. (**b**) Quantification of centrosomal SIRT4 and Pericentrin levels during G_2_/M progression. Endogenous SIRT4 and Pericentrin were detected in HeLa cells as indicated above. Shown are mean signal intensities (±S.D.) which were analyzed using ImageJ software (v1.49k; Materials and Methods). Numbers of cells analyzed per cell cycle phase: G_2_, *n* = 8; P, prophase, *n* = 17; P/M, prometaphase, *n* = 42; M, metaphase, *n* = 38; A, anaphase, *n* = 15; T, telophase, *n* = 32; and C, cytokinesis, *n* = 13. To evaluate statistical significance (comparison of SIRT4 intensities between Prophase and G_2_ or mitotic phases) one-way ANOVA followed-up by Tukey’s test was performed (* *p* < 0.05; ** *p* < 0.01; *** *p* < 0.001; **** *p* < 0.0001; n.s., not significant).

**Figure 2:**
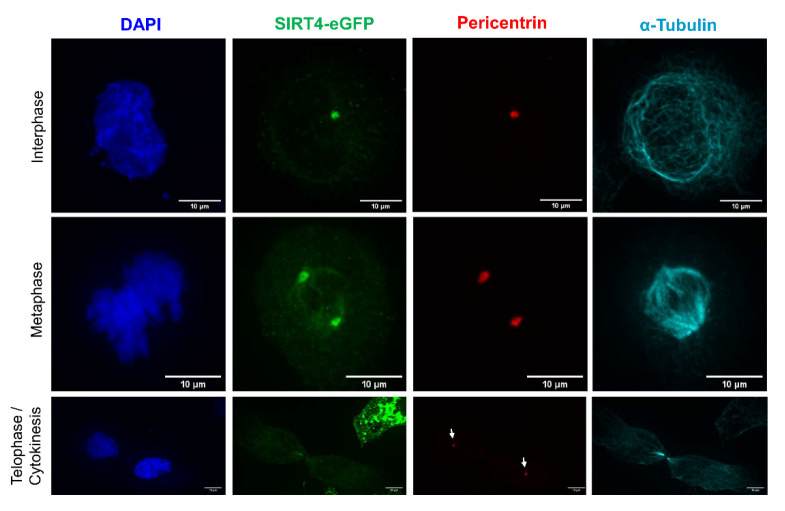
Centrosomal localization of SIRT4-eGFP during interphase and mitotic cell division. An expression construct for SIRT4-eGFP was transiently transfected into HeLa cells and SIRT4-eGFP was imaged by confocal spinning disk microscopy. The corresponding movies of these confocal pictures are provided in the supplementary information (Videos S2–S4). Antibodies against Pericentrin and α-tubulin were employed to visualize centrosomes and microtubules, respectively. DAPI was usedto detect DNA. Bar: 10 µm.

During our studies on the expression and mitochondrial function of SIRT4 [40,42,48], we noticed an extra-mitochondrial localization of SIRT4 primarily at centrosomes and apparently in part at the mitotic spindle in confocal laser scanning and spinning disk microscopy-based analyses of various human cell lines. We employed two anti-human SIRT4 antibodies, H-234 (sc-135053, rabbit polyclonal; Santa Cruz Biotechnology, Heidelberg, Germany) raised against a N-terminally truncated version of human SIRT4 (a.a. 81–314), and SAB1407208 (mouse monoclonal; Sigma-Aldrich, München, Germany) raised against full-length human SIRT4 (a.a. 1–314). As indicated in Figures S1 and S2, we observed a clear centrosomal localization of SIRT4 during interphase/G_2_ and mitosis in HeLa and HT1080 cells when using the SIRT4 antibody from Santa Cruz Biotechnology in single antibody stainings and DAPI-mediated DNA detection. Moreover, staining with the SIRT4 antibody from Sigma-Aldrich revealed also a centrosomal/mitotic spindle pole associated localization of endogenous SIRT4 in HeLa cells (Figure S3). Similar to endogenously expressed SIRT4, C-terminal eGFP fusion proteins of SIRT4 and SIRT4(ΔN28), the latter representing an N-terminally (a.a. 1–28) truncated SIRT4 mutant unable to translocate into mitochondria [42,49], were also detected at interphase centrosomes of HT1080 fibrosarcoma and HeLa cervix carcinoma cells (Figure S4 and Video S1). The bona fide centrosomal localization of SIRT4 and SIRT4-eGFP has been verified in further experiments described below by co-staining against Pericentrin (Figure 1 and Figure 2; Videos S2–S4). Besides its extramitochondrial/centrosomal localization, SIRT4 was also observed as described in mitochondria [23,42] using co-staining against the mitochondrial marker MTC02 (Figure S5; [40,42]). In clear contrast to SIRT4, SIRT3 was not detectable at interphase or mitotic centrosomes or at the mitotic spindle apparatus, but displayed solely a mitochondrial localization (Figure S6) as previously described [50].

### 3.2. Centrosomal Localization Kinetics of SIRT4 during Cell Cycle Progression

Next, we quantitatively analyzed SIRT4 at centrosomes during G_2_ and the course of mitotic cell division using Pericentrin as centrosomal marker. In HeLa cervix carcinoma cells, SIRT4 showed a dynamic centrosomal localization pattern where it displayed the highest signals in centrosomal staining during G_2_ and early mitosis, followed by a significant drop in signal intensity from prophase onwards until late mitosis/cytokinesis (Figure 1a,b). At the same time, centrosomal Pericentrin levels were comparable between G_2_ and metaphase, but significantly dropped thereafter in the second half of mitosis (Figure 1a,b), similar to the centrosomal dynamics of Pericentrin as originally described by Dictenburg et al. [51]. Similarly, SIRT4-eGFP that was transiently expressed in HeLa cells localized at centrosomes in interphase cells (Figure 2 and Video S2), prominently decorated Pericentrin at spindle poles in metaphase cells (Figure 2, Video S3), and lastly disappeared from centrosomes during telophase/cytokinesis (Figure 2, Video S4). Parallel control imaging experiments failed to detect eGFP localization at centrosomes (Video S5). Thus, centrosomal localization of SIRT4 or ectopically expressed SIRT4-eGFP seems to be dynamically regulated during cell cycle progression with a peak in G_2_ and early mitosis.

### 3.3. Subcellular Fractionation Reveals a Cytosolic, Extra-Mitochondrial Pool of SIRT4, But Not SIRT3

**Figure 3:**
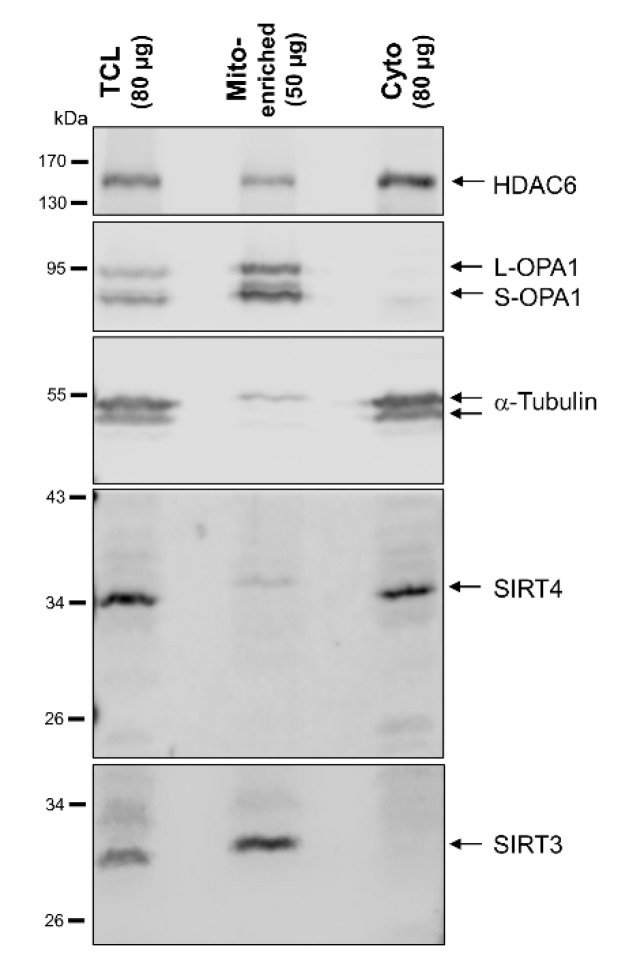
Subcellular fractionation analysis of endogenous SIRT4 and SIRT3 protein levels in HEK293 cells. Total cell lysates (TCL; 80 µg) and the respective mitochondria enriched (Mito-enriched; 50 µg) and cytosolic (Cyto; 80 µg) fractions were subjected to immunoblot analysis. Subcellular marker proteins detected were OPA1 (long and short forms of OPA1; mitochondria), α-tubulin (cytoplasm), and HDAC6 (predominantly cytoplasmatic localization).

Given the extramitochondrial localization of SIRT4 at centrosomes, we next analyzed the intracellular SIRT4 protein distribution by subjecting total cell lysates to a subcellular fractionation protocol via differential centrifugation steps (Material and Methods). The method yielded in a highly cleared cytosolic fraction together with a mitochondrial-enriched particulate fraction as controlled by marker proteins specific for subcellular compartments. Interestingly, in addition to their mitochondrial localization, both endogenous SIRT4 (Figure 3) as well as ectopically expressed SIRT4-eGFP (Figure S7) were also found at substantial levels in the cytosolic fraction. In the case of SIRT5, we also observed ectopically expressed, C-terminally Flag-tagged SIRT5 in the cleared cytosolic fraction (Figure S8), consistent with previous findings [31]. In contrast, endogenous SIRT3 or C-terminally Flag-tagged SIRT3 were only found in the mitochondrial enriched fraction (Figure 3 and Figure S8, respectively). Taken together, our findings obtained from confocal microscopic imaging and subcellular fractionation analyses both indicate that substantial levels of SIRT4, but not SIRT3, localize outside mitochondria in the cytoplasm.

### 3.4. Ectopic Overexpression of SIRT4 or the Extra-Mitochondrial Localized Deletion Mutant SIRT4(ΔN28) Inhibits Mitotic Progression and Cell Proliferation

**Figure 4:**
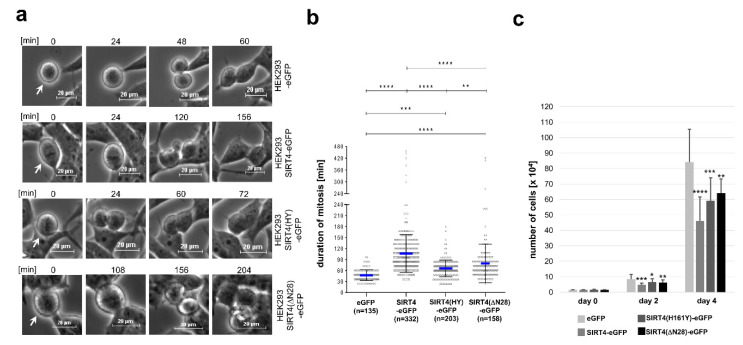
Ectopic expression of SIRT4 prolongs mitotic progression and inhibits cell proliferation. (**a**) HEK293 cell lines stably expressing SIRT4-eGFP, the enzymatically inactive mutant SIRT4(H161Y)-eGFP, or SIRT4(ΔN28)-eGFP lacking the N-terminal mitochondrial targeting signal were analyzed by live cell imaging. Cell lines were cultured in CO_2_-independent, HEPES containing media. Pictures of cells undergoing cell division were taken in intervals of 12 min. (**b**) Duration of mitosis was calculated from cell rounding/early mitosis to cytokinesis/cell reattachment. To evaluate statistical significance one-way ANOVA followed-up by Tukey’s test was performed (** *p* < 0.01; *** *p* < 0.001; **** *p* < 0.0001). (**c**) Proliferation kinetics of HEK293 cells expressing SIRT4-eGFP or the indicated SIRT4 mutants. Cells were seeded at day 0 (15,000 cells/well) in triplicates and total cell numbers were counted at the indicated time points (*n* = 3–5 independent experiments). To evaluate statistical significance one-way ANOVA followed-up by Tukey’s test was performed (* *p* < 0.05; ** *p* < 0.01; *** *p* < 0.001; **** *p* < 0.0001).

Given the stress induced/DNA damage-associated upregulation of SIRT4 and its anti-proliferative role [37,40], we next aimed to link extramitochondrially localized SIRT4 to a possible inhibitory function on cell cycle progression and proliferation. HEK293 cells stably expressing SIRT4-eGFP, SIRT4(ΔN28)-eGFP, the catalytically inactive mutant SIRT4(H161Y)-eGFP, or eGFP as control, were subjected to continuous live cell imaging analyses during cell division. As depicted and quantitatively analyzed in Figure 4, expression of all three SIRT4 variants led to a significant prolongation of mitosis with strongest impacts of SIRT4-eGFP and the exclusively outside mitochondria localized SIRT4(ΔN28)-eGFP fusion protein. In accordance with these findings, cellular proliferation was significantly reduced by all three SIRT4 variants as compared to eGFP-expressing cells (Figure 4c). Of note, expression of SIRT4(ΔN28)-eGFP was associated with an almost three-fold increase in bi- or multinucleated cells (Figure S9). The observation that SIRT4(H161Y)-eGFP, albeit catalytically inactive, still delays mitosis and inhibits proliferation indicates that SIRT4 possibly targets structural or regulatory factors in cell cycle progression (and/or mitochondrial function that then impacts on mitosis) through both catalytically dependent and independent mechanisms.

### 3.5. The Mitotic SIRT4 Interactome Comprises Microtubule-Associated Structural and Regulatory Proteins

**Figure 5:**
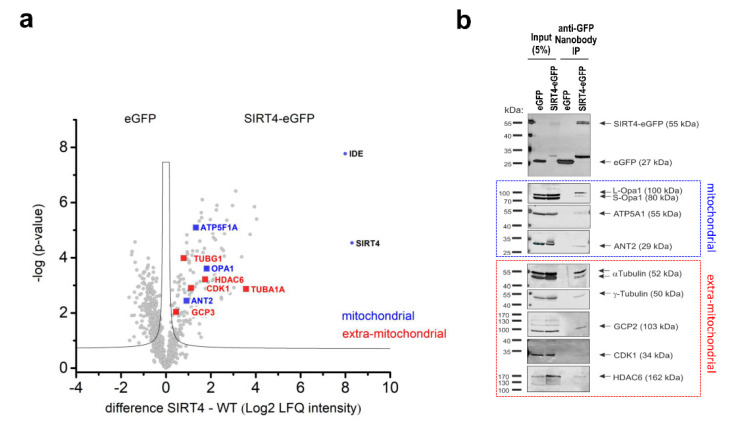
Analysis of the mitotic SIRT4 interactome by mass spectrometry. (**a**) Volcano plot analysis of mitochondrial and extra-mitochondrial proteins interacting with SIRT4. HEK293 cells stably expressing eGFP or SIRT4-eGFP (*n* = 4 replicates each) were arrested in G_2_ by the reversible CDK1 inhibitor RO3306 followed by release into mitosis (45 min after RO3306 wash out). Total cell lysates were subjected to anti-eGFP nanobody co-immunoprecipitations followed by their mass spectrometric analysis. (**b**) Anti-eGFP nanobody co-immunoprecipitations of selected mitochondrial and extra-mitochondrial SIRT4 interacting proteins as compared to eGFP controls. Total cell lysates analyzed in (**a**) were employed.

To better understand the mechanism(s) through which SIRT4 impacts on mitosis, we next analyzed the SIRT4 interactome in mitotic SIRT4-eGFP-expressing HEK293 cells as compared to eGFP-expressing control cells. Cells were synchronized in G_2_ by Ro3306-mediated, reversible inhibition of cyclin-dependent kinase 1 (CDK1) followed by release into mitosis for 45 min. Native SIRT4 containing protein complexes were isolated by anti-eGFP nanobody-based co-immunoprecipitation from total cell lysates followed by mass spectrometric characterization of SIRT4-interacting proteins (Table S1 and Figure S10). Protein network analyses revealed several known (e.g., DNA damage response [37]; mitochondrial respiratory chain components and glutamate metabolism regulators [34]; regulation of mitochondrial organization [42,48]) as well as novel functions and components associated with the mitotic SIRT4 interactome (e.g., tRNA aminoacylation and mitochondrial translation; cell cycle regulation; microtubule regulation) (Figure S10). In particular, as depicted in Figure 5, we identified several mitochondrial SIRT4-interacting proteins and potential substrates (OPA1 [42]; ATP5F1A [34]; ANT2 [21]; IDE [52]) as well as mostly novel, extra-mitochondrial localized SIRT4 interactors. The latter comprise α- and β-tubulin as subunits of microtubules, components of the centrosomally localized γTURC complex (γ-tubulin, TUBGCP2, TUBGCP3) [53,54] that nucleates microtubules at their minus poles, the microtubule deacetylase HDAC6 that is critically involved in the regulation of microtubule stability and dynamics [9], and the G_2_/M cell cycle regulator CDK1 [55]. These SIRT4 interactions were confirmed by nanobody-mediated co-immunoprecipitation (Figure 5b and Figure S11) and confocal colocalization analyses (Figure S11). The observed mitotic interaction pattern of SIRT4 appears specific, given that SIRT4 failed to co-immunoprecipitate with other centrosomal or mitotic spindle localized proteins like Pericentrin (Figure S12) or the TACC protein family member TACC3 [42], respectively.

### 3.6. SIRT4 Interacts with Microtubules and Negatively Regulates Acetyl-α-Tubulin (K40) Levels

**Figure 6:**
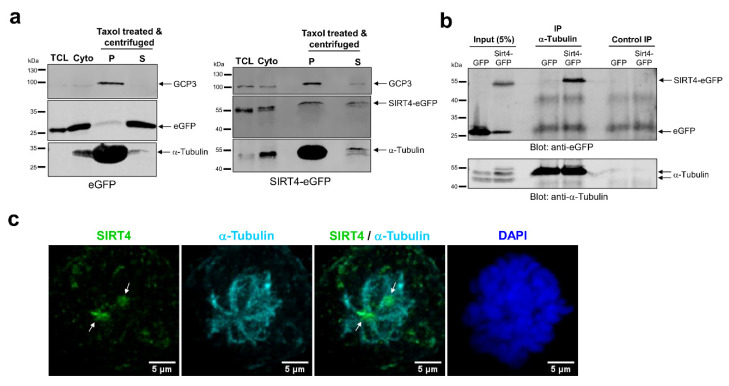
SIRT4 precipitates with microtubules and co-immunoprecipitates with α-tubulin in HEK293 cells. (**a**) SIRT4-eGFP, but not eGFP, is present in the pelleted fraction (P) of microtubules which were Taxol-stabilized in the cytosolic fraction (Cyto) followed by pelleting via centrifugation through a sucrose cushion. TCL, Total cell lysate; S, supernatant. Tubulin Gamma Complex Associated Protein 3 (TUBGCP3 or GCP3) was detected as co-marker for microtubules (**b**). An α-tubulin specific antibody co-immunoprecipitates SIRT4-eGFP, but not eGFP, from total cell lysates of stably transfected HEK293 cells. As control, immunoprecipitation without α-tubulin antibody was performed. (**c**) Localization of SIRT4 at spindle poles/Microtubule Organizing Centers (MTOCs) of mitotic HeLa cells using a polyclonal antibody against SIRT4 (sc-135053, Santa Cruz Biotechnology, Heidelberg, Germany) and analysis by spinning disk microscopy. Antibodies against α-Tubulin were employed to visualize microtubules. DAPI was used to detect DNA. Bar: 5 µm.

**Figure 7:**
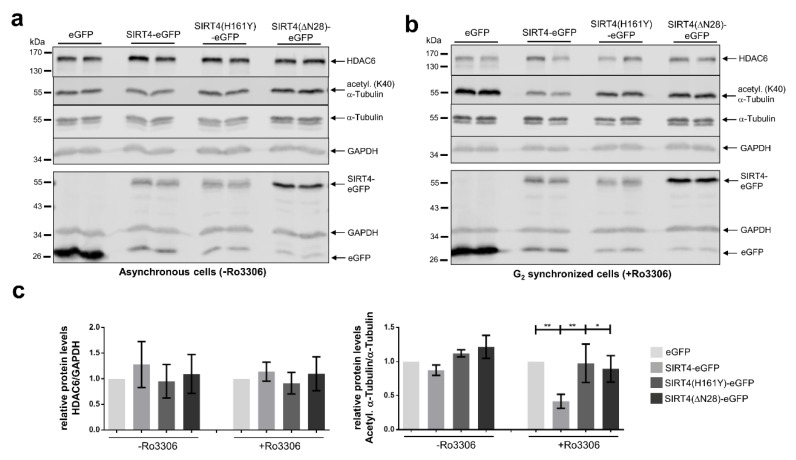
Ectopic expression of SIRT4 impacts negatively on acetylated α-tubulin (K40) during mitosis. HEK293 cells stably expressing eGFP, SIRT4-eGFP, or mutants thereof were either asynchronously grown (**a**) or subjected to Ro3306-mediated G_2_ synchronization (**b**) followed by immunoblot analysis of HDAC6, acetylated α-tubulin (K40), and α-tubulin. Expression of eGFP, SIRT4-eGFP, or mutants thereof was analyzed in a second immunoblot (lower panels in **a** and **b**). Probing against GAPDH was employed as loading control. Relative HDAC6 amounts and acetylated α-tubulin (K40)/α-tubulin levels were quantified by densitometric analysis (**c**). To evaluate statistical significance one-way ANOVA followed-up by Tukey’s test was performed (three independent experiments; * *p* < 0.05; ** *p* < 0.01). All P-values of the analysis of acetylated α-tubulin (K40)/α-tubulin levels (+Ro3306) refer to comparison with SIRT4-eGFP.

Given the link between SIRT4 and proteins of the microtubule network, we next addressed the interaction of SIRT4 with microtubules and its role in regulation of microtubule dynamics. We performed microtubule pulldown assays and observed SIRT4-eGFP in the pelleted fraction of Taxol-stabilized microtubules (Figure 6a). In contrast to this, eGFP as control was almost exclusively detected in the soluble fraction of Taxol-stabilized microtubules (Figure 6a). Consistent with these findings, (i) an α-tubulin specific antibody coimmunoprecipitated α-tubulin and SIRT4-eGFP, but not eGFP (Figure 6b), and (ii) α-tubulin co-localized with endogenous SIRT4 at MTOCs in mitotic cells as detected by spinning disk microscopy (Figure 6c). Given the putative protein interaction of SIRT4 with the microtubule deacetylase HDAC6 (Figure 5), we next analyzed the levels of K40-acetylated α-tubulin upon ectopic expression of SIRT4-eGFP or mutants thereof. Interestingly, as indicated in Figure 7, SIRT4-eGFP, but not the catalytically inactive mutant SIRT4(H161Y)-eGFP or SIRT4(ΔN28)-eGFP, led to a profound decrease in the ratio of K40-acetylated α-tubulin vs. total α-tubulin levels in G_2_ synchronized HEK293 cells as compared to asynchronously growing cells. Thus, our findings indicate that full-length SIRT4 impacts in an enzymatically dependent manner on microtubule dynamics in G_2_/M by decreasing microtubule stability, which presumably translates into inhibition of mitotic progression and proliferation (Figure 4).

## 4. Discussion

This study provides insights into a potential role of extra-mitochondrially localized SIRT4 in mitotic cell division. Our findings show that (i) SIRT4 localizes not only in mitochondria, but also in the cytosol, where it is found at centrosomes especially in early mitotic phases; (ii) as revealed by mass spectrometric, co-immunoprecipitation, and microtubule pulldown analyses, SIRT4 co-pellets with Taxol-stabilized microtubules and interacts with microtubule components, in particular α-tubulin, with components of the centrosome associated γTURC complex (γ-tubulin, GCP2, GCP3), and with the α-tubulin deacetylase HDAC6; (iii) linked to the SIRT4-HDAC6 interaction, increased SIRT4 expression results in decreased acetyl-α-tubulin (K40) levels, which are typically associated with decreased stability and altered dynamics of mitotic microtubules; (iv) at the cellular level, ectopic expression of SIRT4 or SIRT4(ΔN28) lacking the N-terminal mitochondrial targeting signal prolongs mitotic progression and inhibits cell proliferation. Consistent with the subcellular localization profile of endogenous SIRT4 and ectopically expressed SIRT4-eGFP (Figure 1 and Figure 2), it has been recently reported that ectopic SIRT4 expressed even at very low levels shows a dual localization in mitochondria as well as in the cytosol and nucleus. The authors attributed this to a low mitochondrial import kinetics of SIRT4 [36]. Thus, we propose that SIRT4 may exert its cell cycle inhibitory and tumor suppressor function through both mitochondria, i.e., bioenergetics-dependent, and mitochondria-independent, i.e., centrosome/mitotic spindle apparatus-linked mechanisms. Interestingly, the dual mitochondrial and centrosomal localization of SIRT4 (shown in this work) and its increased nuclear localization upon mitochondrial stress [36] is reminiscent of a function of SIRT4 as a “moonlighting protein” that per definition localizes at more than one cellular compartment/structure with similar or different functions [56]. Further examples of mitochondrially and centrosomal localized moonlighting proteins include C21orf33/GATD3A (glutamine amidotransferase like class 1 domain containing 3A), which has been identified within the human protein atlas project [57], and the mitochondrial porin VDAC3 (voltage-dependent anion-selective channel protein 3) that localizes at centrosomes and regulates centriole assembly [58].

In terms of the regulation of extramitochondrial SIRT4, we observed the highest centrosomal SIRT4 levels in G_2_ and early mitosis (Figure 1), indicating that centrosomal recruitment of SIRT4 and possibly its dissociation toward mitotic exit (Videos S2–S4) represents a regulated process. In contrast, total SIRT4 protein levels did not greatly change during cell cycle progression when cells were released from double thymidine block-mediated G_1_/S synchronization [59], although we cannot exclude that the cytosolic (i.e., extramitochondrial) pool of SIRT4 does. The low import kinetics of SIRT4 into mitochondria [36] could be further reduced during G_2_/M when mitochondria increasingly undergo cyclin B1-CDK1 driven fission [60] to become evenly distributed around the mitotic spindle [61] for cell division. Elevated cytosolic SIRT4 levels in G_2_/M might then result in increased recruitment of SIRT4 to the centrosome, a hypothesis that remains to be further tested.

A candidate regulator of centrosomal SIRT4 localization is CDK1, given that both proteins co-immunoprecipitate with each other (Figure 5) and partially colocalize at centrosomes in G_2_ [59]. Interestingly, an additional intramitochondrial role was uncovered for CDK1 where it interacts with and phosphorylates SIRT3 to enhance mitochondrial metabolism [62,63]. Given these findings it will be important to analyze the nature and function of a possible CDK1-SIRT4 axis in mitotic vs. non-mitotic cells. Overall, our findings add to the increasing evidence for a centrosomal localization of deacetylases (HDACs and sirtuins) [64] and their critical function(s) in centrosome biology, microtubule dynamics, and mitotic regulation. For example, the SIRT1-Plk2 (Polo-like kinase 2) and SIRT1-CCDC84-SAS6 axes control centriole duplication [65] and prevent centrosome overduplication [66], respectively. Expression of SIRT2, which is involved in the regulation of microtubule dynamics [8,10], is regulated in a cell cycle-dependent manner where SIRT2 localizes to centrosomes and the mitotic spindle [67]. Phosphorylation of SIRT2 by cyclin A-CDK2 reduces binding of SIRT2 to centrosomes and promotes G_2_/M progression [68]. In line with this, increased SIRT2 levels due to mitotic stress cause an extension of the mitotic phase [69] presumably through the regulatory role of SIRT2 toward the anaphase promoting factor/cyclosome (APC/C) and hence cyclin B1 degradation [70].

Our proteome analysis suggests a role of SIRT4 in the regulation of microtubule dynamics and function. SIRT4 precipitates with microtubules and co-immunoprecipitates with α-Tubulin (Figure 6). Here, SIRT4 may regulate the acetylation status and dynamics of microtubules which at least in part may mediate the inhibitory impact of ectopically expressed SIRT4 on cell division and proliferation (Figure 4), a hypothesis that remains to be further tested. Ectopic expression of SIRT4, but not the enzymatically inactive mutant SIRT4(H161Y), strongly inhibits the levels of acetylated α-tubulin (K40) in G_2_-synchronized HEK293 cells (Figure 7). The absent effect of SIRT4(ΔN28)-eGFP expression on K40-acetylated α-tubulin levels (Figure 7) was unexpected, given that SIRT4(ΔN28)-eGFP coimmunoprecipitates with α-tubulin (Figure S11). Thus, either SIRT4 requires its N-terminus for its extramitochondrial function toward modulation of K40-acetylated α-tubulin levels, or the mitochondrial import of SIRT4 is a prerequisite for its effects on acetylated α-tubulin (K40) levels. Alternatively, SIRT4 impacts through additional acetyl-α-tubulin (K40) independent mechanism(s) on euploidy (Figure S9) and mitotic progression and proliferation (Figure 4). The latter possibility is supported by the reduced, but still significant inhibitory impact of SIRT4(ΔN28) on mitotic duration and proliferation as compared to the full effect of wild-type SIRT4.

Another interaction partner of SIRT4 represents the deacetylase HDAC6 that was also identified as bona fide SIRT4 interactor in the proteomic screen by Mathias et al. [24]. HDAC6 is mainly found in the nucleus and cytosol and also localizes at the centrosome and basal body where it is involved in ciliary disassembly [71]. HDAC6 targets besides HSP90 and cortactin also K40-acetylated α-tubulin, resulting in decreased microtubule stability and altered microtubule dynamics [9,10]. It is currently unclear to which extent SIRT4 negatively regulates acetylated α-tubulin (K40) levels directly as deacetylase or indirectly via interaction with the known microtubule deacetylase HDAC6. The latter mechanism has been e.g., described for the tumor suppressor RITA (RBP-J and tubulin-associated protein) that interacts with HDAC6 and thereby modulates levels of K40-acetylated α-tubulin and microtubule dynamics [72].

Additional acetyl-α-tubulin (K40) independent mechanism(s) of SIRT4 in mitotic regulation may be based on other mitotic SIRT4 interaction partners. For example, SIRT4 also interacts (Figure 5 and Figure S11) and co-localizes (Figure S11) with γ-tubulin, TUBGCP2, and TUBGCP3, which represent core components of the γTURC. The latter is located at the outer region of the pericentriolar material (PCM) [73,74] and functions as nucleator of microtubules at their minus poles [53,54]. It remains to be determined whether SIRT4 regulates recruitment of the γTURC to centrosomes and/or its microtubule nucleation activity. However, besides phosphorylation, only few other post-translational modifications have been so far described for γTURC components, including an acetylation of GCP2 at Lys827 with currently unknown function [75,76]. Lastly, the partial localization of SIRT4-eGFP at the midzone of telophase cells and at the cytokinetic bridge (Video S4; [59]) could indicate a potential involvement of SIRT4 in cytokinesis. Interestingly, based on our proteomic screen (Table S1) SIRT4 may interact with RACK1 (Receptor of activated protein C kinase 1), that functions as major regulator of endosomal trafficking during cytokinesis [77,78].

The NAD^+^-SIRT3 axis has been also implicated in the regulation of microtubule dynamics and chromosomal alignment during mitosis [32,33]. However, this function of SIRT3 is likely mitochondrial-based, given that SIRT3 is predominantly found in mitochondria (Figure 3 and Figure S8) and neither localizes at centrosomes nor at the mitotic spindle (Figure S6).

Recent overviews of the literature revealed that SIRT4, although first described as a metabolic tumor suppressor [37], may display both tumor suppressor and oncogenic/cancer promoting activities, depending on the tumor type and checkpoint activating conditions [79,80]. Our data on the centrosomal localization of SIRT4 and a putative SIRT4-microtubule dynamics axis are rather consistent with an additional extramitochondrial tumor suppressor function of SIRT4. Consistent with this, SIRT4 protein levels increase not only upon treatment with DNA damaging agents effective in S-phase [81], but also through antimitotics like inhibitors of microtubule polymerization (Nocodazole or Vinblastine) or inhibition of microtubule dynamics (Paclitaxel) (Figure S13). Therefore, increased levels of SIRT4 may be critical to link mitotic stress to inhibition of cell proliferation as exemplified by ectopic expression of SIRT4-eGFP (Figure 4).

## 5. Conclusions

Our findings provide a first evidence suggesting that SIRT4 also takes over an extramitochondrial role in the regulation of mitotic cell division. Thus, stress-induced SIRT4 as in the case of DNA damage and senescence induction may exert its anti-proliferative role through both mitochondrial/metabolism dependent and mitochondria independent functions, the latter associated with its localization and function at the mitotic spindle apparatus.

## Supplementary Materials

The following including Supplementary Material and Methods and Supplementary References are available online at https://www.mdpi.com/2073-4409/9/9/1950/s1. Figure S1: Centrosomal localization of endogenous SIRT4 in HeLa cells as determined by single staining analysis. Figure S2: Centrosomal localization of endogenous SIRT4 in HT1080 cells as determined by single staining analysis. Figure S3: Centrosomal/mitotic spindle pole associated localization of endogenous SIRT4 in HeLa cells as determined by single staining analysis, Figure S4: Subcellular localization of SIRT4-eGFP (upper panels) and SIRT4(ΔN28)-eGFP (lower panels) in transiently transfected HT1080 fibrosarcoma cells (interphase) as imaged by spinning disk microscopy. Figure S5: Detection of SIRT4 at the mitotic spindle apparatus and in mitochondria. Figure S6: SIRT3 localizes in mitochondria, but is absent from centrosomes. Figure S7: Subcellular fractionation analysis of ectopically expressed SIRT4-eGFP in HEK293 cells, Figure S8: Subcellular fractionation analysis of ectopically expressed sirtuin proteins. Figure S9: HEK293 cells ectopically expressing SIRT4(ΔN28)-eGFP display an increased percentage of polyploidy. Figure S10: Network analysis of the SIRT4-interactom of mitotically synchronized HEK293 cells using the ClueGO software. Figure S11: SIRT4-eGFP interacts and subcellularly colocalizes with the γTUSC components GCP2 and GCP3. Figure S12: Anti-eGFP nanobody-based immunoprecipitation analysis of mitotic SIRT4-eGFP interactors. Figure S13: Mitotic stress leads to upregulation of SIRT4 protein levels. Table S1: Differential analysis of SIRT4-interacting proteins in mitotically synchronized HEK293 cells stably expressing SIRT4-eGFP as compared to eGFP expressing control cells. Table S2: List of antibodies used for immunoblot analysis. Table S3: List of antibodies used for confocal imaging analysis. Video S1: Subcellular localization of SIRT4(ΔN28)-eGFP in in transiently transfected HeLa cells (interphase) as imaged by spinning disk microscopy. Video S2: Subcellular localization of SIRT4-eGFP in transiently transfected HeLa cells (interphase) as imaged by spinning disk microscopy. Video S3: Subcellular localization of SIRT4-eGFP in transiently transfected HeLa cells (metaphase) as imaged by spinning disk microscopy. Video S4: Subcellular localization of SIRT4-eGFP in transiently transfected HeLa cells (telophase/cytokinesis) as imaged by spinning disk microscopy. Video S5: Subcellular localization of eGFP in transiently transfected HeLa cells (metaphase) as imaged by spinning disk microscopy.
