# Supplementary material for: Subcellular Localization and Mitotic Interactome Analyses Identify SIRT4 as a Centrosomally Localized and Microtubule Associated Protein
Source: Cells. 2020 Aug 24;9(9):1950. doi: 10.3390/cells9091950 (PMC7564595; doi:10.3390/cells9091950)
Supplement: Supplementary file 1 [file cells-09-01950-s001.zip › cells-830061-supplementary/suppl/Bergmann et al. _ Suppl. Figures_Cells_revised_final.pdf]

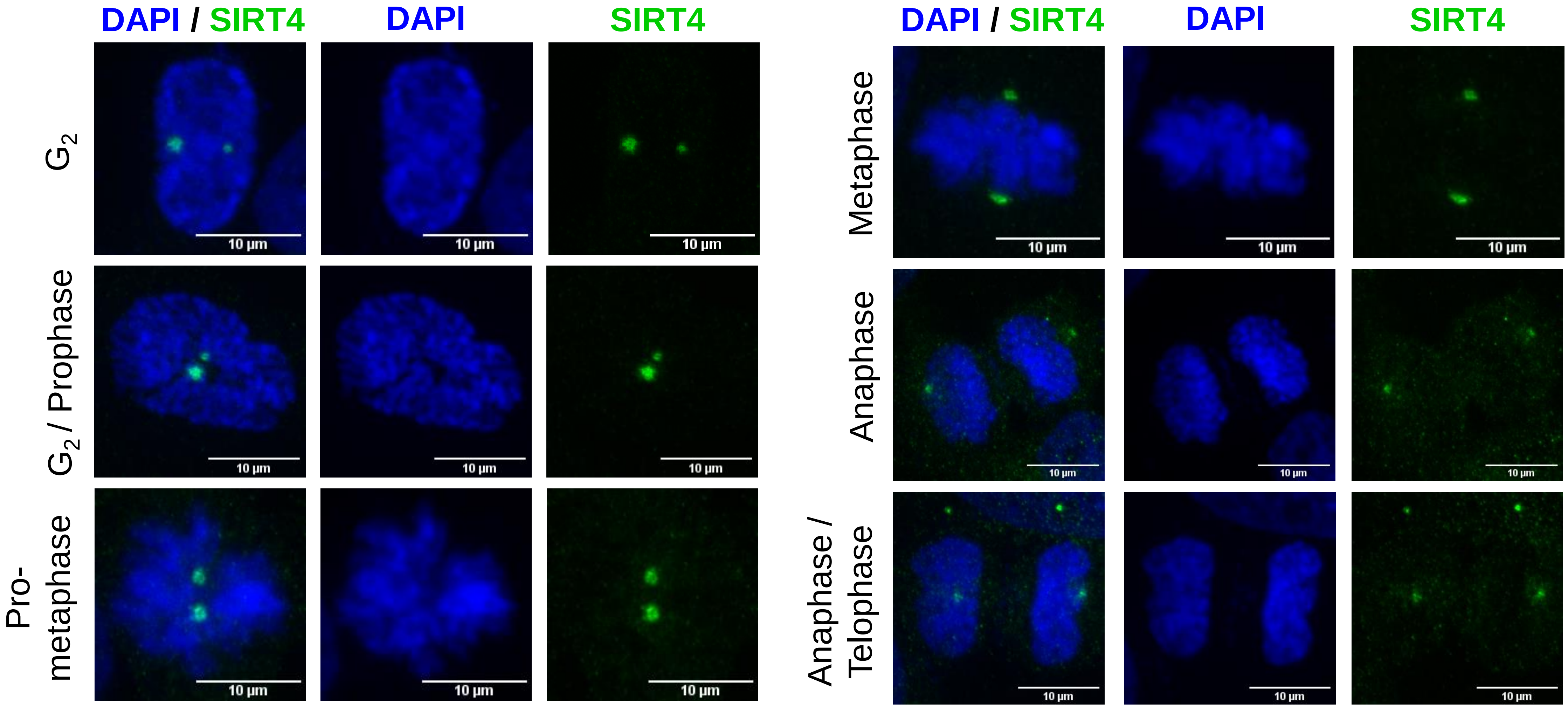
DAPI / SIRT4
DAPI
SIRT4
G2
G2 / Prophase
Pro-metaphase
Metaphase
Anaphase
Anaphase / Telophase
10 µm

Fig. S1

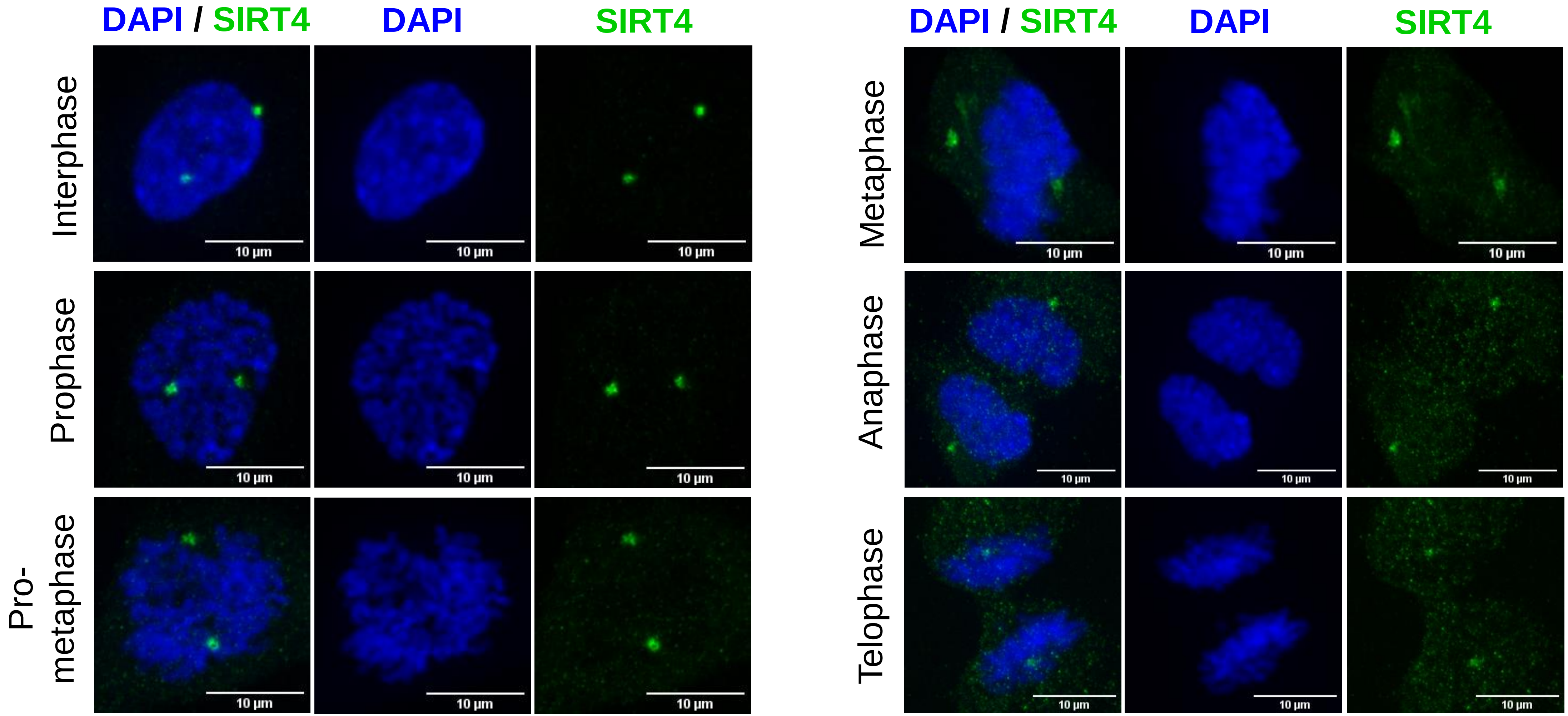
DAPI / SIRT4
DAPI
SIRT4
Interphase
Prophase
Pro-metaphase
Metaphase
Anaphase
Telophase
10 µm

Fig. S2

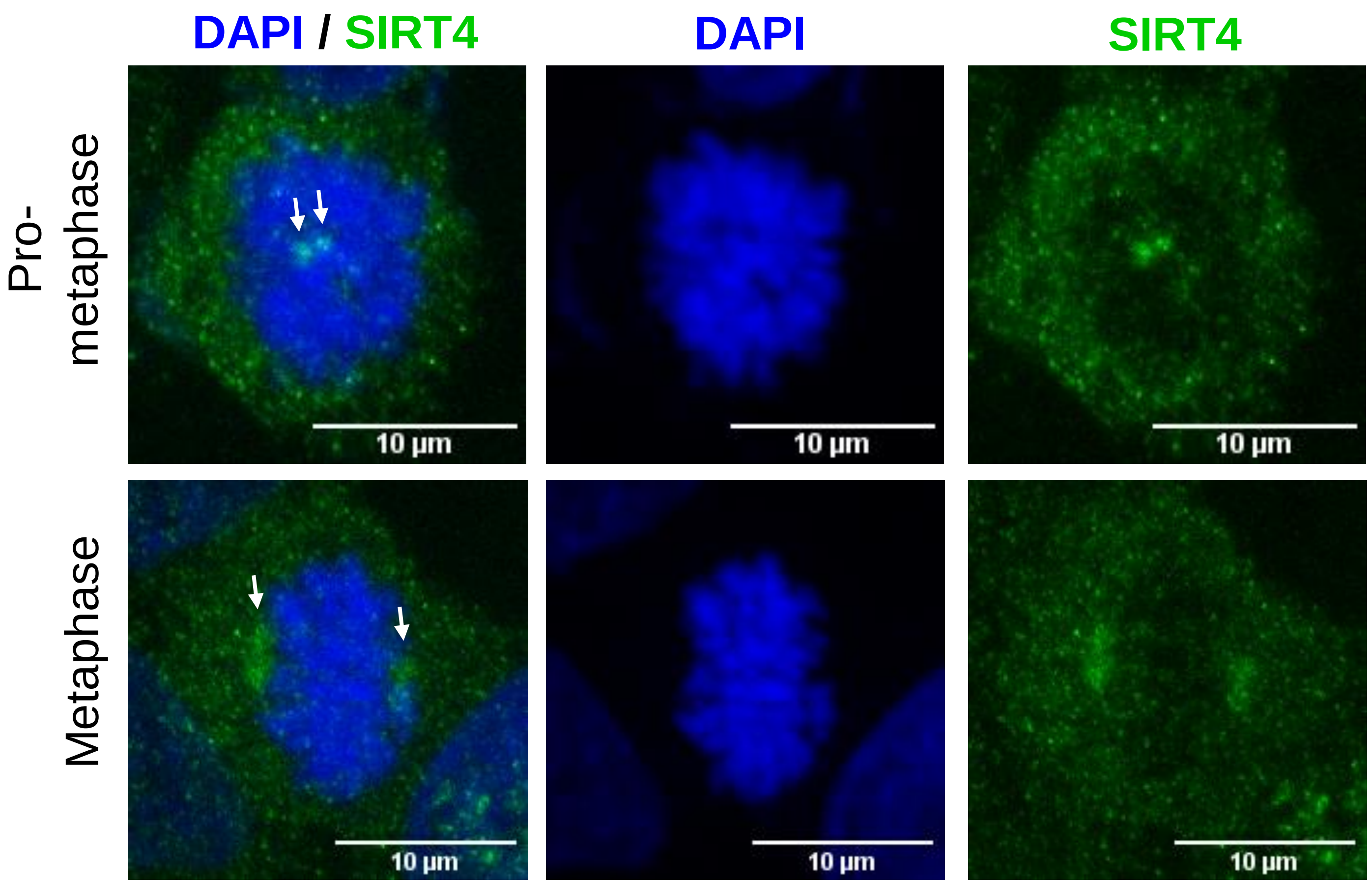

Fig. S3

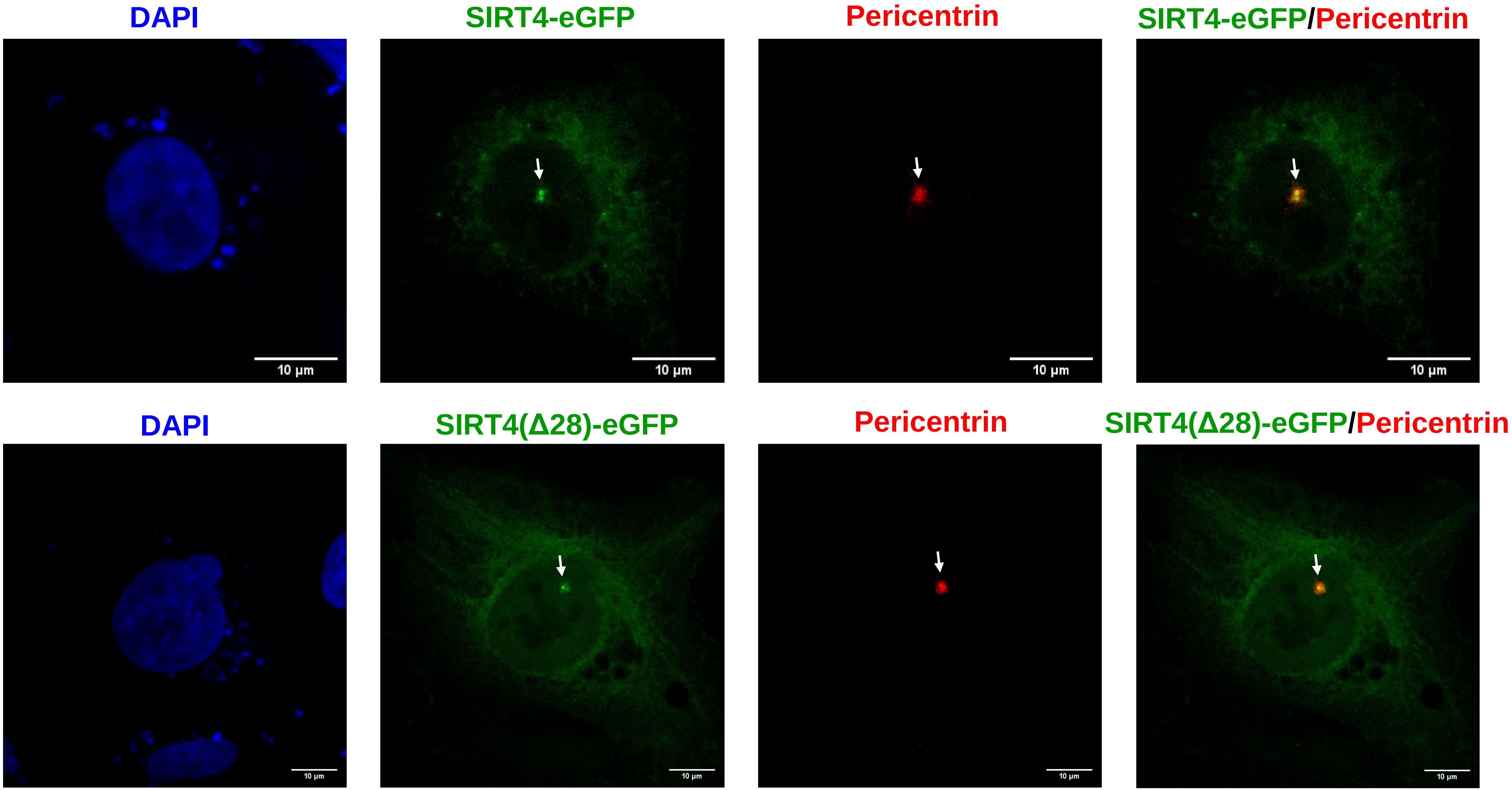
DAPI
SIRT4-eGFP
Pericentrin
SIRT4-eGFP/Pericentrin
10 µm
DAPI
SIRT4(Δ28)-eGFP
Pericentrin
SIRT4(Δ28)-eGFP/Pericentrin

Fig. S4

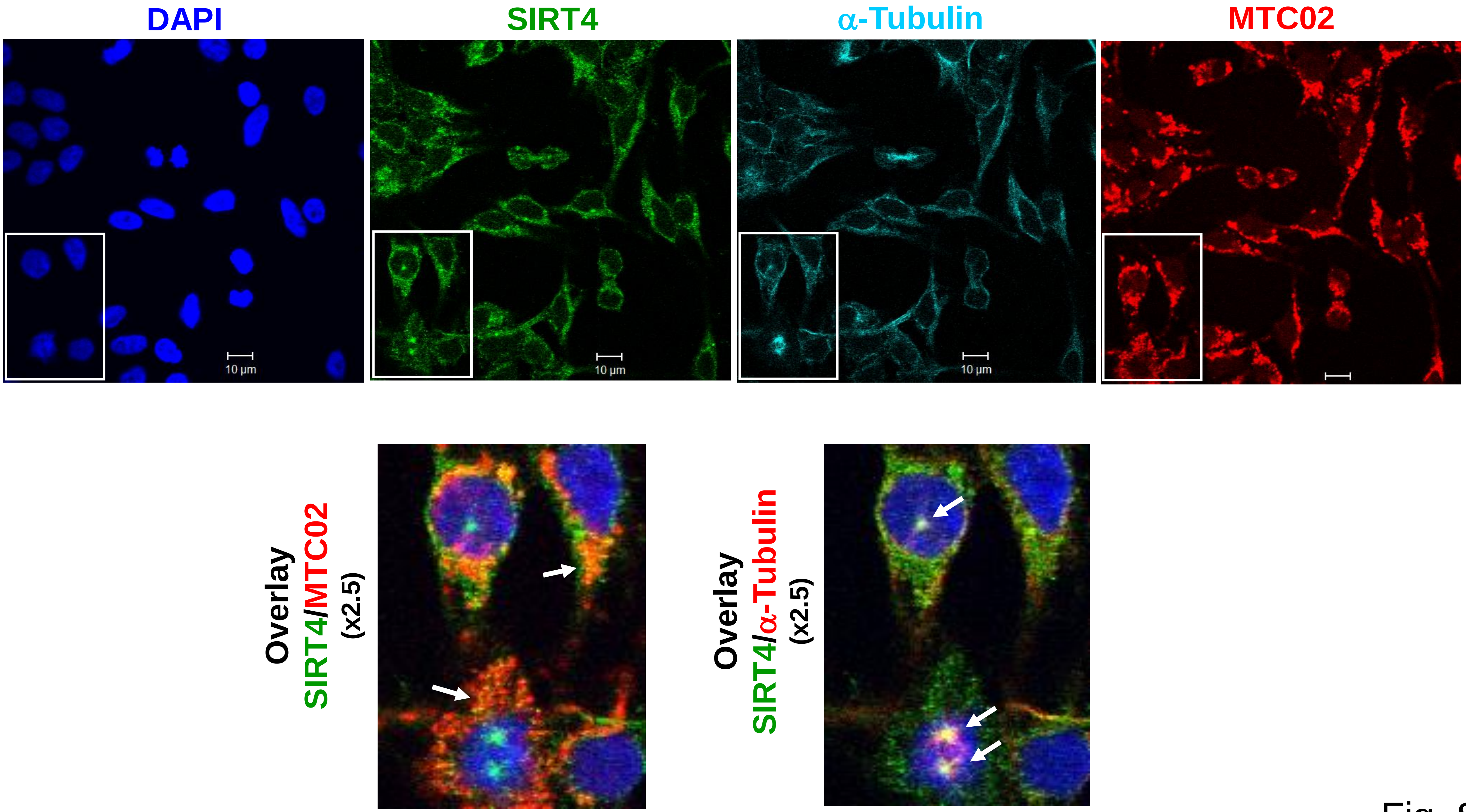

DAPI
SIRT4
α-Tubulin
MTC02
10 µm
10 µm
10 µm
Overlay
SIRT4/MTC02
(x2.5)
Overlay
SIRT4/α-Tubulin
(x2.5)

Fig. S5

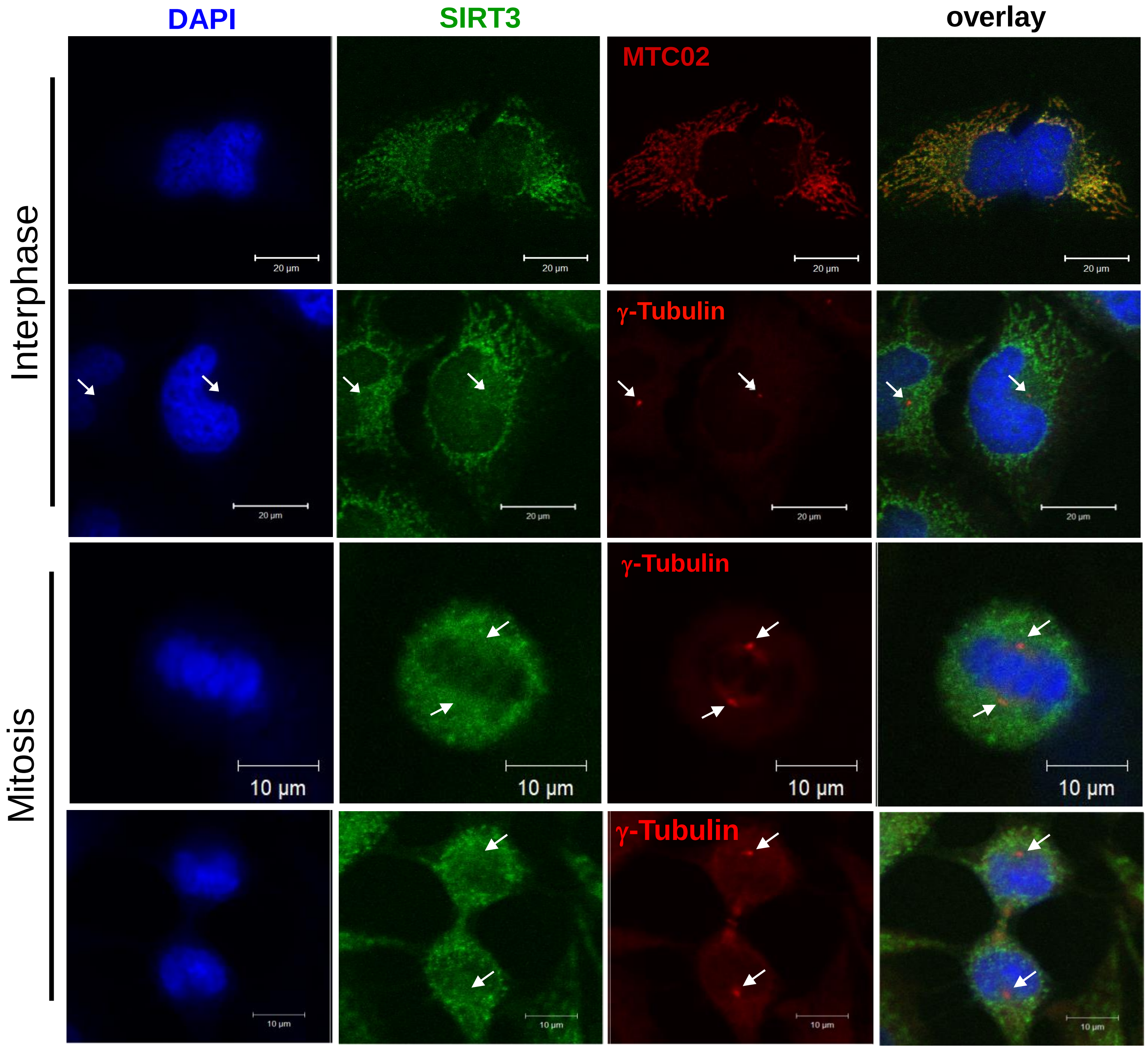
DAPI
SIRT3
overlay
MTC02
γ-Tubulin
γ-Tubulin
γ-Tubulin
Interphase
Mitosis
20 µm
10 µm

Fig. S6

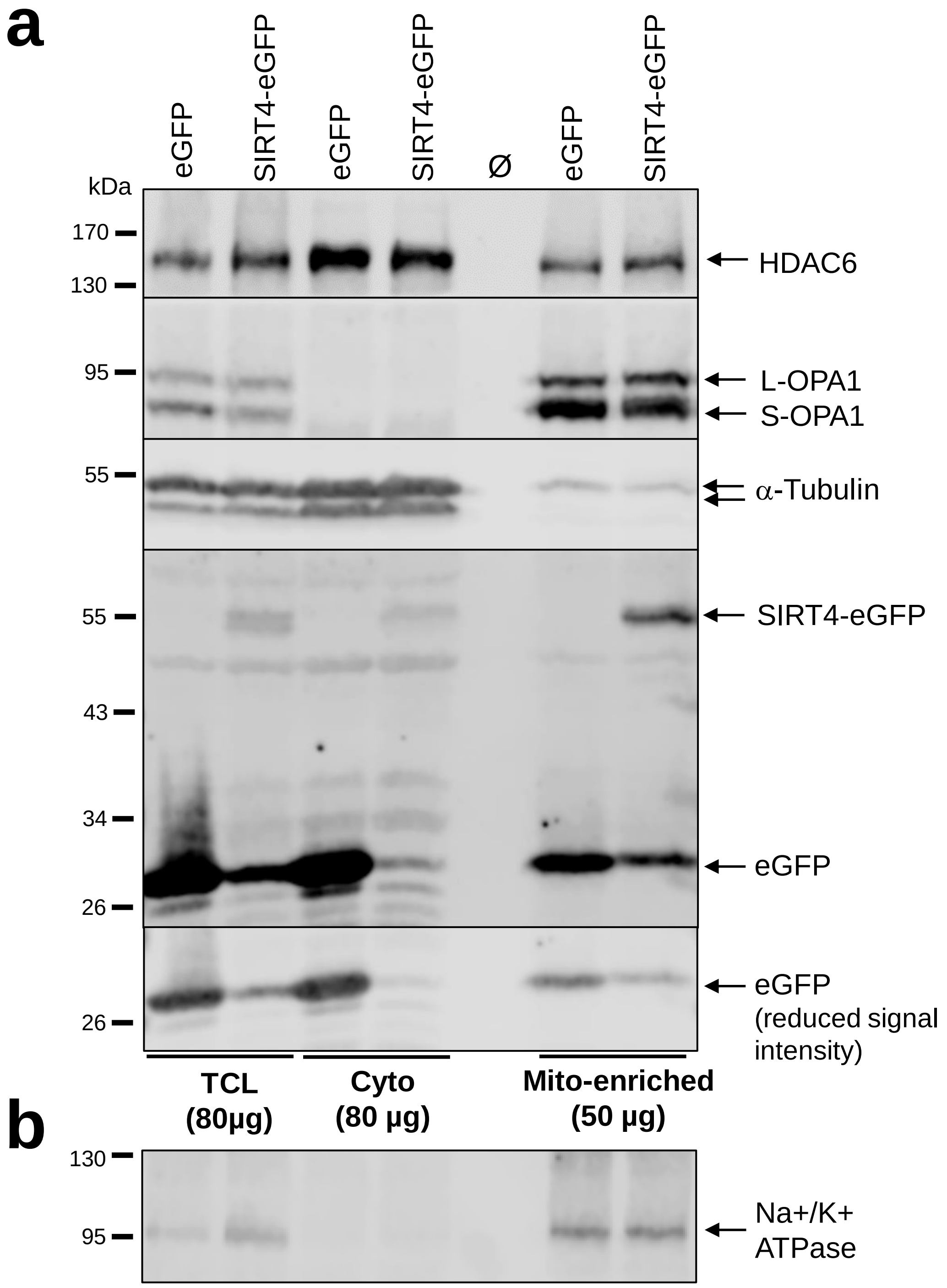
a
eGFP
SIRT4-eGFP
eGFP
SIRT4-eGFP
Ø
eGFP
SIRT4-eGFP
kDa
170
130
HDAC6
95
L-OPA1
S-OPA1
55
α-Tubulin
55
SIRT4-eGFP
43
34
eGFP
26
eGFP
(reduced signal intensity)
26
TCL (80μg)
Cyto (80 μg)
Mito-enriched (50 μg)
b
130
95
Na+/K+ ATPase

Fig. S7

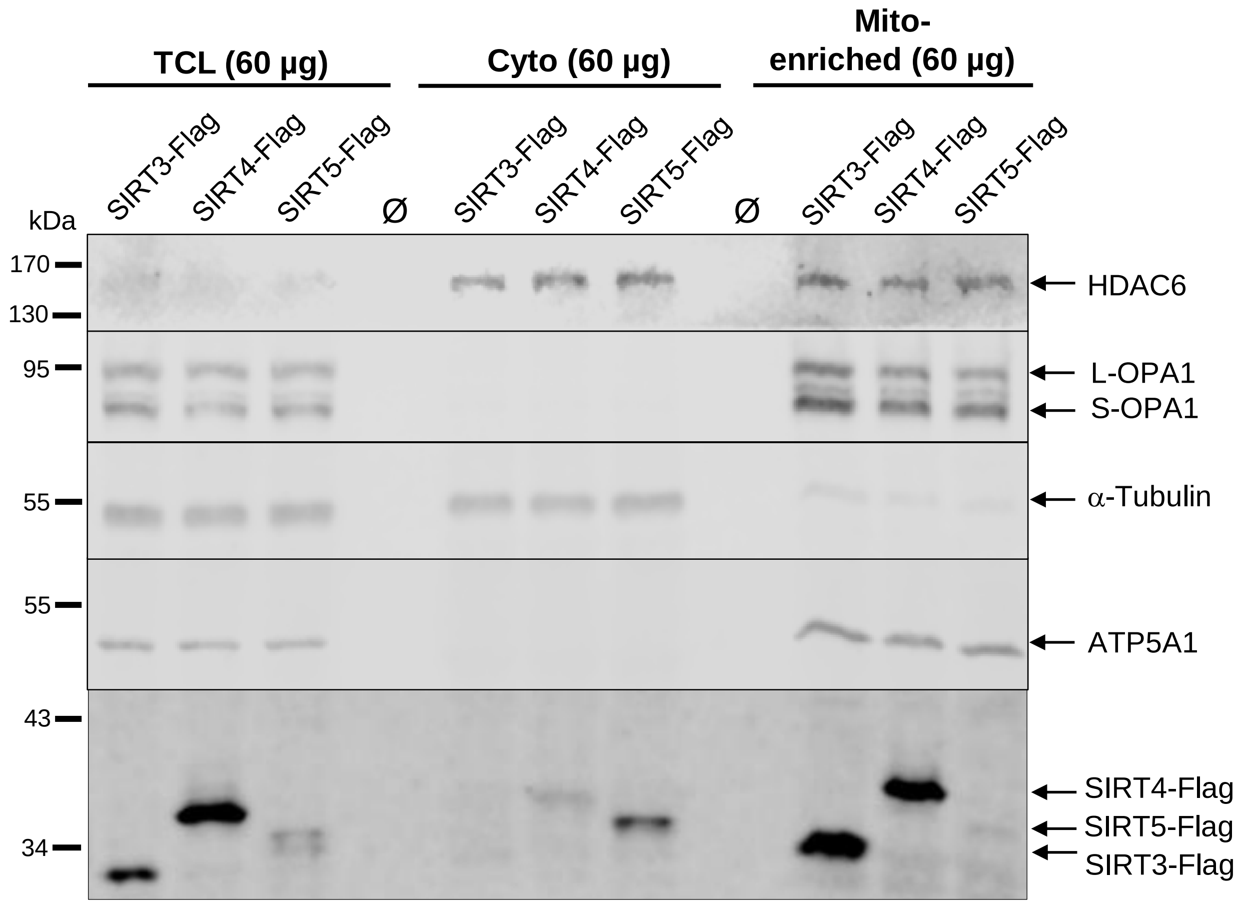
TCL (60 µg)
Cyto (60 µg)
Mito-
enriched (60 µg)
SIRT3-Flag
SIRT4-Flag
SIRT5-Flag
Ø
SIRT3-Flag
SIRT4-Flag
SIRT5-Flag
Ø
SIRT3-Flag
SIRT4-Flag
SIRT5-Flag
kDa
170
130
95
55
55
43
34
HDAC6
L-OPA1
S-OPA1
α-Tubulin
ATP5A1
SIRT4-Flag
SIRT5-Flag
SIRT3-Flag

Fig. S8

a

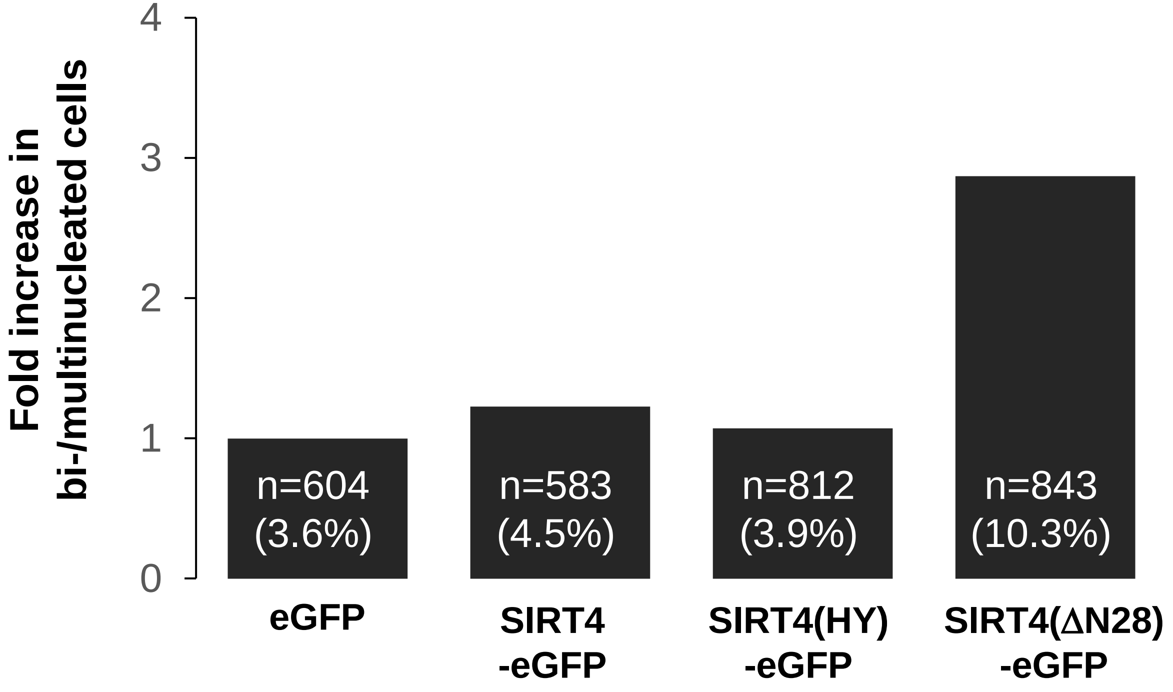
Fold increase in
bi-/multinucleated cells
4
3
2
1
0
n=604
(3.6%)
n=583
(4.5%)
n=812
(3.9%)
n=843
(10.3%)
eGFP
SIRT4
-eGFP
SIRT4(HY)
-eGFP
SIRT4(ΔN28)
-eGFP

b

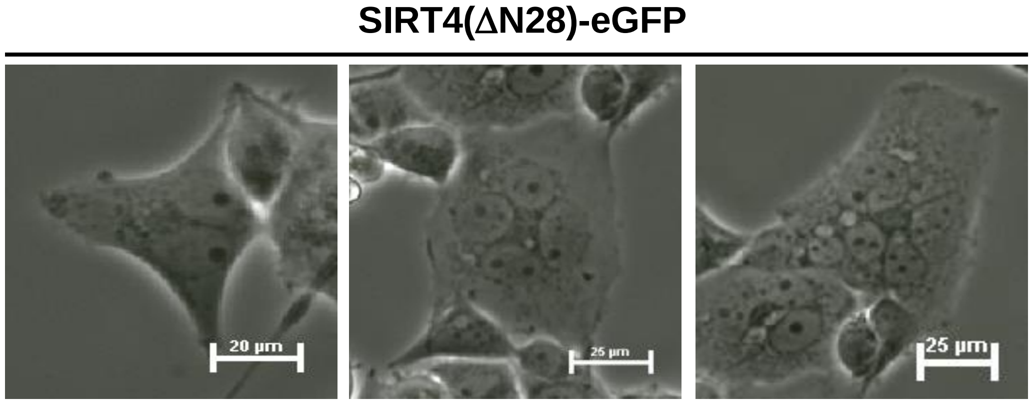
SIRT4(ΔN28)-eGFP
20 µm
25 µm
25 µm

Fig. S9

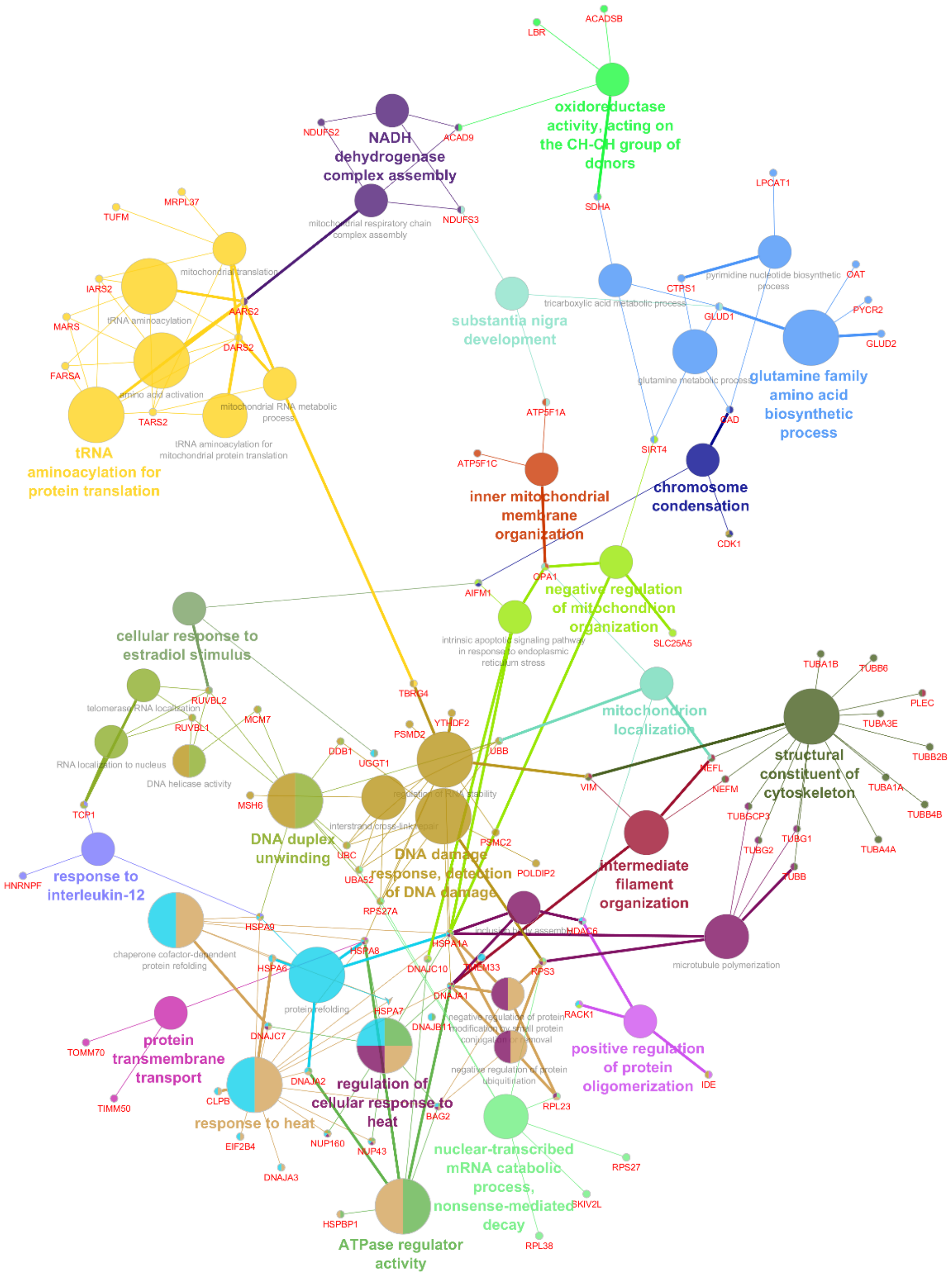
oxidoreductase activity, acting on the CH-CH group of donors
NADH dehydrogenase complex assembly
mitochondrial respiratory chain complex assembly
mitochondrial translation
tRNA aminoacylation
amino acid activation
mitochondrial RNA metabolic process
tRNA aminoacylation for mitochondrial protein translation
tRNA aminoacylation for protein translation
substantia nigra development
tricarboxylic acid metabolic process
pyrimidine nucleotide biosynthetic process
glutamine metabolic process
glutamine family amino acid biosynthetic process
inner mitochondrial membrane organization
chromosome condensation
negative regulation of mitochondrion organization
intrinsic apoptotic signaling pathway in response to endoplasmic reticulum stress
cellular response to estradiol stimulus
telomerase RNA localization
RNA localization to nucleus
DNA helicase activity
mitochondrion localization
structural constituent of cytoskeleton
DNA duplex unwinding
interstrand cross-link repair
regulation of mRNA stability
DNA damage response, detection of DNA damage
intermediate filament organization
response to interleukin-12
chaperone cofactor-dependent protein refolding
protein refolding
inclusion body assembly
microtubule polymerization
negative regulation of protein modification by small protein conjugation or removal
negative regulation of protein ubiquitination
protein transmembrane transport
positive regulation of protein oligomerization
regulation of cellular response to heat
response to heat
nuclear-transcribed mRNA catabolic process, nonsense-mediated decay
ATPase regulator activity
LBR
ACADSB
NDUFS2
ACAD9
SDHA
NDUFS3
LPCAT1
TUFM
MRPL37
IARS2
MARS
FARSA
AARS2
DARS2
TARS2
CTPS1
OAT
PYCR2
GLUD1
GLUD2
CAD
ATP5F1A
ATP5F1C
SIRT4
CDK1
OPA1
AIFM1
SLC25A5
TUBA1B
TUBB6
PLEC
TUBA3E
TUBB2B
TUBA1A
TUBB4B
TUBA4A
NEFL
NEFM
TUBGCP3
TUBG1
TUBG2
TUBB
VIM
RUVBL2
RUVBL1
MCM7
TBRG4
YTHDF2
PSMD2
DDB1
UGGT1
UBB
MSH6
TCP1
UBC
UBA52
PSMC2
POLDIP2
RPS27A
HNRNPF
HSPA9
HSPA1A
HDAC6
HSPA8
HSPA6
DNAJC10
TMEM33
RPS3
DNAJA1
HSPA7
RACK1
DNAJB1
DNAJC7
TOMM70
TIMM50
DNAJA2
CLPB
IDE
BAG2
RPL23
NUP160
NUP43
EIF2B4
DNAJA3
RPS27
SKIV2L
HSPBP1
RPL38

Fig. S10

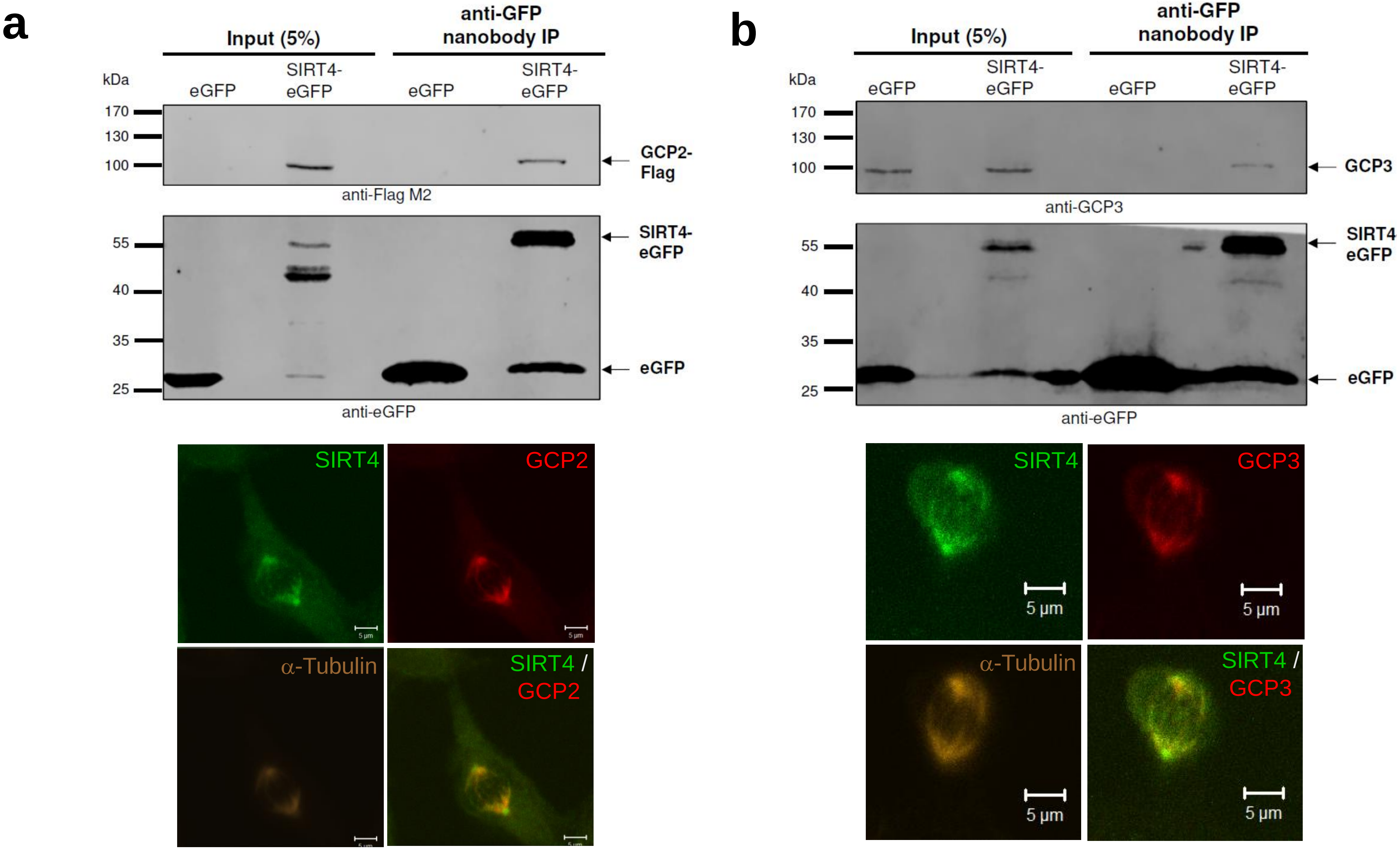
a
Input (5%)
anti-GFP nanobody IP
kDa
eGFP
SIRT4-eGFP
eGFP
SIRT4-eGFP
170
130
100
GCP2-Flag
anti-Flag M2
55
40
35
25
SIRT4-eGFP
eGFP
anti-eGFP
SIRT4
GCP2
α-Tubulin
SIRT4 / GCP2
b
Input (5%)
anti-GFP nanobody IP
kDa
eGFP
SIRT4-eGFP
eGFP
SIRT4-eGFP
170
130
100
GCP3
anti-GCP3
55
40
35
25
SIRT4 eGFP
eGFP
anti-eGFP
SIRT4
GCP3
5 µm
α-Tubulin
SIRT4 / GCP3

Fig. S11

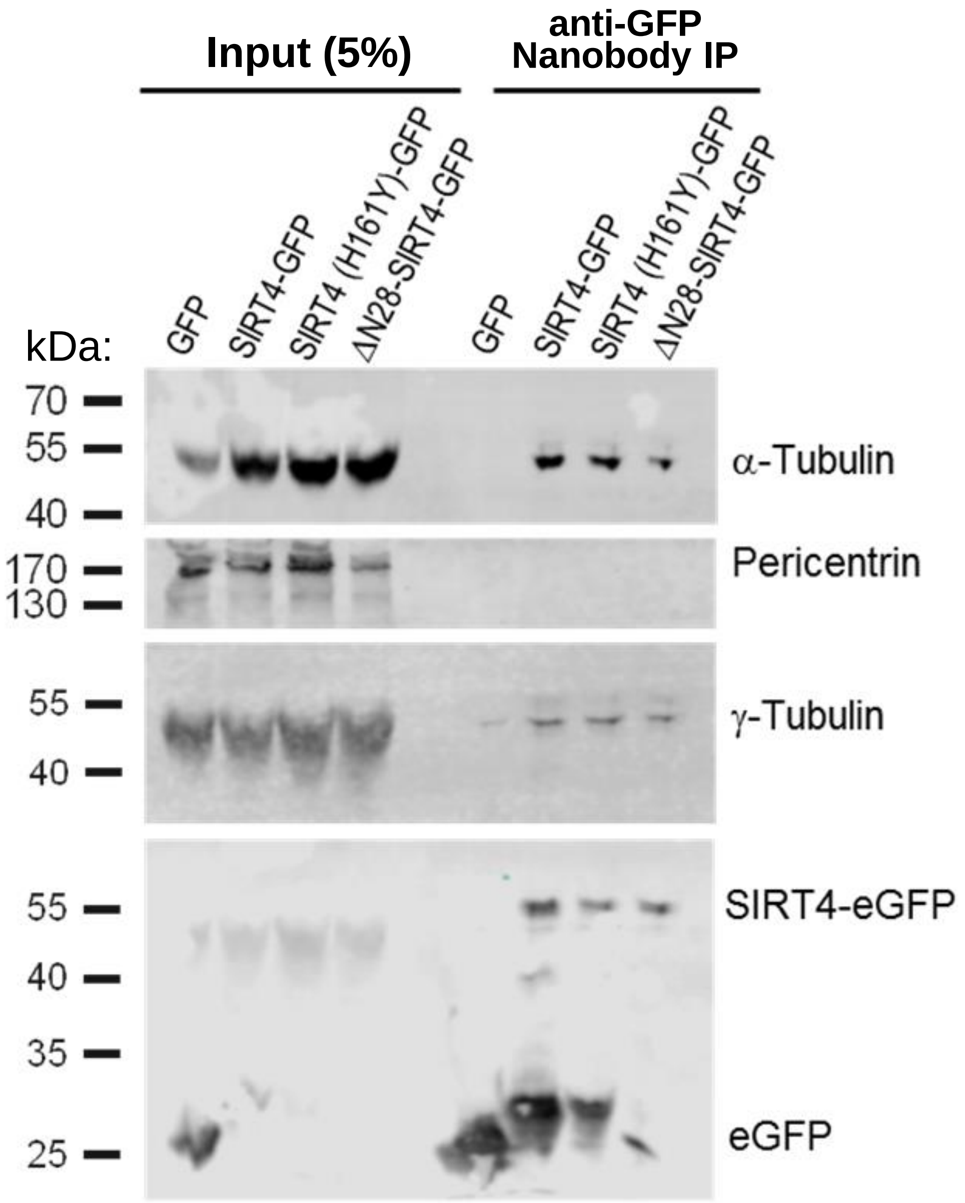
Input (5%)
anti-GFP
Nanobody IP
GFP
SIRT4-GFP
SIRT4 (H161Y)-GFP
ΔN28-SIRT4-GFP
GFP
SIRT4-GFP
SIRT4 (H161Y)-GFP
ΔN28-SIRT4-GFP
kDa:
70
55
40
α-Tubulin
170
130
Pericentrin
55
40
γ-Tubulin
55
40
35
25
SIRT4-eGFP
eGFP

Fig. S12

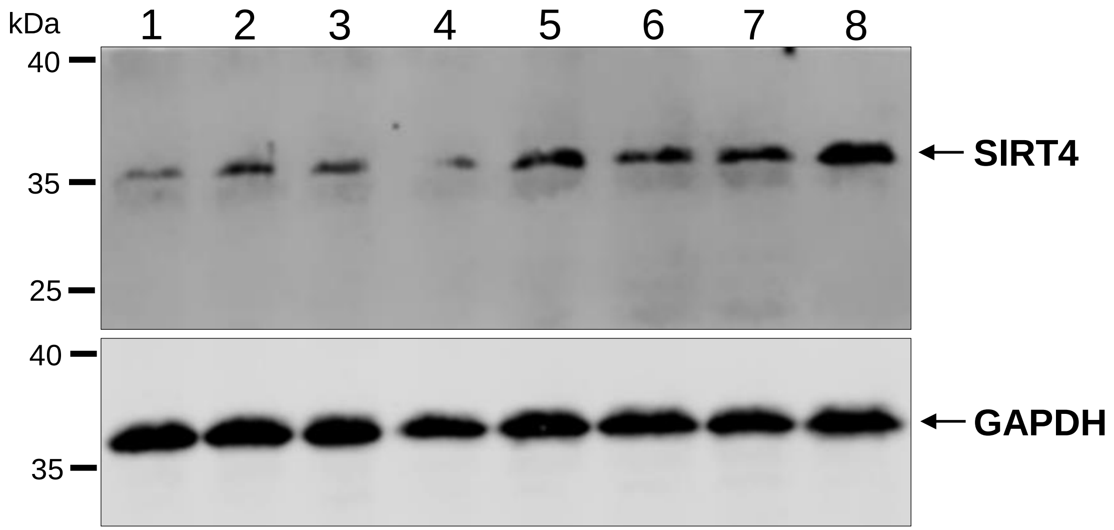

Fig. S13
