# Supplementary material for: Subcellular Localization and Mitotic Interactome Analyses Identify SIRT4 as a Centrosomally Localized and Microtubule Associated Protein
Source: Cells. 2020 Aug 24;9(9):1950. doi: 10.3390/cells9091950 (PMC7564595; doi:10.3390/cells9091950)
Supplement: Supplementary file 1 [file cells-09-01950-s001.zip › cells-830061-supplementary/suppl/Table S1_mass spectrometry of SIRT4-eGFP interactors_corrected.pdf]

Table S1. Mass spectrometric analysis of SIRT4-eGFP interacting proteins.

Significant	Protein IDs	Majority protein IDs	Score	Intensity	MS/MS Coun	Unique pepti	t-test p value	t-test Difference	eGFP #1	eGFP #2	eGFP #3	eGFP #4	SIRT4-eGFP #1	SIRT4-eGFP #2	SIRT4-eGFP #3	SIRT4-eGFP #4
+	sp|P14735|IDE_HUMAN;tr|Q5T5N3|Q5T5N3_HUMAN	sp|P14735|IDE_HUMAN	323,31	1802900000	83	32	1,69E-08	7,993219852	19,48581	19,60563	19,44071	19,65087	28,00426	27,28982	27,70827	27,15354
+	sp|P68363|TBA1B_HUMAN;sp|P68366|TBA4A_HUMAN;tr|F8VVB9|F8VVB9_HUMAN;tr|C9JDS9	sp|P68363|TBA1B_HUMAN;sp|P68366|TBA4A_HUMAN;tr|F8VVB9|F8VVB9_HUMAN	323,31	3,2565E+10	200	1	3,85E-07	3,111667633	28,28776	28,52772	28,51608	28,53271	31,82491	31,54324	31,67405	31,26874
+	sp|P0DMV9|HS71B_HUMAN;sp|P0DMV8|HS71A_HUMAN;tr|A0A0G2JIW1|A0A0G2JIW1_HUMA	sp|P0DMV9|HS71B_HUMAN;sp|P0DMV8|HS71A_HUMAN;tr|A0A0G2JIW1|A0A0G2JIW1_HUMA	323,31	2,5768E+10	306	25	7,88E-07	1,624051094	29,38527	29,33745	29,55784	29,63843	31,19601	31,05692	31,06561	31,09666
+	sp|Q9BW92|SYTM_HUMAN;tr|U3KQG0|U3KQG0_HUMAN;tr|F6S7Q7|F6S7Q7_HUMAN;tr|U3K(	sp|Q9BW92|SYTM_HUMAN;tr|U3KQG0|U3KQG0_HUMAN;tr|F6S7Q7|F6S7Q7_HUMAN	24,499	74934000	19	9	1,28E-06	2,275695324	20,70261	20,54482	20,4247	20,40291	22,77125	22,61993	23,06733	22,71931
+	tr|Q5JP53|Q5JP53_HUMAN;sp|P07437|TBB5_HUMAN;tr|Q5ST81|Q5ST81_HUMAN	tr|Q5JP53|Q5JP53_HUMAN;sp|P07437|TBB5_HUMAN;tr|Q5ST81|Q5ST81_HUMAN	323,31	2,5329E+10	152	6	1,28E-06	2,972658634	27,77052	28,1757	28,10993	28,23339	31,29844	31,07304	31,06728	30,74142
+	sp|P31689|DNJA1_HUMAN	sp|P31689|DNJA1_HUMAN	242,01	418600000	29	10	1,43E-06	3,943796635	21,55037	21,45874	21,83082	20,9239	25,51926	25,47636	25,41471	25,12869
+	sp|P49411|EFTU_HUMAN;tr|H3BNU3|H3BNU3_HUMAN	sp|P49411|EFTU_HUMAN	323,31	2997800000	122	27	1,85E-06	2,245566845	25,73229	25,85262	25,82092	26,05935	28,26526	28,08851	28,26535	27,82833
+	sp|Q8NB90|SPAT5_HUMAN;tr|J3QRR3|J3QRR3_HUMAN;tr|J3QRW1|J3QRW1_HUMAN	sp|Q8NB90|SPAT5_HUMAN	25,333	48934000	18	3	2,33E-06	1,578964233	20,05126	20,12994	20,14805	20,23876	21,70438	21,80963	21,87279	21,49707
+	tr|A0A087WXZ3|A0A087WXZ3_HUMAN;sp|Q9Y5A9|YTHD2_HUMAN;tr|A0A024R7W5|A0A024R	tr|A0A087WXZ3|A0A087WXZ3_HUMAN;sp|Q9Y5A9|YTHD2_HUMAN	47,221	121610000	24	7	3,58E-06	1,59357357	21,72146	21,7348	21,76052	21,8055	23,46936	23,23732	23,55192	23,13798
+	sp|O60884|DNJA2_HUMAN;tr|A0A087WT48|A0A087WT48_HUMAN;tr|I3L320|I3L320_HUMAN	sp|O60884|DNJA2_HUMAN	150,4	269080000	32	11	4,01E-06	4,039155483	20,80381	21,07437	20,64157	20,19928	25,06139	24,84024	24,74842	24,2256
+	sp|P07196|NFL_HUMAN;tr|A0A087X0W2|A0A087X0W2_HUMAN	sp|P07196|NFL_HUMAN;tr|A0A087X0W2|A0A087X0W2_HUMAN	323,31	4549900000	94	30	4,17E-06	1,980173588	26,7226	26,70859	26,81164	26,56725	28,94999	28,54709	28,79129	28,44241
+	sp|P27708|PYR1_HUMAN;tr|F8VPD4|F8VPD4_HUMAN;tr|H7C2E4|H7C2E4_HUMAN;tr|H7BZB3	sp|P27708|PYR1_HUMAN;tr|F8VPD4|F8VPD4_HUMAN	323,31	2539900000	170	61	4,42E-06	2,714831352	25,31614	25,33006	25,06435	24,873	27,60915	27,71914	28,22986	27,88472
+	sp|P33993|MCM7_HUMAN;tr|C9J8M6|C9J8M6_HUMAN	sp|P33993|MCM7_HUMAN	271,35	437040000	64	20	4,70E-06	1,488797665	23,98129	23,64482	23,73738	23,64471	25,33217	25,0857	25,24449	25,30103
+	sp|Q14257|RCN2_HUMAN;tr|H0YL43|H0YL43_HUMAN;tr|A8MXP8|A8MXP8_HUMAN	sp|Q14257|RCN2_HUMAN;tr|H0YL43|H0YL43_HUMAN;tr|A8MXP8|A8MXP8_HUMAN	125,94	71262000	22	8	6,42E-06	2,099233627	20,38732	20,69634	20,36196	20,74796	22,84169	22,49045	22,80499	22,4534
+	sp|P30837|AL1B1_HUMAN;tr|A0A0U1RQK9|A0A0U1RQK9_HUMAN;tr|H0Y2X5|H0Y2X5_HUMA	sp|P30837|AL1B1_HUMAN	39,398	72075000	19	7	6,50E-06	1,663305283	20,81495	20,7913	20,91911	21,2237	22,45959	22,71597	22,65679	22,56994
+	tr|A0A087WTA5|A0A087WTA5_HUMAN;sp|Q9UI10|EI2BD_HUMAN;tr|E7ERK9|E7ERK9_HUMAI	tr|A0A087WTA5|A0A087WTA5_HUMAN;sp|Q9UI10|EI2BD_HUMAN;tr|E7ERK9|E7ERK9_HUMAI	13,68	59907000	14	8	6,76E-06	1,781701565	20,73983	20,46573	20,7978	20,28974	22,27989	22,36268	22,34852	22,42882
+	tr|E9PNM1|E9PNM1_HUMAN;sp|P37268|FDFT_HUMAN;tr|E9PS69|E9PS69_HUMAN;tr|E9PJG4	tr|E9PNM1|E9PNM1_HUMAN;sp|P37268|FDFT_HUMAN	27,06	72029000	19	8	7,32E-06	3,003790538	19,70889	NaN	20,02715	19,68522	23,08601	22,57815	22,85719	22,72217
+	sp|Q9BSD7|NTPCR_HUMAN;tr|Q5TDF0|Q5TDF0_HUMAN	sp|Q9BSD7|NTPCR_HUMAN;tr|Q5TDF0|Q5TDF0_HUMAN	65,852	68051000	26	9	7,58E-06	2,808207035	19,2954	19,87048	19,74991	19,9502	22,70094	22,80211	22,31614	22,27963
+	sp|O95831|AIFM1_HUMAN;tr|E9PMA0|E9PMA0_HUMAN	sp|O95831|AIFM1_HUMAN	38,175	121380000	24	8	7,90E-06	1,344748974	22,10008	21,88248	22,05142	22,07535	23,48542	23,14781	23,49997	23,35515
+	sp|P25705|ATPA_HUMAN;tr|K7ERX7|K7ERX7_HUMAN;tr|K7EK77|K7EK77_HUMAN;tr|K7EJP1|I	sp|P25705|ATPA_HUMAN	323,31	1097300000	80	17	8,03E-06	1,334028721	25,23004	25,25469	25,04955	25,23213	26,63616	26,39121	26,69564	26,37951
+	sp|Q9H857|NT5D2_HUMAN;tr|H7C519|H7C519_HUMAN	sp|Q9H857|NT5D2_HUMAN	18,811	67755000	15	2	1,31E-05	2,08548069	20,37231	20,61342	20,60494	20,2674	22,18876	22,73074	22,78738	22,49311
+	sp|Q3ZCQ8|TIM50_HUMAN;tr|M0R0C3|M0R0C3_HUMAN;tr|M0R2F8|M0R2F8_HUMAN;tr|M0I	sp|Q3ZCQ8|TIM50_HUMAN	270,17	180060000	29	7	1,32E-05	2,610735416	21,10399	21,546	21,53621	21,6413	24,38383	24,18064	24,08958	23,61639
+	sp|O95816|BAG2_HUMAN	sp|O95816|BAG2_HUMAN	55,859	208970000	16	5	1,42E-05	3,751319885	20,41648	20,74854	20,43166	20,679	24,65992	24,78756	24,29866	23,53481
+	sp|Q9Y230|RUVB2_HUMAN;tr|M0R0Y3|M0R0Y3_HUMAN;tr|X6R2L4|X6R2L4_HUMAN;tr|M0Q)	sp|Q9Y230|RUVB2_HUMAN;tr|M0R0Y3|M0R0Y3_HUMAN	323,31	659030000	65	18	1,50E-05	0,935032845	24,79996	24,67336	24,80538	24,83123	25,89349	25,59024	25,659	25,70733
+	tr|A0A087WTV6|A0A087WTV6_HUMAN;sp|Q96C36|P5CR2_HUMAN;tr|A0A087WZR9|A0A087V	tr|A0A087WTV6|A0A087WTV6_HUMAN;sp|Q96C36|P5CR2_HUMAN;tr|A0A087WZR9|A0A087V	49,366	166030000	24	9	1,84E-05	2,117117882	21,41085	21,78624	21,78417	22,19436	24,05904	23,96526	23,87615	23,74366
+	sp|P42677|RS27_HUMAN;tr|Q5T4L4|Q5T4L4_HUMAN;tr|C9J1C5|C9J1C5_HUMAN	sp|P42677|RS27_HUMAN;tr|Q5T4L4|Q5T4L4_HUMAN	115,25	753980000	18	3	2,21E-05	2,211357117	23,95314	24,00813	23,62879	23,81636	26,55249	25,87077	25,99561	25,83297
+	sp|Q9BZE1|RM37_HUMAN;tr|S4R369|S4R369_HUMAN;tr|H0Y4J2|H0Y4J2_HUMAN;tr|A6NHR2	sp|Q9BZE1|RM37_HUMAN;tr|S4R369|S4R369_HUMAN	3,466	20280000	9	3	2,51E-05	1,512530804	19,01822	18,8462	19,25776	19,17473	20,66891	20,74098	20,31385	20,62328
+	tr|A0A0C4DGL3|A0A0C4DGL3_HUMAN;tr|H0YNW5|H0YNW5_HUMAN;tr|H0YKC5|H0YKC5_HUI	tr|A0A0C4DGL3|A0A0C4DGL3_HUMAN;tr|H0YNW5|H0YNW5_HUMAN;tr|H0YKC5|H0YKC5_HUI	45,802	100110000	24	6	2,69E-05	-1,372592926	23,21444	23,35259	23,33211	23,5836	22,04597	22,18414	21,74468	22,01757
+	sp|Q9Y265|RUVB1_HUMAN;tr|E7ETR0|E7ETR0_HUMAN;tr|H7C4G5|H7C4G5_HUMAN;tr|H7C4I	sp|Q9Y265|RUVB1_HUMAN;tr|E7ETR0|E7ETR0_HUMAN	62,004	439680000	55	15	2,85E-05	0,922080517	24,01606	24,23213	24,31417	24,22317	25,09563	24,98412	25,18332	25,21078
+	sp|Q9Y6E7|SIR4_HUMAN	sp|Q9Y6E7|SIR4_HUMAN	323,31	1,2696E+10	112	18	2,90E-05	8,292857647	20,05073	23,19656	23,05988	21,9931	30,66306	30,11446	30,32184	30,37233
+	tr|E9PJM3|E9PJM3_HUMAN;sp|Q9UK99|FBX3_HUMAN;tr|G3V1E0|G3V1E0_HUMAN;tr|Q49AF:	tr|E9PJM3|E9PJM3_HUMAN;sp|Q9UK99|FBX3_HUMAN;tr|G3V1E0|G3V1E0_HUMAN	78,21	41665000	13	5	4,60E-05	2,478736401	19,85462	19,59173	19,50787	18,98565	21,52807	22,24403	22,0099	22,07281
+	sp|P52701|MSH6_HUMAN;tr|A0A087WWJ1|A0A087WWJ1_HUMAN;tr|A0A087WYT6|A0A087V	sp|P52701|MSH6_HUMAN;tr|A0A087WWJ1|A0A087WWJ1_HUMAN	50,005	128610000	35	15	4,61E-05	1,717473984	21,71494	22,21247	21,61733	21,60014	23,3648	23,39545	23,7145	23,54002
+	sp|P51570|GALK1_HUMAN;tr|K7ERJ9|K7ERJ9_HUMAN;tr|K7EII7|K7EII7_HUMAN;tr|K7ERN9|K7	sp|P51570|GALK1_HUMAN	38,354	55083000	15	9	5,42E-05	2,562343121	20,35628	19,73235	19,4705	20,18749	22,8766	22,56895	22,16533	22,38511
+	sp|P08670|VIME_HUMAN;tr|B0YJC4|B0YJC4_HUMAN;tr|B0YJC5|B0YJC5_HUMAN;tr|Q5JVS8|Q!	sp|P08670|VIME_HUMAN;tr|B0YJC4|B0YJC4_HUMAN;tr|B0YJC5|B0YJC5_HUMAN	323,31	5736700000	174	35	5,64E-05	1,19369173	27,56413	27,61947	27,7965	27,64897	28,80514	28,81922	29,14834	28,63114
+	sp|Q15149|PLEC_HUMAN;tr|E9PMV1|E9PMV1_HUMAN;tr|H0YDN1|H0YDN1_HUMAN;tr|E9PIA	sp|Q15149|PLEC_HUMAN	323,31	1014800000	193	89	5,73E-05	1,391541958	24,98242	25,20848	25,03649	24,74031	26,23035	26,36139	26,67101	26,27112
+	sp|P07355|ANXA2_HUMAN;tr|H0YN42|H0YN42_HUMAN;tr|H0YMD0|H0YMD0_HUMAN;tr|H0Y	sp|P07355|ANXA2_HUMAN;tr|H0YN42|H0YN42_HUMAN;tr|H0YMD0|H0YMD0_HUMAN;tr|H0Y	308,07	414470000	51	16	5,93E-05	-1,506282806	25,18117	25,52543	25,61591	25,57903	23,94726	24,17988	23,65992	24,08934
+	sp|P11142|HSP7C_HUMAN;tr|E9PKE3|E9PKE3_HUMAN;tr|E9PN89|E9PN89_HUMAN;tr|E9PNE6	sp|P11142|HSP7C_HUMAN;tr|E9PKE3|E9PKE3_HUMAN;tr|E9PN89|E9PN89_HUMAN;tr|E9PNE6	323,31	6069600000	158	27	6,11E-05	0,950216293	27,77649	27,89058	28,01919	27,94101	29,07217	28,86183	28,81357	28,68057
+	sp|P17066|HSP76_HUMAN;sp|P48741|HSP77_HUMAN	sp|P17066|HSP76_HUMAN;sp|P48741|HSP77_HUMAN	323,31	1759500000	7	2	6,49E-05	1,657655239	25,44695	25,79897	25,66117	25,84372	26,95571	27,32543	27,62631	27,47398
+	sp|P00367|DHE3_HUMAN;sp|P49448|DHE4_HUMAN	sp|P00367|DHE3_HUMAN;sp|P49448|DHE4_HUMAN	34,797	175770000	23	10	7,34E-05	0,966827869	22,87147	22,57646	22,92544	22,93744	23,90371	23,84986	23,65403	23,77052
+	sp|P30101|PDIA3_HUMAN	sp|P30101|PDIA3_HUMAN	147,39	792260000	74	16	7,73E-05	-1,526230812	26,05906	26,5747	26,50361	26,60484	24,80907	25,05205	24,68357	25,09261
+	sp|P17812|PYRG1_HUMAN;sp|Q9NRF8|PYRG2_HUMAN	sp|P17812|PYRG1_HUMAN	206,48	300720000	47	14	8,09E-05	1,167341709	23,25624	23,37862	23,38943	23,56813	24,83306	24,50743	24,59747	24,32382
+	tr|E7ESP9|E7ESP9_HUMAN;sp|P07197|NFM_HUMAN;tr|E7EMV2|E7EMV2_HUMAN;tr|A0A087'	tr|E7ESP9|E7ESP9_HUMAN;sp|P07197|NFM_HUMAN;tr|E7EMV2|E7EMV2_HUMAN	323,31	1,1138E+10	254	49	8,33E-05	1,693645	28,37501	28,35502	28,29408	27,9042	29,8416	29,6376	30,31786	29,90584
+	sp|P68371|TBB4B_HUMAN;tr|A0A075B736|A0A075B736_HUMAN;tr|Q5SQY0|Q5SQY0_HUMAI	sp|P68371|TBB4B_HUMAN	79,011	1177700000	16	2	8,84E-05	2,596793175	23,84355	24,19182	24,06357	24,39663	26,8411	27,09245	26,97662	25,97257
+	sp|Q9NU22|MDN1_HUMAN;tr|Q5T795|Q5T795_HUMAN;tr|M0QXR3|M0QXR3_HUMAN	sp|Q9NU22|MDN1_HUMAN	271,28	322600000	73	55	9,33E-05	3,377799511	21,512	21,99314	21,51663	20,79297	24,42355	25,03355	25,50724	24,36159
+	sp|Q9NRH3|TBG2_HUMAN;sp|P23258|TBG1_HUMAN;tr|K7EKE5|K7EKE5_HUMAN;tr|K7EIS0|K	sp|Q9NRH3|TBG2_HUMAN;sp|P23258|TBG1_HUMAN;tr|K7EKE5|K7EKE5_HUMAN;tr|K7EIS0|K	41,672	49071000	8	4	0,00010381	0,787096024	21,25569	NaN	21,06336	21,0921	21,86306	21,93524	22,04006	21,85821
+	sp|Q8NC51|PAIRB_HUMAN	sp|Q8NC51|PAIRB_HUMAN	96,365	380830000	39	12	0,00010394	-0,785710335	24,8944	24,90679	25,17866	25,19682	24,24547	24,3332	24,24612	24,20903
+	sp|P61962|DCAF7_HUMAN;tr|A0A087WWI6|A0A087WWI6_HUMAN	sp|P61962|DCAF7_HUMAN;tr|A0A087WWI6|A0A087WWI6_HUMAN	5,0395	20265000	7	4	0,00010861	0,942657948	19,41332	19,65081	19,70078	19,78328	20,51133	20,74476	20,63343	20,42931
+	sp|P23396|RS3_HUMAN;tr|E9PL09|E9PL09_HUMAN;tr|E9PPU1|E9PPU1_HUMAN;tr|F2Z2S8|F2	sp|P23396|RS3_HUMAN;tr|E9PL09|E9PL09_HUMAN;tr|E9PPU1|E9PPU1_HUMAN;tr|F2Z2S8|F2	323,31	1651000000	71	18	0,00013305	1,041637421	25,86665	26,05192	25,98986	26,00996	27,3132	27,04635	26,96157	26,76383
+	tr|A0A087X1B2|A0A087X1B2_HUMAN;tr|B9A018|B9A018_HUMAN;sp|Q53GS9|SNUT2_HUMAI	tr|A0A087X1B2|A0A087X1B2_HUMAN;tr|B9A018|B9A018_HUMAN;sp|Q53GS9|SNUT2_HUMAI	7,7371	6683600	9	2	0,00014675	-1,371304989	19,2978	19,35907	19,45069	19,40815	17,69927	18,40711	17,81753	18,10656
+	sp|P56192|SYMC_HUMAN;tr|F5H2V6|F5H2V6_HUMAN;tr|F8VS26|F8VS26_HUMAN;tr|F8W0M	sp|P56192|SYMC_HUMAN	139,09	292260000	42	15	0,00015626	1,039328098	23,58818	23,52504	23,54144	23,36038	24,75343	24,22177	24,57606	24,62109
+	sp|P14625|ENPL_HUMAN;tr|Q96GW1|Q96GW1_HUMAN;tr|H0YIV0|H0YIV0_HUMAN;tr|F8W0z	sp|P14625|ENPL_HUMAN	205,41	772570000	70	20	0,00018703	-0,790207863	26,07926	25,98895	26,10095	26,13401	25,25613	25,23184	25,10949	25,54489
+	tr|A0A087WXM6|A0A087WXM6_HUMAN;tr|J3QQT2|J3QQT2_HUMAN;tr|J3KRX5|J3KRX5_HUM	tr|A0A087WXM6|A0A087WXM6_HUMAN;tr|J3QQT2|J3QQT2_HUMAN;tr|J3KRX5|J3KRX5_HUM	19,026	213360000	26	4	0,00022319	-0,826394558	24,29824	24,44481	24,32079	24,09852	23,5064	23,64812	23,42034	23,28193
+	sp|O75822|EIF3J_HUMAN;tr|H0YGJ7|H0YGJ7_HUMAN;tr|H0YLP3|H0YLP3_HUMAN	sp|O75822|EIF3J_HUMAN;tr|H0YGJ7|H0YGJ7_HUMAN;tr|H0YLP3|H0YLP3_HUMAN	4,8066	11310000	3	3	0,00022948	-0,562249978	19,84189	19,94492	20,06316	19,95177	19,38062	19,33702	19,44692	NaN
+	sp|Q9Y285|SYFA_HUMAN;tr|K7ER00|K7ER00_HUMAN;tr|K7ER16|K7ER16_HUMAN;tr|K7EPH2|	sp|Q9Y285|SYFA_HUMAN;tr|K7ER00|K7ER00_HUMAN;tr|K7ER16|K7ER16_HUMAN;tr|K7EPH2|	53,979	157870000	17	6	0,00023005	1,818000317	21,85011	21,88703	22,4789	21,85445	24,27441	23,81617	23,42291	23,829
+	sp|Q9BUF5|TBB6_HUMAN;tr|K7ESM5|K7ESM5_HUMAN;tr|K7ES63|K7ES63_HUMAN;tr|K7EL29	sp|Q9BUF5|TBB6_HUMAN;tr|K7ESM5|K7ESM5_HUMAN	35,551	222220000	11	3	0,00023459	2,785657883	21,65692	21,45061	21,10636	21,52392	23,4768	24,77274	24,81054	23,82036
+	sp|P27824|CALX_HUMAN;tr|H0Y9Q7|H0Y9Q7_HUMAN;tr|D6RGY2|D6RGY2_HUMAN;tr|D6RFL1	sp|P27824|CALX_HUMAN;tr|H0Y9Q7|H0Y9Q7_HUMAN;tr|D6RGY2|D6RGY2_HUMAN	86,802	229350000	13	8	0,0002483	-0,838822842	24,11585	24,37981	24,41493	24,09031	23,26999	23,34204	23,57681	23,45676
+	tr|E5KLK1|E5KLK1_HUMAN;tr|E5KLJ9|E5KLJ9_HUMAN;tr|E5KLJ5|E5KLJ5_HUMAN;tr|E5KLJ6|E5I	tr|E5KLK1|E5KLK1_HUMAN;tr|E5KLJ9|E5KLJ9_HUMAN;tr|E5KLJ5|E5KLJ5_HUMAN;tr|E5KLJ6|E5	37,168	116830000	21	1	0,00024838	1,813467503	21,50368	21,53825	21,15919	21,704	23,49094	23,42355	23,56454	22,67997
+	sp|P78417|GSTO1_HUMAN;tr|Q5TA02|Q5TA02_HUMAN;tr|Q5TA01|Q5TA01_HUMAN	sp|P78417|GSTO1_HUMAN;tr|Q5TA02|Q5TA02_HUMAN;tr|Q5TA01|Q5TA01_HUMAN	12,189	83141000	17	4	0,00027883	-1,300671577	22,87085	23,16566	23,02442	23,20784	21,79749	22,15224	21,40145	21,7149
+	sp|P36542|ATPG_HUMAN	sp|P36542|ATPG_HUMAN	28,01	127180000	23	6	0,00028262	1,090640068	22,25324	22,24566	22,19346	22,23637	23,5657	23,42944	23,39061	22,90555
+	sp|P14314|GLU2B_HUMAN;tr|K7ELL7|K7ELL7_HUMAN;tr|K7EKX1|K7EKX1_HUMAN;tr|K7EJ70|I	sp|P14314|GLU2B_HUMAN;tr|K7ELL7|K7ELL7_HUMAN	14,311	101410000	20	7	0,0003205	-0,94900465	23,05886	23,20647	23,4443	23,16079	22,42637	22,38835	22,27378	21,9859
+	sp|P05023|AT1A1_HUMAN;tr|M0R116|M0R116_HUMAN;sp|P13637|AT1A3_HUMAN;tr|A0A0A	sp|P05023|AT1A1_HUMAN	13,813	39108000	12	7	0,00033114	-0,977594852	21,91987	21,78592	21,97337	21,81464	21,03322	20,54312	20,89378	21,11331
+	sp|Q15477|SKIV2_HUMAN;tr|H7C5N0|H7C5N0_HUMAN;tr|H7C4L3|H7C4L3_HUMAN;tr|F8WDE	sp|Q15477|SKIV2_HUMAN	26,43	23625000	17	8	0,00033242	2,378138383	18,75887	18,75392	NaN	18,74613	21,11832	21,50465	21,41761	20,48385
+	sp|P07900|HS90A_HUMAN;tr|G3V2J8|G3V2J8_HUMAN;sp|Q14568|HS902_HUMAN;sp|Q58FF6	sp|P07900|HS90A_HUMAN	323,31	1630900000	89	16	0,00033571	-0,556956768	27,04781	27,06476	27,08487	27,03118	26,47653	26,53224	26,3136	26,67842
+	sp|Q9NYU2|UGGG1_HUMAN;tr|H7BZG0|H7BZG0_HUMAN	sp|Q9NYU2|UGGG1_HUMAN	17,749	37467000	13	7	0,00034602	1,223903656	20,48611	20,63476	20,14146	20,3229	21,30279	21,51114	21,80388	21,86302
+	sp|P26583|HMGB2_HUMAN;tr|D6R9A6|D6R9A6_HUMAN	sp|P26583|HMGB2_HUMAN;tr|D6R9A6|D6R9A6_HUMAN	10,895	112640000	6	2	0,00036772	-1,000659307	23,44405	23,49644	NaN	23,47729	NaN	22,33615	22,44177	22,63788
+	sp|P08758|ANXA5_HUMAN;tr|D6RBE9|D6RBE9_HUMAN;tr|D6RBL5|D6RBL5_HUMAN;tr|E9PHT	sp|P08758|ANXA5_HUMAN;tr|D6RBE9|D6RBE9_HUMAN;tr|D6RBL5|D6RBL5_HUMAN;tr|E9PHT	44,126	204970000	22	8	0,00038828	-0,755225182	24,03565	24,2786	24,24372	24,20292	23,35582	23,54851	23,21681	23,61885
+	sp|Q9Y2S7|PDIP2_HUMAN;tr|B4DEM9|B4DEM9_HUMAN	sp|Q9Y2S7|PDIP2_HUMAN;tr|B4DEM9|B4DEM9_HUMAN	10,183	62187000	11	6	0,00041839	2,16742897	19,91003	20,32553	20,22835	20,77394	22,87701	22,76525	21,7565	22,50882
+	tr|J3QS39|J3QS39_HUMAN;tr|J3QTR3|J3QTR3_HUMAN;tr|F5H6Q2|F5H6Q2_HUMAN;sp|P6298	tr|J3QS39|J3QS39_HUMAN;tr|J3QTR3|J3QTR3_HUMAN;tr|F5H6Q2|F5H6Q2_HUMAN;sp|P6298	323,31	1500000000	39	6	0,00042066	0,66782093	26,25308	26,18592	26,13022	26,18215	26,76231	26,98577	26,64361	27,03097
+	sp|P32969|RL9_HUMAN;tr|D6RAN4|D6RAN4_HUMAN;tr|H0Y9V9|H0Y9V9_HUMAN;tr|H0Y9R4|	sp|P32969|RL9_HUMAN;tr|D6RAN4|D6RAN4_HUMAN;tr|H0Y9V9|H0Y9V9_HUMAN;tr|H0Y9R4	122,8	76848000	16	6	0,00044692	-1,155788898	22,98456	23,047	22,8975	22,37241	21,66166	21,49799	21,71892	21,79974
+	sp|P35579|MYH9_HUMAN;tr|A0A0C4DFM8|A0A0C4DFM8_HUMAN;tr|E7ERA5|E7ERA5_HUMAI	sp|P35579|MYH9_HUMAN	86,019	191800000	52	19	0,00045389	-0,792589664	24,07863	24,07243	24,05457	23,97727	23,00136	23,19104	23,54191	23,27824
+	sp|Q13200|PSMD2_HUMAN;tr|H7C1H2|H7C1H2_HUMAN;tr|H7C2Q3|H7C2Q3_HUMAN;tr|C9JI	sp|Q13200|PSMD2_HUMAN	272,49	182810000	45	14	0,00049349	0,551928997	23,21846	23,05013	23,39401	23,10396	23,67799	23,74725	23,73376	23,81529
+	tr|A0A087X1S2|A0A087X1S2_HUMAN;sp|P67809|YBOX1_HUMAN;tr|H0Y449|H0Y449_HUMAN	tr|A0A087X1S2|A0A087X1S2_HUMAN;sp|P67809|YBOX1_HUMAN;tr|H0Y449|H0Y449_HUMAN	270,93	210540000	39	5	0,00054162	-0,590010166	24,03607	23,93148	24,2627	23,86872	23,38627	23,46837	23,44139	23,44291
+	tr|B8ZZK4|B8ZZK4_HUMAN;tr|H7C2W9|H7C2W9_HUMAN;tr|C9JU56|C9JU56_HUMAN;tr|B7Z4I	tr|B8ZZK4|B8ZZK4_HUMAN;tr|H7C2W9|H7C2W9_HUMAN;tr|C9JU56|C9JU56_HUMAN;tr|B7Z4	10,182	378870000	13	4	0,00055599	-1,053188801	25,01572	25,478	25,18044	24,89592	24,31264	24,04335	24,14538	23,85595
+	sp|Q92973|TNPO1_HUMAN;tr|E7EW37|E7EW37_HUMAN;tr|S4R398|S4R398_HUMAN	sp|Q92973|TNPO1_HUMAN	24,541	68774000	17	6	0,00055834	-0,408211708	22,35982	22,39788	22,32833	22,41784	21,80116	22,06161	22,02901	21,97925
+	tr|E9PKZ0|E9PKZ0_HUMAN;sp|P62917|RL8_HUMAN;tr|G3V1A1|G3V1A1_HUMAN;tr|E9PKU4|E	tr|E9PKZ0|E9PKZ0_HUMAN;sp|P62917|RL8_HUMAN;tr|G3V1A1|G3V1A1_HUMAN;tr|E9PKU4|E	52,151	550350000	23	5	0,00057795	-0,98334837	25,92334	25,77957	25,67069	25,41976	24,49375	24,65638	24,99386	24,71597
+	sp|Q7Z6Z7|HUWE1_HUMAN;tr|H0Y659|H0Y659_HUMAN;tr|A0A087X1S3|A0A087X1S3_HUMAI	sp|Q7Z6Z7|HUWE1_HUMAN	11,083	20462000	8	7	0,00059228	0,781539917	20,02296	19,91704	19,65529	19,74787	20,37039	20,64271	20,70269	20,75352
+	sp|P46060|RAGP1_HUMAN;tr|B0QYT5|B0QYT5_HUMAN;tr|B0QYT4|B0QYT4_HUMAN;tr|H0Y4(	sp|P46060|RAGP1_HUMAN	58,542	127960000	34	12	0,00059938	-0,570707321	23,23919	23,46114	23,27469	23,31576	22,96201	22,64309	22,67734	22,72551
+	sp|Q96EY1|DNJA3_HUMAN;tr|I3L1T6|I3L1T6_HUMAN	sp|Q96EY1|DNJA3_HUMAN	42,708	210120000	21	9	0,00060506	2,926895142	20,92716	20,68252	21,73198	21,34392	24,84566	24,40886	24,0878	23,05084
+	sp|Q9UBN7|HDAC6_HUMAN;tr|A6NDI8|A6NDI8_HUMAN;tr|E7EPS2|E7EPS2_HUMAN;tr|C9JEF4	sp|Q9UBN7|HDAC6_HUMAN	151,94	253370000	41	9	0,00060567	1,741656303	22,47593	23,09905	22,80473	22,54409	23,93787	24,27597	24,75062	24,92597
+	sp|P04181|OAT_HUMAN	sp|P04181|OAT_HUMAN	110,17	734870000	54	14	0,00061078	2,119787693	22,99039	23,9478	24,20508	24,45098	26,13673	26,05397	26,00123	25,88148
+	sp|Q16531|DDB1_HUMAN;tr|F5GY55|F5GY55_HUMAN;tr|F5H2L3|F5H2L3_HUMAN;tr|F5GWI0	sp|Q16531|DDB1_HUMAN;tr|F5GY55|F5GY55_HUMAN	37,577	226570000	39	14	0,00061404	0,64963007	23,56999	23,40133	23,55415	23,52767	24,13469	23,91534	24,2798	24,32182
+	sp|Q9NSE4|SYIM_HUMAN	sp|Q9NSE4|SYIM_HUMAN	96,834	188470000	30	14	0,00067096	2,066092968	21,73674	22,10582	21,4108	22,7066	23,79064	23,84019	24,49802	24,09547
+	sp|Q9Y490|TLN1_HUMAN;sp|Q9Y4G6|TLN2_HUMAN	sp|Q9Y490|TLN1_HUMAN	9,635	46212000	10	8	0,00072246	-0,43302536	21,74082	21,75772	21,70485	21,58568	21,18392	21,22653	21,21573	21,43079
+	sp|P35232|PHB_HUMAN;tr|C9JW96|C9JW96_HUMAN;tr|C9JZ20|C9JZ20_HUMAN;tr|E7ESE2|E7	sp|P35232|PHB_HUMAN;tr|C9JW96|C9JW96_HUMAN;tr|C9JZ20|C9JZ20_HUMAN;tr|E7ESE2|E7	33,803	113170000	25	10	0,00072661	1,528509617	21,5888	21,37279	21,32729	22,16023	23,07405	23,56546	22,8866	23,03703
+	sp|P17987|TCPA_HUMAN;tr|E7ERF2|E7ERF2_HUMAN;tr|E7EQR6|E7EQR6_HUMAN;tr|F5H282|	sp|P17987|TCPA_HUMAN;tr|E7ERF2|E7ERF2_HUMAN;tr|E7EQR6|E7EQR6_HUMAN;tr|F5H282|	176,47	964890000	56	20	0,00074599	0,393635273	25,60147	25,5846	25,80519	25,74707	26,15509	26,05316	26,00973	26,09489

+	tr|I3L2C7|I3L2C7_HUMAN;sp|P57678|GEMI4_HUMAN	tr|I3L2C7|I3L2C7_HUMAN;sp|P57678|GEMI4_HUMAN	19,787	37735000	13	7	0,00075825	1,887225628	19,99162	19,67635	19,65017	19,58893	20,79352	21,67616	22,0971	21,88919
+	sp|P13995|MTDC_HUMAN;tr|B9A062|B9A062_HUMAN;tr|B8ZZU9|B8ZZU9_HUMAN	sp|P13995|MTDC_HUMAN;tr|B9A062|B9A062_HUMAN	11,923	93732000	10	4	0,00076056	0,593276978	22,26284	22,45194	22,0733	22,20425	22,87049	22,97722	22,75371	22,76401
+	sp|P12277|KCRB_HUMAN;tr|H0YJG0|H0YJG0_HUMAN;tr|G3V4N7|G3V4N7_HUMAN;tr|G3V461	sp|P12277|KCRB_HUMAN	175,99	704460000	44	12	0,00078612	-0,981456757	25,77228	25,89913	26,25641	26,18741	25,04573	25,06899	24,77575	25,29894
+	sp|Q8NF37|PCAT1_HUMAN;tr|A0A0G2JQ62|A0A0G2JQ62_HUMAN;tr|A0A0G2JRI7|A0A0G2JRI7	sp|Q8NF37|PCAT1_HUMAN;tr|A0A0G2JQ62|A0A0G2JQ62_HUMAN	13,498	15984000	4	3	0,00080639	0,544814587	19,70818	19,6263	19,71139	19,72384	20,08317	20,45854	20,1278	20,27946
+	tr|Q5JR95|Q5JR95_HUMAN;sp|P62241|RS8_HUMAN	tr|Q5JR95|Q5JR95_HUMAN;sp|P62241|RS8_HUMAN	323,31	308230000	27	7	0,00082726	-0,61090374	24,87895	24,76875	24,61661	24,46781	24,05084	24,05192	23,99499	24,19077
+	sp|P24752|THIL_HUMAN;tr|H0YEL7|H0YEL7_HUMAN;tr|E9PRQ6|E9PRQ6_HUMAN	sp|P24752|THIL_HUMAN	26,289	133110000	17	8	0,00083332	-1,046599865	23,54097	23,61392	23,60513	23,80548	22,60716	22,30129	22,43489	23,03576
+	sp|P62829|RL23_HUMAN;tr|J3KT29|J3KT29_HUMAN;tr|C9JD32|C9JD32_HUMAN;tr|B9ZVP7|B9	sp|P62829|RL23_HUMAN;tr|J3KT29|J3KT29_HUMAN;tr|C9JD32|C9JD32_HUMAN;tr|B9ZVP7|B9	120,48	1252200000	26	9	0,00086671	1,369823456	25,02323	25,59619	25,3839	24,94566	26,77731	26,86597	26,6487	26,1363
+	sp|P53618|COPB_HUMAN;tr|E9PP73|E9PP73_HUMAN	sp|P53618|COPB_HUMAN	15,061	93797000	15	7	0,00086886	0,823623657	22,28351	21,86982	22,2321	22,10965	22,77888	22,82083	23,21202	22,97783
+	sp|Q13838|DX39B_HUMAN;tr|A0A0G2JJZ9|A0A0G2JJZ9_HUMAN;tr|Q5STU3|Q5STU3_HUMAN;	sp|Q13838|DX39B_HUMAN;tr|A0A0G2JJZ9|A0A0G2JJZ9_HUMAN;tr|Q5STU3|Q5STU3_HUMAN;	88,343	523240000	46	6	0,00087173	-0,734306335	25,31057	25,46005	25,48557	25,63675	24,66503	24,88311	24,49082	24,91675
+	sp|P18206|VINC_HUMAN;tr|Q5JQ13|Q5JQ13_HUMAN;tr|A0A096LPE1|A0A096LPE1_HUMAN	sp|P18206|VINC_HUMAN;tr|Q5JQ13|Q5JQ13_HUMAN	20,001	55526000	27	9	0,00090733	-0,942325592	22,40852	22,58456	22,21824	22,35861	21,41338	21,38337	21,1784	21,82547
+	sp|P47914|RL29_HUMAN	sp|P47914|RL29_HUMAN	13,433	179600000	13	2	0,00096315	-0,734152794	24,0694	24,18155	23,95748	23,72203	23,44316	23,08918	23,23326	23,22825
+	sp|P05198|IF2A_HUMAN;tr|H0YJS4|H0YJS4_HUMAN;tr|G3V4T5|G3V4T5_HUMAN	sp|P05198|IF2A_HUMAN;tr|H0YJS4|H0YJS4_HUMAN;tr|G3V4T5|G3V4T5_HUMAN	18,648	59429000	20	7	0,001048	-0,799869537	22,08748	22,46501	22,47551	22,2554	21,78093	21,38685	21,35035	21,56579
+	sp|Q9UQ80|PA2G4_HUMAN;tr|F8VR77|F8VR77_HUMAN;tr|H0YIN7|H0YIN7_HUMAN;tr|F8W0A	sp|Q9UQ80|PA2G4_HUMAN;tr|F8VR77|F8VR77_HUMAN	138,58	508840000	48	14	0,00106077	-0,773202419	25,36326	25,37352	25,69067	25,58035	24,85234	24,84364	24,41944	24,79956
+	sp|P14550|AK1A1_HUMAN;tr|V9GYG2|V9GYG2_HUMAN;tr|V9GYP9|V9GYP9_HUMAN;tr|Q5T6	sp|P14550|AK1A1_HUMAN	4,4608	46852000	10	4	0,0010795	-1,200034618	22,01808	22,17316	22,15576	22,37289	21,08622	21,1249	20,42234	21,2863
+	tr|K7EPP7|K7EPP7_HUMAN;tr|K7ESP1|K7ESP1_HUMAN;sp|Q99615|DNJC7_HUMAN;tr|K7EIH8|	tr|K7EPP7|K7EPP7_HUMAN;tr|K7ESP1|K7ESP1_HUMAN;sp|Q99615|DNJC7_HUMAN;tr|K7EIH8	16,907	33388000	7	5	0,00108629	0,612635612	20,85536	20,90353	20,83098	NaN	21,65932	21,5354	21,32893	21,38005
+	sp|P09429|HMGB1_HUMAN;tr|Q5T7C4|Q5T7C4_HUMAN;sp|B2RPK0|HGB1A_HUMAN;sp|P234	sp|P09429|HMGB1_HUMAN;tr|Q5T7C4|Q5T7C4_HUMAN;sp|B2RPK0|HGB1A_HUMAN	57,811	410720000	25	6	0,00112742	-1,25265646	25,09317	25,5402	25,45403	25,43654	24,61863	23,96737	23,72067	24,20665
+	sp|P00918|CAH2_HUMAN;tr|E5RID5|E5RID5_HUMAN;tr|E5RK37|E5RK37_HUMAN	sp|P00918|CAH2_HUMAN	141,03	670490000	46	9	0,00113062	-1,216906071	25,86901	26,02035	26,04839	26,3124	24,88428	24,97136	24,32079	25,20609
+	sp|P45954|ACDSB_HUMAN	sp|P45954|ACDSB_HUMAN	9,5977	45390000	9	6	0,00113667	1,296061039	20,62471	20,51912	20,24016	20,56672	21,79634	22,32127	21,69379	21,32356
+	sp|Q9P258|RCC2_HUMAN	sp|Q9P258|RCC2_HUMAN	29,334	84786000	22	6	0,00115435	-0,630110264	22,47108	22,85369	22,87341	22,87093	22,18123	22,2127	22,00751	22,14722
+	sp|P62826|RAN_HUMAN;tr|B5MDF5|B5MDF5_HUMAN;tr|J3KQE5|J3KQE5_HUMAN;tr|F5H018	sp|P62826|RAN_HUMAN;tr|B5MDF5|B5MDF5_HUMAN;tr|J3KQE5|J3KQE5_HUMAN;tr|F5H018	151,53	701400000	25	8	0,00119533	-0,818963051	25,78756	25,91216	26,06003	26,0625	25,09547	25,49485	25,04856	24,90752
+	tr|A0A0A6YYJ8|A0A0A6YYJ8_HUMAN;tr|A0A0A6YYC3|A0A0A6YYC3_HUMAN;sp|Q9Y383|LC7L2	tr|A0A0A6YYJ8|A0A0A6YYJ8_HUMAN;tr|A0A0A6YYC3|A0A0A6YYC3_HUMAN;sp|Q9Y383|LC7L2	12,778	54416000	15	3	0,00119659	-0,832974911	21,9132	22,17937	22,38287	22,38696	21,28918	21,30196	21,28167	21,6577
+	sp|Q9UBS4|DJB11_HUMAN;tr|H7C2Y5|H7C2Y5_HUMAN	sp|Q9UBS4|DJB11_HUMAN	77,51	75304000	18	8	0,00121307	1,912279129	20,51316	20,63556	20,67736	20,74608	22,59562	22,98722	23,02722	21,61122
+	sp|P10398|ARAF_HUMAN;tr|Q96II5|Q96II5_HUMAN;tr|H7C4S5|H7C4S5_HUMAN	sp|P10398|ARAF_HUMAN;tr|Q96II5|Q96II5_HUMAN	52,797	284800000	31	9	0,00121403	3,031464577	20,84736	22,64229	21,6897	21,03617	23,80892	25,46136	24,54475	24,52636
+	tr|A0A024QZP7|A0A024QZP7_HUMAN;sp|P06493|CDK1_HUMAN;tr|E5RIU6|E5RIU6_HUMAN;t	tr|A0A024QZP7|A0A024QZP7_HUMAN;sp|P06493|CDK1_HUMAN;tr|E5RIU6|E5RIU6_HUMAN;t	53,959	348720000	31	10	0,0012461	1,116753101	23,39048	24,06587	23,68067	23,7168	25,2288	24,76263	24,59121	24,7382
+	sp|P31040|SDHA_HUMAN;tr|A0A087X1I3|A0A087X1I3_HUMAN;tr|D6RFM5|D6RFM5_HUMAN;	sp|P31040|SDHA_HUMAN;tr|A0A087X1I3|A0A087X1I3_HUMAN;tr|D6RFM5|D6RFM5_HUMAN	17,419	81060000	10	6	0,00130795	1,760872841	20,64808	21,34131	21,13458	20,82602	22,58217	23,1888	22,05696	23,16555
+	sp|P84077|ARF1_HUMAN;sp|P61204|ARF3_HUMAN;tr|F5H423|F5H423_HUMAN;tr|F5H0C7|F5	sp|P84077|ARF1_HUMAN;sp|P61204|ARF3_HUMAN;tr|F5H423|F5H423_HUMAN	105,77	253460000	27	5	0,00131495	-1,008330822	24,69285	24,38667	24,56349	24,27199	23,64262	23,7679	23,07897	23,39218
+	sp|O15269|SPTC1_HUMAN	sp|O15269|SPTC1_HUMAN	78,421	86465000	16	10	0,00132178	1,771913528	21,20174	20,76806	21,12666	21,15146	23,6075	22,80043	22,77173	22,15592
+	sp|P09960|LKHA4_HUMAN;tr|B4DEH5|B4DEH5_HUMAN	sp|P09960|LKHA4_HUMAN	78,287	56529000	22	8	0,0013224	-0,71632719	22,04363	22,25468	22,29286	22,26937	21,28991	21,80613	21,521	21,37819
+	sp|Q71U36|TBA1A_HUMAN;sp|Q13748|TBA3C_HUMAN;sp|Q6PEY2|TBA3E_HUMAN;tr|F8VQQ	sp|Q71U36|TBA1A_HUMAN;sp|Q13748|TBA3C_HUMAN;sp|Q6PEY2|TBA3E_HUMAN	17,633	187250000	12	1	0,0013516	3,567385197	20,45192	20,00826	20,46802	20,17719	22,79753	24,34969	22,85217	25,37554
+	sp|P23921|RIR1_HUMAN;tr|E9PL69|E9PL69_HUMAN;tr|E9PP77|E9PP77_HUMAN;tr|H0YCY7|H	sp|P23921|RIR1_HUMAN;tr|E9PL69|E9PL69_HUMAN	23,924	80699000	27	11	0,0013625	-0,798774242	22,48866	23,05137	22,84621	22,77181	21,75727	22,09487	21,99814	22,11267
+	sp|Q14739|LBR_HUMAN;tr|C9JXK0|C9JXK0_HUMAN;tr|C9JES9|C9JES9_HUMAN	sp|Q14739|LBR_HUMAN;tr|C9JXK0|C9JXK0_HUMAN	131,08	67814000	18	4	0,00140903	0,792063713	21,29922	21,43731	21,76547	21,47571	22,26405	22,10572	22,57907	22,19713
+	sp|Q9Y4R8|TELO2_HUMAN;tr|H3BRS3|H3BRS3_HUMAN;tr|H3BU45|H3BU45_HUMAN	sp|Q9Y4R8|TELO2_HUMAN	71,605	66864000	21	13	0,00141988	1,948773861	20,70329	21,08005	19,82208	20,5418	22,48971	22,61865	22,96842	21,86552
+	sp|Q9NZL4|HPBP1_HUMAN;tr|K7EN20|K7EN20_HUMAN;tr|K7EL16|K7EL16_HUMAN;tr|K7EMN	sp|Q9NZL4|HPBP1_HUMAN;tr|K7EN20|K7EN20_HUMAN	13,696	32255000	11	5	0,00144032	0,580550671	20,83391	20,77523	20,56141	20,66622	21,47833	21,19856	21,10162	21,38047
+	sp|Q96AE4|FUBP1_HUMAN;tr|E9PEB5|E9PEB5_HUMAN;tr|C9JSZ1|C9JSZ1_HUMAN;sp|Q96I24|	sp|Q96AE4|FUBP1_HUMAN;tr|E9PEB5|E9PEB5_HUMAN	30,436	176010000	34	9	0,00148576	-0,585185528	23,69424	23,79005	23,77766	24,01546	23,16378	23,16016	23,13717	23,47556
+	sp|Q969Z0|TBRG4_HUMAN;tr|H7C4R5|H7C4R5_HUMAN;tr|C9IZN7|C9IZN7_HUMAN	sp|Q969Z0|TBRG4_HUMAN;tr|H7C4R5|H7C4R5_HUMAN	6,976	15149000	7	3	0,00148813	0,288615704	19,69569	19,61061	19,7147	19,78931	19,95035	20,08537	19,91615	20,01291
+	sp|Q96HE7|ERO1A_HUMAN;tr|G3V2H0|G3V2H0_HUMAN;tr|G3V5B3|G3V5B3_HUMAN;tr|Q5T	sp|Q96HE7|ERO1A_HUMAN	29,697	39694000	18	5	0,00149457	-0,791732788	21,69387	22,03224	21,90183	21,85152	21,10738	21,22594	20,71549	21,26373
+	sp|Q5JTZ9|SYAM_HUMAN	sp|Q5JTZ9|SYAM_HUMAN	37,946	50867000	24	8	0,00152133	1,008375168	20,94185	21,36906	20,78564	21,10495	22,24025	21,66583	22,10137	22,22756
+	sp|Q8N1F7|NUP93_HUMAN;tr|H3BVG0|H3BVG0_HUMAN;tr|H3BNN5|H3BNN5_HUMAN;tr|H3I	sp|Q8N1F7|NUP93_HUMAN;tr|H3BVG0|H3BVG0_HUMAN	14,611	36334000	16	8	0,00154619	-0,650156498	21,55075	21,61796	21,80148	21,64518	20,90551	21,08037	20,77	21,25885
+	sp|P50990|TCPQ_HUMAN;tr|H7C4C8|H7C4C8_HUMAN;tr|H7C2U0|H7C2U0_HUMAN	sp|P50990|TCPQ_HUMAN;tr|H7C4C8|H7C4C8_HUMAN	294,5	868830000	85	23	0,00175494	-0,494951725	25,92334	26,00759	26,17491	26,23392	25,50585	25,64878	25,48081	25,72453
+	tr|A0A087X054|A0A087X054_HUMAN;sp|Q9Y4L1|HYOU1_HUMAN;tr|E9PJ21|E9PJ21_HUMAN;	tr|A0A087X054|A0A087X054_HUMAN;sp|Q9Y4L1|HYOU1_HUMAN;tr|E9PJ21|E9PJ21_HUMAN;	11,5	34830000	11	6	0,00176657	-1,403389295	21,52057	22,01318	22,20786	22,07114	NaN	20,33604	20,40997	20,90338
+	tr|B7Z7P8|B7Z7P8_HUMAN;sp|P62495|ERF1_HUMAN;tr|I3L492|I3L492_HUMAN;tr|D6RCB3|D	tr|B7Z7P8|B7Z7P8_HUMAN;sp|P62495|ERF1_HUMAN	16,12	57649000	18	7	0,00177915	-0,60273838	22,08047	22,112	22,28481	22,32237	21,41183	21,86469	21,55544	21,55675
+	sp|Q15084|PDIA6_HUMAN	sp|Q15084|PDIA6_HUMAN	219,42	296170000	34	9	0,0017825	-0,844445229	24,62556	24,80336	24,80312	24,97998	23,78596	24,12629	23,66036	24,26163
+	sp|Q13724|MOGS_HUMAN;tr|C9J8D4|C9J8D4_HUMAN;tr|C9JDQ1|C9JDQ1_HUMAN	sp|Q13724|MOGS_HUMAN;tr|C9J8D4|C9J8D4_HUMAN	20,964	51140000	20	9	0,00178258	1,135101795	20,88442	20,88181	20,99514	20,78421	22,38393	22,11032	22,171	21,42075
+	tr|Q5HYB6|Q5HYB6_HUMAN;tr|A0A087WWU8|A0A087WWU8_HUMAN;tr|D6R904|D6R904_HU	tr|Q5HYB6|Q5HYB6_HUMAN;tr|A0A087WWU8|A0A087WWU8_HUMAN;tr|D6R904|D6R904_HU	102,23	328610000	44	7	0,00179774	-0,794735432	24,5335	24,8412	24,72817	24,76759	24,05631	24,23586	23,68014	23,71921
+	sp|P04075|ALDOA_HUMAN;tr|J3KPS3|J3KPS3_HUMAN;tr|H3BQN4|H3BQN4_HUMAN;tr|H3BPS	sp|P04075|ALDOA_HUMAN;tr|J3KPS3|J3KPS3_HUMAN;tr|H3BQN4|H3BQN4_HUMAN;tr|H3BPS	323,31	2723800000	92	16	0,00182535	-0,788691998	27,67963	27,73635	27,87203	27,99863	27,05051	27,25124	26,66166	27,16844
+	tr|E9PBK6|E9PBK6_HUMAN;tr|E9PDU5|E9PDU5_HUMAN;sp|Q9NNW5|WDR6_HUMAN;tr|A0A	tr|E9PBK6|E9PBK6_HUMAN;tr|E9PDU5|E9PDU5_HUMAN;sp|Q9NNW5|WDR6_HUMAN;tr|A0A	2,1418	9605000	4	2	0,00184011	0,846937021	18,96747	NaN	18,70215	18,6989	19,77043	19,84387	19,42232	19,50916
+	sp|O43670|ZN207_HUMAN;tr|J3QRS9|J3QRS9_HUMAN;tr|X6R4W8|X6R4W8_HUMAN;tr|J3KS3	sp|O43670|ZN207_HUMAN;tr|J3QRS9|J3QRS9_HUMAN;tr|X6R4W8|X6R4W8_HUMAN;tr|J3KS3	10,189	37035000	11	3	0,00186501	-0,68453598	21,56211	21,27725	21,86533	21,59578	20,98294	20,9556	20,85064	20,77314
+	sp|P42704|LPPRC_HUMAN;tr|B8ZZ38|B8ZZ38_HUMAN;tr|A0A0C4DG06|A0A0C4DG06_HUMAN	sp|P42704|LPPRC_HUMAN	200,57	435190000	71	28	0,00190324	-0,57439518	25,03381	25,18643	25,19888	25,25429	24,62199	24,47494	24,41886	24,86003
+	sp|P27797|CALR_HUMAN;tr|K7EJB9|K7EJB9_HUMAN	sp|P27797|CALR_HUMAN;tr|K7EJB9|K7EJB9_HUMAN	61,769	178700000	26	7	0,00194021	-0,609566212	23,85833	23,93814	24,06242	24,10597	23,24367	23,61122	23,18126	23,49045
+	tr|E7EPM6|E7EPM6_HUMAN;sp|P33121|ACSL1_HUMAN;tr|B7Z3Z9|B7Z3Z9_HUMAN;tr|H0Y9U	tr|E7EPM6|E7EPM6_HUMAN;sp|P33121|ACSL1_HUMAN;tr|B7Z3Z9|B7Z3Z9_HUMAN	17,224	122920000	17	11	0,00197248	2,852953593	20,53488	20,42039	20,66926	NaN	23,03063	23,64284	24,39146	22,51292
+	sp|P30040|ERP29_HUMAN;tr|F8VY02|F8VY02_HUMAN;tr|F8W1G0|F8W1G0_HUMAN	sp|P30040|ERP29_HUMAN;tr|F8VY02|F8VY02_HUMAN	7,0946	38650000	12	4	0,00203031	-1,100649357	21,43201	21,85871	21,97386	21,90228	20,89112	21,01842	20,23538	20,61935
+	sp|P09012|SNRPA_HUMAN;tr|M0R268|M0R268_HUMAN;tr|M0R0G9|M0R0G9_HUMAN;tr|M0	sp|P09012|SNRPA_HUMAN;tr|M0R268|M0R268_HUMAN;tr|M0R0G9|M0R0G9_HUMAN;tr|M0	6,5371	85966000	12	2	0,00214446	-0,570259571	22,77167	22,55494	22,95807	22,8938	22,12174	22,22726	22,13655	22,41189
+	sp|P26440|IVD_HUMAN;tr|A0A0A0MT83|A0A0A0MT83_HUMAN;tr|H0YKV0|H0YKV0_HUMAN;	sp|P26440|IVD_HUMAN;tr|A0A0A0MT83|A0A0A0MT83_HUMAN;tr|H0YKV0|H0YKV0_HUMAN;	4,6962	19020000	8	2	0,00216746	-1,154844761	20,78205	20,92904	20,88696	21,23433	20,15326	19,77703	19,47945	NaN
+	sp|Q6PI48|SYDM_HUMAN	sp|Q6PI48|SYDM_HUMAN	7,5667	24740000	8	5	0,00220421	1,044651508	19,7408	19,86497	19,51549	19,98663	20,60467	21,04946	21,19135	20,44101
+	sp|P52597|HNRPF_HUMAN	sp|P52597|HNRPF_HUMAN	187,36	115920000	19	4	0,00221584	0,537383556	22,58456	22,36284	22,63808	22,47013	23,06651	23,2029	23,12726	22,80847
+	sp|P62750|RL23A_HUMAN;tr|H7BY10|H7BY10_HUMAN;tr|K7EJV9|K7EJV9_HUMAN;tr|K7ERT8|	sp|P62750|RL23A_HUMAN;tr|H7BY10|H7BY10_HUMAN;tr|K7EJV9|K7EJV9_HUMAN;tr|K7ERT8|	206,98	419160000	34	8	0,00229548	-0,819807529	25,14048	25,3946	25,19242	24,90981	24,37504	24,68105	24,20628	24,09571
+	sp|P14868|SYDC_HUMAN;tr|C9J7S3|C9J7S3_HUMAN;tr|C9JLC1|C9JLC1_HUMAN;tr|C9JQM9|C9	sp|P14868|SYDC_HUMAN	31,406	139240000	30	11	0,00232226	-0,263328552	23,25136	23,30438	23,43275	23,23997	23,01698	23,12367	22,99926	23,03525
+	sp|P09661|RU2A_HUMAN;tr|H0YKK0|H0YKK0_HUMAN;tr|H0YMA0|H0YMA0_HUMAN;tr|H0YLF	sp|P09661|RU2A_HUMAN;tr|H0YKK0|H0YKK0_HUMAN;tr|H0YMA0|H0YMA0_HUMAN	59,101	61274000	15	6	0,00237895	-0,301971912	22,30897	22,10789	22,2232	22,35531	21,97914	21,98992	21,9411	21,87733
+	sp|P35998|PRS7_HUMAN;tr|C9JLS9|C9JLS9_HUMAN	sp|P35998|PRS7_HUMAN	72,267	194570000	33	15	0,00244237	0,515362263	23,04845	23,14436	23,37703	23,39008	23,7214	23,92687	23,6796	23,69349
+	sp|P61981|1433G_HUMAN	sp|P61981|1433G_HUMAN	88,21	65321000	14	3	0,00253232	-0,410300255	22,21386	22,29911	22,36327	22,34335	21,93844	21,87721	21,6996	22,06313
+	sp|P62424|RL7A_HUMAN;tr|Q5T8U3|Q5T8U3_HUMAN;tr|Q5T8U2|Q5T8U2_HUMAN	sp|P62424|RL7A_HUMAN;tr|Q5T8U3|Q5T8U3_HUMAN	150,81	550420000	42	11	0,0026583	-0,689347267	25,66405	25,69988	25,73684	25,23524	25,1031	24,91766	24,77465	24,78322
+	sp|P04083|ANXA1_HUMAN;tr|Q5T3N1|Q5T3N1_HUMAN;tr|Q5T3N0|Q5T3N0_HUMAN	sp|P04083|ANXA1_HUMAN;tr|Q5T3N1|Q5T3N1_HUMAN	46,482	27491000	13	5	0,00267362	-0,800383568	21,1327	21,59094	21,48419	21,15777	20,38005	20,65368	20,32158	20,80876
+	sp|Q9BTT0|AN32E_HUMAN;tr|E9PPH5|E9PPH5_HUMAN;tr|Q5TB19|Q5TB19_HUMAN;tr|E9PLC	sp|Q9BTT0|AN32E_HUMAN;tr|E9PPH5|E9PPH5_HUMAN	21,987	49568000	17	5	0,00271371	-1,640761852	21,72655	22,66562	22,55166	22,56377	21,04434	21,27042	20,45443	20,17536
+	sp|Q6PGP7|TTC37_HUMAN;tr|D6RCE2|D6RCE2_HUMAN	sp|Q6PGP7|TTC37_HUMAN	16,588	33843000	11	8	0,00273207	2,465813637	19,261	18,5908	18,8842	18,94341	21,42347	20,84053	22,73355	20,5451
+	sp|P42766|RL35_HUMAN;tr|F2Z388|F2Z388_HUMAN	sp|P42766|RL35_HUMAN;tr|F2Z388|F2Z388_HUMAN	61,536	292940000	17	3	0,00285157	-1,29214859	25,01014	25,0127	25,23787	24,09418	23,73892	23,50591	23,58967	23,35178
+	sp|P13667|PDIA4_HUMAN	sp|P13667|PDIA4_HUMAN	35,324	157100000	34	12	0,00289328	-0,770794392	23,34109	23,77946	23,64119	23,66427	22,64742	23,10559	22,58472	23,00512
+	sp|P06733|ENOA_HUMAN;tr|K7EM90|K7EM90_HUMAN;sp|P13929|ENOB_HUMAN;sp|P09104	sp|P06733|ENOA_HUMAN	323,31	3310600000	92	19	0,00296564	-0,523750305	27,98354	28,02454	28,06286	28,21002	27,34257	27,59413	27,45591	27,79334
+	sp|Q9BRK5|CAB45_HUMAN;tr|G3V1E2|G3V1E2_HUMAN;tr|H0Y3T6|H0Y3T6_HUMAN	sp|Q9BRK5|CAB45_HUMAN;tr|G3V1E2|G3V1E2_HUMAN;tr|H0Y3T6|H0Y3T6_HUMAN	6,6076	38840000	14	4	0,00298467	1,118905067	20,34505	20,55056	20,4491	20,39686	22,00247	21,82912	21,40468	20,98092
+	sp|Q9H845|ACAD9_HUMAN;tr|H0Y8Z9|H0Y8Z9_HUMAN;tr|D6RCD8|D6RCD8_HUMAN	sp|Q9H845|ACAD9_HUMAN;tr|H0Y8Z9|H0Y8Z9_HUMAN	4,5969	14291000	8	4	0,00310701	1,542518616	18,81863	18,43713	18,62881	18,91751	19,66293	20,60123	20,91612	19,79187
+	sp|P40926|MDHM_HUMAN;tr|G3XAL0|G3XAL0_HUMAN	sp|P40926|MDHM_HUMAN;tr|G3XAL0|G3XAL0_HUMAN	90,861	706180000	35	10	0,00311689	-0,915482521	25,73575	25,9327	26,13128	26,29626	25,0912	25,19809	24,71508	25,42969
+	sp|Q8IXB1|DJC10_HUMAN;tr|E7EP04|E7EP04_HUMAN;tr|A0A087WXH7|A0A087WXH7_HUMAI	sp|Q8IXB1|DJC10_HUMAN;tr|E7EP04|E7EP04_HUMAN	121,25	129590000	23	11	0,00313407	2,479356925	NaN	21,34743	20,78883	20,92586	24,2712	24,01265	22,92582	22,79058
+	sp|O75306|NDUS2_HUMAN	sp|O75306|NDUS2_HUMAN	13,935	49756000	17	7	0,00315491	1,886797905	19,97603	20,15078	20,03255	20,23023	21,75103	22,41343	22,77925	20,99307
+	tr|K7EJT5|K7EJT5_HUMAN;tr|K7EP65|K7EP65_HUMAN;tr|K7EKS7|K7EKS7_HUMAN;tr|K7ELC4|I	tr|K7EJT5|K7EJT5_HUMAN;tr|K7EP65|K7EP65_HUMAN;tr|K7EKS7|K7EKS7_HUMAN;tr|K7ELC4|	11,737	142410000	11	2	0,00321051	-0,76941967	23,71513	23,54581	23,80597	23,31493	22,898	23,09058	22,80843	22,50715
+	sp|P14618|KPYM_HUMAN;tr|H3BTN5|H3BTN5_HUMAN;tr|B4DNK4|B4DNK4_HUMAN;tr|H3BQ	sp|P14618|KPYM_HUMAN;tr|H3BTN5|H3BTN5_HUMAN;tr|B4DNK4|B4DNK4_HUMAN;tr|H3BQ	323,31	1377900000	89	20	0,00321538	-0,857488632	26,63893	27,03044	27,03842	27,07948	26,05654	26,21653	25,69254	26,39172
+	sp|P27348|1433T_HUMAN;tr|E9PG15|E9PG15_HUMAN	sp|P27348|1433T_HUMAN;tr|E9PG15|E9PG15_HUMAN	214,97	177110000	24	8	0,00338744	-0,657623291	23,6903	23,9188	24,01299	23,87493	23,30215	23,45073	22,87185	23,24179
+	tr|J3QR09|J3QR09_HUMAN;tr|J3KTE4|J3KTE4_HUMAN;sp|P84098|RL19_HUMAN;tr|J3QL15|J3	tr|J3QR09|J3QR09_HUMAN;tr|J3KTE4|J3KTE4_HUMAN;sp|P84098|RL19_HUMAN	40,287	291980000	9	3	0,00350743	-0,614631176	24,66871	24,76527	25,02069	24,41893	24,03255	24,10925	24,22817	24,0451
+	sp|P05141|ADT2_HUMAN	sp|P05141|ADT2_HUMAN	23,111	176950000	16	1	0,00361502	0,915919304	22,92136	22,59773	22,62582	22,8697	23,66243	24,15792	23,56139	23,29656
+	sp|P36957|ODO2_HUMAN;tr|Q86SW4|Q86SW4_HUMAN	sp|P36957|ODO2_HUMAN;tr|Q86SW4|Q86SW4_HUMAN	11,1	56567000	12	6	0,00366467	-0,43711853	22,16346	21,95387	22,23634	22,07887	21,79027	21,58284	21,79935	21,51162
+	tr|H7C3I1|H7C3I1_HUMAN;tr|F6VDH7|F6VDH7_HUMAN;tr|Q3KNR6|Q3KNR6_HUMAN;sp|Q8IZ	tr|H7C3I1|H7C3I1_HUMAN;tr|F6VDH7|F6VDH7_HUMAN;tr|Q3KNR6|Q3KNR6_HUMAN;sp|Q8IZ	5,4402	63691000	16	3	0,0038984	-0,645644665	22,16907	22,49346	22,4839	22,5395	21,64821	21,73769	21,61014	22,10732
+	tr|F8W1A4|F8W1A4_HUMAN;sp|P54819|KAD2_HUMAN;tr|F8VZG5|F8VZG5_HUMAN;tr|F8VY0	tr|F8W1A4|F8W1A4_HUMAN;sp|P54819|KAD2_HUMAN;tr|F8VZG5|F8VZG5_HUMAN;tr|F8VY0	77,785	228900000	41	10	0,00417223	-0,901432991	24,14196	24,19144	24,30382	24,57213	23,41442	23,73593	22,90971	23,54357
+	tr|A0A0C4DFV9|A0A0C4DFV9_HUMAN;sp|Q01105|SET_HUMAN;tr|A0A087X027|A0A087X027_	tr|A0A0C4DFV9|A0A0C4DFV9_HUMAN;sp|Q01105|SET_HUMAN;tr|A0A087X027|A0A087X027_	283,38	325600000	25	6	0,00423565	-1,36027813	24,29334	25,26191	25,61172	24,98037	23,70988	23,57057	23,43097	23,99481
+	sp|Q6P1M0|S27A4_HUMAN	sp|Q6P1M0|S27A4_HUMAN	3,0704	6965400	5	3	0,00433192	0,388032118	18,7091	18,49635	18,57913	NaN	18,90416	19,0631	19,07517	18,88914
+	sp|P60842|IF4A1_HUMAN;tr|J3KT12|J3KT12_HUMAN;tr|J3KTB5|J3KTB5_HUMAN;tr|J3QL43|J3	sp|P60842|IF4A1_HUMAN;tr|J3KT12|J3KT12_HUMAN;tr|J3KTB5|J3KTB5_HUMAN;tr|J3QL43|J3	252,46	1187500000	89	9	0,00437112	-0,531524181	26,33514	26,54597	26,62171	26,54146	25,9608	26,10536	25,69726	26,15476
+	sp|P13639|EF2_HUMAN;tr|K7EJ74|K7EJ74_HUMAN;tr|K7EP67|K7EP67_HUMAN	sp|P13639|EF2_HUMAN	323,31	3653900000	150	38	0,00438507	-0,419523239	28,11123	28,08806	28,15957	28,14836	27,68732	27,71424	27,48639	27,94117
+	sp|P49321|NASP_HUMAN;tr|Q5T624|Q5T624_HUMAN;tr|H0YDS9|H0YDS9_HUMAN;tr|E9PRH9	sp|P49321|NASP_HUMAN;tr|Q5T624|Q5T624_HUMAN;tr|H0YDS9|H0YDS9_HUMAN;tr|E9PRH9	71,932	289350000	29	12	0,00454214	-0,670901775	24,68859	24,55713	24,60371	24,55292	23,62344	23,95828	23,81039	24,32663
+	sp|P63104|1433Z_HUMAN;tr|B0AZS6|B0AZS6_HUMAN;tr|E7EX29|E7EX29_HUMAN;tr|B7Z2E6|	sp|P63104|1433Z_HUMAN;tr|B0AZS6|B0AZS6_HUMAN;tr|E7EX29|E7EX29_HUMAN;tr|B7Z2E6|	323,31	398910000	46	8	0,00461586	-0,596014023	25,01945	25,09933	25,03246	25,07867	24,58675	24,77826	24,20105	24,2798
+	tr|F5GXX5|F5GXX5_HUMAN;sp|P61803|DAD1_HUMAN;tr|F5H895|F5H895_HUMAN;tr|A0A0B4	tr|F5GXX5|F5GXX5_HUMAN;sp|P61803|DAD1_HUMAN;tr|F5H895|F5H895_HUMAN;tr|A0A0B4	16,966	66729000	7	4	0,00471883	-1,009846528	22,0036	NaN	22,54903	22,21285	21,52401	21,39765	20,9113	21,1483
+	sp|Q9BVA1|TBB2B_HUMAN;tr|G3V5W4|G3V5W4_HUMAN;tr|G3V2R8|G3V2R8_HUMAN;tr|G3V	sp|Q9BVA1|TBB2B_HUMAN	25,907	407880000	9	2	0,00471946	1,963197231	22,63057	22,86524	22,70725	22,93912	24,84211	23,66362	25,82936	24,65987
+	sp|O75694|NU155_HUMAN;tr|E9PF10|E9PF10_HUMAN	sp|O75694|NU155_HUMAN;tr|E9PF10|E9PF10_HUMAN	20,56	83978000	26	12	0,00478979	-0,746948719	23,06062	22,76203	22,42452	23,11862	22,19003	21,93049	22,19743	22,06006

+	tr|M0R3D6|M0R3D6_HUMAN;tr|M0R1A7|M0R1A7_HUMAN;tr|M0R117|M0R117_HUMAN;sp|	tr|M0R3D6|M0R3D6_HUMAN;tr|M0R1A7|M0R1A7_HUMAN;tr|M0R117|M0R117_HUMAN;sp|	57,728	287110000	28	4	0,00483517	-0,614959717	24,56483	24,63893	24,61526	24,22869	23,79233	23,9178	24,17904	23,69869
+	tr|H3BMH2|H3BMH2_HUMAN;tr|H3BSC1|H3BSC1_HUMAN;sp|P62491|RB11A_HUMAN;sp|Q15	tr|H3BMH2|H3BMH2_HUMAN;tr|H3BSC1|H3BSC1_HUMAN;sp|P62491|RB11A_HUMAN;sp|Q15	6,41	117870000	17	5	0,00493035	-0,622634411	23,1275	23,41274	23,20131	23,36693	22,73246	22,82621	22,27782	22,78145
+	sp|P41227|NAA10_HUMAN;tr|F8W808|F8W808_HUMAN;tr|A8MWP7|A8MWP7_HUMAN;tr|C9	sp|P41227|NAA10_HUMAN;tr|F8W808|F8W808_HUMAN;tr|A8MWP7|A8MWP7_HUMAN	16,91	28980000	8	3	0,00493732	-0,461495399	21,10942	21,06815	21,25032	21,26802	20,7381	20,93272	20,47338	20,70573
+	sp|P41091|IF2G_HUMAN;tr|F8W810|F8W810_HUMAN;sp|Q2VIR3|IF2GL_HUMAN;tr|H7BZU1|	sp|P41091|IF2G_HUMAN;tr|F8W810|F8W810_HUMAN;sp|Q2VIR3|IF2GL_HUMAN	37,002	85161000	23	8	0,00497699	-0,475537777	22,74337	22,53825	22,96395	22,62874	22,31396	22,35114	22,07874	22,22832
+	sp|P16152|CBR1_HUMAN;tr|E9PQ63|E9PQ63_HUMAN;tr|A8MTM1|A8MTM1_HUMAN;sp|O75	sp|P16152|CBR1_HUMAN;tr|E9PQ63|E9PQ63_HUMAN;tr|A8MTM1|A8MTM1_HUMAN	104,5	80448000	24	7	0,00507766	-0,797192574	22,55223	22,89624	22,7977	22,92823	21,90647	22,38601	21,60141	22,09175
+	sp|P04040|CATA_HUMAN	sp|P04040|CATA_HUMAN	6,7078	23459000	12	5	0,00508515	-0,715298653	20,85575	21,24464	21,00313	20,94592	20,47912	20,57487	19,93905	20,1952
+	tr|G3V198|G3V198_HUMAN;sp|Q12769|NU160_HUMAN;tr|E9PR16|E9PR16_HUMAN;tr|E9PSI3	tr|G3V198|G3V198_HUMAN;sp|Q12769|NU160_HUMAN;tr|E9PR16|E9PR16_HUMAN	52,738	47681000	18	8	0,00519233	1,360752106	20,50659	20,59195	20,48787	20,38617	21,08401	22,03275	22,59149	21,70734
+	tr|Q32Q12|Q32Q12_HUMAN;sp|P22392|NDKB_HUMAN;tr|J3KPD9|J3KPD9_HUMAN;sp|O6036	tr|Q32Q12|Q32Q12_HUMAN;sp|P22392|NDKB_HUMAN;tr|J3KPD9|J3KPD9_HUMAN;sp|O6036	117,44	760700000	48	2	0,00525774	-1,004197121	26,03103	25,87371	25,97612	26,46234	25,34736	25,48726	24,72338	24,7684
+	tr|I3L397|I3L397_HUMAN;sp|P63241|IF5A1_HUMAN;tr|I3L504|I3L504_HUMAN;sp|Q6IS14|IF5	tr|I3L397|I3L397_HUMAN;sp|P63241|IF5A1_HUMAN;tr|I3L504|I3L504_HUMAN;sp|Q6IS14|IF5	164,62	458020000	31	6	0,00525882	-0,933624268	25,09865	25,49677	25,31731	25,49945	24,53831	24,92506	24,09031	24,124
+	sp|P38646|GRP75_HUMAN;tr|D6RJI2|D6RJI2_HUMAN;tr|D6RA73|D6RA73_HUMAN;tr|H0Y8S0|	sp|P38646|GRP75_HUMAN	323,31	1118300000	98	23	0,00529435	0,229138851	26,06723	26,00329	25,9514	26,17467	26,3195	26,21334	26,30895	26,27135
+	sp|P25205|MCM3_HUMAN;tr|J3KQ69|J3KQ69_HUMAN;tr|Q7Z6P5|Q7Z6P5_HUMAN	sp|P25205|MCM3_HUMAN;tr|J3KQ69|J3KQ69_HUMAN	84,668	190210000	53	21	0,00531815	0,302826881	23,62098	23,36145	23,41712	23,47469	23,6609	23,74396	23,85994	23,82075
+	tr|M0QXU7|M0QXU7_HUMAN;sp|O43615|TIM44_HUMAN;tr|M0R301|M0R301_HUMAN	tr|M0QXU7|M0QXU7_HUMAN;sp|O43615|TIM44_HUMAN	12,842	61540000	9	5	0,00534169	-0,611789227	22,72386	22,12698	22,31083	22,36022	21,75071	21,92506	21,58522	21,81374
+	tr|A0A087WWJ2|A0A087WWJ2_HUMAN;tr|C9J5D1|C9J5D1_HUMAN;tr|E7EQ69|E7EQ69_HUM	tr|A0A087WWJ2|A0A087WWJ2_HUMAN;tr|C9J5D1|C9J5D1_HUMAN;tr|E7EQ69|E7EQ69_HUM	2,4156	9659500	5	2	0,00534184	-0,422999541	19,58327	19,6855	19,74394	19,83712	19,41844	19,29762	NaN	19,15232
+	tr|C9JRH2|C9JRH2_HUMAN;tr|C9JMJ4|C9JMJ4_HUMAN;tr|C9J3R0|C9J3R0_HUMAN;tr|C9JQZ4|	tr|C9JRH2|C9JRH2_HUMAN;tr|C9JMJ4|C9JMJ4_HUMAN;tr|C9J3R0|C9J3R0_HUMAN;tr|C9JQZ4|	11,338	41493000	7	3	0,00575809	-0,479221821	21,31595	21,66631	21,74763	21,68551	21,26413	21,13496	20,97176	21,12767
+	sp|Q02878|RL6_HUMAN;tr|F8VZ45|F8VZ45_HUMAN;tr|U3KQR5|U3KQR5_HUMAN;tr|F8VR69|	sp|Q02878|RL6_HUMAN	157,98	822330000	41	13	0,00581462	-0,730208397	26,34669	26,27915	26,37003	25,76025	25,45861	25,25764	25,72669	25,39234
+	sp|Q01518|CAP1_HUMAN;tr|Q5T0R7|Q5T0R7_HUMAN;tr|Q5T0R6|Q5T0R6_HUMAN;tr|Q5T0R5	sp|Q01518|CAP1_HUMAN;tr|Q5T0R7|Q5T0R7_HUMAN;tr|Q5T0R6|Q5T0R6_HUMAN;tr|Q5T0R	21,847	154660000	38	11	0,00584187	-0,557275295	23,30466	23,68848	23,87014	23,6633	22,90674	23,10093	23,08594	23,20386
+	sp|P23528|COF1_HUMAN;tr|G3V1A4|G3V1A4_HUMAN;tr|E9PP50|E9PP50_HUMAN;tr|E9PK25	sp|P23528|COF1_HUMAN;tr|G3V1A4|G3V1A4_HUMAN;tr|E9PP50|E9PP50_HUMAN;tr|E9PK25	287,43	486440000	36	9	0,00590807	-1,091351986	25,41616	25,37951	25,21108	25,78995	24,12297	25,04097	24,02732	24,24002
+	sp|P46777|RL5_HUMAN;tr|Q5T7N0|Q5T7N0_HUMAN	sp|P46777|RL5_HUMAN	183,25	240400000	28	8	0,00594497	-0,71329689	24,52773	24,0242	24,34245	24,37776	23,36279	23,52886	23,53611	23,99118
+	sp|P15531|NDKA_HUMAN;tr|E7ERL0|E7ERL0_HUMAN;tr|E5RHP0|E5RHP0_HUMAN;tr|C9K028|	sp|P15531|NDKA_HUMAN;tr|E7ERL0|E7ERL0_HUMAN	31,581	70338000	14	1	0,00604461	-1,161620617	22,36581	22,64315	23,06802	22,65347	21,7205	22,01869	21,45573	20,88904
+	sp|P63173|RL38_HUMAN;tr|J3KT73|J3KT73_HUMAN;tr|J3QL01|J3QL01_HUMAN;tr|J3KSP2|J3K	sp|P63173|RL38_HUMAN;tr|J3KT73|J3KT73_HUMAN;tr|J3QL01|J3QL01_HUMAN	11,243	406450000	19	5	0,00609778	1,137300014	23,50191	23,94976	23,89264	23,88558	25,4788	25,23301	24,72536	24,34191
+	sp|Q9NR30|DDX21_HUMAN	sp|Q9NR30|DDX21_HUMAN	108,78	604670000	82	24	0,00617578	-0,567584515	25,65952	25,60493	25,75307	25,49317	24,87633	24,87465	25,41119	25,07818
+	tr|E7EVA0|E7EVA0_HUMAN;sp|P27816|MAP4_HUMAN;tr|H7C4C5|H7C4C5_HUMAN;tr|H0Y2V1	tr|E7EVA0|E7EVA0_HUMAN;sp|P27816|MAP4_HUMAN	52,996	93443000	29	9	0,00636403	-0,476967335	22,9446	22,90139	23,06112	22,67161	22,19668	22,41186	22,46852	22,5938
+	sp|P06744|G6PI_HUMAN;tr|A0A0A0MTS2|A0A0A0MTS2_HUMAN;tr|K7EQ48|K7EQ48_HUMAN	sp|P06744|G6PI_HUMAN;tr|A0A0A0MTS2|A0A0A0MTS2_HUMAN;tr|K7EQ48|K7EQ48_HUMAN	83,289	327060000	27	9	0,00637343	-0,930177212	24,44474	24,79576	25,24576	24,97259	23,85785	23,99774	23,57358	24,30897
+	sp|P52209|6PGD_HUMAN;tr|K7EMN2|K7EMN2_HUMAN;tr|K7EM49|K7EM49_HUMAN;tr|K7EP	sp|P52209|6PGD_HUMAN;tr|K7EMN2|K7EMN2_HUMAN;tr|K7EM49|K7EM49_HUMAN;tr|K7EF	16,679	70379000	11	6	0,00651081	-0,802506447	22,48753	22,34308	22,86931	22,70774	21,90511	22,03668	21,33172	21,92412
+	sp|Q8NFH3|NUP43_HUMAN	sp|Q8NFH3|NUP43_HUMAN	2,9215	15149000	6	2	0,00688356	0,486164729	NaN	19,78539	19,80945	19,92988	20,3626	20,45222	NaN	20,1684
+	sp|P63244|RACK1_HUMAN;tr|H0YAF8|H0YAF8_HUMAN;tr|D6R9Z1|D6R9Z1_HUMAN;tr|H0YAN	sp|P63244|RACK1_HUMAN;tr|H0YAF8|H0YAF8_HUMAN;tr|D6R9Z1|D6R9Z1_HUMAN;tr|H0YAN	114,79	298750000	22	4	0,00716314	0,558656693	23,57207	24,04935	23,97762	23,86089	24,58463	24,58049	24,28905	24,24038
+	tr|D3YTB1|D3YTB1_HUMAN;sp|P62910|RL32_HUMAN;tr|F8W727|F8W727_HUMAN	tr|D3YTB1|D3YTB1_HUMAN;sp|P62910|RL32_HUMAN;tr|F8W727|F8W727_HUMAN	18,418	204150000	15	3	0,00733527	-0,819906235	24,52337	24,34069	24,28221	23,68368	23,63201	23,45601	23,22897	23,23333
+	sp|P23246|SFPQ_HUMAN;tr|H0Y9K7|H0Y9K7_HUMAN;tr|H0Y9U2|H0Y9U2_HUMAN	sp|P23246|SFPQ_HUMAN	294,96	938820000	86	18	0,00743994	-0,495457649	26,2637	26,058	26,23456	26,15143	25,63857	25,52079	25,54483	26,02168
+	sp|Q04837|SSBP_HUMAN;tr|C9K0U8|C9K0U8_HUMAN;tr|A0A0G2JLD8|A0A0G2JLD8_HUMAN;t	sp|Q04837|SSBP_HUMAN;tr|C9K0U8|C9K0U8_HUMAN;tr|A0A0G2JLD8|A0A0G2JLD8_HUMAN;t	5,1814	171810000	9	4	0,00746978	-0,698662758	23,90701	23,86183	23,8187	23,82182	23,59889	23,24674	22,79223	22,97685
+	tr|H0YN26|H0YN26_HUMAN;sp|P39687|AN32A_HUMAN;tr|H7BZ09|H7BZ09_HUMAN;sp|O956	tr|H0YN26|H0YN26_HUMAN;sp|P39687|AN32A_HUMAN	27,805	108770000	15	4	0,00787889	-1,06729269	22,72821	22,96001	23,74704	23,58646	22,19442	22,28726	21,85125	22,41962
+	tr|C9JXB8|C9JXB8_HUMAN;tr|C9JNW5|C9JNW5_HUMAN;sp|P83731|RL24_HUMAN	tr|C9JXB8|C9JXB8_HUMAN;tr|C9JNW5|C9JNW5_HUMAN;sp|P83731|RL24_HUMAN	30,86	147330000	11	4	0,00838501	-0,911384583	23,69051	23,92561	23,82406	23,35595	23,15675	22,5255	22,68069	NaN
+	sp|P28066|PSA5_HUMAN	sp|P28066|PSA5_HUMAN	51,687	95658000	12	6	0,00848357	-0,30734094	22,69485	22,86435	22,76893	22,8121	22,48013	22,59991	22,3531	NaN
+	sp|Q15427|SF3B4_HUMAN;tr|Q5SZ64|Q5SZ64_HUMAN	sp|Q15427|SF3B4_HUMAN;tr|Q5SZ64|Q5SZ64_HUMAN	17,664	17603000	7	4	0,00886468	-0,301215649	20,40343	20,15165	20,29109	20,12956	19,82299	20,043	19,92829	19,97659
+	sp|Q92522|H1X_HUMAN	sp|Q92522|H1X_HUMAN	25,677	78590000	16	4	0,00887523	-0,802844524	22,50753	23,18345	23,0112	22,38603	22,04103	21,88401	22,16984	21,78197
+	sp|Q9H078|CLPB_HUMAN;tr|H0YGM0|H0YGM0_HUMAN;tr|F5H392|F5H392_HUMAN	sp|Q9H078|CLPB_HUMAN;tr|H0YGM0|H0YGM0_HUMAN	16,662	27626000	9	2	0,00903796	0,835834821	NaN	20,5682	19,90029	20,27401	21,20126	21,03234	21,2815	20,81824
+	sp|Q9P2J5|SYLC_HUMAN;tr|A0A087WXY1|A0A087WXY1_HUMAN	sp|Q9P2J5|SYLC_HUMAN	9,6947	52475000	21	6	0,00905716	-0,528905869	22,15354	22,30129	21,93038	22,08232	21,30952	21,48513	21,80487	21,75238
+	sp|Q00688|FKBP3_HUMAN;tr|G3V5F2|G3V5F2_HUMAN	sp|Q00688|FKBP3_HUMAN	4,2708	43805000	8	3	0,00912528	-0,814116001	21,42578	21,9272	22,0353	22,38422	21,18659	21,2502	20,88122	21,19802
+	sp|Q96CW5|GCP3_HUMAN;tr|A0A087WWB5|A0A087WWB5_HUMAN;tr|A0A087WU06|A0A08	sp|Q96CW5|GCP3_HUMAN	7,528	13511000	6	5	0,00918123	0,452664693	NaN	19,4974	19,31115	19,40195	19,82842	19,73455	20,00553	NaN
+	tr|F8W914|F8W914_HUMAN;sp|Q9NQC3|RTN4_HUMAN;tr|H7C106|H7C106_HUMAN;tr|A0A0	tr|F8W914|F8W914_HUMAN;sp|Q9NQC3|RTN4_HUMAN;tr|H7C106|H7C106_HUMAN;tr|A0A0	19,944	91502000	19	5	0,00934968	-0,46598196	22,72939	22,97072	22,90303	22,8348	22,36172	22,45043	22,6525	22,10936
+	sp|P31939|PUR9_HUMAN;tr|H7C1S2|H7C1S2_HUMAN;tr|F8WEF0|F8WEF0_HUMAN;tr|C9JLK0	sp|P31939|PUR9_HUMAN	112,65	162830000	37	16	0,00954089	-0,705348015	23,59275	23,6872	23,82464	23,96499	22,8678	23,1861	22,71614	23,47816
+	tr|E7EU96|E7EU96_HUMAN;sp|Q8NEV1|CSK23_HUMAN;sp|P68400|CSK21_HUMAN;tr|Q5U5J2	tr|E7EU96|E7EU96_HUMAN;sp|Q8NEV1|CSK23_HUMAN;sp|P68400|CSK21_HUMAN;tr|Q5U5J2	5,4784	20225000	7	4	0,00964983	-0,626465797	20,30263	20,74329	20,96169	20,696	19,78879	20,24656	20,11553	20,04687
+	tr|E7EQR4|E7EQR4_HUMAN;sp|P15311|EZRI_HUMAN	tr|E7EQR4|E7EQR4_HUMAN;sp|P15311|EZRI_HUMAN	111,24	272880000	35	7	0,00967817	-0,4265728	24,29313	24,39034	24,45098	24,57837	23,86041	24,13399	23,81078	24,20135
+	sp|P57088|TMM33_HUMAN;tr|H0Y8N0|H0Y8N0_HUMAN;tr|D6RAA6|D6RAA6_HUMAN	sp|P57088|TMM33_HUMAN;tr|H0Y8N0|H0Y8N0_HUMAN;tr|D6RAA6|D6RAA6_HUMAN	5,296	62096000	11	3	0,00974205	0,714122295	21,5443	21,66049	21,668	21,24284	22,618	22,1134	22,37894	21,86178
+	sp|O75616|ERAL1_HUMAN;tr|J3QT61|J3QT61_HUMAN;tr|J3QSB2|J3QSB2_HUMAN;tr|J3QRV9|	sp|O75616|ERAL1_HUMAN;tr|J3QT61|J3QT61_HUMAN;tr|J3QSB2|J3QSB2_HUMAN;tr|J3QRV9	4,9013	25529000	5	3	0,01008015	0,513780117	20,01672	20,47289	20,37369	20,33625	20,78748	21,09352	20,72709	20,64658
+	sp|Q9H9Y6|RPA2_HUMAN;tr|C9JJG2|C9JJG2_HUMAN;tr|C9JS83|C9JS83_HUMAN	sp|Q9H9Y6|RPA2_HUMAN	6,4863	20118000	10	6	0,01017488	0,94793272	19,78112	19,59244	19,18401	19,64091	19,93027	20,707	20,96247	20,39048
+	sp|Q92769|HDAC2_HUMAN;tr|E5RFI6|E5RFI6_HUMAN;tr|E5RJ04|E5RJ04_HUMAN;tr|E5RGV4|	sp|Q92769|HDAC2_HUMAN;tr|E5RFI6|E5RFI6_HUMAN;tr|E5RJ04|E5RJ04_HUMAN;tr|E5RGV4|	12,714	95073000	10	1	0,01028619	0,383629322	22,05947	22,11181	22,19487	21,997	22,30669	22,44038	22,74772	22,40289
+	sp|P39023|RL3_HUMAN;tr|G5E9G0|G5E9G0_HUMAN;tr|B5MCW2|B5MCW2_HUMAN;tr|H7C3I	sp|P39023|RL3_HUMAN;tr|G5E9G0|G5E9G0_HUMAN;tr|B5MCW2|B5MCW2_HUMAN;tr|H7C3I	305,86	560160000	34	8	0,01032995	-0,537353992	25,57516	25,39617	25,56666	25,1653	24,8775	24,58852	25,10113	24,98672
+	sp|P27695|APEX1_HUMAN;tr|G3V3M6|G3V3M6_HUMAN;tr|G3V5M0|G3V5M0_HUMAN;tr|G3	sp|P27695|APEX1_HUMAN;tr|G3V3M6|G3V3M6_HUMAN;tr|G3V5M0|G3V5M0_HUMAN;tr|G3	35,541	56693000	19	6	0,01039999	-0,815618992	21,97572	22,09607	22,29751	22,23617	21,2577	21,38548	20,84007	21,85973
+	sp|P62258|1433E_HUMAN;tr|K7EM20|K7EM20_HUMAN;tr|K7EIT4|K7EIT4_HUMAN;tr|B4DJF2|	sp|P62258|1433E_HUMAN;tr|K7EM20|K7EM20_HUMAN	302,98	1625500000	59	16	0,01060341	-0,544057846	26,75595	26,89412	27,17988	26,94347	26,22172	26,74535	26,25818	26,37194
+	sp|P54577|SYYC_HUMAN;tr|A0A0C4DGZ5|A0A0C4DGZ5_HUMAN	sp|P54577|SYYC_HUMAN;tr|A0A0C4DGZ5|A0A0C4DGZ5_HUMAN	46,158	270150000	30	15	0,01068888	-0,575435162	24,32389	24,38311	24,58852	24,54657	23,96807	23,83932	23,52013	24,21282
+	sp|Q8TEQ6|GEMI5_HUMAN	sp|Q8TEQ6|GEMI5_HUMAN	8,7732	25150000	10	7	0,01124197	0,635028839	20,20192	20,25523	20,37994	19,8994	20,64869	20,73686	21,24464	20,64641
+	tr|H3BQF1|H3BQF1_HUMAN;sp|P07741|APT_HUMAN;tr|H3BQB1|H3BQB1_HUMAN;tr|H3BSW	tr|H3BQF1|H3BQF1_HUMAN;sp|P07741|APT_HUMAN;tr|H3BQB1|H3BQB1_HUMAN;tr|H3BSW	15,393	172950000	30	6	0,01125575	-0,7317729	23,71513	23,8531	23,72151	24,11347	23,41738	23,40795	22,67618	22,9746
+	sp|P28074|PSB5_HUMAN;tr|H0YJM8|H0YJM8_HUMAN	sp|P28074|PSB5_HUMAN	57,37	161110000	30	8	0,01141585	-0,472658157	23,55309	23,57011	23,82833	23,6936	23,4438	23,12044	22,90663	23,28362
+	sp|Q9UQE7|SMC3_HUMAN	sp|Q9UQE7|SMC3_HUMAN	5,0321	21308000	7	5	0,01141772	-0,350851536	20,56607	20,50795	20,32433	20,35423	20,01468	20,02296	20,22423	NaN
+	sp|P60228|EIF3E_HUMAN;tr|H0YBR5|H0YBR5_HUMAN;tr|E5RGA2|E5RGA2_HUMAN;tr|E5RIT4	sp|P60228|EIF3E_HUMAN;tr|H0YBR5|H0YBR5_HUMAN;tr|E5RGA2|E5RGA2_HUMAN;tr|E5RIT4	10,642	79615000	14	6	0,01197811	-0,320909023	22,55457	22,52378	22,71846	22,59439	22,29499	22,11994	22,20281	22,48981
+	tr|A0A075B730|A0A075B730_HUMAN;tr|A0A087X1U6|A0A087X1U6_HUMAN;sp|P58107|EPIPL	tr|A0A075B730|A0A075B730_HUMAN;tr|A0A087X1U6|A0A087X1U6_HUMAN;sp|P58107|EPIPL	23,268	49679000	18	7	0,01210063	-1,128225803	21,77318	22,7017	22,34199	22,00768	20,61423	21,34716	21,63125	20,719
+	sp|Q9NR31|SAR1A_HUMAN;tr|H0Y5E8|H0Y5E8_HUMAN	sp|Q9NR31|SAR1A_HUMAN;tr|H0Y5E8|H0Y5E8_HUMAN	33,657	313790000	18	3	0,01214241	-1,407602787	24,64488	24,82818	25,44503	24,95762	24,42073	23,46513	23,68024	22,67919
+	sp|O14744|ANM5_HUMAN;tr|C9JSX3|C9JSX3_HUMAN;tr|H0YJX6|H0YJX6_HUMAN;tr|G3V580|	sp|O14744|ANM5_HUMAN;tr|C9JSX3|C9JSX3_HUMAN;tr|H0YJX6|H0YJX6_HUMAN	94,102	108610000	14	8	0,01222411	0,303728104	22,59864	22,57448	22,54026	22,5242	23,03382	22,9287	22,63629	22,85369
+	sp|O95202|LETM1_HUMAN	sp|O95202|LETM1_HUMAN	4,6557	25495000	7	4	0,01226566	-0,875522614	20,60919	21,4314	20,94235	21,65373	20,15882	20,27776	20,2024	20,49561
+	tr|C9JG87|C9JG87_HUMAN;sp|Q9NYK5|RM39_HUMAN	tr|C9JG87|C9JG87_HUMAN;sp|Q9NYK5|RM39_HUMAN	3,5846	9901600	5	2	0,01226851	-0,342995644	19,5514	19,61924	19,65517	19,46653	19,21817	19,25622	19,00939	19,43659
+	sp|P04080|CYTB_HUMAN	sp|P04080|CYTB_HUMAN	8,4856	52927000	11	2	0,01247187	-1,518151919	22,52652	22,43639	22,5004	22,25908	NaN	21,79249	20,19484	20,75001
+	sp|Q15031|SYLM_HUMAN;tr|C9JYR8|C9JYR8_HUMAN;tr|E9PHM2|E9PHM2_HUMAN	sp|Q15031|SYLM_HUMAN;tr|C9JYR8|C9JYR8_HUMAN;tr|E9PHM2|E9PHM2_HUMAN	2,6913	16462000	5	2	0,01264495	0,805853844	19,60771	19,53096	19,40775	19,62038	20,89689	20,52888	19,95291	20,01154
+	sp|O43707|ACTN4_HUMAN;tr|F5GXS2|F5GXS2_HUMAN;tr|H7C144|H7C144_HUMAN;tr|G3V2E	sp|O43707|ACTN4_HUMAN;tr|F5GXS2|F5GXS2_HUMAN;tr|H7C144|H7C144_HUMAN	81,1	167120000	38	11	0,01274842	-0,468725204	23,82036	23,58715	23,78776	23,75124	23,24787	23,24136	22,99099	23,59138
+	sp|P26038|MOES_HUMAN;tr|V9GZ54|V9GZ54_HUMAN	sp|P26038|MOES_HUMAN	22,192	96948000	11	8	0,01288976	-0,498198509	22,64823	22,93971	22,94244	22,7666	22,26705	22,2854	22,08261	22,66912
+	tr|A0A024R4M0|A0A024R4M0_HUMAN;sp|P46781|RS9_HUMAN;tr|B5MCT8|B5MCT8_HUMAN	tr|A0A024R4M0|A0A024R4M0_HUMAN;sp|P46781|RS9_HUMAN;tr|B5MCT8|B5MCT8_HUMAN	26,537	816250000	34	11	0,01295552	-0,61784029	26,14367	26,34349	26,09523	25,72578	25,76476	25,37156	25,51111	25,18937
+	sp|P62269|RS18_HUMAN;tr|A0A0G2JQH2|A0A0G2JQH2_HUMAN;tr|Q5GGW2|Q5GGW2_HUM	sp|P62269|RS18_HUMAN	291,58	1624400000	41	10	0,01302904	0,573236942	26,17465	26,64828	26,3186	26,05759	27,1671	26,69471	26,7753	26,85496
+	sp|Q9UBT2|SAE2_HUMAN;tr|U3KQ93|U3KQ93_HUMAN;tr|K7ESK7|K7ESK7_HUMAN;tr|K7EPL2	sp|Q9UBT2|SAE2_HUMAN	39,042	31281000	16	8	0,0130669	-0,717599869	21,50548	21,50208	21,49194	21,24621	20,31617	21,01903	20,82128	NaN
+	tr|A0A087WT27|A0A087WT27_HUMAN;tr|J3KN95|J3KN95_HUMAN;sp|O95394|AGM1_HUMAI	tr|A0A087WT27|A0A087WT27_HUMAN;tr|J3KN95|J3KN95_HUMAN;sp|O95394|AGM1_HUMAI	37,437	20869000	13	4	0,01308845	1,639611244	19,44113	19,18255	19,38	17,96871	20,18192	21,0229	21,32092	20,00512
+	tr|A0A0D9SF54|A0A0D9SF54_HUMAN;sp|Q13813|SPTN1_HUMAN;tr|A0A0D9SGF6|A0A0D9SGF	tr|A0A0D9SF54|A0A0D9SF54_HUMAN;sp|Q13813|SPTN1_HUMAN;tr|A0A0D9SGF6|A0A0D9SGF	30,093	75435000	29	16	0,01327359	-0,604607582	22,92732	22,69183	22,60815	22,25733	21,91951	21,78085	22,10447	22,26138
+	tr|C9J4M6|C9J4M6_HUMAN;tr|C9J2Y9|C9J2Y9_HUMAN;sp|P30876|RPB2_HUMAN	tr|C9J4M6|C9J4M6_HUMAN;tr|C9J2Y9|C9J2Y9_HUMAN;sp|P30876|RPB2_HUMAN	15,555	37161000	15	10	0,01328791	1,784231981	19,76812	NaN	19,69459	19,84144	20,83969	21,52176	22,68027	21,16742
+	sp|Q15366|PCBP2_HUMAN;tr|H3BRU6|H3BRU6_HUMAN;tr|F8VZX2|F8VZX2_HUMAN;tr|F8W0	sp|Q15366|PCBP2_HUMAN;tr|H3BRU6|H3BRU6_HUMAN;tr|F8VZX2|F8VZX2_HUMAN;tr|F8W0	37,708	142510000	22	6	0,01333092	-0,48533535	23,2339	23,33892	23,60093	23,62344	22,82712	23,08391	22,76048	23,18433
+	tr|F8VZJ2|F8VZJ2_HUMAN;tr|F8W0W4|F8W0W4_HUMAN;tr|H0YHX9|H0YHX9_HUMAN;sp|Q13	tr|F8VZJ2|F8VZJ2_HUMAN;tr|F8W0W4|F8W0W4_HUMAN;tr|H0YHX9|H0YHX9_HUMAN;sp|Q13	9,0649	98724000	14	4	0,013415	-0,989088535	22,62631	23,05909	23,83663	23,04589	22,34262	22,16555	21,77096	22,33243
+	sp|P40429|RL13A_HUMAN;tr|M0QYS1|M0QYS1_HUMAN;sp|Q6NVV1|R13P3_HUMAN;tr|Q8J01	sp|P40429|RL13A_HUMAN;tr|M0QYS1|M0QYS1_HUMAN;sp|Q6NVV1|R13P3_HUMAN;tr|Q8J01	7,4493	229640000	18	4	0,01351677	-0,686992645	24,23922	24,66628	24,31181	24,03364	24,00333	23,47853	23,70398	23,31714
+	tr|C9J9K3|C9J9K3_HUMAN;sp|P08865|RSSA_HUMAN;tr|A0A0C4DG17|A0A0C4DG17_HUMAN;t	tr|C9J9K3|C9J9K3_HUMAN;sp|P08865|RSSA_HUMAN;tr|A0A0C4DG17|A0A0C4DG17_HUMAN;t	323,31	465250000	45	13	0,01380034	-0,61745739	25,12372	25,1106	25,23517	25,43759	24,64807	24,57358	24,21082	25,00479
+	sp|P61247|RS3A_HUMAN;tr|D6RG13|D6RG13_HUMAN;tr|D6RAT0|D6RAT0_HUMAN;tr|H0Y9Y4	sp|P61247|RS3A_HUMAN;tr|D6RG13|D6RG13_HUMAN;tr|D6RAT0|D6RAT0_HUMAN;tr|H0Y9Y4	300,37	500570000	43	8	0,01415593	-0,31191349	25,30998	25,18658	25,34547	25,03523	24,89855	24,9795	25,00517	24,74638
+	sp|P22102|PUR2_HUMAN;tr|C9JBJ1|C9JBJ1_HUMAN;tr|C9JTV6|C9JTV6_HUMAN;tr|F8WD69|F8	sp|P22102|PUR2_HUMAN	111,58	375700000	71	22	0,01419263	-0,266494751	24,7305	24,85291	24,83543	24,77671	24,42349	24,4222	24,55374	24,73014
+	tr|J3KPX7|J3KPX7_HUMAN;sp|Q99623|PHB2_HUMAN;tr|F5GY37|F5GY37_HUMAN;tr|F5H3X6|	tr|J3KPX7|J3KPX7_HUMAN;sp|Q99623|PHB2_HUMAN;tr|F5GY37|F5GY37_HUMAN;tr|F5H3X6|	21,622	68735000	16	10	0,01419859	1,23408556	21,43268	20,61243	20,85003	21,46493	21,68693	23,0925	22,34981	22,16717
+	sp|P00558|PGK1_HUMAN;sp|P07205|PGK2_HUMAN	sp|P00558|PGK1_HUMAN	299,99	841260000	59	14	0,0152625	-0,667688847	25,775	26,29556	26,13046	26,34518	25,4073	25,68563	25,06571	25,7168
+	sp|Q8IYD1|ERF3B_HUMAN	sp|Q8IYD1|ERF3B_HUMAN	14,07	44400000	14	1	0,01529154	-0,28772068	21,60589	21,5904	21,81409	21,55689	21,29401	21,35896	21,23649	21,52693
+	sp|P62314|SMD1_HUMAN;tr|J3QLR7|J3QLR7_HUMAN;tr|J3QLI9|J3QLI9_HUMAN	sp|P62314|SMD1_HUMAN;tr|J3QLR7|J3QLR7_HUMAN;tr|J3QLI9|J3QLI9_HUMAN	4,9921	51791000	13	2	0,01542155	-0,528588772	22,21836	22,01638	22,10053	21,86242	21,17164	21,50281	21,55974	21,84915
+	sp|P51665|PSMD7_HUMAN;tr|H3BNT7|H3BNT7_HUMAN;tr|H3BTM8|H3BTM8_HUMAN	sp|P51665|PSMD7_HUMAN	8,111	34312000	6	6	0,01563391	-0,470638275	21,44743	21,4894	21,24039	21,31976	20,66718	20,9132	21,13044	NaN
+	sp|Q07021|C1QBP_HUMAN;tr|I3L3Q7|I3L3Q7_HUMAN;tr|I3L3B0|I3L3B0_HUMAN	sp|Q07021|C1QBP_HUMAN;tr|I3L3Q7|I3L3Q7_HUMAN;tr|I3L3B0|I3L3B0_HUMAN	298,9	201850000	24	6	0,015653	-0,488958359	24,0023	23,71858	24,33722	23,95056	23,54981	23,70092	23,41571	23,3864
+	sp|P62316|SMD2_HUMAN;tr|K7ERG4|K7ERG4_HUMAN;tr|K7EJB5|K7EJB5_HUMAN	sp|P62316|SMD2_HUMAN;tr|K7ERG4|K7ERG4_HUMAN	11,502	39174000	10	3	0,01600694	-0,332040151	21,84311	21,76498	21,76085	NaN	21,38237	21,61432	21,37613	NaN
+	sp|P61978|HNRPK_HUMAN;tr|Q5T6W2|Q5T6W2_HUMAN;tr|S4R457|S4R457_HUMAN;tr|S4R3	sp|P61978|HNRPK_HUMAN;tr|Q5T6W2|Q5T6W2_HUMAN	294,95	1043200000	90	17	0,01605701	-0,275334358	26,08347	25,98941	26,26166	26,33657	25,82355	25,92341	25,91351	25,90931
+	sp|Q99714|HCD2_HUMAN;tr|Q5H928|Q5H928_HUMAN	sp|Q99714|HCD2_HUMAN;tr|Q5H928|Q5H928_HUMAN	55,523	119800000	29	7	0,01618167	-0,439275742	23,15741	23,21576	23,06149	23,22187	22,69341	23,03066	22,41093	22,76444
+	sp|Q04917|1433F_HUMAN;tr|A2IDB2|A2IDB2_HUMAN	sp|Q04917|1433F_HUMAN;tr|A2IDB2|A2IDB2_HUMAN	25,399	40595000	11	5	0,0169469	-0,686452389	21,24853	22,01981	21,80243	21,85776	21,24336	21,20341	21,04547	20,69047
+	sp|P41250|SYG_HUMAN;tr|H7C443|H7C443_HUMAN	sp|P41250|SYG_HUMAN	38,81	132740000	27	9	0,01698833	-0,326687813	23,08691	23,19625	23,25451	23,32882	22,92767	23,00345	22,63739	22,99124
	tr|D6RHZ5|D6RHZ5_HUMAN;tr|H7BXG7|H7BXG7_HUMAN;sp|O94979|SC31A_HUMAN;tr|D6RE	tr|D6RHZ5|D6RHZ5_HUMAN;tr|H7BXG7|H7BXG7_HUMAN;sp|O94979|SC31A_HUMAN;tr|D6RE	3,4856	19865000	6	3	0,01731335	-0,238198598	NaN	20,70117	20,61522	20,69319	NaN	20,3625	20,39373	20,53877
+	sp|P05455|LA_HUMAN;tr|E7ERC4|E7ERC4_HUMAN;tr|E9PGX9|E9PGX9_HUMAN;tr|E9PFL9|E9I	sp|P05455|LA_HUMAN;tr|E7ERC4|E7ERC4_HUMAN	64,152	129160000	37	8	0,01750869	-0,550943375	23,24013	23,0558	23,51713	23,47023	22,68368	23,09325	22,83505	22,46755

+	sp|P10155|RO60_HUMAN;tr|H0Y9N5|H0Y9N5_HUMAN;tr|G5E9R9|G5E9R9_HUMAN	sp|P10155|RO60_HUMAN	9,4226	64285000	9	4	0,01756114	1,077261766	20,9775	21,04793	20,96261	21,15283	21,8081	22,83798	NaN	21,69136
+	sp|O75937|DNJC8_HUMAN;tr|S4R3J5|S4R3J5_HUMAN	sp|O75937|DNJC8_HUMAN	9,4041	63035000	11	4	0,01769836	-0,498673598	22,37247	22,13734	22,30025	22,46643	NaN	21,78676	21,59587	22,07871
+	sp|Q01082|SPTB2_HUMAN;tr|A0A087WUZ3|A0A087WUZ3_HUMAN;tr|F8W6C1|F8W6C1_HUM	sp|Q01082|SPTB2_HUMAN;tr|A0A087WUZ3|A0A087WUZ3_HUMAN	10,086	50217000	18	8	0,01811352	-0,648217678	22,33759	22,22573	22,1928	21,74288	21,34316	21,15047	21,5475	21,86499
+	tr|M0QXF7|M0QXF7_HUMAN;sp|Q969H8|MYDGF_HUMAN;tr|M0QYN0|M0QYN0_HUMAN	tr|M0QXF7|M0QXF7_HUMAN;sp|Q969H8|MYDGF_HUMAN;tr|M0QYN0|M0QYN0_HUMAN	2,9626	35875000	4	2	0,01836792	-0,706797759	21,37814	21,59108	21,80463	22,17231	21,1283	20,94564	NaN	21,01529
+	sp|Q9H3N1|TMX1_HUMAN;tr|G3V448|G3V448_HUMAN	sp|Q9H3N1|TMX1_HUMAN	33,73	68474000	9	6	0,01843914	-0,542056084	22,79009	22,59823	22,13624	22,58534	21,78009	21,95731	21,95677	22,24751
+	sp|P60900|PSA6_HUMAN;tr|G3V5Z7|G3V5Z7_HUMAN;tr|G3V295|G3V295_HUMAN;tr|G3V3U4	sp|P60900|PSA6_HUMAN;tr|G3V5Z7|G3V5Z7_HUMAN;tr|G3V295|G3V295_HUMAN;tr|G3V3U4	53,591	133050000	23	6	0,0185487	-0,423551083	23,41532	23,28503	23,39807	23,46139	23,17821	23,06199	22,59837	23,02704
+	sp|Q53GQ0|DHB12_HUMAN;tr|E9PI21|E9PI21_HUMAN	sp|Q53GQ0|DHB12_HUMAN	25,339	156220000	19	7	0,01860571	0,903837681	22,25525	23,01803	22,66171	22,29387	23,70314	23,07287	23,95465	23,11356
+	tr|F5GYN4|F5GYN4_HUMAN;sp|Q96FW1|OTUB1_HUMAN;tr|J3KR44|J3KR44_HUMAN;tr|F5H6C	tr|F5GYN4|F5GYN4_HUMAN;sp|Q96FW1|OTUB1_HUMAN;tr|J3KR44|J3KR44_HUMAN;tr|F5H6C	18,629	53110000	20	5	0,01891751	-0,605463982	21,76393	22,28133	22,13	22,0902	21,42281	21,76709	21,0442	21,6095
+	sp|P48047|ATPO_HUMAN;tr|H7C0C1|H7C0C1_HUMAN;tr|H7C086|H7C086_HUMAN;tr|H7C068	sp|P48047|ATPO_HUMAN;tr|H7C0C1|H7C0C1_HUMAN;tr|H7C086|H7C086_HUMAN	8,1415	46092000	14	4	0,01900868	-0,333629131	21,778	21,48949	21,77896	21,51567	21,41132	21,43446	21,2165	21,16533
+	sp|P30050|RL12_HUMAN	sp|P30050|RL12_HUMAN	40,751	384750000	35	6	0,01903145	-0,303929329	24,74929	24,78042	25,00967	24,58228	24,43065	24,57045	24,50045	24,40438
+	sp|P00505|AATM_HUMAN	sp|P00505|AATM_HUMAN	56,656	162360000	18	7	0,0190778	-0,683907032	23,17668	23,88138	23,83287	23,832	23,02989	23,09981	22,61755	23,24006
+	sp|P61086|UBE2K_HUMAN;tr|D6RDM7|D6RDM7_HUMAN	sp|P61086|UBE2K_HUMAN;tr|D6RDM7|D6RDM7_HUMAN	11,718	50281000	14	4	0,01931677	-0,569315434	21,67681	21,85018	22,28311	22,24867	21,35499	21,71733	21,45558	21,25361
+	sp|Q07666|KHDR1_HUMAN	sp|Q07666|KHDR1_HUMAN	59,74	113520000	16	4	0,01932657	-0,467144012	23,29586	22,72823	23,26428	22,97094	22,47778	22,53205	22,62158	22,75931
+	sp|Q53H96|P5CR3_HUMAN;tr|A0A0A0MQS1|A0A0A0MQS1_HUMAN;tr|F8WEI0|F8WEI0_HUM	sp|Q53H96|P5CR3_HUMAN;tr|A0A0A0MQS1|A0A0A0MQS1_HUMAN;tr|F8WEI0|F8WEI0_HUM	3,6983	7054600	2	2	0,01943331	0,791384379	18,51542	NaN	18,37105	18,43718	NaN	19,61829	18,91853	19,16098
+	sp|Q96CS3|FAF2_HUMAN	sp|Q96CS3|FAF2_HUMAN	37,742	23905000	8	5	0,01951841	0,621451855	20,19292	19,88408	20,10303	20,00977	20,794	21,00717	20,73198	20,14246
+	tr|H3BTA2|H3BTA2_HUMAN;sp|P60510|PP4C_HUMAN;tr|H3BV22|H3BV22_HUMAN;tr|I3L4X0|	tr|H3BTA2|H3BTA2_HUMAN;sp|P60510|PP4C_HUMAN;tr|H3BV22|H3BV22_HUMAN	9,2841	18504000	6	2	0,01952442	0,567709605	NaN	19,95205	19,96846	20,25396	20,68723	20,78548	20,40489	NaN
+	sp|P40925|MDHC_HUMAN;tr|B9A041|B9A041_HUMAN;tr|B8ZZ51|B8ZZ51_HUMAN;tr|C9JF79|	sp|P40925|MDHC_HUMAN;tr|B9A041|B9A041_HUMAN;tr|B8ZZ51|B8ZZ51_HUMAN;tr|C9JF79|	24,299	122880000	18	5	0,01964679	-1,065024535	23,29446	23,51953	23,60162	23,7166	NaN	21,97232	22,2764	23,15536
+	sp|Q14166|TTL12_HUMAN	sp|Q14166|TTL12_HUMAN	5,9564	37099000	8	6	0,01987841	-0,47847271	21,33276	21,82427	21,44673	21,45171	21,1266	21,18659	20,71515	21,11324
+	sp|P61313|RL15_HUMAN;tr|E7EQV9|E7EQV9_HUMAN;tr|E7EX53|E7EX53_HUMAN;tr|E7ENU7|	sp|P61313|RL15_HUMAN;tr|E7EQV9|E7EQV9_HUMAN;tr|E7EX53|E7EX53_HUMAN;tr|E7ENU7|	148,12	437700000	24	7	0,02011304	-0,705985069	25,39002	25,53157	25,17695	24,74396	24,22133	24,43714	24,91118	24,44891
+	sp|P19105|ML12A_HUMAN;sp|O14950|ML12B_HUMAN;tr|J3QRS3|J3QRS3_HUMAN;tr|J3KTJ1|	sp|P19105|ML12A_HUMAN;sp|O14950|ML12B_HUMAN;tr|J3QRS3|J3QRS3_HUMAN	8,0677	21446000	7	4	0,02022174	-0,500338554	20,73115	20,43553	20,90866	20,908	20,55684	20,107	20,27196	20,0462
+	tr|E9PIA8|E9PIA8_HUMAN;sp|P50897|PPT1_HUMAN;tr|E9PSE5|E9PSE5_HUMAN;tr|Q5T0S4|Q	tr|E9PIA8|E9PIA8_HUMAN;sp|P50897|PPT1_HUMAN;tr|E9PSE5|E9PSE5_HUMAN;tr|Q5T0S4|Q	3,6415	20623000	3	2	0,02030718	0,993688583	19,59992	19,61367	19,42614	19,79454	21,51176	20,29961	20,43675	20,16091
+	sp|P31946|1433B_HUMAN;tr|Q4VY20|Q4VY20_HUMAN;tr|A0A0J9YWZ2|A0A0J9YWZ2_HUMAN	sp|P31946|1433B_HUMAN	139,49	102330000	22	4	0,02079415	-0,590592861	22,8786	22,75778	23,45526	23,01178	22,44208	22,75022	22,23541	22,31335
+	sp|Q8NI60|ADCK3_HUMAN	sp|Q8NI60|ADCK3_HUMAN	2,9443	7581900	4	3	0,02089976	0,893830935	18,54898	NaN	18,76526	17,89936	19,18015	19,69554	19,19556	19,1222
+	sp|Q13428|TCOF_HUMAN;tr|J3KQ96|J3KQ96_HUMAN;tr|E7ETY2|E7ETY2_HUMAN;tr|H0YA99|	sp|Q13428|TCOF_HUMAN;tr|J3KQ96|J3KQ96_HUMAN;tr|E7ETY2|E7ETY2_HUMAN	9,8033	44300000	12	6	0,02093694	-0,565973282	21,62015	22,10569	21,94314	21,70299	20,91452	21,17475	21,51648	21,50232
	sp|P40938|RFC3_HUMAN;tr|A0A087X270|A0A087X270_HUMAN	sp|P40938|RFC3_HUMAN	8,036	14228000	9	4	0,02183168	0,21730566	19,79877	19,6686	19,64324	19,67071	20,01876	19,8608	20,00758	19,7634
+	sp|P60174|TPIS_HUMAN;tr|U3KPZ0|U3KPZ0_HUMAN;tr|U3KQF3|U3KQF3_HUMAN;tr|U3KPS5|	sp|P60174|TPIS_HUMAN	238,77	576120000	79	13	0,0220559	-0,627157211	25,44436	25,47417	25,55798	25,7304	24,95403	25,09297	24,3763	25,27498
+	sp|P09211|GSTP1_HUMAN;tr|A8MX94|A8MX94_HUMAN;tr|A0A087X2E9|A0A087X2E9_HUMA	sp|P09211|GSTP1_HUMAN;tr|A8MX94|A8MX94_HUMAN	106,79	146430000	11	6	0,02207613	-0,568893433	23,29754	23,28616	23,99757	23,60093	22,88861	23,2123	22,8642	22,94152
+	sp|Q9NTK5|OLA1_HUMAN;tr|J3KQ32|J3KQ32_HUMAN;tr|C9JTK6|C9JTK6_HUMAN	sp|Q9NTK5|OLA1_HUMAN;tr|J3KQ32|J3KQ32_HUMAN;tr|C9JTK6|C9JTK6_HUMAN	4,3863	41770000	5	4	0,02239891	-1,299921036	NaN	21,48267	22,5417	21,6737	20,70033	20,79614	20,30184	NaN
+	sp|O00299|CLIC1_HUMAN	sp|O00299|CLIC1_HUMAN	52,508	117500000	29	9	0,02277999	-0,569822788	22,95216	23,12089	23,10262	23,39428	22,63068	22,74948	22,09642	22,81408
+	tr|E9PLL6|E9PLL6_HUMAN;sp|P46776|RL27A_HUMAN;tr|E9PJD9|E9PJD9_HUMAN;tr|E9PLX7|E	tr|E9PLL6|E9PLL6_HUMAN;sp|P46776|RL27A_HUMAN;tr|E9PJD9|E9PJD9_HUMAN	20,372	265070000	13	3	0,02278772	-0,526943366	24,42892	24,81939	24,55315	24,2281	24,07284	24,05556	23,81294	NaN
+	tr|F8W7C6|F8W7C6_HUMAN;tr|A0A087WV22|A0A087WV22_HUMAN;tr|X1WI28|X1WI28_HUN	tr|F8W7C6|F8W7C6_HUMAN;tr|A0A087WV22|A0A087WV22_HUMAN;tr|X1WI28|X1WI28_HUN	19,698	264680000	22	5	0,0229246	-0,677290916	24,76243	24,37425	24,89004	23,97229	23,71156	23,65862	24,01026	23,9094
+	sp|P43490|NAMPT_HUMAN;tr|A0A0C4DFS8|A0A0C4DFS8_HUMAN	sp|P43490|NAMPT_HUMAN;tr|A0A0C4DFS8|A0A0C4DFS8_HUMAN	6,1468	32838000	12	5	0,02325624	-0,442714691	21,154	21,36922	21,55791	21,76276	21,1432	20,91145	20,89349	21,1249
+	sp|Q15008|PSMD6_HUMAN;tr|H7C531|H7C531_HUMAN;tr|C9J7B7|C9J7B7_HUMAN;tr|C9J0E9	sp|Q15008|PSMD6_HUMAN	21,132	44770000	17	7	0,0234856	-0,345909278	21,72713	22,0126	21,94831	21,71791	21,37968	21,62574	21,51133	NaN
+	sp|P36578|RL4_HUMAN;tr|H3BM89|H3BM89_HUMAN;tr|H3BTP7|H3BTP7_HUMAN;tr|H3BU31	sp|P36578|RL4_HUMAN;tr|H3BM89|H3BM89_HUMAN	28,989	400950000	25	6	0,02357385	-0,461757183	24,91365	24,95978	25,21608	24,80055	24,30327	24,38726	24,87122	24,4813
+	sp|Q04637|IF4G1_HUMAN;tr|E7EX73|E7EX73_HUMAN;tr|E9PGM1|E9PGM1_HUMAN;tr|E7EU	sp|Q04637|IF4G1_HUMAN;tr|E7EX73|E7EX73_HUMAN;tr|E9PGM1|E9PGM1_HUMAN;tr|E7EU	123,35	208480000	30	16	0,02366756	-0,499803066	24,23535	24,12123	24,17843	23,73603	23,31147	23,49826	23,56744	23,89467
+	tr|H0Y2W2|H0Y2W2_HUMAN;sp|Q9NVI7|ATD3A_HUMAN;tr|Q5SV16|Q5SV16_HUMAN	tr|H0Y2W2|H0Y2W2_HUMAN;sp|Q9NVI7|ATD3A_HUMAN	29,313	93794000	15	1	0,02445898	1,082183361	21,46643	21,67642	21,08284	22,74917	22,92573	22,94324	22,70824	22,72638
+	tr|A0A0A0MSQ0|A0A0A0MSQ0_HUMAN;sp|P13797|PLST_HUMAN;sp|P13796|PLSL_HUMAN;tr	tr|A0A0A0MSQ0|A0A0A0MSQ0_HUMAN;sp|P13797|PLST_HUMAN	37,702	191170000	27	9	0,02471147	-0,573723793	23,43314	24,14686	23,97334	24,02496	23,22202	23,56848	23,06819	23,4247
+	sp|P32119|PRDX2_HUMAN;tr|A6NIW5|A6NIW5_HUMAN	sp|P32119|PRDX2_HUMAN;tr|A6NIW5|A6NIW5_HUMAN	9,433	163500000	14	3	0,02483824	-0,415596008	23,49863	23,84995	23,62979	23,70535	23,48493	23,42803	22,998	23,11038
+	tr|J3KT10|J3KT10_HUMAN;sp|Q9BW27|NUP85_HUMAN;tr|J3KSH3|J3KSH3_HUMAN;tr|J3KRC0	tr|J3KT10|J3KT10_HUMAN;sp|Q9BW27|NUP85_HUMAN;tr|J3KSH3|J3KSH3_HUMAN;tr|J3KRC0	7,0456	29948000	7	6	0,02500511	1,495354176	19,90046	19,96353	20,01386	19,0044	20,16066	21,43781	20,98273	22,28246
+	sp|P52907|CAZA1_HUMAN;tr|C9JUG7|C9JUG7_HUMAN;tr|F8W9N7|F8W9N7_HUMAN;tr|A0A0	sp|P52907|CAZA1_HUMAN	13,75	56916000	20	6	0,02537738	-0,285956383	21,824	21,88342	22,16628	22,059	21,61063	21,81386	21,5925	21,77189
+	sp|P30086|PEBP1_HUMAN	sp|P30086|PEBP1_HUMAN	7,9942	140690000	16	5	0,02554978	-0,588324547	23,41326	23,34096	23,2037	23,96296	23,09875	22,98531	22,58898	22,89454
+	tr|K7EQA1|K7EQA1_HUMAN;sp|O14737|PDCD5_HUMAN;tr|K7ESJ4|K7ESJ4_HUMAN	tr|K7EQA1|K7EQA1_HUMAN;sp|O14737|PDCD5_HUMAN	5,0121	21463000	8	3	0,02622635	-0,396943569	20,6759	20,56179	20,90911	20,80262	20,2815	20,645	20,10534	20,32981
+	sp|P53680|AP2S1_HUMAN;tr|M0R0N4|M0R0N4_HUMAN;tr|M0QYZ2|M0QYZ2_HUMAN;tr|M0	sp|P53680|AP2S1_HUMAN;tr|M0R0N4|M0R0N4_HUMAN;tr|M0QYZ2|M0QYZ2_HUMAN;tr|M0	2,5375	41933000	3	3	0,02626943	0,770856857	21,23175	20,89608	20,62194	20,6943	21,74624	22,21798	21,33041	21,23286
	tr|B1AHB1|B1AHB1_HUMAN;sp|P33992|MCM5_HUMAN;tr|B1AHB2|B1AHB2_HUMAN;tr|B1AH	tr|B1AHB1|B1AHB1_HUMAN;sp|P33992|MCM5_HUMAN	22,119	53991000	15	5	0,02741495	-0,254722118	22,13824	22,06494	21,7634	22,07051	21,76652	21,67857	21,76624	21,80688
+	sp|P55060|XPO2_HUMAN	sp|P55060|XPO2_HUMAN	116,49	340470000	67	26	0,0275836	-0,450892448	24,44563	24,82881	24,6634	24,78985	23,97448	24,28849	24,09273	24,56842
+	sp|Q13263|TIF1B_HUMAN;tr|M0R0K9|M0R0K9_HUMAN;tr|M0R3C0|M0R3C0_HUMAN;tr|M0R	sp|Q13263|TIF1B_HUMAN;tr|M0R0K9|M0R0K9_HUMAN	151,02	301080000	32	16	0,02784337	0,644395828	23,62288	23,56267	24,23352	23,96137	24,30452	24,2839	24,40912	24,96049
+	sp|Q14247|SRC8_HUMAN;tr|H0YEV2|H0YEV2_HUMAN;tr|H0YCD9|H0YCD9_HUMAN	sp|Q14247|SRC8_HUMAN	7,7053	21512000	7	4	0,02806582	-0,330437342	NaN	20,72983	20,7159	20,75638	20,57598	20,39582	20,239	NaN
+	sp|P62753|RS6_HUMAN;tr|A2A3R7|A2A3R7_HUMAN;tr|A2A3R5|A2A3R5_HUMAN	sp|P62753|RS6_HUMAN;tr|A2A3R7|A2A3R7_HUMAN	14,549	179590000	14	3	0,02883162	-1,092433453	23,81029	23,94101	24,64702	23,61964	22,60198	23,83634	22,53424	22,67566
+	tr|K7ENG2|K7ENG2_HUMAN;sp|P26368|U2AF2_HUMAN	tr|K7ENG2|K7ENG2_HUMAN;sp|P26368|U2AF2_HUMAN	21,179	43261000	15	4	0,02884216	-0,444363117	21,48704	21,61225	21,84869	21,72034	20,83105	21,42726	21,37066	21,2619
+	sp|P09972|ALDOC_HUMAN;tr|K7EKH5|K7EKH5_HUMAN;tr|C9J8F3|C9J8F3_HUMAN;tr|J3KSV6|	sp|P09972|ALDOC_HUMAN;tr|K7EKH5|K7EKH5_HUMAN;tr|C9J8F3|C9J8F3_HUMAN;tr|J3KSV6|	18,473	42890000	9	2	0,02885066	-0,751086076	NaN	21,8385	22,18807	21,45141	20,94871	21,24592	20,73115	21,37385
+	tr|A0A087WUT6|A0A087WUT6_HUMAN;sp|O60841|IF2P_HUMAN	tr|A0A087WUT6|A0A087WUT6_HUMAN;sp|O60841|IF2P_HUMAN	5,1968	26140000	7	4	0,02909065	-0,606412888	20,91765	21,06415	21,38047	20,7997	19,93962	20,73066	20,50911	20,55693
+	sp|Q13162|PRDX4_HUMAN;tr|H7C3T4|H7C3T4_HUMAN;tr|A6NG45|A6NG45_HUMAN;tr|A6NJ	sp|Q13162|PRDX4_HUMAN;tr|H7C3T4|H7C3T4_HUMAN	29,15	260170000	28	6	0,02963216	-0,400496006	24,36306	24,18026	24,23856	24,33108	23,96173	23,93715	23,49228	24,11981
+	tr|A0A087WTP3|A0A087WTP3_HUMAN;sp|Q92945|FUBP2_HUMAN;tr|M0R0I5|M0R0I5_HUMA	tr|A0A087WTP3|A0A087WTP3_HUMAN;sp|Q92945|FUBP2_HUMAN;tr|M0R0I5|M0R0I5_HUMA	83,796	351590000	47	15	0,03082711	-0,435122013	24,72208	24,72401	24,79074	24,97075	24,39598	24,30862	24,03313	24,72936
+	sp|Q16658|FSCN1_HUMAN;tr|C9JFC0|C9JFC0_HUMAN;tr|A0A0A0MSB2|A0A0A0MSB2_HUMAN	sp|Q16658|FSCN1_HUMAN	81,022	419780000	56	14	0,03142751	-0,404056072	24,80386	24,92823	24,97976	25,07928	24,45086	24,74319	24,20978	24,77107
+	sp|Q15365|PCBP1_HUMAN;tr|F8VTZ0|F8VTZ0_HUMAN;tr|H3BSP4|H3BSP4_HUMAN;tr|C9K0A2	sp|Q15365|PCBP1_HUMAN	241,45	404520000	42	6	0,03234676	-0,495625019	24,9608	25,00058	24,82512	25,27352	24,64422	24,46675	24,12502	24,84153
+	tr|B1AHF3|B1AHF3_HUMAN;sp|P00387|NB5R3_HUMAN	tr|B1AHF3|B1AHF3_HUMAN;sp|P00387|NB5R3_HUMAN	8,5449	43656000	6	2	0,03247584	-0,469380856	21,45634	21,6868	22,09513	21,65351	21,27333	21,4913	21,26459	20,98503
	sp|O43242|PSMD3_HUMAN	sp|O43242|PSMD3_HUMAN	35,956	62642000	18	10	0,03268939	-0,167665005	22,00518	22,1379	22,23468	22,04434	21,93506	21,91506	22,02736	21,87395
	sp|P63208|SKP1_HUMAN;tr|E7ERH2|E7ERH2_HUMAN;tr|F8W8N3|F8W8N3_HUMAN;tr|E5RJR5	sp|P63208|SKP1_HUMAN;tr|E7ERH2|E7ERH2_HUMAN;tr|F8W8N3|F8W8N3_HUMAN;tr|E5RJR5	31,048	47289000	10	3	0,03396073	0,285891056	21,55178	21,2921	21,24383	21,47581	21,62796	21,72521	21,85289	21,50101
+	sp|P14923|PLAK_HUMAN;tr|C9JTX4|C9JTX4_HUMAN;tr|C9J826|C9J826_HUMAN;tr|C9JK18|C9	sp|P14923|PLAK_HUMAN	14,16	52100000	14	7	0,03404141	-1,140361786	22,59092	23,1437	21,48812	22,09462	21,62819	21,02513	21,46174	20,64086
+	sp|P49915|GUAA_HUMAN	sp|P49915|GUAA_HUMAN	30,691	108200000	28	10	0,03411056	-0,345901012	22,63761	22,98888	23,12589	23,04737	22,56458	22,71678	22,43334	22,70145
+	sp|P10515|ODP2_HUMAN;tr|H0YDD4|H0YDD4_HUMAN;tr|E9PEJ4|E9PEJ4_HUMAN	sp|P10515|ODP2_HUMAN;tr|H0YDD4|H0YDD4_HUMAN;tr|E9PEJ4|E9PEJ4_HUMAN	11,235	29519000	15	4	0,03485199	-0,989527702	21,29132	21,20174	21,59455	21,52492	20,56895	20,86469	20,84437	19,37641
+	sp|P31948|STIP1_HUMAN;tr|F5H783|F5H783_HUMAN;tr|F5GXD8|F5GXD8_HUMAN;tr|H0YGI8	sp|P31948|STIP1_HUMAN;tr|F5H783|F5H783_HUMAN;tr|F5GXD8|F5GXD8_HUMAN	44,63	326340000	33	12	0,03519777	-0,449115753	24,21978	24,67126	24,75129	24,92746	24,15352	24,3927	24,0721	24,15499
+	sp|P12956|XRCC6_HUMAN;tr|B1AHC9|B1AHC9_HUMAN	sp|P12956|XRCC6_HUMAN;tr|B1AHC9|B1AHC9_HUMAN	153,96	425010000	61	18	0,03540524	-0,416163921	24,80247	24,95527	25,0542	25,16645	24,30299	24,82813	24,78397	24,39865
+	sp|P25786|PSA1_HUMAN;tr|F5GX11|F5GX11_HUMAN;tr|B4DEV8|B4DEV8_HUMAN	sp|P25786|PSA1_HUMAN;tr|F5GX11|F5GX11_HUMAN;tr|B4DEV8|B4DEV8_HUMAN	22,768	83272000	14	6	0,0354676	-0,372682571	22,25488	22,64665	22,80136	22,28122	22,11709	22,09278	22,08528	22,19823
	tr|D6RD66|D6RD66_HUMAN;sp|O75083|WDR1_HUMAN	tr|D6RD66|D6RD66_HUMAN;sp|O75083|WDR1_HUMAN	4,5092	14281000	2	3	0,03602224	-0,162070274	19,86097	19,90718	19,89996	20,11108	19,79357	19,73757	19,7632	19,83657
+	sp|Q15293|RCN1_HUMAN;tr|E9PP27|E9PP27_HUMAN	sp|Q15293|RCN1_HUMAN	47,289	69569000	16	6	0,03606879	0,696277618	21,37369	22,23357	21,55211	21,52903	22,55642	22,36842	21,87938	22,66928
	sp|Q9HAV4|XPO5_HUMAN;tr|H0Y9I3|H0Y9I3_HUMAN	sp|Q9HAV4|XPO5_HUMAN	4,0811	11734000	8	4	0,03632218	-0,283772945	19,7312	19,78545	19,80708	19,81731	19,27935	19,60708	19,38315	19,73638
+	sp|O75369|FLNB_HUMAN;tr|E7EN95|E7EN95_HUMAN	sp|O75369|FLNB_HUMAN;tr|E7EN95|E7EN95_HUMAN	20,611	53953000	27	13	0,03633214	-0,621090412	22,20353	22,32149	22,31005	21,90481	21,04946	21,44081	21,73032	22,03493
+	sp|P56545|CTBP2_HUMAN;tr|Q5SQP8|Q5SQP8_HUMAN;tr|E9PGB1|E9PGB1_HUMAN;tr|E7EUE	sp|P56545|CTBP2_HUMAN;tr|Q5SQP8|Q5SQP8_HUMAN	11,175	11951000	8	3	0,03645986	-0,475417455	NaN	19,80573	20,0713	19,96084	NaN	19,61072	19,59623	19,20467
	tr|G5E9R3|G5E9R3_HUMAN;tr|M0R0A1|M0R0A1_HUMAN;tr|C9J4Z3|C9J4Z3_HUMAN;tr|Q6P4	tr|G5E9R3|G5E9R3_HUMAN;tr|M0R0A1|M0R0A1_HUMAN;tr|C9J4Z3|C9J4Z3_HUMAN;tr|Q6P4	2,4585	59791000	7	3	0,03664276	-0,294931412	NaN	22,33276	22,2265	21,96716	21,97631	21,85878	21,91397	21,7731
+	sp|Q9Y266|NUDC_HUMAN;tr|A0A0A0MSS4|A0A0A0MSS4_HUMAN;tr|A0A0A0MSU9|A0A0A0M	sp|Q9Y266|NUDC_HUMAN	5,0729	99511000	8	5	0,0367068	-0,473195076	22,64454	22,77746	22,93959	23,00012	22,15035	22,82052	22,15694	22,34112
	sp|Q13283|G3BP1_HUMAN;tr|E5RIZ6|E5RIZ6_HUMAN;tr|E5RH00|E5RH00_HUMAN;tr|D6R9X5	sp|Q13283|G3BP1_HUMAN	69,872	139130000	28	6	0,03672985	-0,266420364	23,23488	23,11962	23,41995	23,41687	22,88565	22,94969	23,13215	23,15815
+	sp|P39748|FEN1_HUMAN;tr|I3L3E9|I3L3E9_HUMAN;tr|F5H1Y3|F5H1Y3_HUMAN	sp|P39748|FEN1_HUMAN;tr|I3L3E9|I3L3E9_HUMAN;tr|F5H1Y3|F5H1Y3_HUMAN	21,474	38008000	14	4	0,03757439	-0,502916813	21,43553	22,00388	21,63781	21,63112	21,59751	21,14065	21,02486	20,93366
+	sp|O75821|EIF3G_HUMAN;tr|K7EP16|K7EP16_HUMAN;tr|K7ER90|K7ER90_HUMAN;tr|K7EL20|	sp|O75821|EIF3G_HUMAN;tr|K7EP16|K7EP16_HUMAN;tr|K7ER90|K7ER90_HUMAN;tr|K7EL20|	18,907	91998000	18	5	0,03766112	-0,557163239	22,33655	22,7274	22,96932	22,82018	21,8338	22,49962	22,35348	21,9379
+	sp|P82979|SARNP_HUMAN;tr|F8VZQ9|F8VZQ9_HUMAN;tr|H0YHG0|H0YHG0_HUMAN	sp|P82979|SARNP_HUMAN;tr|F8VZQ9|F8VZQ9_HUMAN;tr|H0YHG0|H0YHG0_HUMAN	7,2802	63375000	15	4	0,03804926	-0,323263645	22,42029	22,01229	22,22535	22,37692	21,90746	21,80306	21,86476	22,16653
+	sp|O76031|CLPX_HUMAN	sp|O76031|CLPX_HUMAN	16,04	49262000	6	7	0,03817021	0,60101827	20,92412	21,37729	21,31705	21,3811	NaN	22,01971	22,08994	21,44309
+	tr|E7EQB2|E7EQB2_HUMAN;tr|E7ER44|E7ER44_HUMAN;sp|P02788|TRFL_HUMAN;tr|C9JCF5|C	tr|E7EQB2|E7EQB2_HUMAN;tr|E7ER44|E7ER44_HUMAN;sp|P02788|TRFL_HUMAN	5,1511	27395000	11	4	0,03884417	-0,859153271	22,14808	20,95333	20,97155	21,53227	20,91481	20,14358	20,45944	20,65079
+	sp|P11177|ODPB_HUMAN;tr|C9J634|C9J634_HUMAN;tr|F8WF02|F8WF02_HUMAN	sp|P11177|ODPB_HUMAN;tr|C9J634|C9J634_HUMAN	19,204	42708000	7	5	0,03892641	0,423658848	21,11794	20,67444	21,18646	21,37883	21,55154	21,47338	21,65779	21,36959
+	sp|P23588|IF4B_HUMAN;tr|E7EX17|E7EX17_HUMAN;tr|F8W0K0|F8W0K0_HUMAN;tr|F8VRU1|	sp|P23588|IF4B_HUMAN;tr|E7EX17|E7EX17_HUMAN	13,417	59830000	10	3	0,04020749	-0,437034607	22,29732	22,37132	22,2596	22,2676	NaN	21,98458	21,4958	22,1054
+	tr|A0A140T936|A0A140T936_HUMAN;sp|P26640|SYVC_HUMAN;tr|A0A140T954|A0A140T954_	tr|A0A140T936|A0A140T936_HUMAN;sp|P26640|SYVC_HUMAN	38,311	63452000	24	11	0,04080661	-0,46546936	22,25716	22,56225	22,00624	22,46827	22,23681	21,71255	21,66887	21,81382
+	sp|Q96PK6|RBM14_HUMAN;tr|A0A0A6YYI9|A0A0A6YYI9_HUMAN;tr|F8WDX3|F8WDX3_HUMA	sp|Q96PK6|RBM14_HUMAN	18,241	82957000	18	9	0,04081104	0,390176296	22,23515	22,13019	22,24348	22,17871	22,67373	22,14995	22,72752	22,79703
+	sp|P62081|RS7_HUMAN;tr|B5MCP9|B5MCP9_HUMAN	sp|P62081|RS7_HUMAN;tr|B5MCP9|B5MCP9_HUMAN	24,424	251770000	21	5	0,04290173	-0,560080051	24,40646	24,30452	24,55713	24,08716	23,38851	23,95376	24,24096	23,53172
+	tr|A0A0C4DGI3|A0A0C4DGI3_HUMAN;tr|B4DJV2|B4DJV2_HUMAN;sp|O75390|CISY_HUMAN;tr	tr|A0A0C4DGI3|A0A0C4DGI3_HUMAN;tr|B4DJV2|B4DJV2_HUMAN;sp|O75390|CISY_HUMAN	72,532	444870000	41	9	0,04298975	-0,461630344	25,05846	25,09442	25,10322	25,30414	24,81142	24,67487	24,20903	25,01839
+	sp|P26641|EF1G_HUMAN	sp|P26641|EF1G_HUMAN	119,63	572530000	51	10	0,04329094	-0,368853092	25,34194	25,43435	25,57888	25,50812	25,19551	25,04143	24,75471	25,39624
	sp|P20618|PSB1_HUMAN	sp|P20618|PSB1_HUMAN	14,238	137110000	13	7	0,0438934	-0,313484192	23,23498	23,17811	23,22582	23,43797	23,17936	23,0412	22,6644	22,93799
+	tr|A0A087WZH7|A0A087WZH7_HUMAN;sp|P29966|MARCS_HUMAN	tr|A0A087WZH7|A0A087WZH7_HUMAN;sp|P29966|MARCS_HUMAN	216,74	173920000	34	6	0,0439693	-0,689446926	23,6147	23,70398	23,85823	23,52097	22,80392	23,46799	23,33879	22,3294
+	sp|Q9BQ39|DDX50_HUMAN;tr|A0A087WVC1|A0A087WVC1_HUMAN	sp|Q9BQ39|DDX50_HUMAN;tr|A0A087WVC1|A0A087WVC1_HUMAN	5,8621	37400000	15	5	0,04404501	-0,349396229	21,77688	21,38906	21,4314	21,34262	21,10591	20,88398	21,22305	21,32943
+	tr|H0Y8E6|H0Y8E6_HUMAN;sp|P49736|MCM2_HUMAN	tr|H0Y8E6|H0Y8E6_HUMAN;sp|P49736|MCM2_HUMAN	32,421	104890000	38	12	0,04448233	-0,471290112	23,26728	23,16665	23,01243	22,79622	22,41589	22,49833	22,39312	23,05008
+	sp|P07954|FUMH_HUMAN	sp|P07954|FUMH_HUMAN	17,141	37825000	12	5	0,0446785	-0,436800003	21,26562	21,58045	21,70683	21,69859	21,01679	21,14345	20,84537	21,49868

	sp|P02786|TFR1_HUMAN;tr|G3V0E5|G3V0E5_HUMAN;tr|H7C3V5|H7C3V5_HUMAN;tr|F8WBE5	sp|P02786|TFR1_HUMAN;tr|G3V0E5|G3V0E5_HUMAN	10,259	44524000	10	7	0,04512877	-0,245581627	21,60639	21,75422	21,59291	21,60883	21,18973	21,3009	21,56137	21,52803
	sp|Q13155|AIMP2_HUMAN;tr|A8MU58|A8MU58_HUMAN;tr|F8W950|F8W950_HUMAN	sp|Q13155|AIMP2_HUMAN;tr|A8MU58|A8MU58_HUMAN;tr|F8W950|F8W950_HUMAN	6,8678	14442000	5	3	0,0477264	-0,258010864	19,94235	19,67883	20,05973	20,05245	19,54772	19,80747	19,69518	19,65094
+	tr|M0QWZ7|M0QWZ7_HUMAN;sp|Q9NP81|SYSM_HUMAN;tr|B4DJM9|B4DJM9_HUMAN;tr|M(	tr|M0QWZ7|M0QWZ7_HUMAN;sp|Q9NP81|SYSM_HUMAN;tr|B4DJM9|B4DJM9_HUMAN;tr|M(	4,235	25979000	8	4	0,04783429	0,579963684	20,31717	20,35165	20,32301	20,39194	21,48513	21,08128	20,74296	20,39425
+	sp|Q02790|FKBP4_HUMAN;tr|H0YFG2|H0YFG2_HUMAN;tr|F5H1U3|F5H1U3_HUMAN	sp|Q02790|FKBP4_HUMAN	120,83	261940000	35	14	0,0481356	-0,450437069	24,08205	24,36486	24,53996	24,61689	23,9486	24,08529	23,56663	24,2015
+	sp|P46821|MAP1B_HUMAN;tr|D6RA32|D6RA32_HUMAN;tr|D6RCL2|D6RCL2_HUMAN;tr|D6RG	sp|P46821|MAP1B_HUMAN;tr|D6RA32|D6RA32_HUMAN	5,6041	10276000	5	4	0,04814222	0,630137444	18,93759	18,88369	18,71102	18,88313	18,96101	19,70737	20,06999	19,19761
+	tr|G3V203|G3V203_HUMAN;sp|Q07020|RL18_HUMAN;tr|J3QQ67|J3QQ67_HUMAN;tr|H0YHA7	tr|G3V203|G3V203_HUMAN;sp|Q07020|RL18_HUMAN;tr|J3QQ67|J3QQ67_HUMAN;tr|H0YHA7	183,54	341810000	21	6	0,04844183	-0,661359787	25,17919	25,29302	25,0599	24,4279	24,85191	24,03733	24,32697	24,09836
+	sp|P23526|SAHH_HUMAN	sp|P23526|SAHH_HUMAN	55,992	389680000	28	7	0,04898085	-0,357131004	24,55941	24,83788	24,88721	24,82211	24,26549	24,76981	24,21756	24,42522
+	sp|P78347|GTF2I_HUMAN	sp|P78347|GTF2I_HUMAN	79,061	38222000	18	6	0,04955229	-0,67649889	22,22506	20,90272	21,56263	21,48488	20,90095	20,80349	20,76474	21,0001
	sp|P06576|ATPB_HUMAN;tr|H0YH81|H0YH81_HUMAN;tr|F8W079|F8W079_HUMAN;tr|F8W0I	sp|P06576|ATPB_HUMAN;tr|H0YH81|H0YH81_HUMAN;tr|F8W079|F8W079_HUMAN	323,31	600530000	74	15	0,04986613	0,179504871	25,09362	25,04293	25,14663	25,32306	25,31437	25,43259	25,34201	25,23528
+	sp|P43686|PRS6B_HUMAN	sp|P43686|PRS6B_HUMAN	26,722	35922000	6	4	0,04993586	0,591880798	20,64878	20,46981	20,87252	20,85392	20,80112	21,83248	21,11566	21,46329
+	sp|P61081|UBC12_HUMAN;tr|M0QX69|M0QX69_HUMAN;tr|M0QYI6|M0QYI6_HUMAN	sp|P61081|UBC12_HUMAN	16,362	45684000	17	5	0,05052219	-0,672322909	NaN	22,00038	21,8723	22,09139	21,51581	21,41225	20,68123	21,65351
+	sp|Q9Y3F4|STRAP_HUMAN;tr|H0YH33|H0YH33_HUMAN	sp|Q9Y3F4|STRAP_HUMAN	18,552	99513000	16	7	0,05080591	-0,654750347	22,61648	22,98163	23,10181	23,09692	22,59181	22,68389	22,29367	21,60847
+	tr|A0A087WVM4|A0A087WVM4_HUMAN;sp|Q6UB35|C1TM_HUMAN;tr|B7ZM99|B7ZM99_HU	tr|A0A087WVM4|A0A087WVM4_HUMAN;sp|Q6UB35|C1TM_HUMAN;tr|B7ZM99|B7ZM99_HU	26,457	223840000	35	14	0,05141246	0,428504467	23,38903	23,3232	23,61088	23,53552	23,56813	23,71272	23,97832	24,31347
+	sp|Q99536|VAT1_HUMAN;tr|K7ERT7|K7ERT7_HUMAN;tr|K7EJM4|K7EJM4_HUMAN;tr|K7ER81	sp|Q99536|VAT1_HUMAN;tr|K7ERT7|K7ERT7_HUMAN	30,726	21659000	9	5	0,05162074	-0,60836935	20,43685	20,56653	21,24144	20,77435	20,09352	20,62827	20,09854	19,76536
+	sp|P23284|PPIB_HUMAN	sp|P23284|PPIB_HUMAN	17,803	65828000	8	4	0,05185497	-0,941598892	22,19736	21,71749	23,13189	22,98612	21,46707	21,58261	21,12692	22,08987
+	sp|Q96QK1|VPS35_HUMAN	sp|Q96QK1|VPS35_HUMAN	15,746	32901000	14	7	0,0525898	-0,395576	21,45046	21,31396	21,18144	21,34044	20,88733	20,73595	20,70649	21,37422
+	tr|A0A0A0MSI0|A0A0A0MSI0_HUMAN;sp|Q06830|PRDX1_HUMAN;tr|A0A0A0MRQ5|A0A0A0M	tr|A0A0A0MSI0|A0A0A0MSI0_HUMAN;sp|Q06830|PRDX1_HUMAN	25,947	2886700000	36	7	0,05292747	-0,408394814	27,48516	27,82705	27,66099	27,91028	27,52103	27,56935	26,97509	27,18442
+	sp|Q96P70|IPO9_HUMAN	sp|Q96P70|IPO9_HUMAN	80,507	21735000	10	4	0,05367894	0,687067032	20,3469	19,8142	19,92647	20,53934	20,72758	20,56076	20,563	21,52382
	sp|P38919|IF4A3_HUMAN;tr|I3L3H2|I3L3H2_HUMAN	sp|P38919|IF4A3_HUMAN	15,213	45714000	11	5	0,05374339	0,189486027	21,37719	21,51735	21,37183	21,32092	21,4402	21,73909	21,51523	21,65071
+	sp|P18077|RL35A_HUMAN;tr|F8WBS5|F8WBS5_HUMAN;tr|F8WB72|F8WB72_HUMAN;tr|C9K(	sp|P18077|RL35A_HUMAN;tr|F8WBS5|F8WBS5_HUMAN;tr|F8WB72|F8WB72_HUMAN;tr|C9K(	76,291	385120000	11	5	0,05482654	-0,710458279	24,95593	25,59446	25,11577	24,48757	24,49454	23,76638	24,63378	24,41719
+	sp|Q12788|TBL3_HUMAN;tr|J3KNP2|J3KNP2_HUMAN;tr|A0A087WYP7|A0A087WYP7_HUMAN	sp|Q12788|TBL3_HUMAN;tr|J3KNP2|J3KNP2_HUMAN;tr|A0A087WYP7|A0A087WYP7_HUMAN	5,5018	9777300	7	4	0,05510277	0,474680424	18,9061	18,89454	18,91713	19,06834	18,9502	19,50996	19,8919	19,33276
+	tr|M0R026|M0R026_HUMAN;sp|A1L0T0|ILVBL_HUMAN;tr|M0R1B5|M0R1B5_HUMAN;tr|E9PJ!	tr|M0R026|M0R026_HUMAN;sp|A1L0T0|ILVBL_HUMAN;tr|M0R1B5|M0R1B5_HUMAN;tr|E9PJ!	60,854	31529000	10	5	0,0554821	1,49037981	NaN	19,45932	19,73319	19,66706	19,78556	21,65923	22,08317	20,91298
+	sp|P61088|UBE2N_HUMAN;tr|F8VZ29|F8VZ29_HUMAN;tr|F8VSD4|F8VSD4_HUMAN;tr|F8VV71	sp|P61088|UBE2N_HUMAN;tr|F8VZ29|F8VZ29_HUMAN;tr|F8VSD4|F8VSD4_HUMAN;tr|F8VV71	12,864	63229000	7	3	0,05595312	-0,996338367	21,6197	22,09394	22,88276	23,24766	21,50033	21,94095	21,46623	20,9512
+	sp|Q9BZX2|UCK2_HUMAN;sp|Q9HA47|UCK1_HUMAN	sp|Q9BZX2|UCK2_HUMAN	7,5105	20202000	9	5	0,0577204	0,389351368	19,88178	20,23795	20,13521	20,29569	20,3932	20,73727	20,20048	20,77708
+	sp|Q9BQA1|MEP50_HUMAN;tr|H0Y711|H0Y711_HUMAN	sp|Q9BQA1|MEP50_HUMAN;tr|H0Y711|H0Y711_HUMAN	12,859	31014000	10	4	0,05844179	0,606595993	20,32003	20,37284	20,77113	20,45964	21,39482	21,5935	20,75888	20,61702
	sp|Q13347|EIF3I_HUMAN;tr|Q5TFK1|Q5TFK1_HUMAN	sp|Q13347|EIF3I_HUMAN	25,429	101130000	22	8	0,05853696	-0,356485367	22,70898	22,8596	23,23664	22,78734	22,57309	22,73107	22,26508	22,59737
	sp|Q15645|PCH2_HUMAN;tr|H0YAL2|H0YAL2_HUMAN	sp|Q15645|PCH2_HUMAN	5,546	37683000	7	4	0,05930415	0,259716988	20,82912	21,04527	21,07294	21,13984	21,41736	21,40857	21,03436	21,26574
+	sp|P61604|CH10_HUMAN;tr|B8ZZL8|B8ZZL8_HUMAN;tr|S4R3N1|S4R3N1_HUMAN;tr|B8ZZ54|I	sp|P61604|CH10_HUMAN;tr|B8ZZL8|B8ZZL8_HUMAN;tr|S4R3N1|S4R3N1_HUMAN	55,388	237180000	14	6	0,0600002	0,700476964	NaN	23,53445	23,06886	23,80193	24,56477	24,3678	23,68603	24,05697
	sp|Q92499|DDX1_HUMAN;tr|F1T0B3|F1T0B3_HUMAN;tr|A0A087X2G1|A0A087X2G1_HUMAN	sp|Q92499|DDX1_HUMAN;tr|F1T0B3|F1T0B3_HUMAN;tr|A0A087X2G1|A0A087X2G1_HUMAN	82,242	72139000	33	10	0,06011566	-0,343268394	22,34101	22,28382	22,42322	22,50758	21,67719	22,27125	22,2569	21,97722
+	tr|E7EPB3|E7EPB3_HUMAN;sp|P50914|RL14_HUMAN	tr|E7EPB3|E7EPB3_HUMAN;sp|P50914|RL14_HUMAN	8,6939	246370000	13	3	0,06231429	-0,601507664	24,85334	24,06497	24,61127	23,81411	23,65185	23,5438	24,04618	23,69583
+	tr|F8W1K5|F8W1K5_HUMAN;tr|F8VXJ7|F8VXJ7_HUMAN;sp|Q9Y2B0|CNPY2_HUMAN;tr|F8W0:	tr|F8W1K5|F8W1K5_HUMAN;tr|F8VXJ7|F8VXJ7_HUMAN;sp|Q9Y2B0|CNPY2_HUMAN;tr|F8W0:	22,818	18762000	9	4	0,06244805	-0,454566479	20,19713	20,79725	20,89852	20,64386	NaN	20,25327	20,05748	20,22811
	sp|P50991|TCPD_HUMAN	sp|P50991|TCPD_HUMAN	165,38	812450000	66	20	0,06310362	-0,285310745	25,57764	25,89384	25,86689	26,1541	25,56506	25,66947	25,47822	25,63848
+	sp|P61353|RL27_HUMAN;tr|K7ELC7|K7ELC7_HUMAN;tr|K7EQQ9|K7EQQ9_HUMAN;tr|K7ERY7	sp|P61353|RL27_HUMAN;tr|K7ELC7|K7ELC7_HUMAN;tr|K7EQQ9|K7EQQ9_HUMAN	24,056	190470000	12	4	0,0634605	-0,501555443	23,74479	24,03322	23,82182	23,39545	23,56605	23,40639	23,26556	22,75105
+	sp|P30048|PRDX3_HUMAN	sp|P30048|PRDX3_HUMAN	14,312	135750000	28	6	0,06412876	-0,510133743	23,18828	23,26857	23,67228	23,89966	23,0845	23,20086	23,25368	23,14922
+	sp|P62191|PRS4_HUMAN;tr|G3V4X1|G3V4X1_HUMAN	sp|P62191|PRS4_HUMAN	105,45	88205000	10	7	0,06413009	0,399179459	22,20494	22,50756	22,30349	22,36175	22,25419	22,85933	22,89877	22,96215
	tr|J3QT28|J3QT28_HUMAN;sp|O43684|BUB3_HUMAN;tr|J3QSX4|J3QSX4_HUMAN	tr|J3QT28|J3QT28_HUMAN;sp|O43684|BUB3_HUMAN;tr|J3QSX4|J3QSX4_HUMAN	10,966	57097000	13	4	0,06426207	-0,277745724	21,89682	22,04507	22,12682	22,08648	21,90415	21,73723	21,45408	21,94874
+	tr|A0A087WZZ5|A0A087WZZ5_HUMAN;sp|Q13435|SF3B2_HUMAN;tr|E9PJ04|E9PJ04_HUMAN	tr|A0A087WZZ5|A0A087WZZ5_HUMAN;sp|Q13435|SF3B2_HUMAN;tr|E9PJ04|E9PJ04_HUMAN	14,899	88058000	16	6	0,06436892	-0,428311825	22,8226	22,69515	22,81423	22,42278	21,98423	22,01764	22,35251	22,68714
+	sp|Q9Y696|CLIC4_HUMAN	sp|Q9Y696|CLIC4_HUMAN	12,052	23291000	6	3	0,06455772	-0,466456413	20,57339	20,97218	20,57967	21,02709	20,76182	20,12149	20,01713	20,38606
	sp|P62266|RS23_HUMAN;tr|D6RD47|D6RD47_HUMAN;tr|D6RDJ2|D6RDJ2_HUMAN;tr|D6RIX0	sp|P62266|RS23_HUMAN;tr|D6RD47|D6RD47_HUMAN	32,37	323820000	24	7	0,06463604	-0,391702175	24,95323	24,66855	24,23411	24,28263	24,21971	24,14383	24,1536	24,05457
+	tr|M0R0P1|M0R0P1_HUMAN;tr|M0R299|M0R299_HUMAN;tr|M0QXL5|M0QXL5_HUMAN;tr|M	tr|M0R0P1|M0R0P1_HUMAN;tr|M0R299|M0R299_HUMAN;tr|M0QXL5|M0QXL5_HUMAN;tr|M	6,8239	33751000	4	3	0,06505102	0,751845837	20,5966	20,22176	20,49775	20,37624	20,73207	21,04627	22,12414	20,79725
	sp|P52292|IMA1_HUMAN;tr|J3QLL0|J3QLL0_HUMAN;tr|J3KS65|J3KS65_HUMAN	sp|P52292|IMA1_HUMAN;tr|J3QLL0|J3QLL0_HUMAN;tr|J3KS65|J3KS65_HUMAN	70,427	167110000	30	8	0,06567912	0,322941303	23,40315	23,05755	23,34394	23,14886	23,52013	23,44063	23,37822	23,90628
+	sp|Q9H0U4|RAB1B_HUMAN;tr|E9PLD0|E9PLD0_HUMAN;tr|A0A087WTI1|A0A087WTI1_HUMAI	sp|Q9H0U4|RAB1B_HUMAN;tr|E9PLD0|E9PLD0_HUMAN;tr|A0A087WTI1|A0A087WTI1_HUMAI	183,78	163580000	24	2	0,06667763	-0,641528606	23,27256	24,14709	23,29516	23,42021	22,86671	23,43505	22,78213	22,48503
	sp|Q9NZI8|IF2B1_HUMAN	sp|Q9NZI8|IF2B1_HUMAN	88,672	311700000	40	12	0,06683914	0,345441818	24,01699	23,71796	24,19648	23,90408	24,06102	24,16875	24,58469	24,40282
	sp|P15880|RS2_HUMAN;tr|E9PQD7|E9PQD7_HUMAN;tr|H0YEN5|H0YEN5_HUMAN;tr|E9PMM!	sp|P15880|RS2_HUMAN;tr|E9PQD7|E9PQD7_HUMAN;tr|H0YEN5|H0YEN5_HUMAN;tr|E9PMM!	80,599	791100000	37	8	0,06703049	-0,291960239	25,83048	26,06919	25,92802	25,52046	25,67906	25,55183	25,56098	25,38844
	tr|B1AK87|B1AK87_HUMAN;tr|B1AK88|B1AK88_HUMAN;tr|B1AK85|B1AK85_HUMAN;sp|P477	tr|B1AK87|B1AK87_HUMAN;tr|B1AK88|B1AK88_HUMAN;tr|B1AK85|B1AK85_HUMAN;sp|P477	4,157	23261000	9	3	0,06706537	-0,375564575	20,78093	20,89178	20,62658	20,81495	20,77467	20,53687	20,05378	20,24667
	sp|P29401|TKT_HUMAN;tr|A0A0B4J1R6|A0A0B4J1R6_HUMAN;tr|E9PFF2|E9PFF2_HUMAN;tr|F	sp|P29401|TKT_HUMAN;tr|A0A0B4J1R6|A0A0B4J1R6_HUMAN	51,114	582600000	37	15	0,06781055	-0,326499462	25,24104	25,43317	25,27402	25,62642	24,94855	25,18835	24,80582	25,32591
	sp|Q5VTE0|EF1A3_HUMAN;sp|P68104|EF1A1_HUMAN;tr|A0A087WV01|A0A087WV01_HUMAI	sp|Q5VTE0|EF1A3_HUMAN;sp|P68104|EF1A1_HUMAN;tr|A0A087WV01|A0A087WV01_HUMAI	323,31	8615500000	84	11	0,06859263	0,157012939	28,83744	29,05938	28,9493	29,1159	29,12196	29,25169	29,10262	29,11379
	sp|P30041|PRDX6_HUMAN	sp|P30041|PRDX6_HUMAN	242,47	302420000	47	10	0,06908716	-0,396425724	24,2617	24,45419	24,501	24,64312	24,34001	24,34422	23,71712	23,87296
	tr|E5RHW4|E5RHW4_HUMAN;sp|O94905|ERLN2_HUMAN;tr|E5RJ09|E5RJ09_HUMAN;tr|B0QZ4	tr|E5RHW4|E5RHW4_HUMAN;sp|O94905|ERLN2_HUMAN;tr|E5RJ09|E5RJ09_HUMAN;tr|B0QZ4	8,1707	46480000	9	5	0,06931958	0,215353489	21,47308	21,30374	21,49492	21,51152	21,91284	21,54274	21,60558	21,58353
	tr|C9J8M3|C9J8M3_HUMAN;tr|C9JZI1|C9JZI1_HUMAN;sp|P35249|RFC4_HUMAN;tr|C9JXZ7|C9	tr|C9J8M3|C9J8M3_HUMAN;tr|C9JZI1|C9JZI1_HUMAN;sp|P35249|RFC4_HUMAN;tr|C9JXZ7|C9	1,7462	21725000	6	2	0,06967797	0,223766804	20,26465	20,38501	20,37093	20,17779	20,78636	20,46364	20,39163	20,45181
+	sp|Q9BQ67|GRWD1_HUMAN;tr|M0QX71|M0QX71_HUMAN	sp|Q9BQ67|GRWD1_HUMAN;tr|M0QX71|M0QX71_HUMAN	7,3949	19100000	8	4	0,06983388	0,469418844	20,30508	NaN	20,43573	20,07848	20,46184	20,75001	21,0157	NaN
	sp|Q86VP6|CAND1_HUMAN;tr|A0A0C4DGH5|A0A0C4DGH5_HUMAN;tr|H0YH27|H0YH27_HUM	sp|Q86VP6|CAND1_HUMAN;tr|A0A0C4DGH5|A0A0C4DGH5_HUMAN	26,168	192440000	38	16	0,06999916	-0,40903759	23,99826	23,74263	23,75236	24,01052	23,21105	23,32375	23,36413	23,96869
+	sp|O00410|IPO5_HUMAN;tr|H0Y8C6|H0Y8C6_HUMAN;tr|E7ETV3|E7ETV3_HUMAN;tr|E7EQT5|	sp|O00410|IPO5_HUMAN;tr|H0Y8C6|H0Y8C6_HUMAN	114,79	117920000	33	11	0,07102946	-0,487422466	23,15643	23,12127	23,53457	23,30076	22,579	22,56041	22,62939	23,39454
	sp|P46109|CRKL_HUMAN	sp|P46109|CRKL_HUMAN	2,9743	7408000	4	2	0,0711893	-0,219335397	NaN	19,0162	19,10034	19,29134	18,77684	18,94567	18,89205	19,05195
+	tr|A0A087WX29|A0A087WX29_HUMAN;tr|B1AKP7|B1AKP7_HUMAN;tr|G3V162|G3V162_HUM	tr|A0A087WX29|A0A087WX29_HUMAN;tr|B1AKP7|B1AKP7_HUMAN;tr|G3V162|G3V162_HUM	23,477	52700000	5	3	0,07158843	0,499164104	20,79408	21,40432	21,35515	21,83996	21,61764	21,96088	21,93614	21,87549
	sp|Q8WUM4|PDC6I_HUMAN;tr|F8WDK9|F8WDK9_HUMAN	sp|Q8WUM4|PDC6I_HUMAN	40,711	56211000	22	9	0,07221416	-0,287000179	22,0624	22,12921	21,95269	22,09291	21,73698	21,83642	21,45367	22,06214
+	sp|P35637|FUS_HUMAN;tr|H3BPE7|H3BPE7_HUMAN;tr|A0A075B7D9|A0A075B7D9_HUMAN;s	sp|P35637|FUS_HUMAN;tr|H3BPE7|H3BPE7_HUMAN	69,795	86463000	12	4	0,07286724	-0,856314659	22,69905	22,7091	23,13863	22,87994	22,4465	22,54875	22,12884	20,89519
+	sp|P04259|K2C6B_HUMAN	sp|P04259|K2C6B_HUMAN	163,61	610840000	60	1	0,0734617	-1,418388367	25,6126	27,11734	24,89606	26,09033	25,36429	23,6413	25,24514	23,79203
	tr|A0A0B4J2E5|A0A0B4J2E5_HUMAN;sp|Q15269|PWP2_HUMAN	tr|A0A0B4J2E5|A0A0B4J2E5_HUMAN;sp|Q15269|PWP2_HUMAN	5,0086	9690500	6	2	0,07409676	0,344618956	19,01362	NaN	19,05502	19,10352	19,11319	19,43011	19,73047	19,33425
+	sp|Q15691|MARE1_HUMAN	sp|Q15691|MARE1_HUMAN	15,913	84595000	21	6	0,07434192	-0,660497189	22,0746	23,02933	23,08876	22,92038	22,31703	22,09529	21,58545	22,47331
	sp|P07814|SYEP_HUMAN;tr|V9GYZ6|V9GYZ6_HUMAN;tr|V9GZ76|V9GZ76_HUMAN	sp|P07814|SYEP_HUMAN;tr|V9GYZ6|V9GYZ6_HUMAN	134,53	265510000	39	19	0,07437472	0,366682053	24,02293	23,80499	24,00624	23,62187	24,03984	23,97954	24,30682	24,59656
	sp|P16403|H12_HUMAN;sp|P10412|H14_HUMAN;sp|P16402|H13_HUMAN;sp|P22492|H1T_H	sp|P16403|H12_HUMAN;sp|P10412|H14_HUMAN;sp|P16402|H13_HUMAN	71,322	1495000000	40	8	0,07475707	-0,225836754	26,6476	26,7082	26,7571	26,7057	26,19342	26,50253	26,67936	26,53993
+	sp|Q15843|NEDD8_HUMAN;tr|F8VSA6|F8VSA6_HUMAN;tr|E9PS38|E9PS38_HUMAN;tr|S4R3E9	sp|Q15843|NEDD8_HUMAN;tr|F8VSA6|F8VSA6_HUMAN;tr|E9PS38|E9PS38_HUMAN;tr|S4R3E9	1,8077	9127100	2	2	0,07509098	0,461065928	NaN	19,07041	19,13786	18,67131	19,27733	19,79308	19,14803	19,46527
	tr|B5MCX3|B5MCX3_HUMAN;sp|Q15019|SEPT2_HUMAN;tr|C9J2Q4|C9J2Q4_HUMAN;tr|C9IY9	tr|B5MCX3|B5MCX3_HUMAN;sp|Q15019|SEPT2_HUMAN;tr|C9J2Q4|C9J2Q4_HUMAN;tr|C9IY9	46,892	44933000	17	7	0,07518187	-0,311841488	21,62885	21,58063	21,67189	21,7402	21,55272	21,31689	20,95419	21,55042
	tr|G5EA06|G5EA06_HUMAN;sp|Q92552|RT27_HUMAN;tr|D6RJC7|D6RJC7_HUMAN;tr|D6RH20	tr|G5EA06|G5EA06_HUMAN;sp|Q92552|RT27_HUMAN;tr|D6RJC7|D6RJC7_HUMAN;tr|D6RH20	2,1188	18637000	4	2	0,07530489	0,254801273	20,09995	20,34006	20,29412	20,17865	20,26075	20,57015	20,61809	NaN
+	sp|Q71DI3|H32_HUMAN	sp|Q71DI3|H32_HUMAN	250,06	2575100000	19	1	0,07615468	1,572467327	25,45164	24,70007	26,65499	26,22601	25,70279	27,65799	28,54657	27,41522
	sp|Q99832|TCPH_HUMAN;tr|F8WAM2|F8WAM2_HUMAN	sp|Q99832|TCPH_HUMAN	104,33	723810000	69	15	0,07626191	-0,147293091	25,50963	25,61608	25,55195	25,74065	25,51881	25,48873	25,31759	25,504
	sp|P12004|PCNA_HUMAN	sp|P12004|PCNA_HUMAN	7,1592	68543000	9	4	0,08043469	-0,32506752	22,09291	22,30627	22,55705	22,53174	22,36303	21,97722	21,8519	21,99555
	sp|P28838|AMPL_HUMAN;tr|H0Y983|H0Y983_HUMAN;tr|H0Y9Q1|H0Y9Q1_HUMAN	sp|P28838|AMPL_HUMAN	20,556	31896000	9	6	0,08328562	-0,352873802	21,14781	21,20234	NaN	21,50349	20,98266	20,9507	20,62266	21,17066
+	sp|P40937|RFC5_HUMAN;tr|E9PEP3|E9PEP3_HUMAN;tr|F5H5S0|F5H5S0_HUMAN	sp|P40937|RFC5_HUMAN	4,9116	53601000	5	4	0,08349595	0,533476353	21,68752	21,52172	21,69417	21,61679	22,05854	22,4066	21,5048	22,68417
	sp|P38117|ETFB_HUMAN;tr|M0QY67|M0QY67_HUMAN	sp|P38117|ETFB_HUMAN	13,915	116010000	22	7	0,08381332	-0,458454132	23,12398	23,20044	23,04348	23,41274	23,0234	22,63429	22,19559	23,09354
	sp|P22314|UBA1_HUMAN;tr|Q5JRR6|Q5JRR6_HUMAN;tr|Q5JRR9|Q5JRR9_HUMAN;tr|Q5JRS0|	sp|P22314|UBA1_HUMAN	323,31	554990000	89	22	0,08460817	-0,44711256	25,46105	25,40224	25,40688	25,48551	24,95082	24,86802	24,56058	25,58781
	sp|P63261|ACTG_HUMAN;sp|P60709|ACTB_HUMAN;sp|P63267|ACTH_HUMAN;sp|P68133|AC	sp|P63261|ACTG_HUMAN;sp|P60709|ACTB_HUMAN;sp|P63267|ACTH_HUMAN;sp|P68133|AC	323,31	1,1263E+10	85	12	0,08616405	-0,39780283	29,47628	29,72224	29,73841	30,04726	29,41689	29,33153	28,94737	29,69719
	sp|P51148|RAB5C_HUMAN;tr|F8VVK3|F8VVK3_HUMAN;tr|K7ERI8|K7ERI8_HUMAN;tr|K7ERQ8	sp|P51148|RAB5C_HUMAN;tr|F8VVK3|F8VVK3_HUMAN;tr|K7ERI8|K7ERI8_HUMAN;tr|K7ERQ8	100,44	93073000	28	2	0,08727119	-0,458457186	22,29061	23,15372	22,65755	22,79275	22,56519	22,42335	22,06121	22,02594
	sp|O75534|CSDE1_HUMAN;tr|E9PLT0|E9PLT0_HUMAN;tr|E9PLD4|E9PLD4_HUMAN;tr|E9PNG3	sp|O75534|CSDE1_HUMAN;tr|E9PLT0|E9PLT0_HUMAN	17,046	84755000	17	10	0,08737773	-0,170923233	22,32501	22,66941	22,54738	22,59735	22,39796	22,25278	22,37472	22,43
	sp|Q7KZF4|SND1_HUMAN;tr|H7C597|H7C597_HUMAN	sp|Q7KZF4|SND1_HUMAN	32,138	156400000	50	13	0,08758826	-0,323251247	23,61189	23,4325	23,66275	23,64724	23,26799	23,05336	23,05303	23,68699
	sp|O43776|SYNC_HUMAN;tr|K7EQ35|K7EQ35_HUMAN;tr|K7EJ19|K7EJ19_HUMAN;tr|K7EIU7|I	sp|O43776|SYNC_HUMAN	40,6	81152000	27	10	0,08810745	0,37095499	21,63914	21,98096	22,22075	22,16947	22,12921	22,56134	22,61899	22,18459
	sp|O14818|PSA7_HUMAN;tr|H0Y586|H0Y586_HUMAN;sp|Q8TAA3|PSA7L_HUMAN;tr|F5GY34|	sp|O14818|PSA7_HUMAN;tr|H0Y586|H0Y586_HUMAN	40,174	136350000	23	8	0,08915091	-0,306781292	23,29081	23,01419	23,54085	23,27014	23,1817	23,08508	22,69347	22,92861
	sp|P49588|SYAC_HUMAN;tr|H3BPK7|H3BPK7_HUMAN	sp|P49588|SYAC_HUMAN;tr|H3BPK7|H3BPK7_HUMAN	14,057	82194000	13	6	0,08939716	-0,456945419	22,41232	22,75609	22,78921	22,63657	22,55852	21,86522	21,79563	22,54706
	sp|Q9Y333|LSM2_HUMAN	sp|Q9Y333|LSM2_HUMAN	18,665	6663100	7	2	0,08948242	-0,205940406	18,83306	18,9851	19,08755	18,95589	18,74931	18,92135	18,60773	NaN
	sp|Q96T76|MMS19_HUMAN;tr|H0Y746|H0Y746_HUMAN;tr|H7C1W5|H7C1W5_HUMAN;tr|Q5'	sp|Q96T76|MMS19_HUMAN	10,816	26867000	7	7	0,09024501	0,227381229	20,63343	20,60648	20,64403	20,38911	20,61468	20,91772	20,65255	20,99762
	sp|Q15459|SF3A1_HUMAN;tr|F8WC79|F8WC79_HUMAN;tr|F8WB66|F8WB66_HUMAN	sp|Q15459|SF3A1_HUMAN	16,783	68708000	15	7	0,09090692	-0,330760956	22,34589	22,22926	22,52499	22,28116	21,78089	21,7383	22,18737	22,3517
	sp|O00303|EIF3F_HUMAN;tr|H0YDT6|H0YDT6_HUMAN;tr|E9PQV8|E9PQV8_HUMAN;tr|A0A0D	sp|O00303|EIF3F_HUMAN	51,276	53206000	11	5	0,0920955	0,273540974	21,2307	21,86283	21,6759	21,71469	21,91944	21,87916	21,87605	21,90364
+	sp|Q15181|IPYR_HUMAN;tr|Q5SQT6|Q5SQT6_HUMAN	sp|Q15181|IPYR_HUMAN;tr|Q5SQT6|Q5SQT6_HUMAN	12,569	156450000	16	6	0,09238405	-0,550578594	23,2535	23,50942	23,74366	24,09362	22,93184	23,59269	22,61767	23,25768
	sp|Q14103|HNRPD_HUMAN;tr|H0YA96|H0YA96_HUMAN;tr|H0Y8G5|H0Y8G5_HUMAN;tr|D6R/	sp|Q14103|HNRPD_HUMAN;tr|H0YA96|H0YA96_HUMAN;tr|H0Y8G5|H0Y8G5_HUMAN;tr|D6R/	58,601	250710000	17	7	0,09325007	-0,429338455	23,86844	23,71931	24,1141	24,23965	23,27469	23,51905	24,07553	23,35488
+	sp|Q13404|UB2V1_HUMAN;tr|I3L0A0|I3L0A0_HUMAN;tr|A0A0A0MSL3|A0A0A0MSL3_HUMAN	sp|Q13404|UB2V1_HUMAN;tr|I3L0A0|I3L0A0_HUMAN;tr|A0A0A0MSL3|A0A0A0MSL3_HUMAN	3,9341	65575000	10	3	0,09381718	-0,60307471	22,05815	NaN	22,36185	22,4032	21,90437	22,15761	21,07666	21,54666
+	sp|P28340|DPOD1_HUMAN;tr|M0R2B7|M0R2B7_HUMAN;tr|M0QZR8|M0QZR8_HUMAN;tr|M(	sp|P28340|DPOD1_HUMAN;tr|M0R2B7|M0R2B7_HUMAN;tr|M0QZR8|M0QZR8_HUMAN	6,0462	15385000	10	5	0,09444932	0,562349637	NaN	19,95674	19,05022	19,94392	19,94707	20,33963	20,31628	20,2476
	tr|E9PKP7|E9PKP7_HUMAN;sp|P17480|UBF1_HUMAN;tr|E9PLT2|E9PLT2_HUMAN	tr|E9PKP7|E9PKP7_HUMAN;sp|P17480|UBF1_HUMAN;tr|E9PLT2|E9PLT2_HUMAN	2,4579	17171000	3	2	0,09532972	-0,128938039	NaN	20,18302	20,39352	20,2383	20,11553	20,09275	20,22482	20,13759
+	tr|D6R974|D6R974_HUMAN;sp|P09936|UCHL1_HUMAN;tr|D6R956|D6R956_HUMAN;tr|D6RE8	tr|D6R974|D6R974_HUMAN;sp|P09936|UCHL1_HUMAN;tr|D6R956|D6R956_HUMAN;tr|D6RE8	39,807	134180000	13	4	0,09560425	-0,583829403	23,0581	23,21079	23,74755	23,48296	22,69341	23,4025	22,17725	22,89093

	sp|P40227|TCPZ_HUMAN;sp|Q92526|TCPW_HUMAN;tr|J3KRI6|J3KRI6_HUMAN	sp|P40227|TCPZ_HUMAN	320,1	1048700000	53	19	0,09645953	0,205569267	25,93818	25,87352	25,83328	26,01148	25,94829	26,35788	25,97631	26,19625
	tr|Q3BDU5|Q3BDU5_HUMAN;sp|P02545|LMNA_HUMAN;tr|Q5TCI8|Q5TCI8_HUMAN;tr|H0YAB	tr|Q3BDU5|Q3BDU5_HUMAN;sp|P02545|LMNA_HUMAN;tr|Q5TCI8|Q5TCI8_HUMAN	22,935	99286000	24	11	0,09711616	0,282410622	22,48242	22,12436	22,30446	22,63112	22,4877	22,6093	22,92723	22,64777
+	sp|Q709C8|VP13C_HUMAN	sp|Q709C8|VP13C_HUMAN	24,222	59499000	24	15	0,09869647	-1,614850521	21,28771	22,67805	21,48045	23,84163	21,56973	20,53403	21,5833	19,14138
	sp|P07237|PDIA1_HUMAN;tr|H7BZ94|H7BZ94_HUMAN;tr|H0Y3Z3|H0Y3Z3_HUMAN;tr|I3L398|	sp|P07237|PDIA1_HUMAN;tr|H7BZ94|H7BZ94_HUMAN;tr|H0Y3Z3|H0Y3Z3_HUMAN;tr|I3L398|	123,6	265930000	38	14	0,09920975	0,177336216	23,87961	23,88995	24,12258	24,04419	24,05532	24,30382	24,03238	24,25415
+	sp|Q01469|FABP5_HUMAN;tr|I6L8B7|I6L8B7_HUMAN	sp|Q01469|FABP5_HUMAN	47,63	106070000	15	3	0,09927779	-0,675936699	23,26728	23,27668	23,02102	NaN	22,91989	22,90896	21,74263	22,47808
	tr|E7ES33|E7ES33_HUMAN;tr|E7EPK1|E7EPK1_HUMAN;sp|Q16181|SEPT7_HUMAN;tr|G3V1Q4	tr|E7ES33|E7ES33_HUMAN;tr|E7EPK1|E7EPK1_HUMAN;sp|Q16181|SEPT7_HUMAN;tr|G3V1Q4	7,2208	50028000	12	5	0,09982743	-0,317928791	21,53488	21,82695	21,72974	21,67401	21,419	21,27378	21,03812	21,76296
	sp|P45974|UBP5_HUMAN;tr|F5H571|F5H571_HUMAN	sp|P45974|UBP5_HUMAN	28,481	19869000	13	6	0,10069731	-0,481610298	20,45342	20,76733	20,62979	20,56086	19,70315	20,18507	19,82537	20,77137
	sp|P46779|RL28_HUMAN;tr|H0YKD8|H0YKD8_HUMAN;tr|H0YLP6|H0YLP6_HUMAN;tr|H0YMF4	sp|P46779|RL28_HUMAN;tr|H0YKD8|H0YKD8_HUMAN;tr|H0YLP6|H0YLP6_HUMAN;tr|H0YMF4	18,748	194450000	9	4	0,10229955	-0,321056366	24,1043	23,97176	23,74929	23,56523	23,55063	23,41455	23,84029	23,3009
	sp|P11172|UMPS_HUMAN;tr|E9PFD2|E9PFD2_HUMAN	sp|P11172|UMPS_HUMAN	3,2636	23664000	10	3	0,10272557	0,485900402	19,7552	20,71339	20,37029	20,29849	20,7432	21,21721	20,5093	20,61126
	sp|Q9Y295|DRG1_HUMAN	sp|Q9Y295|DRG1_HUMAN	4,4206	14367000	5	2	0,10326112	-0,360818386	20,23841	20,1061	20,10738	20,23351	19,99397	19,48695	19,52081	20,24039
	tr|H7C1U0|H7C1U0_HUMAN;sp|P13798|ACPH_HUMAN;tr|C9JIF9|C9JIF9_HUMAN;tr|C9JLK2|C9	tr|H7C1U0|H7C1U0_HUMAN;sp|P13798|ACPH_HUMAN;tr|C9JIF9|C9JIF9_HUMAN;tr|C9JLK2|C9	5,777	9411700	4	2	0,1037114	-0,172354221	19,44581	19,40707	19,37063	19,55165	19,23804	19,19648	19,14385	19,50736
	sp|P12273|PIP_HUMAN	sp|P12273|PIP_HUMAN	14,554	31158000	8	4	0,10430597	-0,414711952	21,64961	20,97939	20,82943	21,63923	20,87065	20,80554	20,90566	20,85696
	sp|P62701|RS4X_HUMAN;tr|C9JEH7|C9JEH7_HUMAN;sp|P22090|RS4Y1_HUMAN;sp|Q8TD47|F	sp|P62701|RS4X_HUMAN;tr|C9JEH7|C9JEH7_HUMAN;sp|P22090|RS4Y1_HUMAN;sp|Q8TD47|F	56,941	994590000	35	8	0,10509357	-0,376177311	26,10159	26,35301	26,38096	25,87516	25,55514	25,9205	26,19263	25,53774
	tr|B4DXZ6|B4DXZ6_HUMAN;sp|P51114|FXR1_HUMAN;tr|E7EU85|E7EU85_HUMAN;tr|E9PFF5|	tr|B4DXZ6|B4DXZ6_HUMAN;sp|P51114|FXR1_HUMAN;tr|E7EU85|E7EU85_HUMAN;tr|E9PFF5|	75,704	40630000	17	3	0,10655255	0,343827248	21,29995	20,86537	21,43939	20,75197	21,27355	21,45287	21,38527	21,62029
	tr|J3QSB4|J3QSB4_HUMAN;sp|P26373|RL13_HUMAN	tr|J3QSB4|J3QSB4_HUMAN;sp|P26373|RL13_HUMAN	323,31	270640000	7	3	0,10655361	-0,510612011	24,80454	24,3536	24,72728	23,92624	23,82474	23,62232	23,86872	24,45344
	sp|P30520|PURA2_HUMAN	sp|P30520|PURA2_HUMAN	28,394	47076000	17	6	0,10712594	0,44738102	21,53853	21,28884	21,44935	20,86688	21,82236	21,5285	21,37449	22,20777
	sp|Q9UMS4|PRP19_HUMAN;tr|F5GY56|F5GY56_HUMAN;tr|H0YGF3|H0YGF3_HUMAN;tr|F5H2I	sp|Q9UMS4|PRP19_HUMAN	16,806	233790000	20	7	0,10935396	0,272353649	23,37663	23,61897	23,96543	23,60851	23,80754	23,94735	24,1255	23,77856
	tr|E9PCY7|E9PCY7_HUMAN;sp|P31943|HNRH1_HUMAN;tr|G8JLB6|G8JLB6_HUMAN;tr|D6RIU0	tr|E9PCY7|E9PCY7_HUMAN;sp|P31943|HNRH1_HUMAN;tr|G8JLB6|G8JLB6_HUMAN;tr|D6RIU0	276,89	550200000	48	4	0,11098454	0,241907597	24,70525	24,80966	24,97631	25,2787	25,08865	25,19197	25,22494	25,23199
	sp|P10809|CH60_HUMAN;tr|E7EXB4|E7EXB4_HUMAN;tr|E7ESH4|E7ESH4_HUMAN;tr|C9JL25|C	sp|P10809|CH60_HUMAN	323,31	5182700000	125	32	0,11323955	-0,241659164	28,17131	28,45787	28,50154	28,77859	28,14375	28,29321	28,1969	28,30882
	sp|Q8N163|CCAR2_HUMAN;tr|G3V119|G3V119_HUMAN;tr|H0YB24|H0YB24_HUMAN;tr|H0YC	sp|Q8N163|CCAR2_HUMAN;tr|G3V119|G3V119_HUMAN;tr|H0YB24|H0YB24_HUMAN	17,731	51214000	22	8	0,11535343	0,397939682	21,516	21,49634	21,68787	21,0764	21,67077	21,45478	22,01356	22,22926
	tr|C9JBI3|C9JBI3_HUMAN;sp|P78330|SERB_HUMAN	tr|C9JBI3|C9JBI3_HUMAN;sp|P78330|SERB_HUMAN	4,506	14356000	4	3	0,11608391	-0,189425151	20,10457	NaN	20,06815	19,96113	20,02351	19,78094	19,76113	NaN
	sp|Q08J23|NSUN2_HUMAN;tr|A0A140T9Y7|A0A140T9Y7_HUMAN	sp|Q08J23|NSUN2_HUMAN	11,019	49579000	15	5	0,11610128	0,267057896	21,25119	21,63378	21,40401	21,58192	21,44779	21,9333	21,64342	21,91463
	sp|P11586|C1TC_HUMAN;tr|F5H2F4|F5H2F4_HUMAN;tr|V9GYY3|V9GYY3_HUMAN	sp|P11586|C1TC_HUMAN;tr|F5H2F4|F5H2F4_HUMAN	93,415	417540000	76	23	0,11618624	0,187161922	24,54251	24,78362	24,56721	24,80563	24,92034	24,79313	24,69588	25,03825
	sp|Q15185|TEBP_HUMAN;tr|A0A087WYT3|A0A087WYT3_HUMAN	sp|Q15185|TEBP_HUMAN;tr|A0A087WYT3|A0A087WYT3_HUMAN	18,443	48910000	13	3	0,11656907	-0,463601589	21,66375	21,97456	22,06046	22,28881	21,38158	22,1169	21,08245	21,55225
	sp|P04843|RPN1_HUMAN;tr|B7Z4L4|B7Z4L4_HUMAN;tr|F8WF32|F8WF32_HUMAN	sp|P04843|RPN1_HUMAN;tr|B7Z4L4|B7Z4L4_HUMAN	96,578	243140000	47	12	0,11977585	-0,18811512	23,96288	24,00573	24,19746	24,08966	23,89901	23,86656	24,08837	23,64933
	tr|C9J8B8|C9J8B8_HUMAN;sp|Q969S8|HDA10_HUMAN	tr|C9J8B8|C9J8B8_HUMAN;sp|Q969S8|HDA10_HUMAN	1,6331	4228600	6	1	0,12041853	-0,549518744	NaN	18,88804	18,65179	18,3169	18,33447	17,42955	18,3699	18,14365
	sp|P13010|XRCC5_HUMAN;tr|C9JZ81|C9JZ81_HUMAN;tr|H7C0H9|H7C0H9_HUMAN	sp|P13010|XRCC5_HUMAN	168,84	295950000	50	17	0,12253405	-0,244042397	24,50936	24,20053	24,64581	24,36853	23,90159	24,24235	24,32574	24,27838
	sp|O15067|PUR4_HUMAN;tr|H0YGH1|H0YGH1_HUMAN;tr|J3KTQ5|J3KTQ5_HUMAN;tr|J3KT98	sp|O15067|PUR4_HUMAN	38,998	88998000	22	9	0,12256413	0,326598644	22,44096	22,36933	22,2247	22,22635	22,64625	22,26396	22,55569	23,10183
+	sp|P01876|IGHA1_HUMAN	sp|P01876|IGHA1_HUMAN	4,0982	27526000	6	2	0,12271062	-1,20516332	22,65513	NaN	20,60991	21,12452	20,28704	20,13646	20,35057	NaN
	sp|P47897|SYQ_HUMAN;tr|A0A0U1RQT0|A0A0U1RQT0_HUMAN;tr|A0A0U1RQM8|A0A0U1RQI	sp|P47897|SYQ_HUMAN;tr|A0A0U1RQT0|A0A0U1RQT0_HUMAN;tr|A0A0U1RQM8|A0A0U1RQI	36,361	138240000	26	14	0,12595124	-0,143039227	23,37518	23,19768	23,4467	23,19301	23,11009	23,06154	23,28673	23,18204
	tr|A0A087WYU1|A0A087WYU1_HUMAN;sp|Q9Y5X1|SNX9_HUMAN	tr|A0A087WYU1|A0A087WYU1_HUMAN;sp|Q9Y5X1|SNX9_HUMAN	8,4674	10741000	6	3	0,12632349	0,167490482	19,62412	19,35954	19,4616	19,52338	19,75589	19,50959	19,71347	NaN
	sp|Q8N766|EMC1_HUMAN;tr|Q5TG59|Q5TG59_HUMAN	sp|Q8N766|EMC1_HUMAN	17,418	39526000	15	11	0,12762022	0,32606554	20,90492	20,91933	21,00093	20,82306	20,96077	21,0466	21,76332	21,1818
	sp|P49257|LMAN1_HUMAN	sp|P49257|LMAN1_HUMAN	7,6047	57603000	6	3	0,12812458	0,426394939	21,55581	21,60598	21,74833	21,48493	21,67263	22,30003	22,54399	21,58399
	sp|P16989|YBOX3_HUMAN;tr|A0A0D9SEI8|A0A0D9SEI8_HUMAN	sp|P16989|YBOX3_HUMAN	52,366	31941000	8	3	0,13050955	-0,298365593	20,97435	20,83368	21,3849	21,22317	20,71482	20,63741	21,15351	20,71691
	sp|P46977|STT3A_HUMAN;tr|A0A0C4DH80|A0A0C4DH80_HUMAN;tr|E9PN73|E9PN73_HUMAN	sp|P46977|STT3A_HUMAN	3,4546	29146000	4	3	0,13122143	-0,563631535	20,9693	21,23806	21,98621	21,06848	NaN	20,68765	21,0959	20,47209
	sp|P55884|EIF3B_HUMAN;tr|C9JQN7|C9JQN7_HUMAN;tr|C9JZG1|C9JZG1_HUMAN	sp|P55884|EIF3B_HUMAN	29,746	147490000	21	10	0,131658	-0,246012688	23,48333	23,3948	23,51977	23,20271	23,0443	22,98536	23,07097	23,51593
	sp|O60701|UGDH_HUMAN;tr|D6RHF4|D6RHF4_HUMAN;tr|E9PBD2|E9PBD2_HUMAN;tr|E7ER9	sp|O60701|UGDH_HUMAN	3,8425	17435000	10	3	0,13325023	-0,510213852	20,41473	20,56123	20,27435	20,76871	20,35402	19,79992	19,31134	20,51287
	sp|Q9P0L0|VAPA_HUMAN;tr|J3QKM9|J3QKM9_HUMAN	sp|Q9P0L0|VAPA_HUMAN	14,683	60335000	12	3	0,1335238	0,471261501	21,20467	21,95787	22,0483	21,91605	22,09152	22,10776	22,55967	NaN
	tr|G3V1B3|G3V1B3_HUMAN;tr|M0R181|M0R181_HUMAN;sp|P46778|RL21_HUMAN	tr|G3V1B3|G3V1B3_HUMAN;tr|M0R181|M0R181_HUMAN;sp|P46778|RL21_HUMAN	3,956	193080000	6	3	0,13466814	-0,400205612	23,98833	23,79729	24,37465	23,48726	NaN	23,50543	23,61054	23,41905
	sp|P04406|G3P_HUMAN;tr|E7EUT5|E7EUT5_HUMAN;sp|O14556|G3PT_HUMAN	sp|P04406|G3P_HUMAN;tr|E7EUT5|E7EUT5_HUMAN	323,31	1583200000	43	9	0,13521725	-0,378370285	26,65676	26,64636	27,0203	27,15364	26,4447	26,65349	26,01844	26,84697
	tr|A0A0A0MRA5|A0A0A0MRA5_HUMAN;tr|B7Z4B8|B7Z4B8_HUMAN;sp|Q9BUJ2|HNRL1_HUM/	tr|A0A0A0MRA5|A0A0A0MRA5_HUMAN;tr|B7Z4B8|B7Z4B8_HUMAN;sp|Q9BUJ2|HNRL1_HUM/	11,932	38540000	12	5	0,13540792	-0,212114334	21,44035	21,3048	21,64917	21,38427	21,38021	21,00867	21,12622	21,41504
	sp|Q15046|SYK_HUMAN;tr|J3KRL2|J3KRL2_HUMAN;tr|H3BPV7|H3BPV7_HUMAN	sp|Q15046|SYK_HUMAN	7,4094	22847000	12	3	0,13570844	-0,296369076	20,58838	20,40312	20,86771	20,74911	20,1545	20,36954	20,74517	20,15363
	sp|Q13561|DCTN2_HUMAN;tr|H0YI98|H0YI98_HUMAN;tr|F8W1I6|F8W1I6_HUMAN;tr|F8VW1	sp|Q13561|DCTN2_HUMAN;tr|H0YI98|H0YI98_HUMAN;tr|F8W1I6|F8W1I6_HUMAN;tr|F8VW1	7,2322	35801000	13	6	0,13758418	-0,152151108	21,15728	21,20371	21,34066	21,26264	20,87673	21,2191	21,05729	21,20258
	sp|P51858|HDGF_HUMAN	sp|P51858|HDGF_HUMAN	15,619	80460000	14	4	0,13960727	-0,420668125	22,56086	22,54328	22,30123	22,75654	21,90936	22,40026	21,58325	22,58637
	sp|P18124|RL7_HUMAN;tr|A8MUD9|A8MUD9_HUMAN;tr|C9JIJ5|C9JIJ5_HUMAN;tr|C9JZ88|C9	sp|P18124|RL7_HUMAN;tr|A8MUD9|A8MUD9_HUMAN	52,349	584430000	42	10	0,1411243	-0,329071522	25,17043	25,50054	25,60908	25,14767	24,91862	25,13073	25,39663	24,66546
	sp|P10599|THIO_HUMAN	sp|P10599|THIO_HUMAN	38,109	233240000	13	5	0,14169246	0,486328125	23,98616	23,92071	23,20939	23,47209	24,43619	24,2467	23,48444	24,36633
	sp|P05109|S10A8_HUMAN	sp|P05109|S10A8_HUMAN	7,3461	66786000	9	3	0,14510511	-0,548692226	23,11824	22,65176	21,77278	22,62536	22,23386	22,26436	21,53269	21,94246
	tr|J3QLR8|J3QLR8_HUMAN;sp|Q9Y3D9|RT23_HUMAN	tr|J3QLR8|J3QLR8_HUMAN;sp|Q9Y3D9|RT23_HUMAN	5,7921	26361000	7	4	0,14645904	-0,15183115	20,82376	20,81026	21,12408	20,96684	20,76247	20,81855	NaN	20,75719
	sp|P81605|DCD_HUMAN	sp|P81605|DCD_HUMAN	6,5784	148150000	11	3	0,14690426	-0,391135693	23,75929	23,46139	23,38034	23,67206	23,75348	22,7633	23,25609	22,93566
	sp|P63151|2ABA_HUMAN;tr|E5RFR9|E5RFR9_HUMAN;tr|E5RIY1|E5RIY1_HUMAN;sp|Q00005|2	sp|P63151|2ABA_HUMAN	11,782	59601000	20	6	0,14746177	0,180468559	21,53588	21,76927	21,81769	21,86876	21,98211	21,89667	21,72713	22,10757
	sp|P33176|KINH_HUMAN;tr|A0A0G2JMZ6|A0A0G2JMZ6_HUMAN;tr|J3KNA1|J3KNA1_HUMAN;	sp|P33176|KINH_HUMAN	26,828	67643000	38	12	0,14750611	-0,318947315	22,40026	22,35305	22,36076	22,27674	22,03842	21,53564	22,07858	22,46236
	tr|E9PLK3|E9PLK3_HUMAN;sp|P55786|PSA_HUMAN;sp|A6NEC2|PSAL_HUMAN;tr|H0YCQ5|H0	tr|E9PLK3|E9PLK3_HUMAN;sp|P55786|PSA_HUMAN	18,983	121930000	25	11	0,14955002	-0,18057251	22,82409	23,08413	22,87793	23,12605	22,86221	22,69211	22,64414	22,99144
	sp|Q13243|SRSF5_HUMAN;tr|G3V5K8|G3V5K8_HUMAN;tr|B4DJK0|B4DJK0_HUMAN;tr|B4DUA	sp|Q13243|SRSF5_HUMAN;tr|G3V5K8|G3V5K8_HUMAN;tr|B4DJK0|B4DJK0_HUMAN;tr|B4DUA	12,33	23300000	15	3	0,15406353	-0,407518387	19,9073	20,51528	20,54952	20,13884	19,51032	20,06697	20,32663	19,57694
	sp|P34932|HSP74_HUMAN;tr|A0A087WYC1|A0A087WYC1_HUMAN;tr|A0A087WTS8|A0A087W	sp|P34932|HSP74_HUMAN;tr|A0A087WYC1|A0A087WYC1_HUMAN;tr|A0A087WTS8|A0A087W	115,98	224170000	40	14	0,15669938	0,12815237	23,829	23,91369	23,71471	23,75746	23,80361	23,83306	24,03036	24,06044
	tr|F8W1R7|F8W1R7_HUMAN;sp|P60660|MYL6_HUMAN;tr|J3KND3|J3KND3_HUMAN;tr|G8JLA2	tr|F8W1R7|F8W1R7_HUMAN;sp|P60660|MYL6_HUMAN;tr|J3KND3|J3KND3_HUMAN;tr|G8JLA2	6,9853	55008000	10	4	0,15719874	-0,195950985	21,96782	21,88807	22,01086	22,1549	22,08832	21,85068	21,5855	21,71335
	sp|Q9UBE0|SAE1_HUMAN;tr|M0QZS6|M0QZS6_HUMAN;tr|M0QX65|M0QX65_HUMAN;tr|B3K	sp|Q9UBE0|SAE1_HUMAN;tr|M0QZS6|M0QZS6_HUMAN;tr|M0QX65|M0QX65_HUMAN;tr|B3K	71,714	31240000	13	7	0,15811278	-0,490921021	21,02378	21,19701	21,47788	21,87429	20,9132	21,20508	20,21703	21,27395
	sp|Q9UBB6|NCDN_HUMAN;tr|C9J5H8|C9J5H8_HUMAN	sp|Q9UBB6|NCDN_HUMAN;tr|C9J5H8|C9J5H8_HUMAN	3,9546	8359200	8	2	0,15903865	0,337044398	18,9922	NaN	18,94524	19,27542	NaN	19,10319	19,44845	19,67235
	sp|P36404|ARL2_HUMAN	sp|P36404|ARL2_HUMAN	3,0058	14781000	5	2	0,15912224	0,896163305	NaN	19,35281	19,26091	19,30672	21,32405	20,57404	19,50999	19,40383
	sp|O15355|PPM1G_HUMAN	sp|O15355|PPM1G_HUMAN	13,976	37478000	13	5	0,15918311	-0,397400379	21,6189	21,25523	21,813	21,53022	21,09539	21,13515	20,66709	21,73012
	sp|P34897|GLYM_HUMAN;tr|H0YIZ0|H0YIZ0_HUMAN;tr|G3V5L0|G3V5L0_HUMAN;tr|G3V2Y4|	sp|P34897|GLYM_HUMAN;tr|H0YIZ0|H0YIZ0_HUMAN	54,759	334280000	38	13	0,15927192	-0,271460533	24,59206	24,3573	24,69678	24,82026	24,30932	24,30375	24,05142	24,71607
	sp|Q14204|DYHC1_HUMAN;tr|H0YJ21|H0YJ21_HUMAN	sp|Q14204|DYHC1_HUMAN	159,39	464100000	110	58	0,16078751	0,243444443	24,68528	24,86858	24,68233	24,54828	24,57323	25,01665	25,23257	24,9358
	sp|P26196|DDX6_HUMAN;tr|Q8IV96|Q8IV96_HUMAN	sp|P26196|DDX6_HUMAN	11,828	43629000	13	6	0,16197725	-0,227039814	21,27855	21,60937	21,8273	21,7195	21,52363	21,44486	21,15845	21,39963
	sp|P24390|ERD21_HUMAN;tr|M0R1Y2|M0R1Y2_HUMAN	sp|P24390|ERD21_HUMAN;tr|M0R1Y2|M0R1Y2_HUMAN	43,566	67132000	20	2	0,16295209	0,585076332	22,0124	20,96212	22,2287	21,27923	22,50582	22,35081	22,39616	21,56997
	sp|Q9NVP1|DDX18_HUMAN;tr|H7C452|H7C452_HUMAN	sp|Q9NVP1|DDX18_HUMAN;tr|H7C452|H7C452_HUMAN	5,2104	10479000	6	3	0,16438328	0,663415909	19,13016	18,78625	19,19302	18,06844	18,67731	20,05179	19,95617	19,14627
	sp|Q13409|DC1I2_HUMAN;tr|E9PGG1|E9PGG1_HUMAN;tr|E7EUM4|E7EUM4_HUMAN;tr|E7ER	sp|Q13409|DC1I2_HUMAN;tr|E9PGG1|E9PGG1_HUMAN;tr|E7EUM4|E7EUM4_HUMAN;tr|E7ER	27,671	70679000	9	3	0,16439875	-0,563911438	22,224	22,22205	22,45249	23,20653	22,26316	21,87867	21,24197	22,46563
	sp|P00492|HPRT_HUMAN	sp|P00492|HPRT_HUMAN	14,916	90418000	22	6	0,1679295	-0,183864117	22,24284	22,46037	22,66573	22,55985	22,19376	22,51944	22,21877	22,26135
	sp|Q92616|GCN1_HUMAN	sp|Q92616|GCN1_HUMAN	200,78	257500000	81	36	0,16868516	0,369713306	23,40341	23,97544	23,51509	23,83547	23,51328	24,22949	24,4168	24,04868
	sp|P33991|MCM4_HUMAN;tr|E5RG31|E5RG31_HUMAN;tr|E5RFJ8|E5RFJ8_HUMAN	sp|P33991|MCM4_HUMAN;tr|E5RG31|E5RG31_HUMAN	22,808	133630000	28	13	0,16966084	0,169204235	23,01141	22,90533	22,9985	23,15417	22,99016	23,05752	23,3243	23,37425
	tr|K7EJE8|K7EJE8_HUMAN;tr|K7EKE6|K7EKE6_HUMAN;sp|P36776|LONM_HUMAN;tr|K7ER27|I	tr|K7EJE8|K7EJE8_HUMAN;tr|K7EKE6|K7EKE6_HUMAN;sp|P36776|LONM_HUMAN	11,265	35084000	22	6	0,17093158	-0,349708557	21,28433	21,46339	21,33222	21,60616	21,01025	20,70868	20,88517	21,68316
	sp|O15160|RPAC1_HUMAN;tr|E7EQB9|E7EQB9_HUMAN;tr|D6RDJ3|D6RDJ3_HUMAN;tr|H0Y72	sp|O15160|RPAC1_HUMAN;tr|E7EQB9|E7EQB9_HUMAN	7,9664	15572000	8	3	0,17159407	0,257406712	NaN	19,93229	20,01808	19,7957	20,20693	19,88011	20,1047	20,49931
	sp|P24534|EF1B_HUMAN;tr|F2Z2G2|F2Z2G2_HUMAN;tr|C9JZW3|C9JZW3_HUMAN;tr|F8WF65	sp|P24534|EF1B_HUMAN;tr|F2Z2G2|F2Z2G2_HUMAN;tr|C9JZW3|C9JZW3_HUMAN	121,45	156350000	8	3	0,17190672	-0,257378101	23,13016	23,3868	23,53172	23,72067	23,0353	23,3507	22,95955	23,39428
	sp|Q00059|TFAM_HUMAN;tr|H7BYN3|H7BYN3_HUMAN	sp|Q00059|TFAM_HUMAN;tr|H7BYN3|H7BYN3_HUMAN	3,4353	72939000	7	3	0,17592566	-0,336787224	21,87804	22,47586	22,51058	22,69271	22,02296	22,04126	22,38987	21,75593
	tr|A0A0B4J1W3|A0A0B4J1W3_HUMAN;sp|Q9BXJ9|NAA15_HUMAN;sp|Q6N069|NAA16_HUMA	tr|A0A0B4J1W3|A0A0B4J1W3_HUMAN;sp|Q9BXJ9|NAA15_HUMAN	12,039	23147000	8	5	0,17775511	-0,218058427	20,57228	20,53136	20,86718	20,58683	NaN	20,43288	20,19785	20,63334
	sp|P78527|PRKDC_HUMAN	sp|P78527|PRKDC_HUMAN	273,63	572230000	145	55	0,1787566	0,201046944	24,98011	25,15661	24,8739	25,04181	24,8624	25,29204	25,35867	25,3435
	sp|P61106|RAB14_HUMAN;tr|X6RFL8|X6RFL8_HUMAN	sp|P61106|RAB14_HUMAN;tr|X6RFL8|X6RFL8_HUMAN	93,583	249500000	27	8	0,17893438	-0,531393528	23,406	24,52898	24,31693	24,27987	23,61728	24,28221	23,34516	23,16156
	sp|O43395|PRPF3_HUMAN	sp|O43395|PRPF3_HUMAN	2,7448	8852900	3	2	0,18304553	-0,226391315	19,48911	19,27912	19,32133	19,33756	19,02948	18,79592	19,22651	19,46965
	sp|Q14008|CKAP5_HUMAN;tr|H0YDX5|H0YDX5_HUMAN	sp|Q14008|CKAP5_HUMAN	55,078	31429000	19	9	0,18405575	0,19549036	21,04733	20,89267	20,90139	20,73355	20,74985	21,21537	21,18101	21,21068
	sp|P26599|PTBP1_HUMAN;tr|A0A0U1RRM4|A0A0U1RRM4_HUMAN;tr|A6NLN1|A6NLN1_HUM	sp|P26599|PTBP1_HUMAN;tr|A0A0U1RRM4|A0A0U1RRM4_HUMAN;tr|A6NLN1|A6NLN1_HUM	148,16	424820000	51	13	0,18493656	-0,298606873	25,01979	24,6371	24,91534	24,92089	24,07022	24,59309	24,71046	24,92493
	tr|F8WAU4|F8WAU4_HUMAN;tr|C9IZ01|C9IZ01_HUMAN;sp|Q96RP9|EFGM_HUMAN	tr|F8WAU4|F8WAU4_HUMAN;tr|C9IZ01|C9IZ01_HUMAN;sp|Q96RP9|EFGM_HUMAN	13,996	41660000	20	7	0,18861781	0,169564247	21,41875	21,34646	21,34061	21,53825	21,62123	21,43858	21,4026	21,85992
	sp|Q8NBJ5|GT251_HUMAN;tr|M0QYH0|M0QYH0_HUMAN;tr|M0QX72|M0QX72_HUMAN	sp|Q8NBJ5|GT251_HUMAN	8,4581	32379000	11	5	0,19254319	-0,277807236	20,8155	21,20848	21,32899	21,66384	21,09642	21,01311	21,02823	20,76782
	tr|A2A2D0|A2A2D0_HUMAN;sp|P16949|STMN1_HUMAN;tr|E5RGX5|E5RGX5_HUMAN;sp|Q93(	tr|A2A2D0|A2A2D0_HUMAN;sp|P16949|STMN1_HUMAN;tr|E5RGX5|E5RGX5_HUMAN;sp|Q93(	5,1669	53851000	3	2	0,19692365	-0,404438019	22,12918	21,89138	21,92027	22,09861	21,81355	22,19214	21,51167	20,90434
	sp|Q99613|EIF3C_HUMAN;sp|B5ME19|EIFCL_HUMAN;tr|H3BPE3|H3BPE3_HUMAN;tr|H3BPE4|	sp|Q99613|EIF3C_HUMAN;sp|B5ME19|EIFCL_HUMAN	17,126	145800000	18	8	0,19909856	-0,31782198	23,68453	23,36132	23,30647	22,8303	23,06911	22,64092	23,27071	22,93059
	sp|P26639|SYTC_HUMAN;tr|D6RBR8|D6RBR8_HUMAN;tr|D6RCA5|D6RCA5_HUMAN;tr|D6R9F8	sp|P26639|SYTC_HUMAN	28,354	131410000	29	13	0,20135263	-0,208114624	23,3362	23,22142	23,22005	23,29011	23,12907	23,03539	22,6917	23,37915
	sp|P39019|RS19_HUMAN;tr|A0A075B6E2|A0A075B6E2_HUMAN;tr|M0R2L9|M0R2L9_HUMAN;	sp|P39019|RS19_HUMAN;tr|A0A075B6E2|A0A075B6E2_HUMAN	24,3	243710000	19	6	0,2031218	-0,342044353	24,6284	23,67066	24,09869	24,30855	24,10406	23,91743	23,48087	23,83576
	tr|E9PD53|E9PD53_HUMAN;sp|Q9NTJ3|SMC4_HUMAN;tr|C9JR83|C9JR83_HUMAN	tr|E9PD53|E9PD53_HUMAN;sp|Q9NTJ3|SMC4_HUMAN;tr|C9JR83|C9JR83_HUMAN	33,535	11944000	12	4	0,20403778	0,268901984	19,42173	19,7376	NaN	19,39427	19,77077	19,42577	19,88527	20,06526
	tr|E9PMD8|E9PMD8_HUMAN;tr|E9PKD5|E9PKD5_HUMAN;tr|E9PM69|E9PM69_HUMAN;tr|R4(	tr|E9PMD8|E9PMD8_HUMAN;tr|E9PKD5|E9PKD5_HUMAN;tr|E9PM69|E9PM69_HUMAN;tr|R4(	9,1517	40658000	12	5	0,20709165	0,454024315	21,19063	20,52965	20,84023	20,99106	21,21555	21,34706	22,10287	20,70219
	sp|P53396|ACLY_HUMAN;tr|K7ESG8|K7ESG8_HUMAN;tr|K7EIE7|K7EIE7_HUMAN	sp|P53396|ACLY_HUMAN	87,441	230470000	47	12	0,212533	-0,225792885	24,22817	24,06587	24,06266	23,9581	23,49692	24,02183	23,71796	24,17493
	tr|H0Y4R1|H0Y4R1_HUMAN;sp|P12268|IMDH2_HUMAN;tr|E7ETK5|E7ETK5_HUMAN	tr|H0Y4R1|H0Y4R1_HUMAN;sp|P12268|IMDH2_HUMAN;tr|E7ETK5|E7ETK5_HUMAN	93,24	202260000	38	10	0,21384732	-0,146356106	23,85043	23,73913	23,99412	23,74324	23,65895	23,67508	23,49351	23,91397
	sp|P55036|PSMD4_HUMAN;tr|Q5VWC4|Q5VWC4_HUMAN;tr|A6PVX3|A6PVX3_HUMAN;tr|H0	sp|P55036|PSMD4_HUMAN;tr|Q5VWC4|Q5VWC4_HUMAN;tr|A6PVX3|A6PVX3_HUMAN	9,9217	37102000	4	4	0,21409296	-0,109734376	21,39305	NaN	21,51123	21,39021	21,19394	21,38042	21,45453	21,25816

<table>
<tr><td>sp|O00116|ADAS_HUMAN;tr|B8ZZ81|B8ZZ81_HUMAN</td><td>sp|O00116|ADAS_HUMAN</td><td>9,4745</td><td>15289000</td><td>9</td><td>6</td><td>0,21738728</td><td>-0,168743134</td><td>NaN</td><td>20,21703</td><td>20,32531</td><td>20,24621</td><td>NaN</td><td>19,87263</td><td>20,20383</td><td>20,20586</td></tr>
<tr><td>tr|H0YMZ1|H0YMZ1_HUMAN;tr|H0YL69|H0YL69_HUMAN;sp|P25789|PSA4_HUMAN;tr|H0YMI</td><td>tr|H0YMZ1|H0YMZ1_HUMAN;tr|H0YL69|H0YL69_HUMAN;sp|P25789|PSA4_HUMAN;tr|H0YMI</td><td>8,5554</td><td>120950000</td><td>12</td><td>5</td><td>0,2180686</td><td>-0,31953764</td><td>23,25123</td><td>22,80532</td><td>23,52241</td><td>22,98251</td><td>22,72096</td><td>22,90773</td><td>22,41841</td><td>23,23621</td></tr>
<tr><td>sp|Q9NZ01|TECR_HUMAN;tr|M0R3C3|M0R3C3_HUMAN;tr|M0QXM3|M0QXM3_HUMAN</td><td>sp|Q9NZ01|TECR_HUMAN</td><td>13,032</td><td>43106000</td><td>9</td><td>5</td><td>0,21934331</td><td>-0,22462368</td><td>21,50983</td><td>21,34435</td><td>21,77668</td><td>21,38142</td><td>21,11997</td><td>21,35014</td><td>21,61598</td><td>21,02769</td></tr>
<tr><td>sp|P49189|AL9A1_HUMAN</td><td>sp|P49189|AL9A1_HUMAN</td><td>8,3825</td><td>40894000</td><td>10</td><td>4</td><td>0,21981056</td><td>-0,243035793</td><td>21,32147</td><td>21,40047</td><td>21,75516</td><td>21,56425</td><td>21,36212</td><td>21,62992</td><td>20,93352</td><td>21,14364</td></tr>
<tr><td>sp|Q58FF8|H90B2_HUMAN</td><td>sp|Q58FF8|H90B2_HUMAN</td><td>2,5584</td><td>93121000</td><td>3</td><td>1</td><td>0,22147285</td><td>-0,520754337</td><td>22,79741</td><td>22,72833</td><td>22,90718</td><td>22,82834</td><td>21,19093</td><td>22,90472</td><td>22,44083</td><td>22,64176</td></tr>
<tr><td>tr|H0YLA2|H0YLA2_HUMAN;sp|P37108|SRP14_HUMAN</td><td>tr|H0YLA2|H0YLA2_HUMAN;sp|P37108|SRP14_HUMAN</td><td>9,1154</td><td>47662000</td><td>7</td><td>4</td><td>0,22215115</td><td>-0,452655315</td><td>22,04263</td><td>21,43359</td><td>21,40966</td><td>22,60139</td><td>21,48944</td><td>21,79685</td><td>20,95992</td><td>21,43043</td></tr>
<tr><td>sp|P13804|ETFA_HUMAN;tr|H0YK49|H0YK49_HUMAN;tr|H0YLU7|H0YLU7_HUMAN;tr|H0YL12|</td><td>sp|P13804|ETFA_HUMAN;tr|H0YK49|H0YK49_HUMAN;tr|H0YLU7|H0YLU7_HUMAN;tr|H0YL12|</td><td>70,583</td><td>65227000</td><td>14</td><td>5</td><td>0,22491495</td><td>-0,267136574</td><td>22,23359</td><td>22,33976</td><td>22,11613</td><td>22,58417</td><td>21,89126</td><td>22,3783</td><td>21,6497</td><td>22,28585</td></tr>
<tr><td>sp|Q9Y6M1|IF2B2_HUMAN;tr|F8W930|F8W930_HUMAN</td><td>sp|Q9Y6M1|IF2B2_HUMAN;tr|F8W930|F8W930_HUMAN</td><td>24,342</td><td>25767000</td><td>12</td><td>6</td><td>0,22592891</td><td>0,238020579</td><td>20,66501</td><td>NaN</td><td>20,5696</td><td>20,53877</td><td>20,49473</td><td>20,84529</td><td>21,19244</td><td>20,78413</td></tr>
<tr><td>tr|B7Z9I1|B7Z9I1_HUMAN;sp|P11310|ACADM_HUMAN;tr|Q5T4U5|Q5T4U5_HUMAN;tr|H0YDT</td><td>tr|B7Z9I1|B7Z9I1_HUMAN;sp|P11310|ACADM_HUMAN;tr|Q5T4U5|Q5T4U5_HUMAN</td><td>19,107</td><td>37091000</td><td>20</td><td>5</td><td>0,2274598</td><td>0,139296055</td><td>21,24284</td><td>21,08466</td><td>21,34846</td><td>21,01182</td><td>21,40499</td><td>21,19743</td><td>21,18441</td><td>21,45814</td></tr>
<tr><td>sp|Q14152|EIF3A_HUMAN</td><td>sp|Q14152|EIF3A_HUMAN</td><td>46,584</td><td>59342000</td><td>19</td><td>9</td><td>0,22895053</td><td>-0,13958025</td><td>22,27373</td><td>21,9289</td><td>22,26196</td><td>22,10322</td><td>21,94321</td><td>21,86291</td><td>22,03463</td><td>22,16873</td></tr>
<tr><td>tr|F8W0J6|F8W0J6_HUMAN;tr|F5H4R6|F5H4R6_HUMAN;tr|H0YIV4|H0YIV4_HUMAN;sp|P5520</td><td>tr|F8W0J6|F8W0J6_HUMAN;tr|F5H4R6|F5H4R6_HUMAN;tr|H0YIV4|H0YIV4_HUMAN;sp|P5520</td><td>94,01</td><td>128030000</td><td>22</td><td>7</td><td>0,22919886</td><td>-0,099397659</td><td>22,95846</td><td>23,13002</td><td>23,15225</td><td>23,15019</td><td>23,06456</td><td>23,03825</td><td>22,82611</td><td>23,06441</td></tr>
<tr><td>sp|P25398|RS12_HUMAN</td><td>sp|P25398|RS12_HUMAN</td><td>106,42</td><td>270470000</td><td>23</td><td>4</td><td>0,2295568</td><td>0,28188467</td><td>23,8146</td><td>23,91169</td><td>23,65163</td><td>24,2717</td><td>24,63445</td><td>24,15677</td><td>23,83451</td><td>24,15143</td></tr>
<tr><td>sp|P01040|CYTA_HUMAN;tr|C9J0E4|C9J0E4_HUMAN</td><td>sp|P01040|CYTA_HUMAN;tr|C9J0E4|C9J0E4_HUMAN</td><td>4,8723</td><td>11773000</td><td>5</td><td>3</td><td>0,23050966</td><td>-0,29877758</td><td>19,77001</td><td>19,97029</td><td>20,02648</td><td>19,51651</td><td>19,99466</td><td>19,09962</td><td>19,35767</td><td>19,63623</td></tr>
<tr><td>sp|Q9HD45|TM9S3_HUMAN;tr|Q5TB53|Q5TB53_HUMAN</td><td>sp|Q9HD45|TM9S3_HUMAN;tr|Q5TB53|Q5TB53_HUMAN</td><td>4,4946</td><td>29787000</td><td>8</td><td>3</td><td>0,23119854</td><td>-0,640635967</td><td>20,85855</td><td>20,80947</td><td>21,5048</td><td>21,29961</td><td>19,22055</td><td>20,68517</td><td>21,36057</td><td>20,64359</td></tr>
<tr><td>sp|P27694|RFA1_HUMAN;tr|I3L4R8|I3L4R8_HUMAN;tr|I3L524|I3L524_HUMAN;tr|I3L2M5|I3L2</td><td>sp|P27694|RFA1_HUMAN</td><td>32,133</td><td>73953000</td><td>24</td><td>10</td><td>0,23856193</td><td>-0,299870491</td><td>22,42772</td><td>22,157</td><td>22,28836</td><td>22,35641</td><td>21,45608</td><td>21,94935</td><td>22,53258</td><td>22,092</td></tr>
<tr><td>sp|Q15233|NONO_HUMAN;tr|H7C367|H7C367_HUMAN;tr|C9IZL7|C9IZL7_HUMAN;tr|C9JYS8|</td><td>sp|Q15233|NONO_HUMAN;tr|H7C367|H7C367_HUMAN</td><td>173,2</td><td>444450000</td><td>27</td><td>12</td><td>0,23932759</td><td>-0,232980728</td><td>25,02445</td><td>24,7599</td><td>24,99391</td><td>25,24329</td><td>24,51683</td><td>24,69216</td><td>24,67928</td><td>25,20135</td></tr>
<tr><td>sp|P51572|BAP31_HUMAN;tr|C9JSP1|C9JSP1_HUMAN;tr|C9JQ75|C9JQ75_HUMAN;tr|C9J0M4|</td><td>sp|P51572|BAP31_HUMAN;tr|C9JSP1|C9JSP1_HUMAN;tr|C9JQ75|C9JQ75_HUMAN;tr|C9J0M4|</td><td>8,2766</td><td>237880000</td><td>5</td><td>5</td><td>0,23975269</td><td>0,227694035</td><td>23,82347</td><td>23,61133</td><td>23,64361</td><td>23,71314</td><td>23,81509</td><td>23,9478</td><td>24,37119</td><td>23,56825</td></tr>
<tr><td>sp|Q9BTV4|TMM43_HUMAN</td><td>sp|Q9BTV4|TMM43_HUMAN</td><td>2,2229</td><td>13354000</td><td>5</td><td>2</td><td>0,24059599</td><td>0,435955207</td><td>19,85792</td><td>19,89318</td><td>19,88526</td><td>18,90177</td><td>19,97323</td><td>19,81209</td><td>20,42614</td><td>NaN</td></tr>
<tr><td>sp|Q9NP97|DLRB1_HUMAN;tr|B1AKR6|B1AKR6_HUMAN;sp|Q8TF09|DLRB2_HUMAN;tr|H3BQI</td><td>sp|Q9NP97|DLRB1_HUMAN;tr|B1AKR6|B1AKR6_HUMAN;sp|Q8TF09|DLRB2_HUMAN;tr|H3BQI</td><td>3,4589</td><td>7631000</td><td>8</td><td>2</td><td>0,2455295</td><td>-0,268414179</td><td>18,96781</td><td>NaN</td><td>19,28834</td><td>19,12813</td><td>18,9221</td><td>19,12563</td><td>18,53131</td><td>NaN</td></tr>
<tr><td>sp|O14980|XPO1_HUMAN;tr|C9JF49|C9JF49_HUMAN;tr|C9IYM2|C9IYM2_HUMAN;tr|F8WF71|</td><td>sp|O14980|XPO1_HUMAN</td><td>98,799</td><td>136010000</td><td>18</td><td>10</td><td>0,24598663</td><td>-0,071960449</td><td>22,99108</td><td>23,09779</td><td>23,02335</td><td>23,10266</td><td>22,90218</td><td>22,9581</td><td>22,94339</td><td>23,12337</td></tr>
<tr><td>sp|Q15637|SF01_HUMAN;tr|H7C561|H7C561_HUMAN</td><td>sp|Q15637|SF01_HUMAN</td><td>31,401</td><td>43695000</td><td>14</td><td>3</td><td>0,24768116</td><td>-0,392627716</td><td>21,67349</td><td>21,50606</td><td>21,55754</td><td>21,79404</td><td>21,41916</td><td>21,82268</td><td>21,31639</td><td>20,40239</td></tr>
<tr><td>sp|P61254|RL26_HUMAN;tr|J3KTJ8|J3KTJ8_HUMAN;tr|J3QRI7|J3QRI7_HUMAN;tr|J3QQQ9|J3C</td><td>sp|P61254|RL26_HUMAN;tr|J3KTJ8|J3KTJ8_HUMAN;tr|J3QRI7|J3QRI7_HUMAN;tr|J3QQQ9|J3C</td><td>42,292</td><td>246150000</td><td>19</td><td>2</td><td>0,2484922</td><td>-0,597739697</td><td>24,41306</td><td>24,16469</td><td>24,1916</td><td>23,78097</td><td>23,87708</td><td>23,58875</td><td>24,40224</td><td>22,29129</td></tr>
<tr><td>tr|A0A0J9YXZ5|A0A0J9YXZ5_HUMAN;sp|P46940|IQGA1_HUMAN;tr|H0YKA5|H0YKA5_HUMAN;</td><td>tr|A0A0J9YXZ5|A0A0J9YXZ5_HUMAN;sp|P46940|IQGA1_HUMAN</td><td>8,4215</td><td>22001000</td><td>11</td><td>5</td><td>0,25022245</td><td>-0,226283073</td><td>20,37708</td><td>20,62355</td><td>20,64095</td><td>20,70573</td><td>20,0021</td><td>20,16962</td><td>20,61171</td><td>20,65875</td></tr>
<tr><td>sp|Q92598|HS105_HUMAN;tr|A0A0A0MSM0|A0A0A0MSM0_HUMAN;tr|R4GN69|R4GN69_HU</td><td>sp|Q92598|HS105_HUMAN;tr|A0A0A0MSM0|A0A0A0MSM0_HUMAN</td><td>45,013</td><td>140530000</td><td>36</td><td>14</td><td>0,25454013</td><td>0,119031429</td><td>22,93418</td><td>23,25379</td><td>23,15957</td><td>23,12214</td><td>23,09957</td><td>23,15201</td><td>23,30966</td><td>23,38456</td></tr>
<tr><td>sp|P00491|PNPH_HUMAN;tr|G3V5M2|G3V5M2_HUMAN</td><td>sp|P00491|PNPH_HUMAN</td><td>9,3734</td><td>73366000</td><td>10</td><td>4</td><td>0,25494684</td><td>-0,4996562</td><td>21,90176</td><td>22,02807</td><td>22,43916</td><td>22,52169</td><td>21,8196</td><td>22,72178</td><td>21,11407</td><td>21,2366</td></tr>
<tr><td>sp|P00338|LDHA_HUMAN;tr|F5GXY2|F5GXY2_HUMAN;tr|F5GXH2|F5GXH2_HUMAN;tr|F5GYU2</td><td>sp|P00338|LDHA_HUMAN;tr|F5GXY2|F5GXY2_HUMAN;tr|F5GXH2|F5GXH2_HUMAN;tr|F5GYU2</td><td>114,06</td><td>1303200000</td><td>33</td><td>11</td><td>0,25524608</td><td>-0,227191448</td><td>26,69352</td><td>26,50139</td><td>26,12566</td><td>26,77316</td><td>26,16123</td><td>26,43743</td><td>26,06577</td><td>26,52054</td></tr>
<tr><td>sp|O15144|ARPC2_HUMAN;tr|H7C3F9|H7C3F9_HUMAN</td><td>sp|O15144|ARPC2_HUMAN</td><td>22,109</td><td>39130000</td><td>15</td><td>6</td><td>0,25694553</td><td>-0,224552155</td><td>21,31976</td><td>21,31324</td><td>21,59231</td><td>21,55112</td><td>20,81933</td><td>21,5545</td><td>21,10201</td><td>21,40239</td></tr>
<tr><td>sp|P13861|KAP2_HUMAN;tr|C9J830|C9J830_HUMAN;tr|H7C330|H7C330_HUMAN;tr|H7C1L0|</td><td>sp|P13861|KAP2_HUMAN;tr|C9J830|C9J830_HUMAN</td><td>11,994</td><td>25870000</td><td>11</td><td>4</td><td>0,25877805</td><td>-0,148143291</td><td>20,69693</td><td>20,70759</td><td>21,05225</td><td>20,72692</td><td>20,78987</td><td>20,75148</td><td>20,62507</td><td>20,4247</td></tr>
<tr><td>sp|O15371|EIF3D_HUMAN;tr|B0QYA4|B0QYA4_HUMAN;tr|B0QYA5|B0QYA5_HUMAN</td><td>sp|O15371|EIF3D_HUMAN;tr|B0QYA4|B0QYA4_HUMAN;tr|B0QYA5|B0QYA5_HUMAN</td><td>5,7172</td><td>30278000</td><td>6</td><td>2</td><td>0,2621654</td><td>-0,175513585</td><td>NaN</td><td>21,08031</td><td>21,42183</td><td>21,36464</td><td>20,95943</td><td>21,24603</td><td>21,13477</td><td>NaN</td></tr>
<tr><td>tr|B1ANR0|B1ANR0_HUMAN;sp|Q13310|PABP4_HUMAN;tr|H0YCC8|H0YCC8_HUMAN;tr|B1AN</td><td>tr|B1ANR0|B1ANR0_HUMAN;sp|Q13310|PABP4_HUMAN</td><td>43,615</td><td>236550000</td><td>40</td><td>1</td><td>0,26316812</td><td>0,146863937</td><td>23,7683</td><td>23,75746</td><td>24,08156</td><td>23,62165</td><td>23,84422</td><td>23,84134</td><td>24,00813</td><td>24,12274</td></tr>
<tr><td>sp|O43390|HNRPR_HUMAN;tr|B4DT28|B4DT28_HUMAN</td><td>sp|O43390|HNRPR_HUMAN;tr|B4DT28|B4DT28_HUMAN</td><td>12,664</td><td>46494000</td><td>19</td><td>6</td><td>0,26413665</td><td>-0,348011971</td><td>21,29967</td><td>21,66999</td><td>21,87433</td><td>21,45453</td><td>20,71431</td><td>21,5029</td><td>21,79317</td><td>20,89608</td></tr>
<tr><td>sp|Q99848|EBP2_HUMAN;tr|H7C2Q8|H7C2Q8_HUMAN</td><td>sp|Q99848|EBP2_HUMAN;tr|H7C2Q8|H7C2Q8_HUMAN</td><td>4,3562</td><td>15765000</td><td>7</td><td>3</td><td>0,26474014</td><td>-0,323265076</td><td>NaN</td><td>20,04166</td><td>20,3868</td><td>20,46473</td><td>NaN</td><td>19,72807</td><td>20,39843</td><td>19,7969</td></tr>
<tr><td>sp|P61026|RAB10_HUMAN;tr|H0YLJ8|H0YLJ8_HUMAN;tr|H0YL94|H0YL94_HUMAN;sp|P59190|</td><td>sp|P61026|RAB10_HUMAN</td><td>26,887</td><td>68818000</td><td>15</td><td>4</td><td>0,26835525</td><td>-0,140716076</td><td>22,04966</td><td>22,4852</td><td>22,27946</td><td>22,44998</td><td>22,17916</td><td>22,29734</td><td>22,20753</td><td>22,0174</td></tr>
<tr><td>sp|P52272|HNRPM_HUMAN;tr|A0A087X0X3|A0A087X0X3_HUMAN;tr|M0R019|M0R019_HUM</td><td>sp|P52272|HNRPM_HUMAN;tr|A0A087X0X3|A0A087X0X3_HUMAN;tr|M0R019|M0R019_HUM</td><td>198,67</td><td>635150000</td><td>37</td><td>14</td><td>0,27644635</td><td>0,215538979</td><td>25,00573</td><td>25,22295</td><td>25,3679</td><td>24,85885</td><td>25,20154</td><td>25,32158</td><td>25,72151</td><td>25,07296</td></tr>
<tr><td>sp|Q14697|GANAB_HUMAN;tr|F5H6X6|F5H6X6_HUMAN;tr|E9PKU7|E9PKU7_HUMAN;tr|E9PN</td><td>sp|Q14697|GANAB_HUMAN;tr|F5H6X6|F5H6X6_HUMAN;tr|E9PKU7|E9PKU7_HUMAN</td><td>323,31</td><td>494650000</td><td>59</td><td>18</td><td>0,27742044</td><td>-0,125590801</td><td>24,97338</td><td>25,1189</td><td>25,1949</td><td>24,95753</td><td>24,8551</td><td>24,79248</td><td>24,90408</td><td>25,19069</td></tr>
<tr><td>sp|P55084|ECHB_HUMAN;tr|F5GZQ3|F5GZQ3_HUMAN;tr|B5MD38|B5MD38_HUMAN;tr|C9JEY</td><td>sp|P55084|ECHB_HUMAN;tr|F5GZQ3|F5GZQ3_HUMAN;tr|B5MD38|B5MD38_HUMAN</td><td>7,903</td><td>38747000</td><td>11</td><td>5</td><td>0,2808212</td><td>-0,307119528</td><td>22,20323</td><td>21,48296</td><td>21,36672</td><td>21,51648</td><td>21,39147</td><td>21,54882</td><td>21,0654</td><td>NaN</td></tr>
<tr><td>sp|P61956|SUMO2_HUMAN;sp|Q6EEV6|SUMO4_HUMAN;sp|P55854|SUMO3_HUMAN;tr|A8M</td><td>sp|P61956|SUMO2_HUMAN;sp|Q6EEV6|SUMO4_HUMAN;sp|P55854|SUMO3_HUMAN;tr|A8M</td><td>10,697</td><td>105470000</td><td>9</td><td>2</td><td>0,28121828</td><td>-0,322521051</td><td>NaN</td><td>22,72293</td><td>23,00154</td><td>23,16082</td><td>22,72299</td><td>23,15023</td><td>22,15718</td><td>22,52657</td></tr>
<tr><td>sp|Q9HB71|CYBP_HUMAN;tr|B2ZWH1|B2ZWH1_HUMAN</td><td>sp|Q9HB71|CYBP_HUMAN;tr|B2ZWH1|B2ZWH1_HUMAN</td><td>37,96</td><td>55912000</td><td>9</td><td>4</td><td>0,28379408</td><td>0,147529125</td><td>21,98109</td><td>21,67784</td><td>21,5595</td><td>21,82089</td><td>22,065</td><td>21,95978</td><td>21,66206</td><td>21,9426</td></tr>
<tr><td>sp|P62249|RS16_HUMAN;tr|M0R210|M0R210_HUMAN;tr|A0A087WZ27|A0A087WZ27_HUMA</td><td>sp|P62249|RS16_HUMAN;tr|M0R210|M0R210_HUMAN;tr|A0A087WZ27|A0A087WZ27_HUMA</td><td>158,91</td><td>1631000000</td><td>50</td><td>8</td><td>0,2862231</td><td>0,272425175</td><td>26,26209</td><td>26,83219</td><td>26,73518</td><td>26,11333</td><td>27,07286</td><td>26,93955</td><td>26,61989</td><td>26,4002</td></tr>
<tr><td>sp|P18669|PGAM1_HUMAN;sp|P15259|PGAM2_HUMAN;sp|Q8N0Y7|PGAM4_HUMAN</td><td>sp|P18669|PGAM1_HUMAN;sp|P15259|PGAM2_HUMAN</td><td>26,118</td><td>94667000</td><td>11</td><td>4</td><td>0,29036196</td><td>-0,235364437</td><td>22,77509</td><td>22,65823</td><td>22,81617</td><td>23,02862</td><td>22,43145</td><td>22,88069</td><td>22,12414</td><td>22,90036</td></tr>
<tr><td>tr|A0A0A0MSJ0|A0A0A0MSJ0_HUMAN;sp|Q86XP3|DDX42_HUMAN;tr|J3KRE3|J3KRE3_HUMAN</td><td>tr|A0A0A0MSJ0|A0A0A0MSJ0_HUMAN;sp|Q86XP3|DDX42_HUMAN</td><td>4,1091</td><td>12491000</td><td>3</td><td>3</td><td>0,29064308</td><td>-0,11228466</td><td>19,74319</td><td>19,78705</td><td>19,76633</td><td>19,87938</td><td>19,67984</td><td>19,47508</td><td>19,64883</td><td>19,92305</td></tr>
<tr><td>tr|F8WDD7|F8WDD7_HUMAN;sp|P59998|ARPC4_HUMAN;tr|F8WCF6|F8WCF6_HUMAN;tr|A0A</td><td>tr|F8WDD7|F8WDD7_HUMAN;sp|P59998|ARPC4_HUMAN;tr|F8WCF6|F8WCF6_HUMAN;tr|A0A</td><td>3,2186</td><td>29835000</td><td>7</td><td>3</td><td>0,29376031</td><td>0,73793443</td><td>21,34408</td><td>19,88341</td><td>19,75285</td><td>NaN</td><td>21,66787</td><td>21,31341</td><td>21,36164</td><td>19,91595</td></tr>
<tr><td>tr|J3KN66|J3KN66_HUMAN;tr|A0A0A0MSK5|A0A0A0MSK5_HUMAN;sp|Q5JTV8|TOIP1_HUMAN</td><td>tr|J3KN66|J3KN66_HUMAN;tr|A0A0A0MSK5|A0A0A0MSK5_HUMAN;sp|Q5JTV8|TOIP1_HUMAN</td><td>3,6801</td><td>17080000</td><td>5</td><td>4</td><td>0,29452134</td><td>0,25689888</td><td>NaN</td><td>20,23327</td><td>19,79927</td><td>20,33123</td><td>20,60738</td><td>20,1342</td><td>NaN</td><td>20,39289</td></tr>
<tr><td>sp|Q8WWM7|ATX2L_HUMAN;tr|H3BUF6|H3BUF6_HUMAN;tr|H3BSK9|H3BSK9_HUMAN</td><td>sp|Q8WWM7|ATX2L_HUMAN;tr|H3BUF6|H3BUF6_HUMAN</td><td>5,0985</td><td>31274000</td><td>9</td><td>4</td><td>0,29630579</td><td>0,320298672</td><td>21,15221</td><td>20,72267</td><td>20,14967</td><td>20,83854</td><td>21,24045</td><td>20,4804</td><td>21,17426</td><td>21,24916</td></tr>
<tr><td>sp|P20700|LMNB1_HUMAN;tr|E9PBF6|E9PBF6_HUMAN;tr|A0A0D9SFE5|A0A0D9SFE5_HUMAN</td><td>sp|P20700|LMNB1_HUMAN;tr|E9PBF6|E9PBF6_HUMAN;tr|A0A0D9SFE5|A0A0D9SFE5_HUMAN</td><td>80,849</td><td>445540000</td><td>54</td><td>19</td><td>0,29846155</td><td>0,455297947</td><td>24,32971</td><td>23,90242</td><td>24,12755</td><td>24,57906</td><td>23,92877</td><td>25,71487</td><td>24,6142</td><td>24,50209</td></tr>
<tr><td>sp|Q15029|U5S1_HUMAN</td><td>sp|Q15029|U5S1_HUMAN</td><td>19,558</td><td>63837000</td><td>23</td><td>8</td><td>0,30163424</td><td>0,129235744</td><td>21,97495</td><td>21,8711</td><td>21,9441</td><td>21,87845</td><td>21,77961</td><td>21,94621</td><td>22,21045</td><td>22,24928</td></tr>
<tr><td>sp|P21796|VDAC1_HUMAN;tr|C9JI87|C9JI87_HUMAN</td><td>sp|P21796|VDAC1_HUMAN</td><td>6,2642</td><td>22092000</td><td>6</td><td>3</td><td>0,30304258</td><td>0,436901569</td><td>19,55127</td><td>19,64484</td><td>19,66622</td><td>20,02134</td><td>19,92093</td><td>21,21957</td><td>20,02296</td><td>19,46782</td></tr>
<tr><td>sp|Q9Y678|COPG1_HUMAN;tr|H0Y8X7|H0Y8X7_HUMAN</td><td>sp|Q9Y678|COPG1_HUMAN</td><td>9,5376</td><td>20421000</td><td>11</td><td>4</td><td>0,30337307</td><td>-0,254998684</td><td>20,43145</td><td>20,3721</td><td>20,47773</td><td>20,69753</td><td>20,14892</td><td>20,8233</td><td>20,19976</td><td>19,78683</td></tr>
<tr><td>tr|A0A0C4DGS1|A0A0C4DGS1_HUMAN;sp|P39656|OST48_HUMAN;tr|U3KQ84|U3KQ84_HUMA</td><td>tr|A0A0C4DGS1|A0A0C4DGS1_HUMAN;sp|P39656|OST48_HUMAN</td><td>18,763</td><td>94076000</td><td>29</td><td>8</td><td>0,30342991</td><td>0,15580225</td><td>22,48508</td><td>22,2856</td><td>22,63285</td><td>22,61385</td><td>22,39482</td><td>22,60865</td><td>22,69598</td><td>22,94113</td></tr>
<tr><td>sp|Q15005|SPCS2_HUMAN;tr|A0A087WUC6|A0A087WUC6_HUMAN;tr|E9PI68|E9PI68_HUMAN</td><td>sp|Q15005|SPCS2_HUMAN;tr|A0A087WUC6|A0A087WUC6_HUMAN;tr|E9PI68|E9PI68_HUMAN</td><td>3,7288</td><td>17229000</td><td>4</td><td>2</td><td>0,30597664</td><td>0,380987962</td><td>NaN</td><td>20,10815</td><td>19,92929</td><td>20,07509</td><td>21,19292</td><td>19,88445</td><td>20,41142</td><td>20,18519</td></tr>
<tr><td>tr|A0A0U1RQH7|A0A0U1RQH7_HUMAN;tr|H0Y4X3|H0Y4X3_HUMAN;tr|G3XAC6|G3XAC6_HUM</td><td>tr|A0A0U1RQH7|A0A0U1RQH7_HUMAN;tr|H0Y4X3|H0Y4X3_HUMAN;tr|G3XAC6|G3XAC6_HUM</td><td>32,333</td><td>40157000</td><td>12</td><td>5</td><td>0,31028886</td><td>-0,450322628</td><td>21,61526</td><td>21,4079</td><td>20,56347</td><td>22,4986</td><td>21,13621</td><td>21,02608</td><td>21,26264</td><td>20,85901</td></tr>
<tr><td>sp|P38606|VATA_HUMAN;tr|C9JA17|C9JA17_HUMAN;tr|C9JVW8|C9JVW8_HUMAN</td><td>sp|P38606|VATA_HUMAN</td><td>11,257</td><td>38875000</td><td>14</td><td>7</td><td>0,31521889</td><td>0,135597706</td><td>21,61275</td><td>21,20652</td><td>21,08219</td><td>21,3274</td><td>21,47353</td><td>21,30011</td><td>21,46996</td><td>21,52765</td></tr>
<tr><td>sp|P21281|VATB2_HUMAN;tr|H0YC04|H0YC04_HUMAN;tr|C9JL73|C9JL73_HUMAN;sp|P15313</td><td>sp|P21281|VATB2_HUMAN;tr|H0YC04|H0YC04_HUMAN;tr|C9JL73|C9JL73_HUMAN;sp|P15313</td><td>3,693</td><td>7152300</td><td>7</td><td>2</td><td>0,31972039</td><td>-0,162288189</td><td>18,97958</td><td>18,90745</td><td>19,02443</td><td>19,05973</td><td>18,89457</td><td>18,83556</td><td>18,44298</td><td>19,14892</td></tr>
<tr><td>sp|P60953|CDC42_HUMAN;tr|Q5JYX0|Q5JYX0_HUMAN</td><td>sp|P60953|CDC42_HUMAN;tr|Q5JYX0|Q5JYX0_HUMAN</td><td>85,291</td><td>28187000</td><td>10</td><td>3</td><td>0,3198627</td><td>0,641531467</td><td>19,94192</td><td>19,60206</td><td>20,07744</td><td>19,93531</td><td>20,05854</td><td>22,24519</td><td>19,64146</td><td>20,17767</td></tr>
<tr><td>tr|K7EJC1|K7EJC1_HUMAN;tr|K7EJR3|K7EJR3_HUMAN;tr|R4GMR5|R4GMR5_HUMAN;sp|P4855</td><td>tr|K7EJC1|K7EJC1_HUMAN;tr|K7EJR3|K7EJR3_HUMAN;tr|R4GMR5|R4GMR5_HUMAN;sp|P4855</td><td>3,8714</td><td>49682000</td><td>8</td><td>3</td><td>0,32208442</td><td>-0,161662102</td><td>21,85962</td><td>21,89504</td><td>21,82889</td><td>21,55225</td><td>21,80365</td><td>21,80664</td><td>21,2635</td><td>21,61535</td></tr>
<tr><td>sp|P62805|H4_HUMAN</td><td>sp|P62805|H4_HUMAN</td><td>322,23</td><td>3232700000</td><td>41</td><td>9</td><td>0,32843309</td><td>0,926549435</td><td>25,93159</td><td>25,05001</td><td>27,38105</td><td>27,92603</td><td>25,91593</td><td>27,99814</td><td>28,56518</td><td>27,51563</td></tr>
<tr><td>sp|O95373|IPO7_HUMAN</td><td>sp|O95373|IPO7_HUMAN</td><td>31,034</td><td>72663000</td><td>22</td><td>7</td><td>0,33486215</td><td>-0,27458477</td><td>22,24826</td><td>22,2252</td><td>22,35329</td><td>22,41612</td><td>21,27748</td><td>22,17234</td><td>22,2723</td><td>22,42242</td></tr>
<tr><td>sp|P51398|RT29_HUMAN;tr|V9GYL9|V9GYL9_HUMAN;tr|V9GYF7|V9GYF7_HUMAN;tr|V9GZ61|</td><td>sp|P51398|RT29_HUMAN;tr|V9GYL9|V9GYL9_HUMAN</td><td>9,0526</td><td>15504000</td><td>9</td><td>4</td><td>0,33667055</td><td>0,17797184</td><td>19,65979</td><td>19,79532</td><td>19,8624</td><td>19,85641</td><td>20,18628</td><td>19,67735</td><td>20,31628</td><td>19,7059</td></tr>
<tr><td>sp|Q9UN86|G3BP2_HUMAN</td><td>sp|Q9UN86|G3BP2_HUMAN</td><td>9,1773</td><td>54517000</td><td>10</td><td>2</td><td>0,33715839</td><td>0,219678402</td><td>21,75022</td><td>21,51031</td><td>21,84157</td><td>21,27219</td><td>21,45643</td><td>21,86219</td><td>22,24916</td><td>21,68521</td></tr>
<tr><td>sp|P09874|PARP1_HUMAN;tr|Q5VX84|Q5VX84_HUMAN;tr|Q5VX85|Q5VX85_HUMAN</td><td>sp|P09874|PARP1_HUMAN</td><td>323,31</td><td>588440000</td><td>79</td><td>25</td><td>0,3383473</td><td>-0,149561882</td><td>25,32663</td><td>25,32244</td><td>25,32543</td><td>25,28602</td><td>24,97176</td><td>24,91315</td><td>25,23685</td><td>25,54053</td></tr>
<tr><td>sp|Q86V81|THOC4_HUMAN;tr|E9PB61|E9PB61_HUMAN</td><td>sp|Q86V81|THOC4_HUMAN;tr|E9PB61|E9PB61_HUMAN</td><td>111,95</td><td>83532000</td><td>15</td><td>4</td><td>0,34206675</td><td>0,238220692</td><td>22,21617</td><td>21,46234</td><td>22,36586</td><td>22,1717</td><td>22,42355</td><td>22,5087</td><td>22,00014</td><td>22,23658</td></tr>
<tr><td>sp|Q92688|AN32B_HUMAN</td><td>sp|Q92688|AN32B_HUMAN</td><td>5,7779</td><td>70821000</td><td>7</td><td>3</td><td>0,34214004</td><td>-0,317149639</td><td>22,19547</td><td>22,32808</td><td>22,42406</td><td>22,93776</td><td>21,90205</td><td>22,71123</td><td>NaN</td><td>21,84931</td></tr>
<tr><td>tr|A0A0B4J1Z1|A0A0B4J1Z1_HUMAN;tr|C9JAB2|C9JAB2_HUMAN;sp|Q16629|SRSF7_HUMAN</td><td>tr|A0A0B4J1Z1|A0A0B4J1Z1_HUMAN;tr|C9JAB2|C9JAB2_HUMAN;sp|Q16629|SRSF7_HUMAN</td><td>40,601</td><td>67688000</td><td>11</td><td>3</td><td>0,34316575</td><td>0,456363837</td><td>22,01509</td><td>20,92492</td><td>22,42627</td><td>22,00206</td><td>NaN</td><td>22,18882</td><td>22,78531</td><td>21,92122</td></tr>
<tr><td>sp|Q16777|H2A2C_HUMAN;sp|Q6FI13|H2A2A_HUMAN</td><td>sp|Q16777|H2A2C_HUMAN;sp|Q6FI13|H2A2A_HUMAN</td><td>323,31</td><td>2491900000</td><td>27</td><td>1</td><td>0,34458425</td><td>0,848398209</td><td>25,74491</td><td>25,18079</td><td>27,06332</td><td>27,22803</td><td>25,33214</td><td>27,51758</td><td>28,47127</td><td>27,28965</td></tr>
<tr><td>sp|P50454|SERPH_HUMAN;tr|E9PJH8|E9PJH8_HUMAN;tr|E9PIG2|E9PIG2_HUMAN;tr|E9PRS3|</td><td>sp|P50454|SERPH_HUMAN;tr|E9PJH8|E9PJH8_HUMAN;tr|E9PIG2|E9PIG2_HUMAN;tr|E9PRS3|</td><td>6,1155</td><td>17399000</td><td>3</td><td>3</td><td>0,35691633</td><td>0,115469933</td><td>20,12351</td><td>20,12931</td><td>19,86582</td><td>20,32893</td><td>20,39184</td><td>20,14445</td><td>20,09893</td><td>20,27424</td></tr>
<tr><td>sp|Q5SRE5|NU188_HUMAN</td><td>sp|Q5SRE5|NU188_HUMAN</td><td>1,9449</td><td>6438000</td><td>3</td><td>3</td><td>0,35807135</td><td>-0,13048172</td><td>18,87781</td><td>18,75012</td><td>18,92187</td><td>18,78302</td><td>18,5713</td><td>18,48129</td><td>18,70819</td><td>19,05011</td></tr>
<tr><td>sp|Q01813|PFKAP_HUMAN;tr|V9GY25|V9GY25_HUMAN;tr|B1APP6|B1APP6_HUMAN;tr|B1APF</td><td>sp|Q01813|PFKAP_HUMAN;tr|V9GY25|V9GY25_HUMAN;tr|B1APP6|B1APP6_HUMAN</td><td>4,994</td><td>58581000</td><td>8</td><td>2</td><td>0,36096174</td><td>-0,124455452</td><td>21,6657</td><td>22,05043</td><td>22,05295</td><td>22,09388</td><td>22,06194</td><td>21,81917</td><td>21,74456</td><td>21,73946</td></tr>
<tr><td>sp|Q96AG4|LRC59_HUMAN;tr|I3L223|I3L223_HUMAN</td><td>sp|Q96AG4|LRC59_HUMAN</td><td>155,83</td><td>218760000</td><td>26</td><td>7</td><td>0,36227134</td><td>0,154519558</td><td>23,41828</td><td>23,78347</td><td>23,9393</td><td>23,51461</td><td>23,99162</td><td>23,94842</td><td>23,78766</td><td>23,54604</td></tr>
<tr><td>tr|A0A0A0MTN3|A0A0A0MTN3_HUMAN;sp|P21266|GSTM3_HUMAN;tr|E9PLF1|E9PLF1_HUMA</td><td>tr|A0A0A0MTN3|A0A0A0MTN3_HUMAN;sp|P21266|GSTM3_HUMAN;tr|E9PLF1|E9PLF1_HUMA</td><td>5,9298</td><td>19197000</td><td>6</td><td>2</td><td>0,36325052</td><td>0,334290187</td><td>20,6856</td><td>20,51557</td><td>20,43777</td><td>20,48227</td><td>20,27889</td><td>21,60923</td><td>20,70565</td><td>NaN</td></tr>
<tr><td>tr|E9PID8|E9PID8_HUMAN;sp|P33240|CSTF2_HUMAN;tr|E7EWR4|E7EWR4_HUMAN;sp|Q9H0L</td><td>tr|E9PID8|E9PID8_HUMAN;sp|P33240|CSTF2_HUMAN;tr|E7EWR4|E7EWR4_HUMAN;sp|Q9H0L</td><td>4,1863</td><td>17073000</td><td>5</td><td>2</td><td>0,3644673</td><td>-0,076714198</td><td>20,25026</td><td>20,26957</td><td>20,40748</td><td>20,22847</td><td>20,28681</td><td>20,06815</td><td>20,28173</td><td>NaN</td></tr>
<tr><td>sp|O95347|SMC2_HUMAN</td><td>sp|O95347|SMC2_HUMAN</td><td>7,2129</td><td>28674000</td><td>8</td><td>6</td><td>0,36754493</td><td>-0,104292393</td><td>20,9885</td><td>21,16183</td><td>20,97771</td><td>20,7128</td><td>20,71011</td><td>20,96874</td><td>20,87079</td><td>20,87403</td></tr>
<tr><td>sp|P08238|HS90B_HUMAN;sp|Q58FF7|H90B3_HUMAN</td><td>sp|P08238|HS90B_HUMAN</td><td>323,31</td><td>4256900000</td><td>152</td><td>13</td><td>0,36845341</td><td>-0,125599861</td><td>28,15822</td><td>28,12169</td><td>28,31592</td><td>28,24082</td><td>28,03318</td><td>28,05533</td><td>27,83032</td><td>28,41542</td></tr>
<tr><td>sp|P49755|TMEDA_HUMAN;tr|G3V2K7|G3V2K7_HUMAN</td><td>sp|P49755|TMEDA_HUMAN;tr|G3V2K7|G3V2K7_HUMAN</td><td>20,992</td><td>69604000</td><td>12</td><td>5</td><td>0,3695902</td><td>-0,103110313</td><td>22,06145</td><td>22,16002</td><td>22,38184</td><td>22,02181</td><td>22,05165</td><td>22,14127</td><td>22,1618</td><td>21,85795</td></tr>
<tr><td>sp|Q96CX2|KCD12_HUMAN;sp|Q68DU8|KCD16_HUMAN</td><td>sp|Q96CX2|KCD12_HUMAN</td><td>37,436</td><td>39727000</td><td>15</td><td>6</td><td>0,37353564</td><td>-0,071588993</td><td>21,31689</td><td>21,44673</td><td>21,27804</td><td>21,40681</td><td>21,27071</td><td>21,27923</td><td>21,15233</td><td>21,45984</td></tr>
<tr><td>sp|P31153|METK2_HUMAN;sp|Q00266|METK1_HUMAN</td><td>sp|P31153|METK2_HUMAN</td><td>21,773</td><td>107640000</td><td>19</td><td>6</td><td>0,37363819</td><td>-0,201898098</td><td>22,84573</td><td>23,0012</td><td>22,89992</td><td>23,13127</td><td>22,88327</td><td>22,96104</td><td>22,17509</td><td>23,05114</td></tr>
<tr><td>sp|P62937|PPIA_HUMAN;tr|F8WE65|F8WE65_HUMAN;tr|C9J5S7|C9J5S7_HUMAN;tr|E5RIZ5|E</td><td>sp|P62937|PPIA_HUMAN;tr|F8WE65|F8WE65_HUMAN;tr|C9J5S7|C9J5S7_HUMAN</td><td>323,31</td><td>644280000</td><td>33</td><td>7</td><td>0,37387399</td><td>-0,427820683</td><td>25,33412</td><td>25,29747</td><td>25,41783</td><td>25,83178</td><td>24,72765</td><td>25,10221</td><td>24,15429</td><td>26,18577</td></tr>
<tr><td>tr|A0A024RCR6|A0A024RCR6_HUMAN;tr|A0A0G2JK23|A0A0G2JK23_HUMAN;sp|P46379|BAG6</td><td>tr|A0A024RCR6|A0A024RCR6_HUMAN;tr|A0A0G2JK23|A0A0G2JK23_HUMAN;sp|P46379|BAG6</td><td>26,116</td><td>32358000</td><td>19</td><td>5</td><td>0,37425469</td><td>-0,395913601</td><td>20,62952</td><td>21,4224</td><td>20,63901</td><td>22,24771</td><td>21,12584</td><td>20,46204</td><td>20,75849</td><td>21,00861</td></tr>
<tr><td>sp|Q9Y3U8|RL36_HUMAN;tr|J3QSB5|J3QSB5_HUMAN;tr|J3KTD3|J3KTD3_HUMAN</td><td>sp|Q9Y3U8|RL36_HUMAN;tr|J3QSB5|J3QSB5_HUMAN</td><td>12,636</td><td>88961000</td><td>12</td><td>4</td><td>0,38435316</td><td>-0,415513992</td><td>21,48015</td><td>23,48591</td><td>23,0806</td><td>22,69013</td><td>22,54571</td><td>22,177</td><td>22,14622</td><td>22,2058</td></tr>
<tr><td>sp|P61221|ABCE1_HUMAN;tr|D6R9I9|D6R9I9_HUMAN;tr|D6RGF4|D6RGF4_HUMAN;tr|H0Y99</td><td>sp|P61221|ABCE1_HUMAN;tr|D6R9I9|D6R9I9_HUMAN</td><td>37,584</td><td>143890000</td><td>28</td><td>12</td><td>0,38481038</td><td>-0,191519737</td><td>23,3181</td><td>23,48971</td><td>23,31948</td><td>23,37981</td><td>23,02753</td><td>23,13955</td><td>22,82118</td><td>23,75277</td></tr>
<tr><td>sp|P30084|ECHM_HUMAN</td><td>sp|P30084|ECHM_HUMAN</td><td>33,718</td><td>57076000</td><td>15</td><td>4</td><td>0,38504904</td><td>-0,167257786</td><td>21,70134</td><td>21,87811</td><td>21,85011</td><td>22,29393</td><td>21,78161</td><td>21,89182</td><td>21,4065</td><td>21,97453</td></tr>
<tr><td>sp|Q00796|DHSO_HUMAN;tr|H0YLA4|H0YLA4_HUMAN;tr|H0YKB3|H0YKB3_HUMAN</td><td>sp|Q00796|DHSO_HUMAN;tr|H0YLA4|H0YLA4_HUMAN</td><td>4,1062</td><td>27191000</td><td>10</td><td>3</td><td>0,38632788</td><td>-0,304616928</td><td>20,63112</td><td>20,95851</td><td>21,43695</td><td>21,53758</td><td>20,7102</td><td>21,56983</td><td>20,53479</td><td>20,53089</td></tr>
<tr><td>sp|Q9BRA2|TXD17_HUMAN;tr|I3L0K2|I3L0K2_HUMAN;tr|I3L3M7|I3L3M7_HUMAN;tr|I3L2R6|I</td><td>sp|Q9BRA2|TXD17_HUMAN;tr|I3L0K2|I3L0K2_HUMAN;tr|I3L3M7|I3L3M7_HUMAN;tr|I3L2R6|I</td><td>5,1843</td><td>17722000</td><td>3</td><td>3</td><td>0,38770963</td><td>-0,194065412</td><td>20,27912</td><td>20,31606</td><td>20,3739</td><td>NaN</td><td>20,06026</td><td>20,57034</td><td>19,7336</td><td>20,15165</td></tr>
<tr><td>tr|H0YL72|H0YL72_HUMAN;sp|P50213|IDH3A_HUMAN;tr|H0YMU3|H0YMU3_HUMAN;tr|H0YL</td><td>tr|H0YL72|H0YL72_HUMAN;sp|P50213|IDH3A_HUMAN;tr|H0YMU3|H0YMU3_HUMAN;tr|H0YL</td><td>7,4602</td><td>87143000</td><td>7</td><td>6</td><td>0,38947229</td><td>0,416396936</td><td>21,67211</td><td>21,95369</td><td>22,54402</td><td>22,5612</td><td>23,15971</td><td>22,87418</td><td>NaN</td><td>21,76357</td></tr>
<tr><td>sp|P40939|ECHA_HUMAN;tr|H0YFD6|H0YFD6_HUMAN</td><td>sp|P40939|ECHA_HUMAN;tr|H0YFD6|H0YFD6_HUMAN</td><td>15,19</td><td>54436000</td><td>21</td><td>7</td><td>0,40387191</td><td>-0,159707069</td><td>21,80298</td><td>21,54213</td><td>22,15106</td><td>21,80077</td><td>21,54279</td><td>21,79606</td><td>21,9426</td><td>21,37666</td></tr>
<tr><td>tr|K7EQQ8|K7EQQ8_HUMAN;sp|Q9NXH9|TRM1_HUMAN;tr|K7EQU7|K7EQU7_HUMAN;tr|K7EJ</td><td>tr|K7EQQ8|K7EQQ8_HUMAN;sp|Q9NXH9|TRM1_HUMAN</td><td>3,0056</td><td>6840800</td><td>4</td><td>3</td><td>0,40990098</td><td>0,162866592</td><td>18,56253</td><td>NaN</td><td>18,75411</td><td>18,86876</td><td>18,82208</td><td>19,18403</td><td>18,66789</td><td>NaN</td></tr>
<tr><td>tr|F2Z2V0|F2Z2V0_HUMAN;sp|Q99829|CPNE1_HUMAN;tr|B0QZ18|B0QZ18_HUMAN;tr|A6PVH</td><td>tr|F2Z2V0|F2Z2V0_HUMAN;sp|Q99829|CPNE1_HUMAN;tr|B0QZ18|B0QZ18_HUMAN;tr|A6PVH</td><td>46,997</td><td>31837000</td><td>15</td><td>5</td><td>0,41032643</td><td>0,120182037</td><td>21,10169</td><td>20,85049</td><td>20,90529</td><td>21,29569</td><td>20,98029</td><td>21,39367</td><td>21,06112</td><td>21,1988</td></tr>
<tr><td>sp|P08243|ASNS_HUMAN;tr|F8WEJ5|F8WEJ5_HUMAN;tr|C9J057|C9J057_HUMAN</td><td>sp|P08243|ASNS_HUMAN</td><td>9,1305</td><td>46887000</td><td>9</td><td>5</td><td>0,41059825</td><td>-0,233998775</td><td>21,40484</td><td>21,62649</td><td>21,51917</td><td>22,37132</td><td>21,5657</td><td>21,50257</td><td>21,42111</td><td>NaN</td></tr>
<tr><td>tr|A0A096LNY6|A0A096LNY6_HUMAN;sp|P30566|PUR8_HUMAN;tr|A0A0A6YY92|A0A0A6YY92</td><td>tr|A0A096LNY6|A0A096LNY6_HUMAN;sp|P30566|PUR8_HUMAN;tr|A0A0A6YY92|A0A0A6YY92</td><td>3,2346</td><td>18991000</td><td>6</td><td>2</td><td>0,41113468</td><td>-0,087504069</td><td>20,59113</td><td>20,53592</td><td>20,65875</td><td>20,49843</td><td>20,54821</td><td>20,27685</td><td>NaN</td><td>20,6256</td></tr>
<tr><td>sp|P35606|COPB2_HUMAN;tr|D6R997|D6R997_HUMAN;tr|D6RBZ7|D6RBZ7_HUMAN;tr|D6RCL</td><td>sp|P35606|COPB2_HUMAN</td><td>21,204</td><td>33037000</td><td>12</td><td>8</td><td>0,4113377</td><td>-0,137621403</td><td>21,21175</td><td>21,17286</td><td>21,18519</td><td>20,96522</td><td>21,08661</td><td>20,56449</td><td>21,15468</td><td>21,17877</td></tr>
</table>

sp|Q15021|CND1_HUMAN;tr|E7EN77|E7EN77_HUMAN	sp|Q15021|CND1_HUMAN	5,3087	8809600	4	3	0,4118117	-0,248518626	NaN	19,56657	19,65571	18,84252	NaN	18,94241	19,14298	19,23386
sp|P49006|MRP_HUMAN	sp|P49006|MRP_HUMAN	27,905	29785000	7	2	0,41585255	-0,234923045	21,10604	20,97715	NaN	21,0426	20,84069	21,23333	20,347	NaN
sp|Q8N257|H2B3B_HUMAN;sp|Q16778|H2B2E_HUMAN;sp|P33778|H2B1B_HUMAN;sp|P2352	sp|Q8N257|H2B3B_HUMAN;sp|Q16778|H2B2E_HUMAN;sp|P33778|H2B1B_HUMAN;sp|P2352	321,21	1781800000	23	2	0,41951206	0,882318497	24,97058	24,073	26,73609	26,99118	24,63323	26,8992	28,20191	26,56578
sp|P48444|COPD_HUMAN;tr|B0YIW6|B0YIW6_HUMAN;tr|Q6P1Q5|Q6P1Q5_HUMAN;tr|E9PK3	sp|P48444|COPD_HUMAN;tr|B0YIW6|B0YIW6_HUMAN;tr|Q6P1Q5|Q6P1Q5_HUMAN	18,195	41728000	12	4	0,42436217	-0,064091682	21,27469	21,53711	21,45623	21,40322	21,40265	21,30536	21,46742	21,23946
sp|O75533|SF3B1_HUMAN;tr|H7C341|H7C341_HUMAN	sp|O75533|SF3B1_HUMAN	71,404	99284000	22	9	0,43031619	-0,257849216	22,88373	22,80554	22,73411	22,61113	21,66891	22,46107	22,89946	22,97369
sp|P42166|LAP2A_HUMAN	sp|P42166|LAP2A_HUMAN	44,134	178700000	48	10	0,43482211	-0,234984398	23,29866	23,29642	23,75929	23,76415	23,34394	23,19831	23,91743	22,7189
sp|Q9BSJ8|ESYT1_HUMAN;tr|F8VZB1|F8VZB1_HUMAN	sp|Q9BSJ8|ESYT1_HUMAN	14,69	34666000	22	5	0,43817812	-0,078159809	21,35692	21,17804	21,15851	21,16484	21,05484	20,94999	21,24034	21,3005
sp|Q9UHB9|SRP68_HUMAN	sp|Q9UHB9|SRP68_HUMAN	23,709	34451000	12	6	0,4385765	-0,071462631	21,22117	21,14892	21,29182	21,33615	21,18235	21,38785	21,04019	21,10181
sp|P55072|TERA_HUMAN;tr|C9JUP7|C9JUP7_HUMAN;tr|C9IZA5|C9IZA5_HUMAN	sp|P55072|TERA_HUMAN	41,414	258990000	52	15	0,44006306	-0,069813251	23,99981	23,93517	24,2522	24,08229	23,87998	24,08869	23,95296	24,06858
sp|O95436|NPT2B_HUMAN	sp|O95436|NPT2B_HUMAN	1,626	81232000	5	2	0,44460138	-0,250239849	23,00703	22,91614	21,75307	22,59798	22,5078	22,48311	22,0459	22,23646
sp|P63000|RAC1_HUMAN;sp|P60763|RAC3_HUMAN;sp|P15153|RAC2_HUMAN;tr|J3KSC4|J3KS	sp|P63000|RAC1_HUMAN;sp|P60763|RAC3_HUMAN;sp|P15153|RAC2_HUMAN;tr|J3KSC4|J3KS	2,5272	50325000	7	3	0,44501891	0,522945245	20,14669	20,8064	21,73715	21,10495	20,9686	22,65176	20,79487	NaN
tr|J9JID7|J9JID7_HUMAN;sp|Q03252|LMNB2_HUMAN	tr|J9JID7|J9JID7_HUMAN;sp|Q03252|LMNB2_HUMAN	17,799	74198000	15	8	0,45109064	0,299171925	21,82877	21,74053	22,07633	21,90367	21,46832	23,10537	22,41235	21,75996
sp|P04844|RPN2_HUMAN;tr|Q5JYR7|Q5JYR7_HUMAN;tr|F2Z3K5|F2Z3K5_HUMAN;tr|Q5JYR4|(	sp|P04844|RPN2_HUMAN	190,49	132650000	36	12	0,45475998	-0,104206085	23,04006	22,92922	23,32045	23,14331	22,96534	22,84378	23,29614	22,91095
sp|O76094|SRP72_HUMAN;tr|R4GNC1|R4GNC1_HUMAN;tr|D6RDY6|D6RDY6_HUMAN	sp|O76094|SRP72_HUMAN	11,37	25793000	9	5	0,45793915	-0,20364507	21,28167	20,48572	21,13659	NaN	NaN	20,68243	20,80719	20,80341
sp|P11387|TOP1_HUMAN;tr|E5RJ95|E5RJ95_HUMAN;tr|E5RFS0|E5RFS0_HUMAN;tr|E5RIC7|E5	sp|P11387|TOP1_HUMAN	13,023	43731000	7	4	0,46299284	0,203182697	21,47526	21,06881	20,96437	21,68282	21,12231	21,32075	22,03644	21,52449
sp|P07195|LDHB_HUMAN;tr|A8MW50|A8MW50_HUMAN;tr|F5H793|F5H793_HUMAN;tr|C9J7	sp|P07195|LDHB_HUMAN;tr|A8MW50|A8MW50_HUMAN;tr|F5H793|F5H793_HUMAN;tr|C9J7	323,31	1487400000	50	9	0,46365622	-0,132631779	27,00185	26,53614	26,53906	26,79266	26,54822	26,71751	26,24525	26,8282
sp|O43175|SERA_HUMAN;tr|Q5SZU1|Q5SZU1_HUMAN	sp|O43175|SERA_HUMAN;tr|Q5SZU1|Q5SZU1_HUMAN	30,501	299510000	39	12	0,4710846	-0,125378609	24,22619	24,39126	24,3123	24,52103	24,20464	24,21823	23,89634	24,63006
sp|P35250|RFC2_HUMAN;tr|H7C5P4|H7C5P4_HUMAN;tr|A0A087WVY3|A0A087WVY3_HUMAN	sp|P35250|RFC2_HUMAN;tr|H7C5P4|H7C5P4_HUMAN;tr|A0A087WVY3|A0A087WVY3_HUMAN	4,7165	20837000	8	4	0,47307405	0,092873096	20,23585	20,46533	20,45393	20,57191	20,68286	20,26511	20,47645	20,67409
sp|Q14974|IMB1_HUMAN;tr|J3KTM9|J3KTM9_HUMAN;tr|J3QR48|J3QR48_HUMAN;tr|J3QKQ5	sp|Q14974|IMB1_HUMAN;tr|J3KTM9|J3KTM9_HUMAN	323,31	414920000	54	16	0,47679676	0,12569046	24,79615	24,61341	24,64548	24,67357	24,74181	24,55894	24,6536	25,27704
sp|O43852|CALU_HUMAN;tr|H0Y875|H0Y875_HUMAN	sp|O43852|CALU_HUMAN;tr|H0Y875|H0Y875_HUMAN	24,381	102760000	21	7	0,48899149	0,137719154	22,23017	22,66262	22,8477	22,52578	22,7599	22,87693	22,87044	22,30989
sp|P31942|HNRH3_HUMAN	sp|P31942|HNRH3_HUMAN	23,963	60412000	16	4	0,49053858	0,234766483	21,18804	21,17883	22,09497	21,87083	21,52979	22,10124	22,26728	21,37342
sp|P29692|EF1D_HUMAN;tr|E9PK01|E9PK01_HUMAN;tr|E9PRY8|E9PRY8_HUMAN;tr|E9PMW7	sp|P29692|EF1D_HUMAN;tr|E9PK01|E9PK01_HUMAN;tr|E9PRY8|E9PRY8_HUMAN;tr|E9PMW7	71,865	161890000	22	5	0,49741701	-0,115264893	23,48431	23,15647	23,62255	23,52456	23,3839	23,32334	23,0103	23,6093
sp|Q9Y617|SERC_HUMAN	sp|Q9Y617|SERC_HUMAN	13,727	119570000	20	8	0,49923489	-0,095963478	22,74189	23,01711	22,94156	23,16873	22,63163	23,07233	22,78903	22,99245
sp|Q12931|TRAP1_HUMAN;tr|I3L0K7|I3L0K7_HUMAN;tr|I3L239|I3L239_HUMAN;tr|I3L253|I3L	sp|Q12931|TRAP1_HUMAN;tr|I3L0K7|I3L0K7_HUMAN	323,31	363390000	53	14	0,50407184	-0,114306927	24,5948	24,76233	24,5625	24,89458	24,67131	24,39833	24,33559	24,95176
sp|P48643|TCPE_HUMAN;tr|E7ENZ3|E7ENZ3_HUMAN;tr|B7ZAR1|B7ZAR1_HUMAN;tr|E9PCA1|	sp|P48643|TCPE_HUMAN;tr|E7ENZ3|E7ENZ3_HUMAN;tr|B7ZAR1|B7ZAR1_HUMAN;tr|E9PCA1|	323,31	535120000	64	18	0,50731832	-0,077983856	24,79516	25,16434	25,22733	24,9389	24,88106	25,07985	24,88297	24,96992
tr|E9PMI6|E9PMI6_HUMAN;tr|E9PJF4|E9PJF4_HUMAN;tr|J3KN38|J3KN38_HUMAN;sp|P54105	tr|E9PMI6|E9PMI6_HUMAN;tr|E9PJF4|E9PJF4_HUMAN;tr|J3KN38|J3KN38_HUMAN;sp|P54105	7,8106	14464000	3	2	0,50795326	-0,086969058	20,06592	19,98968	20,03778	20,17487	20,03282	19,72457	NaN	20,18289
sp|Q8N1G4|LRC47_HUMAN	sp|Q8N1G4|LRC47_HUMAN	11,285	16448000	10	3	0,5163547	-0,195269585	NaN	20,19508	20,13132	20,46314	20,39467	19,77054	19,59858	20,50785
tr|A0A0D9SFB3|A0A0D9SFB3_HUMAN;tr|A0A0D9SG12|A0A0D9SG12_HUMAN;sp|O00571|DDX	tr|A0A0D9SFB3|A0A0D9SFB3_HUMAN;tr|A0A0D9SG12|A0A0D9SG12_HUMAN;sp|O00571|DDX	50,184	171150000	45	12	0,52058551	-0,111611366	23,29908	23,37782	23,76739	23,37796	22,97179	23,5093	23,43695	23,45776
sp|P68871|HBB_HUMAN;tr|F8W6P5|F8W6P5_HUMAN;sp|P02042|HBD_HUMAN;tr|E9PFT6|E9	sp|P68871|HBB_HUMAN;tr|F8W6P5|F8W6P5_HUMAN;sp|P02042|HBD_HUMAN;tr|E9PFT6|E9	233,61	305450000	35	6	0,52385666	-0,534651279	24,73738	24,35964	23,56221	25,00894	25,79434	23,59218	23,86231	22,28074
sp|Q15155|NOMO1_HUMAN;sp|P69849|NOMO3_HUMAN;tr|J3KN36|J3KN36_HUMAN;tr|A0A(	sp|Q15155|NOMO1_HUMAN;sp|P69849|NOMO3_HUMAN;tr|J3KN36|J3KN36_HUMAN;tr|A0A(	9,3513	17500000	7	4	0,52461204	-0,069998741	20,36378	20,36965	20,22294	20,06342	20,10546	20,01781	20,28048	20,33604
sp|P05388|RLA0_HUMAN;tr|F8VWS0|F8VWS0_HUMAN;tr|F8VU65|F8VU65_HUMAN;tr|F8VPE8	sp|P05388|RLA0_HUMAN;tr|F8VWS0|F8VWS0_HUMAN;tr|F8VU65|F8VU65_HUMAN;tr|F8VPE8	286,75	771760000	48	11	0,52534426	-0,136325359	25,74319	25,64576	25,87044	25,3652	25,26137	25,76162	25,86512	25,19118
sp|P53597|SUCA_HUMAN	sp|P53597|SUCA_HUMAN	3,7078	22886000	5	2	0,5293824	0,101902485	20,38068	20,39142	20,40873	20,66484	20,59268	20,93869	20,34397	20,37793
sp|Q13895|BYST_HUMAN;tr|H7BY94|H7BY94_HUMAN	sp|Q13895|BYST_HUMAN;tr|H7BY94|H7BY94_HUMAN	2,4791	7576200	3	2	0,53031353	0,095202446	18,88762	18,85538	18,88908	18,877	18,87658	18,65522	19,33632	19,02177
sp|P25788|PSA3_HUMAN;tr|G3V3W4|G3V3W4_HUMAN;tr|G3V5N4|G3V5N4_HUMAN;tr|G3V4	sp|P25788|PSA3_HUMAN	58,925	81110000	19	6	0,53063341	-0,236149311	22,50942	22,54463	22,97699	21,828	22,93135	22,33576	21,78676	21,86056
sp|Q14566|MCM6_HUMAN	sp|Q14566|MCM6_HUMAN	46,097	117230000	28	12	0,53363054	-0,143053532	23,20766	22,72507	23,14782	22,99519	22,68132	22,58072	22,82128	23,42021
sp|P06748|NPM_HUMAN;tr|E5RGW4|E5RGW4_HUMAN;tr|E5RI98|E5RI98_HUMAN	sp|P06748|NPM_HUMAN;tr|E5RGW4|E5RGW4_HUMAN	323,31	1460600000	23	4	0,53485278	-0,191154003	26,06614	26,58492	26,99432	26,54963	25,96193	26,6983	26,7758	25,99436
sp|Q7L2H7|EIF3M_HUMAN;tr|J3KNJ2|J3KNJ2_HUMAN;tr|H0YCQ8|H0YCQ8_HUMAN;tr|E9PRI2|	sp|Q7L2H7|EIF3M_HUMAN;tr|J3KNJ2|J3KNJ2_HUMAN;tr|H0YCQ8|H0YCQ8_HUMAN	128,5	16726000	11	4	0,54386547	-0,185985088	19,77624	NaN	20,70658	20,28816	20,11273	20,09623	19,67623	20,39885
tr|E9PKG1|E9PKG1_HUMAN;sp|Q99873|ANM1_HUMAN;tr|H7C2I1|H7C2I1_HUMAN;tr|A0A087	tr|E9PKG1|E9PKG1_HUMAN;sp|Q99873|ANM1_HUMAN;tr|H7C2I1|H7C2I1_HUMAN;tr|A0A087	13,77	41681000	9	4	0,54479822	0,294005076	21,37978	NaN	20,88904	21,48286	20,92158	22,30541	21,40671	NaN
sp|P19338|NUCL_HUMAN;tr|H7BY16|H7BY16_HUMAN;tr|C9JYW2|C9JYW2_HUMAN;tr|C9JLB1	sp|P19338|NUCL_HUMAN;tr|H7BY16|H7BY16_HUMAN	263,48	2701500000	82	24	0,55281107	0,11093235	27,48631	27,34942	27,61224	27,09356	27,15692	27,39034	27,72592	27,71208
tr|G8JLD5|G8JLD5_HUMAN;sp|O00429|DNM1L_HUMAN;tr|F8VZ52|F8VZ52_HUMAN	tr|G8JLD5|G8JLD5_HUMAN;sp|O00429|DNM1L_HUMAN	2,6087	8784800	5	3	0,55399981	0,063127995	19,16624	19,19371	19,17553	18,98618	19,04484	19,28134	19,04793	19,40006
sp|O60264|SMCA5_HUMAN;sp|P28370|SMCA1_HUMAN;tr|A0A0A0MRP6|A0A0A0MRP6_HUM	sp|O60264|SMCA5_HUMAN	10,333	31764000	10	5	0,56334172	0,223392963	20,31661	20,34093	20,97673	20,7615	20,26178	20,72517	21,76211	20,54028
tr|H9KV75|H9KV75_HUMAN;sp|P12814|ACTN1_HUMAN;tr|H0YJ11|H0YJ11_HUMAN;tr|H0YJW	tr|H9KV75|H9KV75_HUMAN;sp|P12814|ACTN1_HUMAN;tr|H0YJ11|H0YJ11_HUMAN;tr|H0YJW	2,7192	10236000	3	2	0,57153878	-0,114747047	19,75344	18,9725	19,69542	19,61597	19,38821	19,38924	19,2463	19,55461
sp|P62333|PRS10_HUMAN;tr|A0A087X2I1|A0A087X2I1_HUMAN;tr|H0YJC0|H0YJC0_HUMAN;tr	sp|P62333|PRS10_HUMAN;tr|A0A087X2I1|A0A087X2I1_HUMAN;tr|H0YJC0|H0YJC0_HUMAN;tr	19,546	90567000	24	8	0,57154257	0,069863319	22,239	22,52931	22,38118	22,58095	22,72038	22,55258	22,42437	22,31257
sp|P62913|RL11_HUMAN;tr|Q5VVC8|Q5VVC8_HUMAN;tr|Q5VVC9|Q5VVC9_HUMAN	sp|P62913|RL11_HUMAN;tr|Q5VVC8|Q5VVC8_HUMAN	96,374	202950000	20	6	0,57330551	0,321595669	23,38139	23,40393	23,46239	23,31907	23,02284	24,92619	24,29039	22,61374
tr|A0A087WUL2|A0A087WUL2_HUMAN;sp|P49720|PSB3_HUMAN;tr|A0A087WXQ8|A0A087W	tr|A0A087WUL2|A0A087WUL2_HUMAN;sp|P49720|PSB3_HUMAN;tr|A0A087WXQ8|A0A087W	2,7611	31223000	6	2	0,5770458	0,177607536	21,16993	20,07483	21,34234	21,0654	21,07457	21,25753	20,8233	21,20753
sp|P35580|MYH10_HUMAN	sp|P35580|MYH10_HUMAN	24,004	28565000	11	7	0,57914482	-0,198667526	21,33604	21,12742	21,23298	20,6486	20,3393	20,3931	21,48822	21,32975
sp|Q00610|CLH1_HUMAN;tr|A0A087WVQ6|A0A087WVQ6_HUMAN;tr|J3KS13|J3KS13_HUMAN	sp|Q00610|CLH1_HUMAN;tr|A0A087WVQ6|A0A087WVQ6_HUMAN	217,92	353700000	86	28	0,5825193	-0,109719753	24,7113	24,60665	24,68362	24,15004	24,12747	24,26899	24,64669	24,66958
sp|Q9Y3I0|RTCB_HUMAN	sp|Q9Y3I0|RTCB_HUMAN	18,11	54825000	11	5	0,58395948	-0,235686779	21,50586	21,64878	21,63768	22,49629	20,5861	22,07874	21,86189	21,81913
sp|Q15717|ELAV1_HUMAN;tr|M0QZR9|M0QZR9_HUMAN;tr|M0R055|M0R055_HUMAN	sp|Q15717|ELAV1_HUMAN	10,866	65001000	18	7	0,58610058	0,228929996	21,61216	21,33685	22,04217	21,93991	21,10661	22,16162	22,83954	21,73905
tr|A0A0U1RQF0|A0A0U1RQF0_HUMAN;sp|P49327|FAS_HUMAN;tr|A0A0U1RRG3|A0A0U1RRG	tr|A0A0U1RQF0|A0A0U1RQF0_HUMAN;sp|P49327|FAS_HUMAN	323,31	858820000	164	55	0,59260778	-0,108879566	25,91593	26,06437	25,75659	25,59477	25,27753	25,68715	25,91472	26,01674
sp|P15924|DESP_HUMAN	sp|P15924|DESP_HUMAN	84,401	212490000	66	29	0,59430773	-0,146747589	24,03507	24,23936	23,09713	23,81763	23,67982	23,52671	23,8624	23,53326
sp|P62263|RS14_HUMAN;tr|H0YB22|H0YB22_HUMAN;tr|E5RH77|E5RH77_HUMAN	sp|P62263|RS14_HUMAN;tr|H0YB22|H0YB22_HUMAN;tr|E5RH77|E5RH77_HUMAN	80,38	263380000	12	5	0,5993745	-0,506389618	NaN	23,71931	25,11831	23,40276	24,06077	23,64372	21,72451	24,86594
sp|O60506|HNRPQ_HUMAN;tr|B7Z645|B7Z645_HUMAN;tr|F6UXX1|F6UXX1_HUMAN	sp|O60506|HNRPQ_HUMAN;tr|B7Z645|B7Z645_HUMAN	94,154	383690000	50	11	0,60183853	-0,11222744	24,74566	24,43352	24,78561	24,38482	24,02732	24,67314	24,82221	24,37802
sp|P78371|TCPB_HUMAN;tr|F5GWF6|F5GWF6_HUMAN;tr|F8VQ14|F8VQ14_HUMAN	sp|P78371|TCPB_HUMAN;tr|F5GWF6|F5GWF6_HUMAN;tr|F8VQ14|F8VQ14_HUMAN	255,23	634870000	77	21	0,6030227	-0,079109669	25,17615	25,41351	25,4204	25,38752	24,99278	25,3669	25,13038	25,59109
sp|O75643|U520_HUMAN;tr|B4E0P5|B4E0P5_HUMAN	sp|O75643|U520_HUMAN	142,26	151800000	48	22	0,61001557	0,134519577	23,27185	22,85177	22,95463	23,04382	22,49211	23,26069	23,56732	23,34001
sp|P48147|PPCE_HUMAN	sp|P48147|PPCE_HUMAN	4,6291	10131000	10	3	0,61255191	0,069408894	19,12116	19,35518	19,36765	19,54202	19,43335	19,29167	19,25509	19,68353
sp|O94925|GLSK_HUMAN;tr|H7BZD1|H7BZD1_HUMAN;tr|C9JIJ6|C9JIJ6_HUMAN	sp|O94925|GLSK_HUMAN	3,2434	31983000	3	2	0,61486616	-0,150286674	20,92738	21,15875	21,08719	21,28963	21,16454	21,48822	21,00546	20,20359
sp|Q13049|TRI32_HUMAN;tr|Q5JVY0|Q5JVY0_HUMAN	sp|Q13049|TRI32_HUMAN	34,444	125270000	25	13	0,61517467	0,416841507	21,0901	22,98141	21,73802	24,16131	23,98224	22,16383	22,97418	22,51795
sp|Q9P035|HACD3_HUMAN;tr|H3BPZ1|H3BPZ1_HUMAN;tr|H3BS72|H3BS72_HUMAN;tr|H3BM	sp|Q9P035|HACD3_HUMAN;tr|H3BPZ1|H3BPZ1_HUMAN;tr|H3BS72|H3BS72_HUMAN;tr|H3BM	44,752	37970000	13	4	0,62031208	-0,058093389	21,38052	21,65403	21,58449	21,45543	21,33631	21,39032	21,65495	NaN
sp|Q92900|RENT1_HUMAN	sp|Q92900|RENT1_HUMAN	17,222	55507000	23	9	0,62665175	0,091144085	21,94174	21,96526	21,56132	21,79951	21,93146	21,53089	21,89667	22,27338
sp|P62906|RL10A_HUMAN	sp|P62906|RL10A_HUMAN	34,061	592450000	26	8	0,6307285	0,154499054	24,3835	24,90834	25,48299	25,44525	24,73753	25,27508	25,41542	25,41006
sp|P08133|ANXA6_HUMAN;tr|E5RK69|E5RK69_HUMAN;tr|E7EMC6|E7EMC6_HUMAN;tr|H0YC	sp|P08133|ANXA6_HUMAN;tr|E5RK69|E5RK69_HUMAN;tr|E7EMC6|E7EMC6_HUMAN	8,5505	35884000	10	6	0,63696423	-0,096975327	21,12534	21,30145	20,88017	21,69477	20,99252	21,13239	21,0692	21,41972
sp|P62277|RS13_HUMAN;tr|J3KMX5|J3KMX5_HUMAN;tr|E9PS50|E9PS50_HUMAN	sp|P62277|RS13_HUMAN;tr|J3KMX5|J3KMX5_HUMAN	72,03	185700000	20	6	0,63750698	0,169817448	23,81176	22,67816	23,8944	23,1897	23,96622	23,49692	23,07574	23,7144
sp|Q9BY77|PDIP3_HUMAN;tr|F6VRR5|F6VRR5_HUMAN;tr|Q8WUT1|Q8WUT1_HUMAN;tr|F8W	sp|Q9BY77|PDIP3_HUMAN;tr|F6VRR5|F6VRR5_HUMAN;tr|Q8WUT1|Q8WUT1_HUMAN;tr|F8W	4,4483	12174000	5	3	0,63802592	-0,096707185	NaN	19,67908	19,75739	19,69147	19,88927	19,21516	19,47965	19,86635
tr|E9PBS1|E9PBS1_HUMAN;sp|P22234|PUR6_HUMAN;tr|D6RF62|D6RF62_HUMAN	tr|E9PBS1|E9PBS1_HUMAN;sp|P22234|PUR6_HUMAN;tr|D6RF62|D6RF62_HUMAN	66,062	271020000	31	10	0,6414089	-0,143871784	24,21801	23,3652	24,38936	24,55625	23,79838	24,10957	23,75908	24,2863
sp|P49368|TCPG_HUMAN;tr|B4DUR8|B4DUR8_HUMAN;tr|E9PRC8|E9PRC8_HUMAN;tr|Q5SZX6	sp|P49368|TCPG_HUMAN;tr|B4DUR8|B4DUR8_HUMAN	62,936	582630000	60	15	0,64324817	0,06640625	25,04085	24,98272	25,40682	25,23301	25,26417	25,09684	25,07631	25,4917
sp|P07737|PROF1_HUMAN;tr|K7EJ44|K7EJ44_HUMAN;CON__P02584;tr|I3L3D5|I3L3D5_HUMA	sp|P07737|PROF1_HUMAN;tr|K7EJ44|K7EJ44_HUMAN	64,41	301980000	26	6	0,64377029	-0,180504799	24,94033	23,80882	23,88157	24,15522	24,11601	24,05001	23,30619	24,59172
tr|H7BXY3|H7BXY3_HUMAN;sp|Q7L2E3|DHX30_HUMAN	tr|H7BXY3|H7BXY3_HUMAN;sp|Q7L2E3|DHX30_HUMAN	28,381	55371000	19	12	0,64430982	0,149693012	21,40447	21,46503	22,0192	21,39383	21,146	21,94167	22,35877	21,43487
sp|Q9UBB4|ATX10_HUMAN;tr|B1AHE3|B1AHE3_HUMAN;tr|B1AHE4|B1AHE4_HUMAN	sp|Q9UBB4|ATX10_HUMAN	5,8452	11836000	9	3	0,64563632	0,144845486	19,26061	19,38275	19,60357	19,72106	19,54836	19,48235	19,0936	20,42306
tr|A0A024RA52|A0A024RA52_HUMAN;sp|P25787|PSA2_HUMAN;tr|H3BT36|H3BT36_HUMAN;	tr|A0A024RA52|A0A024RA52_HUMAN;sp|P25787|PSA2_HUMAN;tr|H3BT36|H3BT36_HUMAN	21,048	34895000	14	4	0,64604543	-0,111501217	21,25194	20,92904	21,31567	20,91539	21,06789	20,88881	20,51007	21,49926
sp|O00264|PGRC1_HUMAN;tr|U3KQM0|U3KQM0_HUMAN	sp|O00264|PGRC1_HUMAN	9,2433	135690000	13	5	0,64612085	-0,100840092	22,8547	22,71389	23,49277	23,20262	23,12704	23,09553	23,00263	22,63542
sp|Q14978|NOLC1_HUMAN;tr|A0A0A0MRM9|A0A0A0MRM9_HUMAN;tr|S4R349|S4R349_HUN	sp|Q14978|NOLC1_HUMAN;tr|A0A0A0MRM9|A0A0A0MRM9_HUMAN;tr|S4R349|S4R349_HUN	24,632	54395000	8	6	0,64745269	0,138813496	21,8665	21,56384	22,21848	21,4708	21,38401	22,14762	22,44017	21,70307
tr|A0A024R4E5|A0A024R4E5_HUMAN;sp|Q00341|VIGLN_HUMAN;tr|H0Y394|H0Y394_HUMAN	tr|A0A024R4E5|A0A024R4E5_HUMAN;sp|Q00341|VIGLN_HUMAN;tr|H0Y394|H0Y394_HUMAN	7,6908	39687000	10	7	0,65257105	-0,056925297	21,50096	21,34657	21,41617	21,22058	21,14824	21,23637	21,25159	21,62037
sp|Q9H444|CHM4B_HUMAN	sp|Q9H444|CHM4B_HUMAN	127,67	36889000	8	4	0,65735292	0,462702274	20,01332	20,31352	20,72376	20,23806	23,71534	19,95077	19,55822	19,91513
tr|Q5T3Q7|Q5T3Q7_HUMAN;sp|Q9H583|HEAT1_HUMAN	tr|Q5T3Q7|Q5T3Q7_HUMAN;sp|Q9H583|HEAT1_HUMAN	11,601	13914000	7	6	0,65922615	-0,076600869	19,84592	19,91139	19,85271	19,91069	19,44164	19,86427	20,10482	NaN
tr|J3KTF8|J3KTF8_HUMAN;sp|P52565|GDIR1_HUMAN;tr|J3QQX2|J3QQX2_HUMAN;tr|J3KRE2|	tr|J3KTF8|J3KTF8_HUMAN;sp|P52565|GDIR1_HUMAN;tr|J3QQX2|J3QQX2_HUMAN;tr|J3KRE2|	24,262	62291000	12	4	0,65950559	0,050187588	21,79229	21,91747	21,9668	22,11737	22,16303	22,07682	21,76765	21,98718
tr|A0A087WV29|A0A087WV29_HUMAN;sp|Q9H0A0|NAT10_HUMAN	tr|A0A087WV29|A0A087WV29_HUMAN;sp|Q9H0A0|NAT10_HUMAN	8,2658	12076000	6	4	0,66155752	0,124891122	NaN	19,5441	19,60634	19,25306	19,23678	19,48613	20,21288	19,4351
sp|P51659|DHB4_HUMAN;tr|E7ER27|E7ER27_HUMAN;tr|E7EWE5|E7EWE5_HUMAN;tr|G5E9S2	sp|P51659|DHB4_HUMAN;tr|E7ER27|E7ER27_HUMAN;tr|E7EWE5|E7EWE5_HUMAN	71,401	160410000	33	11	0,66186585	-0,096321583	23,25782	23,40834	23,46463	23,70356	23,34706	23,23301	22,9893	23,8797
sp|P37802|TAGL2_HUMAN;tr|X6RJP6|X6RJP6_HUMAN;tr|C9J5W6|C9J5W6_HUMAN;sp|Q9UI15	sp|P37802|TAGL2_HUMAN;tr|X6RJP6|X6RJP6_HUMAN	175,57	320260000	35	7	0,66200617	-0,106769085	24,17333	24,46631	24,45174	24,64642	24,46039	24,54699	23,70134	24,60201
sp|Q9Y262|EIF3L_HUMAN;tr|B0QY89|B0QY89_HUMAN;tr|B0QY90|B0QY90_HUMAN;tr|C9JHP4	sp|Q9Y262|EIF3L_HUMAN;tr|B0QY89|B0QY89_HUMAN;tr|B0QY90|B0QY90_HUMAN	27,943	173900000	42	13	0,66340845	-0,065304279	23,63733	23,47222	23,55415	23,25184	23,17048	23,40743	23,34854	23,72786
sp|Q08945|SSRP1_HUMAN;tr|E9PMD4|E9PMD4_HUMAN;tr|A0A0U1RRK2|A0A0U1RRK2_HUM	sp|Q08945|SSRP1_HUMAN	94,317	103260000	18	10	0,66406505	0,194560528	22,29496	22,16999	22,58155	22,80853	22,0255	22,30922	23,83026	22,46829
tr|E9PDE8|E9PDE8_HUMAN;sp|O95757|HS74L_HUMAN;tr|D6RJ96|D6RJ96_HUMAN	tr|E9PDE8|E9PDE8_HUMAN;sp|O95757|HS74L_HUMAN;tr|D6RJ96|D6RJ96_HUMAN	14,458	33283000	16	8	0,66568268	0,100984097	21,17487	21,15072	20,89726	21,07764	21,17432	20,97386	20,78604	21,7702
sp|P62851|RS25_HUMAN	sp|P62851|RS25_HUMAN	24,245	454900000	14	4	0,66828124	-0,099715233	24,53291	25,04623	24,91857	24,7348	24,88008	25,14344	24,53843	24,2717
tr|F8VYE8|F8VYE8_HUMAN;sp|P36873|PP1G_HUMAN;tr|F8W0W8|F8W0W8_HUMAN;tr|F8VR	tr|F8VYE8|F8VYE8_HUMAN;sp|P36873|PP1G_HUMAN;tr|F8W0W8|F8W0W8_HUMAN;tr|F8VR	70,494	71259000	31	1	0,67178726	0,077483177	21,97876	21,9827	22,3747	22,09313	22,17536	22,49512	22,27736	21,79138
tr|I3L1L3|I3L1L3_HUMAN;sp|Q9BQG0|MBB1A_HUMAN;tr|I3L2H8|I3L2H8_HUMAN	tr|I3L1L3|I3L1L3_HUMAN;sp|Q9BQG0|MBB1A_HUMAN	23,601	52376000	14	13	0,67494626	-0,130426884	21,81014	21,81577	21,92673	21,57565	21,10021	21,46827	22,45408	21,58403
sp|Q07955|SRSF1_HUMAN;tr|J3KTL2|J3KTL2_HUMAN;tr|J3QQV5|J3QQV5_HUMAN;tr|J3KSW7|	sp|Q07955|SRSF1_HUMAN;tr|J3KTL2|J3KTL2_HUMAN	44,686	118180000	22	9	0,67582443	-0,079689503	22,80802	22,61113	23,00122	23,22162	22,55112	22,68607	23,02744	23,05859
sp|P51149|RAB7A_HUMAN;tr|C9J8S3|C9J8S3_HUMAN;tr|C9J4V0|C9J4V0_HUMAN;tr|C9J4S4|C	sp|P51149|RAB7A_HUMAN;tr|C9J8S3|C9J8S3_HUMAN;tr|C9J4V0|C9J4V0_HUMAN;tr|C9J4S4|C	52,424	317310000	30	9	0,67634331	0,11392355	23,625	24,3532	24,21712	24,40633	24,41654	24,72265	23,91971	23,99843
sp|P22626|ROA2_HUMAN;tr|A0A087WUI2|A0A087WUI2_HUMAN	sp|P22626|ROA2_HUMAN;tr|A0A087WUI2|A0A087WUI2_HUMAN	316,15	660500000	58	12	0,68207698	0,143569946	25,15815	24,64339	25,51947	25,34973	24,71639	25,53769	25,96005	25,03091
tr|J3KR24|J3KR24_HUMAN;sp|P41252|SYIC_HUMAN;tr|A0A0A0MSX9|A0A0A0MSX9_HUMAN;t	tr|J3KR24|J3KR24_HUMAN;sp|P41252|SYIC_HUMAN;tr|A0A0A0MSX9|A0A0A0MSX9_HUMAN	21,02	143770000	31	12	0,68784522	-0,056794167	23,44771	23,44329	23,16054	23,05565	23,09719	23,03428	23,39663	23,35192

<table>
<tr><td>sp|Q8IZL8|PELP1_HUMAN;tr|C9JFV4|C9JFV4_HUMAN;tr|I3L445|I3L445_HUMAN;tr|E7EV54|E7</td><td>sp|Q8IZL8|PELP1_HUMAN;tr|C9JFV4|C9JFV4_HUMAN;tr|I3L445|I3L445_HUMAN;tr|E7EV54|E7</td><td>9,3914</td><td>26544000</td><td>10</td><td>6</td><td>0,69255071</td><td>0,121062279</td><td>20,64746</td><td>20,46702</td><td>20,46374</td><td>20,37263</td><td>19,9894</td><td>20,70269</td><td>21,34624</td><td>20,39676</td></tr>
<tr><td>sp|Q8TEX9|IPO4_HUMAN;tr|H0YN14|H0YN14_HUMAN;tr|H0YMR4|H0YMR4_HUMAN;tr|H0YL</td><td>sp|Q8TEX9|IPO4_HUMAN;tr|H0YN14|H0YN14_HUMAN</td><td>26,08</td><td>74376000</td><td>24</td><td>10</td><td>0,69419323</td><td>-0,067342758</td><td>22,22885</td><td>22,32743</td><td>22,24589</td><td>22,40395</td><td>21,98995</td><td>22,29561</td><td>21,994</td><td>22,65718</td></tr>
<tr><td>sp|P62280|RS11_HUMAN;tr|M0QZC5|M0QZC5_HUMAN;tr|M0R1H5|M0R1H5_HUMAN;tr|M0R</td><td>sp|P62280|RS11_HUMAN;tr|M0QZC5|M0QZC5_HUMAN</td><td>69,912</td><td>664810000</td><td>37</td><td>14</td><td>0,69939545</td><td>-0,074040413</td><td>25,43396</td><td>25,89412</td><td>25,56485</td><td>25,08371</td><td>25,45779</td><td>25,43094</td><td>25,22133</td><td>25,57043</td></tr>
<tr><td>sp|P61619|S61A1_HUMAN;tr|B4DR61|B4DR61_HUMAN;tr|H7C1Q9|H7C1Q9_HUMAN;tr|C9JXC</td><td>sp|P61619|S61A1_HUMAN;tr|B4DR61|B4DR61_HUMAN</td><td>74,553</td><td>35289000</td><td>9</td><td>3</td><td>0,70517278</td><td>0,15238142</td><td>20,42777</td><td>21,77133</td><td>21,06763</td><td>20,94149</td><td>20,93948</td><td>21,24632</td><td>21,92933</td><td>20,70261</td></tr>
<tr><td>sp|Q01581|HMCS1_HUMAN;tr|D6RIW1|D6RIW1_HUMAN</td><td>sp|Q01581|HMCS1_HUMAN</td><td>9,5958</td><td>27829000</td><td>3</td><td>4</td><td>0,70797968</td><td>-0,046669483</td><td>20,50659</td><td>20,96176</td><td>20,78101</td><td>20,79685</td><td>20,6677</td><td>20,92289</td><td>20,6802</td><td>20,58875</td></tr>
<tr><td>tr|G3V4W0|G3V4W0_HUMAN;tr|B4DY08|B4DY08_HUMAN;tr|B2R5W2|B2R5W2_HUMAN;tr|G</td><td>tr|G3V4W0|G3V4W0_HUMAN;tr|B4DY08|B4DY08_HUMAN;tr|B2R5W2|B2R5W2_HUMAN;tr|G</td><td>84,011</td><td>580710000</td><td>35</td><td>12</td><td>0,70813194</td><td>0,195635319</td><td>24,659</td><td>24,20672</td><td>25,3017</td><td>25,08618</td><td>23,90454</td><td>25,31721</td><td>25,97373</td><td>24,84067</td></tr>
<tr><td>tr|E9PEX6|E9PEX6_HUMAN;sp|P09622|DLDH_HUMAN</td><td>tr|E9PEX6|E9PEX6_HUMAN;sp|P09622|DLDH_HUMAN</td><td>29,249</td><td>88409000</td><td>20</td><td>6</td><td>0,70950166</td><td>0,04832077</td><td>22,30324</td><td>22,16898</td><td>22,40691</td><td>22,58857</td><td>22,42206</td><td>22,57013</td><td>22,49636</td><td>22,17243</td></tr>
<tr><td>sp|Q8TDN6|BRX1_HUMAN</td><td>sp|Q8TDN6|BRX1_HUMAN</td><td>2,9518</td><td>9222900</td><td>4</td><td>2</td><td>0,71056674</td><td>-0,119003455</td><td>19,51138</td><td>19,21966</td><td>19,57941</td><td>19,252</td><td>18,74678</td><td>19,16362</td><td>19,90442</td><td>NaN</td></tr>
<tr><td>sp|Q13011|ECH1_HUMAN;tr|M0QZW4|M0QZW4_HUMAN;tr|M0R248|M0R248_HUMAN</td><td>sp|Q13011|ECH1_HUMAN;tr|M0QZW4|M0QZW4_HUMAN;tr|M0R248|M0R248_HUMAN</td><td>2,6158</td><td>12539000</td><td>5</td><td>2</td><td>0,71409183</td><td>-0,048322201</td><td>19,81397</td><td>19,73587</td><td>19,68274</td><td>19,93431</td><td>19,84382</td><td>19,58401</td><td>19,53123</td><td>20,01454</td></tr>
<tr><td>sp|P61006|RAB8A_HUMAN</td><td>sp|P61006|RAB8A_HUMAN</td><td>30,677</td><td>351160000</td><td>26</td><td>5</td><td>0,71454235</td><td>-0,117963314</td><td>23,95952</td><td>24,54904</td><td>24,47091</td><td>24,67761</td><td>24,1715</td><td>25,06336</td><td>24,09233</td><td>23,85804</td></tr>
<tr><td>sp|P23381|SYWC_HUMAN;tr|H0YJP3|H0YJP3_HUMAN;tr|G3V3H8|G3V3H8_HUMAN;tr|G3V3Y5</td><td>sp|P23381|SYWC_HUMAN;tr|H0YJP3|H0YJP3_HUMAN;tr|G3V3H8|G3V3H8_HUMAN;tr|G3V3Y5</td><td>4,4775</td><td>22479000</td><td>7</td><td>4</td><td>0,7155321</td><td>-0,06584549</td><td>20,41318</td><td>20,55805</td><td>20,76077</td><td>20,75369</td><td>20,24505</td><td>20,60892</td><td>NaN</td><td>20,81276</td></tr>
<tr><td>sp|P42167|LAP2B_HUMAN;tr|G5E972|G5E972_HUMAN;tr|H0YJH7|H0YJH7_HUMAN</td><td>sp|P42167|LAP2B_HUMAN;tr|G5E972|G5E972_HUMAN</td><td>13,445</td><td>61568000</td><td>13</td><td>5</td><td>0,71615763</td><td>0,184816837</td><td>21,29317</td><td>21,50722</td><td>22,25263</td><td>22,39132</td><td>21,30647</td><td>22,04537</td><td>22,78586</td><td>NaN</td></tr>
<tr><td>sp|O43143|DHX15_HUMAN</td><td>sp|O43143|DHX15_HUMAN</td><td>31,012</td><td>254590000</td><td>35</td><td>12</td><td>0,71781215</td><td>0,046163082</td><td>24,0258</td><td>23,98815</td><td>24,23067</td><td>23,72151</td><td>24,10534</td><td>23,91816</td><td>23,94824</td><td>24,17904</td></tr>
<tr><td>sp|P11021|GRP78_HUMAN</td><td>sp|P11021|GRP78_HUMAN</td><td>323,31</td><td>931180000</td><td>99</td><td>23</td><td>0,71917747</td><td>0,017924309</td><td>25,79875</td><td>25,87572</td><td>25,94592</td><td>25,9844</td><td>25,89696</td><td>25,99162</td><td>25,89904</td><td>25,88888</td></tr>
<tr><td>sp|O00231|PSD11_HUMAN;tr|J3QRY4|J3QRY4_HUMAN</td><td>sp|O00231|PSD11_HUMAN</td><td>18,015</td><td>71450000</td><td>18</td><td>9</td><td>0,72645874</td><td>-0,047335148</td><td>22,3396</td><td>22,05577</td><td>22,19895</td><td>22,15962</td><td>22,1053</td><td>22,11245</td><td>21,89556</td><td>22,45129</td></tr>
<tr><td>sp|P22061|PIMT_HUMAN;tr|A0A0A0MRJ6|A0A0A0MRJ6_HUMAN;tr|H7BY58|H7BY58_HUMAN</td><td>sp|P22061|PIMT_HUMAN;tr|A0A0A0MRJ6|A0A0A0MRJ6_HUMAN;tr|H7BY58|H7BY58_HUMAN</td><td>35,367</td><td>204210000</td><td>31</td><td>5</td><td>0,72773243</td><td>-0,087629318</td><td>23,40717</td><td>23,93085</td><td>23,68239</td><td>23,86041</td><td>23,92443</td><td>23,72713</td><td>23,01512</td><td>23,86363</td></tr>
<tr><td>tr|V9H019|V9H019_HUMAN;tr|E9PHA6|E9PHA6_HUMAN;sp|P43246|MSH2_HUMAN;tr|A0A08</td><td>tr|V9H019|V9H019_HUMAN;tr|E9PHA6|E9PHA6_HUMAN;sp|P43246|MSH2_HUMAN;tr|A0A08</td><td>13,982</td><td>27855000</td><td>9</td><td>7</td><td>0,72823311</td><td>0,070614338</td><td>20,6778</td><td>20,71373</td><td>21,07352</td><td>20,91576</td><td>21,02039</td><td>20,66154</td><td>20,62872</td><td>21,35262</td></tr>
<tr><td>tr|A0A087WW66|A0A087WW66_HUMAN;sp|Q99460|PSMD1_HUMAN;tr|H7C378|H7C378_HU</td><td>tr|A0A087WW66|A0A087WW66_HUMAN;sp|Q99460|PSMD1_HUMAN</td><td>32,542</td><td>56395000</td><td>19</td><td>7</td><td>0,72948643</td><td>0,056792736</td><td>21,57473</td><td>21,78979</td><td>22,05364</td><td>21,89093</td><td>21,77884</td><td>21,6387</td><td>21,91528</td><td>22,20344</td></tr>
<tr><td>sp|P21333|FLNA_HUMAN;tr|Q5HY54|Q5HY54_HUMAN;tr|Q60FE5|Q60FE5_HUMAN;tr|A0A087</td><td>sp|P21333|FLNA_HUMAN;tr|Q5HY54|Q5HY54_HUMAN;tr|Q60FE5|Q60FE5_HUMAN;tr|A0A087</td><td>319,21</td><td>793130000</td><td>146</td><td>51</td><td>0,73288112</td><td>-0,060810089</td><td>25,7619</td><td>25,79694</td><td>25,66998</td><td>25,37657</td><td>25,26585</td><td>25,44922</td><td>25,78024</td><td>25,86684</td></tr>
<tr><td>sp|O14776|TCRG1_HUMAN;tr|G3V220|G3V220_HUMAN</td><td>sp|O14776|TCRG1_HUMAN;tr|G3V220|G3V220_HUMAN</td><td>16,974</td><td>40706000</td><td>13</td><td>6</td><td>0,74621884</td><td>-0,092879295</td><td>21,23719</td><td>21,45558</td><td>21,59478</td><td>21,24743</td><td>20,77523</td><td>21,1483</td><td>21,22712</td><td>22,01281</td></tr>
<tr><td>sp|Q13595|TRA2A_HUMAN</td><td>sp|Q13595|TRA2A_HUMAN</td><td>4,1706</td><td>9730200</td><td>8</td><td>2</td><td>0,75321208</td><td>0,130054474</td><td>18,88748</td><td>18,67252</td><td>19,75427</td><td>19,04014</td><td>18,57223</td><td>19,54518</td><td>19,94364</td><td>18,81359</td></tr>
<tr><td>sp|P54886|P5CS_HUMAN</td><td>sp|P54886|P5CS_HUMAN</td><td>210,62</td><td>261950000</td><td>44</td><td>15</td><td>0,76238981</td><td>-0,047669411</td><td>23,7102</td><td>24,32704</td><td>24,07969</td><td>24,13406</td><td>24,10205</td><td>23,86948</td><td>23,89892</td><td>24,18986</td></tr>
<tr><td>tr|F2Z2Q9|F2Z2Q9_HUMAN;tr|G5E9R5|G5E9R5_HUMAN;tr|D3YTI2|D3YTI2_HUMAN;sp|P2466</td><td>tr|F2Z2Q9|F2Z2Q9_HUMAN;tr|G5E9R5|G5E9R5_HUMAN;tr|D3YTI2|D3YTI2_HUMAN;sp|P2466</td><td>27,507</td><td>34651000</td><td>6</td><td>2</td><td>0,76321988</td><td>0,07427597</td><td>21,13752</td><td>20,82664</td><td>NaN</td><td>21,3899</td><td>NaN</td><td>21,27219</td><td>20,87897</td><td>21,42573</td></tr>
<tr><td>sp|Q8WXF1|PSPC1_HUMAN;tr|X6RDA4|X6RDA4_HUMAN</td><td>sp|Q8WXF1|PSPC1_HUMAN;tr|X6RDA4|X6RDA4_HUMAN</td><td>11,799</td><td>35457000</td><td>8</td><td>5</td><td>0,76513205</td><td>-0,096505006</td><td>21,46822</td><td>NaN</td><td>21,43354</td><td>21,1187</td><td>21,72213</td><td>21,14694</td><td>20,59632</td><td>21,5092</td></tr>
<tr><td>sp|P12081|SYHC_HUMAN;tr|B3KWE1|B3KWE1_HUMAN;tr|E7ETE2|E7ETE2_HUMAN;tr|B4E1C5</td><td>sp|P12081|SYHC_HUMAN;tr|B3KWE1|B3KWE1_HUMAN;tr|E7ETE2|E7ETE2_HUMAN;tr|B4E1C5</td><td>19,478</td><td>62223000</td><td>16</td><td>4</td><td>0,76820493</td><td>0,082029343</td><td>21,24278</td><td>22,38432</td><td>22,13887</td><td>22,12931</td><td>22,18211</td><td>21,88494</td><td>21,92249</td><td>22,23386</td></tr>
<tr><td>sp|Q06203|PUR1_HUMAN</td><td>sp|Q06203|PUR1_HUMAN</td><td>3,4701</td><td>26627000</td><td>8</td><td>3</td><td>0,77516584</td><td>-0,05828619</td><td>20,62774</td><td>NaN</td><td>20,78835</td><td>21,13602</td><td>20,98482</td><td>20,78875</td><td>20,44263</td><td>20,95347</td></tr>
<tr><td>sp|Q08211|DHX9_HUMAN</td><td>sp|Q08211|DHX9_HUMAN</td><td>323,31</td><td>640080000</td><td>62</td><td>24</td><td>0,77847202</td><td>-0,06515646</td><td>25,35525</td><td>25,29309</td><td>25,44291</td><td>25,12605</td><td>24,77294</td><td>25,15151</td><td>25,79556</td><td>25,23666</td></tr>
<tr><td>sp|P30153|2AAA_HUMAN;tr|B3KQV6|B3KQV6_HUMAN;tr|C9J9C1|C9J9C1_HUMAN;sp|P30154</td><td>sp|P30153|2AAA_HUMAN;tr|B3KQV6|B3KQV6_HUMAN</td><td>143,49</td><td>331410000</td><td>39</td><td>13</td><td>0,78038128</td><td>0,041053295</td><td>24,12849</td><td>24,30062</td><td>24,58606</td><td>24,46905</td><td>24,53843</td><td>24,41745</td><td>24,13006</td><td>24,5625</td></tr>
<tr><td>sp|P36871|PGM1_HUMAN</td><td>sp|P36871|PGM1_HUMAN</td><td>2,6432</td><td>4699300</td><td>2</td><td>2</td><td>0,78359171</td><td>0,04398044</td><td>NaN</td><td>18,34863</td><td>18,36098</td><td>18,23547</td><td>18,25179</td><td>18,57163</td><td>18,05574</td><td>18,55686</td></tr>
<tr><td>sp|Q15393|SF3B3_HUMAN;tr|J3QRB2|J3QRB2_HUMAN;tr|J3QL37|J3QL37_HUMAN;tr|H3BMB</td><td>sp|Q15393|SF3B3_HUMAN</td><td>50,35</td><td>216740000</td><td>45</td><td>16</td><td>0,788038</td><td>-0,034948826</td><td>23,80361</td><td>23,94306</td><td>23,95038</td><td>23,61953</td><td>23,62879</td><td>23,62333</td><td>23,95704</td><td>23,96763</td></tr>
<tr><td>sp|P46783|RS10_HUMAN;tr|F6U211|F6U211_HUMAN;tr|S4R435|S4R435_HUMAN;sp|Q9NQ39</td><td>sp|P46783|RS10_HUMAN;tr|F6U211|F6U211_HUMAN;tr|S4R435|S4R435_HUMAN;sp|Q9NQ39</td><td>123,61</td><td>455950000</td><td>31</td><td>6</td><td>0,79246301</td><td>-0,047572136</td><td>24,98316</td><td>24,55818</td><td>24,80302</td><td>24,78801</td><td>24,77997</td><td>25,13426</td><td>24,44828</td><td>24,57957</td></tr>
<tr><td>sp|Q16850|CP51A_HUMAN;tr|A0A0C4DFL7|A0A0C4DFL7_HUMAN;tr|H7C0D0|H7C0D0_HUMA</td><td>sp|Q16850|CP51A_HUMAN;tr|A0A0C4DFL7|A0A0C4DFL7_HUMAN</td><td>10,675</td><td>35096000</td><td>9</td><td>5</td><td>0,79258901</td><td>0,036378384</td><td>21,11184</td><td>21,11946</td><td>21,0283</td><td>21,33554</td><td>21,29821</td><td>20,99983</td><td>20,98815</td><td>21,45448</td></tr>
<tr><td>tr|B4DXW1|B4DXW1_HUMAN;sp|P61158|ARP3_HUMAN;tr|A0A0A0MTI9|A0A0A0MTI9_HUMA</td><td>tr|B4DXW1|B4DXW1_HUMAN;sp|P61158|ARP3_HUMAN</td><td>7,2066</td><td>64382000</td><td>11</td><td>6</td><td>0,79595837</td><td>-0,043965816</td><td>21,86737</td><td>22,0496</td><td>22,06487</td><td>22,19818</td><td>21,66027</td><td>22,28153</td><td>21,85041</td><td>22,21193</td></tr>
<tr><td>tr|A0A087WTT1|A0A087WTT1_HUMAN;sp|P11940|PABP1_HUMAN;tr|E7EQV3|E7EQV3_HUMA</td><td>tr|A0A087WTT1|A0A087WTT1_HUMAN;sp|P11940|PABP1_HUMAN;tr|E7EQV3|E7EQV3_HUMA</td><td>32,577</td><td>337340000</td><td>31</td><td>9</td><td>0,79768441</td><td>0,029750824</td><td>24,53611</td><td>24,30215</td><td>24,58663</td><td>24,24336</td><td>24,45406</td><td>24,25911</td><td>24,46625</td><td>24,60784</td></tr>
<tr><td>sp|P12270|TPR_HUMAN;tr|Q5SWX9|Q5SWX9_HUMAN</td><td>sp|P12270|TPR_HUMAN</td><td>41,646</td><td>84915000</td><td>24</td><td>16</td><td>0,80370712</td><td>-0,054824829</td><td>22,71257</td><td>22,15437</td><td>22,81925</td><td>22,15054</td><td>22,17228</td><td>22,25655</td><td>22,54675</td><td>22,64185</td></tr>
<tr><td>tr|A0A0J9YYL3|A0A0J9YYL3_HUMAN;tr|A0A0J9YXJ8|A0A0J9YXJ8_HUMAN;tr|A0A0J9YVP6|A0A0</td><td>tr|A0A0J9YYL3|A0A0J9YYL3_HUMAN;tr|A0A0J9YXJ8|A0A0J9YXJ8_HUMAN;tr|A0A0J9YVP6|A0A0</td><td>29,684</td><td>39842000</td><td>8</td><td>6</td><td>0,80485071</td><td>0,061664581</td><td>20,86627</td><td>21,32367</td><td>21,29109</td><td>21,60246</td><td>21,21193</td><td>21,0351</td><td>21,87094</td><td>21,21217</td></tr>
<tr><td>sp|Q00839|HNRPU_HUMAN;tr|Q5RI18|Q5RI18_HUMAN</td><td>sp|Q00839|HNRPU_HUMAN</td><td>323,31</td><td>1270600000</td><td>66</td><td>20</td><td>0,80538118</td><td>-0,072595596</td><td>26,28732</td><td>26,17356</td><td>26,50611</td><td>26,12118</td><td>25,59431</td><td>26,13792</td><td>26,90288</td><td>26,16267</td></tr>
<tr><td>tr|A0A0R4J2E8|A0A0R4J2E8_HUMAN;sp|P43243|MATR3_HUMAN;tr|A8MXP9|A8MXP9_HUMA</td><td>tr|A0A0R4J2E8|A0A0R4J2E8_HUMAN;sp|P43243|MATR3_HUMAN;tr|A8MXP9|A8MXP9_HUMA</td><td>44,336</td><td>90983000</td><td>18</td><td>10</td><td>0,80732898</td><td>0,090555191</td><td>22,18029</td><td>22,00607</td><td>22,66082</td><td>22,23491</td><td>21,66049</td><td>22,14942</td><td>23,22425</td><td>22,41015</td></tr>
<tr><td>sp|Q7Z2W4|ZCCHV_HUMAN;tr|C9J6P4|C9J6P4_HUMAN</td><td>sp|Q7Z2W4|ZCCHV_HUMAN;tr|C9J6P4|C9J6P4_HUMAN</td><td>10,784</td><td>19767000</td><td>5</td><td>4</td><td>0,80794505</td><td>-0,032900016</td><td>20,58105</td><td>NaN</td><td>20,26888</td><td>20,40291</td><td>20,38269</td><td>20,1437</td><td>20,55412</td><td>20,45834</td></tr>
<tr><td>tr|H3BT71|H3BT71_HUMAN;sp|P38159|RBMX_HUMAN;tr|H0Y6E7|H0Y6E7_HUMAN;tr|H3BR2</td><td>tr|H3BT71|H3BT71_HUMAN;sp|P38159|RBMX_HUMAN;tr|H0Y6E7|H0Y6E7_HUMAN;tr|H3BR2</td><td>39,792</td><td>173420000</td><td>24</td><td>6</td><td>0,80856229</td><td>0,130537987</td><td>22,84086</td><td>22,80166</td><td>23,56407</td><td>23,38311</td><td>22,23178</td><td>23,73624</td><td>24,3686</td><td>22,77523</td></tr>
<tr><td>sp|P08708|RS17_HUMAN;tr|H0YN88|H0YN88_HUMAN;tr|A0A075B716|A0A075B716_HUMAN;</td><td>sp|P08708|RS17_HUMAN;tr|H0YN88|H0YN88_HUMAN;tr|A0A075B716|A0A075B716_HUMAN;</td><td>10,278</td><td>125060000</td><td>12</td><td>4</td><td>0,81753439</td><td>-0,066148758</td><td>23,5518</td><td>22,56584</td><td>22,82939</td><td>22,92868</td><td>23,2387</td><td>23,16671</td><td>22,69732</td><td>22,50838</td></tr>
<tr><td>tr|B8ZZU8|B8ZZU8_HUMAN;sp|Q15370|ELOB_HUMAN;tr|I3L0M9|I3L0M9_HUMAN;tr|A0A0B4</td><td>tr|B8ZZU8|B8ZZU8_HUMAN;sp|Q15370|ELOB_HUMAN;tr|I3L0M9|I3L0M9_HUMAN</td><td>15,726</td><td>47016000</td><td>13</td><td>4</td><td>0,81953538</td><td>-0,08800602</td><td>21,15512</td><td>21,24951</td><td>21,84881</td><td>21,80715</td><td>21,60372</td><td>21,87222</td><td>21,75528</td><td>20,47734</td></tr>
<tr><td>sp|Q12906|ILF3_HUMAN;tr|K7EKJ9|K7EKJ9_HUMAN;tr|K7ER69|K7ER69_HUMAN;tr|K7EQR9|K</td><td>sp|Q12906|ILF3_HUMAN</td><td>89,617</td><td>363380000</td><td>43</td><td>14</td><td>0,82310683</td><td>-0,058572769</td><td>24,80843</td><td>24,30654</td><td>24,67352</td><td>24,18715</td><td>23,99723</td><td>24,34482</td><td>24,97767</td><td>24,42163</td></tr>
<tr><td>sp|P61586|RHOA_HUMAN;tr|C9JRM1|C9JRM1_HUMAN;tr|C9JNR4|C9JNR4_HUMAN;tr|C9JX21</td><td>sp|P61586|RHOA_HUMAN;tr|C9JRM1|C9JRM1_HUMAN;tr|C9JNR4|C9JNR4_HUMAN;tr|C9JX21</td><td>11,155</td><td>33889000</td><td>6</td><td>2</td><td>0,82664097</td><td>0,148293336</td><td>20,4958</td><td>NaN</td><td>20,56941</td><td>21,1061</td><td>20,36025</td><td>22,44905</td><td>20,36656</td><td>20,31241</td></tr>
<tr><td>tr|G5E9W3|G5E9W3_HUMAN;sp|Q9UKF6|CPSF3_HUMAN</td><td>tr|G5E9W3|G5E9W3_HUMAN;sp|Q9UKF6|CPSF3_HUMAN</td><td>1,8797</td><td>7718300</td><td>2</td><td>2</td><td>0,83098285</td><td>-0,026919683</td><td>19,28697</td><td>19,36818</td><td>NaN</td><td>19,1818</td><td>19,39452</td><td>19,3148</td><td>19,04687</td><td>NaN</td></tr>
<tr><td>tr|E5RI99|E5RI99_HUMAN;sp|P62888|RL30_HUMAN;tr|A0A0B4J213|A0A0B4J213_HUMAN;tr|E</td><td>tr|E5RI99|E5RI99_HUMAN;sp|P62888|RL30_HUMAN;tr|A0A0B4J213|A0A0B4J213_HUMAN</td><td>78,718</td><td>111850000</td><td>12</td><td>4</td><td>0,83256751</td><td>-0,037285328</td><td>22,69356</td><td>22,68942</td><td>23,11601</td><td>22,69136</td><td>22,56923</td><td>22,99319</td><td>22,97956</td><td>22,49924</td></tr>
<tr><td>sp|P08559|ODPA_HUMAN;tr|Q5JPU0|Q5JPU0_HUMAN;tr|Q5JPT9|Q5JPT9_HUMAN;tr|Q5JPU1</td><td>sp|P08559|ODPA_HUMAN;tr|Q5JPU0|Q5JPU0_HUMAN;tr|Q5JPT9|Q5JPT9_HUMAN;tr|Q5JPU1</td><td>8,0388</td><td>114450000</td><td>18</td><td>6</td><td>0,83442408</td><td>-0,031497955</td><td>22,64203</td><td>22,89698</td><td>22,79794</td><td>23,16129</td><td>23,0071</td><td>22,96608</td><td>22,80886</td><td>22,59021</td></tr>
<tr><td>tr|F2Z393|F2Z393_HUMAN;sp|P37837|TALDO_HUMAN</td><td>tr|F2Z393|F2Z393_HUMAN;sp|P37837|TALDO_HUMAN</td><td>16,319</td><td>169490000</td><td>14</td><td>5</td><td>0,83609157</td><td>0,137027423</td><td>23,12901</td><td>24,0253</td><td>22,05639</td><td>24,31341</td><td>23,14718</td><td>23,90288</td><td>NaN</td><td>23,5041</td></tr>
<tr><td>tr|B4DY09|B4DY09_HUMAN;sp|Q12905|ILF2_HUMAN;tr|X6R6Z1|X6R6Z1_HUMAN;tr|A0A0A0M</td><td>tr|B4DY09|B4DY09_HUMAN;sp|Q12905|ILF2_HUMAN;tr|X6R6Z1|X6R6Z1_HUMAN</td><td>59,585</td><td>203340000</td><td>32</td><td>8</td><td>0,83787865</td><td>0,04769516</td><td>23,54439</td><td>23,51147</td><td>23,92162</td><td>23,46787</td><td>23,11626</td><td>23,82172</td><td>24,04051</td><td>23,65764</td></tr>
<tr><td>sp|P60891|PRPS1_HUMAN;tr|B1ALA9|B1ALA9_HUMAN;tr|A0A0B4J207|A0A0B4J207_HUMAN;</td><td>sp|P60891|PRPS1_HUMAN;tr|B1ALA9|B1ALA9_HUMAN;tr|A0A0B4J207|A0A0B4J207_HUMAN;</td><td>36,823</td><td>81797000</td><td>14</td><td>2</td><td>0,85736471</td><td>0,046547413</td><td>22,42668</td><td>22,09484</td><td>22,44223</td><td>22,6804</td><td>22,38055</td><td>22,86081</td><td>21,88024</td><td>22,70872</td></tr>
<tr><td>tr|F5H1S8|F5H1S8_HUMAN;tr|F5GX14|F5GX14_HUMAN;sp|Q14165|MLEC_HUMAN;tr|H0YG07</td><td>tr|F5H1S8|F5H1S8_HUMAN;tr|F5GX14|F5GX14_HUMAN;sp|Q14165|MLEC_HUMAN;tr|H0YG07</td><td>4,2111</td><td>10183000</td><td>5</td><td>2</td><td>0,85777637</td><td>0,033931255</td><td>19,39236</td><td>19,35204</td><td>19,58448</td><td>19,31754</td><td>19,76801</td><td>18,96079</td><td>19,52241</td><td>19,53094</td></tr>
<tr><td>sp|P63092|GNAS2_HUMAN;sp|Q5JWF2|GNAS1_HUMAN;tr|A0A087WTB6|A0A087WTB6_HUM</td><td>sp|P63092|GNAS2_HUMAN;sp|Q5JWF2|GNAS1_HUMAN</td><td>3,4451</td><td>10823000</td><td>4</td><td>3</td><td>0,86025774</td><td>0,099899769</td><td>18,50421</td><td>19,8903</td><td>19,72059</td><td>NaN</td><td>19,79986</td><td>20,07535</td><td>19,47508</td><td>18,53609</td></tr>
<tr><td>sp|Q13151|ROA0_HUMAN</td><td>sp|Q13151|ROA0_HUMAN</td><td>13,581</td><td>123840000</td><td>15</td><td>5</td><td>0,86183289</td><td>-0,086868286</td><td>22,36228</td><td>22,22647</td><td>23,49167</td><td>22,82196</td><td>22,10847</td><td>23,11095</td><td>23,46176</td><td>21,87373</td></tr>
<tr><td>sp|Q13247|SRSF6_HUMAN;tr|A0A0D9SEM4|A0A0D9SEM4_HUMAN;sp|Q08170|SRSF4_HUMAN</td><td>sp|Q13247|SRSF6_HUMAN;tr|A0A0D9SEM4|A0A0D9SEM4_HUMAN;sp|Q08170|SRSF4_HUMAN</td><td>15,459</td><td>57956000</td><td>9</td><td>2</td><td>0,8627858</td><td>0,032172203</td><td>21,63139</td><td>21,59099</td><td>22,03181</td><td>21,79432</td><td>21,6446</td><td>21,84598</td><td>22,18365</td><td>21,50295</td></tr>
<tr><td>sp|P62854|RS26_HUMAN;sp|Q5JNZ5|RS26L_HUMAN</td><td>sp|P62854|RS26_HUMAN;sp|Q5JNZ5|RS26L_HUMAN</td><td>3,8877</td><td>226860000</td><td>10</td><td>2</td><td>0,8644041</td><td>0,099488258</td><td>22,23002</td><td>24,39185</td><td>24,62901</td><td>23,99921</td><td>24,0559</td><td>23,76729</td><td>23,63334</td><td>24,19152</td></tr>
<tr><td>sp|Q6P2Q9|PRP8_HUMAN;tr|I3L0J9|I3L0J9_HUMAN;tr|I3L3Z8|I3L3Z8_HUMAN</td><td>sp|Q6P2Q9|PRP8_HUMAN</td><td>49,856</td><td>73359000</td><td>23</td><td>11</td><td>0,86594539</td><td>-0,032904148</td><td>22,08845</td><td>22,32858</td><td>22,11839</td><td>21,87128</td><td>21,69166</td><td>21,982</td><td>22,46834</td><td>22,13308</td></tr>
<tr><td>sp|Q07065|CKAP4_HUMAN</td><td>sp|Q07065|CKAP4_HUMAN</td><td>11,946</td><td>29357000</td><td>14</td><td>5</td><td>0,86662668</td><td>-0,007584572</td><td>20,92976</td><td>20,82974</td><td>20,9512</td><td>20,92172</td><td>20,9011</td><td>20,8226</td><td>20,89045</td><td>20,98794</td></tr>
<tr><td>sp|P54727|RD23B_HUMAN;tr|Q5W0S5|Q5W0S5_HUMAN;tr|Q5W0S4|Q5W0S4_HUMAN</td><td>sp|P54727|RD23B_HUMAN;tr|Q5W0S5|Q5W0S5_HUMAN</td><td>13,253</td><td>42216000</td><td>5</td><td>3</td><td>0,8690407</td><td>0,048540274</td><td>21,40416</td><td>20,8053</td><td>NaN</td><td>21,8064</td><td>21,16337</td><td>21,35326</td><td>21,31766</td><td>21,71436</td></tr>
<tr><td>sp|O75439|MPPB_HUMAN;tr|G3V0E4|G3V0E4_HUMAN;tr|F8WAZ6|F8WAZ6_HUMAN;tr|F8WE</td><td>sp|O75439|MPPB_HUMAN;tr|G3V0E4|G3V0E4_HUMAN;tr|F8WAZ6|F8WAZ6_HUMAN</td><td>10,087</td><td>27743000</td><td>9</td><td>4</td><td>0,87072684</td><td>0,026700497</td><td>21,0448</td><td>20,84698</td><td>20,99106</td><td>20,61935</td><td>21,24696</td><td>20,79265</td><td>20,66284</td><td>20,90654</td></tr>
<tr><td>sp|P13489|RINI_HUMAN;tr|H0YCR7|H0YCR7_HUMAN;tr|E9PMJ3|E9PMJ3_HUMAN;tr|E9PLZ3|</td><td>sp|P13489|RINI_HUMAN;tr|H0YCR7|H0YCR7_HUMAN</td><td>43,267</td><td>34117000</td><td>16</td><td>5</td><td>0,87154538</td><td>0,027476311</td><td>20,77965</td><td>20,98461</td><td>21,05186</td><td>21,45498</td><td>21,12912</td><td>21,25954</td><td>20,87313</td><td>21,11921</td></tr>
<tr><td>sp|Q8WUA2|PPIL4_HUMAN</td><td>sp|Q8WUA2|PPIL4_HUMAN</td><td>4,5755</td><td>10064000</td><td>5</td><td>3</td><td>0,87301611</td><td>-0,021102428</td><td>19,48792</td><td>19,28604</td><td>19,38934</td><td>19,43831</td><td>19,40786</td><td>19,24488</td><td>19,16268</td><td>19,70178</td></tr>
<tr><td>tr|C9JLU1|C9JLU1_HUMAN;sp|P52434|RPAB3_HUMAN</td><td>tr|C9JLU1|C9JLU1_HUMAN;sp|P52434|RPAB3_HUMAN</td><td>14,056</td><td>42017000</td><td>10</td><td>3</td><td>0,8749092</td><td>0,046723525</td><td>NaN</td><td>21,15047</td><td>21,6356</td><td>21,34727</td><td>21,50964</td><td>21,99828</td><td>21,16282</td><td>21,02729</td></tr>
<tr><td>tr|M0R0F0|M0R0F0_HUMAN;sp|P46782|RS5_HUMAN;tr|M0R0R2|M0R0R2_HUMAN;tr|M0QZN</td><td>tr|M0R0F0|M0R0F0_HUMAN;sp|P46782|RS5_HUMAN;tr|M0R0R2|M0R0R2_HUMAN</td><td>54,622</td><td>470460000</td><td>13</td><td>7</td><td>0,87751129</td><td>-0,035294056</td><td>24,72312</td><td>25,25961</td><td>25,08201</td><td>24,61785</td><td>24,8274</td><td>25,33987</td><td>24,78122</td><td>24,59292</td></tr>
<tr><td>sp|Q04760|LGUL_HUMAN</td><td>sp|Q04760|LGUL_HUMAN</td><td>7,0221</td><td>82145000</td><td>9</td><td>3</td><td>0,87790537</td><td>0,027239323</td><td>22,47719</td><td>22,19352</td><td>22,40795</td><td>22,9456</td><td>22,5154</td><td>22,59391</td><td>22,37008</td><td>22,65384</td></tr>
<tr><td>tr|G3V1C3|G3V1C3_HUMAN;sp|Q9BZZ5|API5_HUMAN;tr|E9PQK6|E9PQK6_HUMAN;tr|H0YER7</td><td>tr|G3V1C3|G3V1C3_HUMAN;sp|Q9BZZ5|API5_HUMAN</td><td>7,6606</td><td>76751000</td><td>11</td><td>7</td><td>0,88089795</td><td>-0,032431126</td><td>22,07078</td><td>22,20934</td><td>22,71507</td><td>22,31838</td><td>22,08654</td><td>22,73568</td><td>22,07467</td><td>22,28695</td></tr>
<tr><td>sp|Q9H0S4|DDX47_HUMAN;tr|F5H1N9|F5H1N9_HUMAN</td><td>sp|Q9H0S4|DDX47_HUMAN</td><td>4,2277</td><td>5405300</td><td>4</td><td>3</td><td>0,8814272</td><td>-0,027896881</td><td>NaN</td><td>18,37984</td><td>18,573</td><td>18,70675</td><td>NaN</td><td>18,58053</td><td>18,74895</td><td>18,24642</td></tr>
<tr><td>sp|P50395|GDIB_HUMAN;tr|Q5SX91|Q5SX91_HUMAN;tr|Q5SX87|Q5SX87_HUMAN;tr|Q5SX86</td><td>sp|P50395|GDIB_HUMAN;tr|Q5SX91|Q5SX91_HUMAN;tr|Q5SX87|Q5SX87_HUMAN</td><td>25,766</td><td>107650000</td><td>20</td><td>4</td><td>0,88231966</td><td>-0,045188427</td><td>22,957</td><td>22,9107</td><td>23,00208</td><td>22,52564</td><td>22,37849</td><td>22,87098</td><td>22,41939</td><td>23,54581</td></tr>
<tr><td>sp|P54136|SYRC_HUMAN;tr|E5RH09|E5RH09_HUMAN;tr|E5RJM9|E5RJM9_HUMAN</td><td>sp|P54136|SYRC_HUMAN</td><td>21,755</td><td>105780000</td><td>25</td><td>12</td><td>0,88657192</td><td>-0,014264107</td><td>22,99226</td><td>22,61758</td><td>22,92078</td><td>22,88653</td><td>22,71777</td><td>22,85055</td><td>22,83101</td><td>22,96076</td></tr>
<tr><td>sp|Q92841|DDX17_HUMAN;tr|H3BLZ8|H3BLZ8_HUMAN;tr|A0A0U1RQJ0|A0A0U1RQJ0_HUMA</td><td>sp|Q92841|DDX17_HUMAN;tr|H3BLZ8|H3BLZ8_HUMAN</td><td>26,256</td><td>50096000</td><td>15</td><td>5</td><td>0,89291029</td><td>0,084549427</td><td>19,78681</td><td>21,61742</td><td>22,15419</td><td>22,33194</td><td>21,39273</td><td>21,30028</td><td>21,54213</td><td>21,99341</td></tr>
<tr><td>sp|Q9Y5B9|SP16H_HUMAN</td><td>sp|Q9Y5B9|SP16H_HUMAN</td><td>27,378</td><td>66768000</td><td>20</td><td>10</td><td>0,90181618</td><td>-0,055014133</td><td>21,82054</td><td>21,96969</td><td>22,18013</td><td>21,87849</td><td>21,29771</td><td>21,48207</td><td>23,1426</td><td>21,70641</td></tr>
<tr><td>sp|P53621|COPA_HUMAN</td><td>sp|P53621|COPA_HUMAN</td><td>14,631</td><td>50398000</td><td>23</td><td>11</td><td>0,90738445</td><td>-0,026607513</td><td>22,1062</td><td>21,65211</td><td>21,5255</td><td>21,53146</td><td>21,4491</td><td>21,34066</td><td>21,84506</td><td>22,07401</td></tr>
<tr><td>sp|P60866|RS20_HUMAN;tr|E5RJX2|E5RJX2_HUMAN;tr|E5RIP1|E5RIP1_HUMAN;tr|G3XAN0|G</td><td>sp|P60866|RS20_HUMAN;tr|E5RJX2|E5RJX2_HUMAN;tr|E5RIP1|E5RIP1_HUMAN</td><td>254,75</td><td>389950000</td><td>14</td><td>4</td><td>0,91025009</td><td>-0,024465084</td><td>24,54975</td><td>24,72775</td><td>24,93112</td><td>24,26019</td><td>24,93939</td><td>24,68244</td><td>24,21104</td><td>24,53807</td></tr>
<tr><td>tr|F8W6I7|F8W6I7_HUMAN;sp|P09651|ROA1_HUMAN;tr|F8VZ49|F8VZ49_HUMAN;tr|F8VTQ5|</td><td>tr|F8W6I7|F8W6I7_HUMAN;sp|P09651|ROA1_HUMAN;tr|F8VZ49|F8VZ49_HUMAN;tr|F8VTQ5|</td><td>237,52</td><td>970900000</td><td>63</td><td>15</td><td>0,91108497</td><td>0,038527966</td><td>25,8149</td><td>25,50588</td><td>26,05007</td><td>25,88816</td><td>25,30758</td><td>26,10554</td><td>26,61344</td><td>25,38657</td></tr>
<tr><td>sp|P42285|SK2L2_HUMAN</td><td>sp|P42285|SK2L2_HUMAN</td><td>3,4012</td><td>23330000</td><td>6</td><td>3</td><td>0,9121795</td><td>0,013482412</td><td>20,78061</td><td>20,65334</td><td>NaN</td><td>20,6644</td><td>20,60711</td><td>20,5417</td><td>20,73529</td><td>20,96762</td></tr>
<tr><td>sp|P16615|AT2A2_HUMAN;tr|H7C5W9|H7C5W9_HUMAN;sp|O14983|AT2A1_HUMAN;sp|Q93</td><td>sp|P16615|AT2A2_HUMAN;tr|H7C5W9|H7C5W9_HUMAN</td><td>79,598</td><td>165810000</td><td>24</td><td>14</td><td>0,91327859</td><td>0,033493519</td><td>23,02837</td><td>23,14246</td><td>23,88064</td><td>23,08618</td><td>23,55344</td><td>23,19268</td><td>23,75562</td><td>22,76987</td></tr>
<tr><td>sp|Q92621|NU205_HUMAN;tr|U3KPX2|U3KPX2_HUMAN;tr|U3KQ47|U3KQ47_HUMAN;tr|U3K</td><td>sp|Q92621|NU205_HUMAN</td><td>11,311</td><td>23199000</td><td>11</td><td>7</td><td>0,91380118</td><td>-0,008509636</td><td>20,5559</td><td>20,61369</td><td>20,48434</td><td>20,64201</td><td>20,39111</td><td>20,68594</td><td>20,53222</td><td>20,65264</td></tr>
<tr><td>tr|J3KTA4|J3KTA4_HUMAN;sp|P17844|DDX5_HUMAN;tr|J3QSF1|J3QSF1_HUMAN;tr|J3KRZ1|J3</td><td>tr|J3KTA4|J3KTA4_HUMAN;sp|P17844|DDX5_HUMAN;tr|J3QSF1|J3QSF1_HUMAN;tr|J3KRZ1|J3</td><td>66,808</td><td>619820000</td><td>69</td><td>12</td><td>0,9168989</td><td>0,013437271</td><td>25,11874</td><td>25,15943</td><td>25,51226</td><td>25,13762</td><td>25,0918</td><td>25,25176</td><td>25,46566</td><td>25,17257</td></tr>
<tr><td>sp|Q96RL7|VP13A_HUMAN;tr|H0Y7P8|H0Y7P8_HUMAN</td><td>sp|Q96RL7|VP13A_HUMAN</td><td>12,882</td><td>33363000</td><td>16</td><td>9</td><td>0,92109773</td><td>0,034092426</td><td>20,81167</td><td>21,16153</td><td>20,37401</td><td>21,77692</td><td>21,15956</td><td>20,97225</td><td>21,40826</td><td>20,72042</td></tr>
<tr><td>sp|P24539|AT5F1_HUMAN;tr|Q5QNZ2|Q5QNZ2_HUMAN</td><td>sp|P24539|AT5F1_HUMAN;tr|Q5QNZ2|Q5QNZ2_HUMAN</td><td>35,898</td><td>55004000</td><td>15</td><td>5</td><td>0,92183065</td><td>-0,017696857</td><td>21,97053</td><td>21,75756</td><td>21,93769</td><td>21,67008</td><td>21,5552</td><td>22,25046</td><td>21,8418</td><td>21,6176</td></tr>
<tr><td>sp|Q9BVP2|GNL3_HUMAN</td><td>sp|Q9BVP2|GNL3_HUMAN</td><td>12,779</td><td>81100000</td><td>15</td><td>8</td><td>0,92442291</td><td>0,013251781</td><td>22,49975</td><td>22,31332</td><td>22,52986</td><td>22,27694</td><td>22,38717</td><td>22,10236</td><td>22,5413</td><td>22,64205</td></tr>
<tr><td>sp|Q5VYK3|ECM29_HUMAN;tr|J3KN16|J3KN16_HUMAN;tr|R4GMY1|R4GMY1_HUMAN</td><td>sp|Q5VYK3|ECM29_HUMAN;tr|J3KN16|J3KN16_HUMAN</td><td>6,9008</td><td>12704000</td><td>11</td><td>6</td><td>0,93132973</td><td>0,017841339</td><td>20,03832</td><td>19,30541</td><td>19,654</td><td>19,63456</td><td>19,44447</td><td>20,04394</td><td>19,65901</td><td>19,55624</td></tr>
<tr><td>tr|F8W726|F8W726_HUMAN;sp|Q14157|UBP2L_HUMAN;tr|H0Y5H6|H0Y5H6_HUMAN;tr|H7C2</td><td>tr|F8W726|F8W726_HUMAN;sp|Q14157|UBP2L_HUMAN</td><td>11,972</td><td>37125000</td><td>13</td><td>5</td><td>0,93455125</td><td>-0,026332378</td><td>20,63803</td><td>21,85167</td><td>21,58486</td><td>21,37311</td><td>21,51523</td><td>20,89896</td><td>21,28337</td><td>21,64478</td></tr>
<tr><td>tr|A0A0J9YXB3|A0A0J9YXB3_HUMAN;sp|P61224|RAP1B_HUMAN;sp|A6NIZ1|RP1BL_HUMAN;sp</td><td>tr|A0A0J9YXB3|A0A0J9YXB3_HUMAN;sp|P61224|RAP1B_HUMAN;sp|A6NIZ1|RP1BL_HUMAN;sp</td><td>18,169</td><td>72371000</td><td>17</td><td>5</td><td>0,94890021</td><td>0,024044991</td><td>21,72617</td><td>22,05417</td><td>22,28427</td><td>21,96353</td><td>21,49678</td><td>22,77282</td><td>21,40914</td><td>22,44559</td></tr>
<tr><td>sp|P49721|PSB2_HUMAN;tr|A0A087WVV1|A0A087WVV1_HUMAN</td><td>sp|P49721|PSB2_HUMAN;tr|A0A087WVV1|A0A087WVV1_HUMAN</td><td>40,094</td><td>68796000</td><td>13</td><td>4</td><td>0,95049717</td><td>-0,010965824</td><td>22,42368</td><td>22,06313</td><td>22,03882</td><td>22,12364</td><td>22,48785</td><td>22,01699</td><td>21,82908</td><td>22,27147</td></tr>
<tr><td>sp|O75396|SC22B_HUMAN;tr|A0A087X1A9|A0A087X1A9_HUMAN</td><td>sp|O75396|SC22B_HUMAN</td><td>18,706</td><td>48361000</td><td>11</td><td>5</td><td>0,96286928</td><td>0,007392406</td><td>21,38822</td><td>21,61993</td><td>21,48335</td><td>21,73099</td><td>21,93009</td><td>21,38195</td><td>21,58288</td><td>21,35714</td></tr>
<tr><td>sp|P49792|RBP2_HUMAN;sp|Q7Z3J3|RGPD4_HUMAN;sp|A6NKT7|RGPD3_HUMAN;tr|J3KNE0|</td><td>sp|P49792|RBP2_HUMAN</td><td>11,109</td><td>28534000</td><td>14</td><td>7</td><td>0,9766062</td><td>0,00496769</td><td>20,66258</td><td>20,86831</td><td>20,94027</td><td>20,6778</td><td>20,39435</td><td>20,86204</td><td>21,10194</td><td>20,81049</td></tr>
<tr><td>sp|P51991|ROA3_HUMAN;tr|H7C1J8|H7C1J8_HUMAN</td><td>sp|P51991|ROA3_HUMAN</td><td>137,69</td><td>313080000</td><td>33</td><td>10</td><td>0,98585009</td><td>0,007914543</td><td>24,03414</td><td>23,82852</td><td>24,34908</td><td>23,99213</td><td>23,18696</td><td>24,32113</td><td>25,0857</td><td>23,64174</td></tr>
<tr><td>sp|Q9H9A6|LRC40_HUMAN</td><td>sp|Q9H9A6|LRC40_HUMAN</td><td>5,2626</td><td>16544000</td><td>5</td><td>4</td><td>0,99428915</td><td>0,000837803</td><td>20,14607</td><td>19,97141</td><td>19,96254</td><td>20,11959</td><td>19,96367</td><td>19,98565</td><td>19,90317</td><td>20,35046</td></tr>
</table>

Footnote to line #61:
tr|E5KLK1|E5KLK1_HUMAN;tr|E5KLJ9|E5KLJ9_HUMAN;tr|E5KLJ5|E5KLJ5_HUMAN;tr|E5KLJ6|E5KLJ6_HUMAN
represents OPA1 (optic atrophy 1) / Dynamin-like 120 kDa protein
