# Supplementary material for: Subcellular Localization and Mitotic Interactome Analyses Identify SIRT4 as a Centrosomally Localized and Microtubule Associated Protein
Source: Cells. 2020 Aug 24;9(9):1950. doi: 10.3390/cells9091950 (PMC7564595; doi:10.3390/cells9091950)
Supplement: Supplementary file 1 [file cells-09-01950-s001.zip › cells-830061-supplementary/suppl/Table S2_Antibodies for immunoblot analysis.pdf]

Table S2. Antibodies for immunoblot analysis.

Primary antibodies	Supplier	Species	Dilution	Reference
Flag M2	Sigmal-Aldrich	mouse	1:500	F3165
eGFP	Roche	mouse	1:2.000	11814460001
SIRT4	Proteintech	mouse	1:40.000	66543-1-Ig
SIRT3	Cell Signaling Technology	rabbit	1:500	5490
HDAC6	Santa Cruz	rabbit	1:1000	sc-11420
HDAC6	Cell Signaling Technology	rabbit	1:1000	7558
acetyl. Tubulin (K40)	Abcam	mouse	1:500	ab24610
α-Tubulin	Abcam	rabbit	1:1000	ab52866
OPA1	BD	mouse	1:1000	612607
OPA1		rabbit	1:1000	Barrera M et al. FEBS Lett.2016; 590:3309–22. https://doi.org/10.1002/1873-3468.12384
ATP5A1	Proteintech	rabbit	1:1000	14676-1-AP
ANT2	Cell Signaling Technology	rabbit	1:1000	14671
γ-Tubulin	Sigma-Aldrich	mouse	1:1000	T6557
GCP2	GeneTex	rabbit	1:1000	GTX102281
GCP3	Santa Cruz	mouse	1:1000	sc-373758
Pericentrin	Abcam	rabbit	1:1000	ab4448
GAPDH	Santa Cruz	mouse	1:1000	sc-47724
Na+ / K+ ATPase	Sigma-Aldrich	mouse	1:1000	A276
CDK1	BD	mouse	1:500	610037
