# Supplementary material for: Subcellular Localization and Mitotic Interactome Analyses Identify SIRT4 as a Centrosomally Localized and Microtubule Associated Protein
Source: Cells. 2020 Aug 24;9(9):1950. doi: 10.3390/cells9091950 (PMC7564595; doi:10.3390/cells9091950)
Supplement: Supplementary file 1 [file cells-09-01950-s001.zip › cells-830061-supplementary/suppl/Table S3_antibodies for confocal analysis.pdf]

Primary antibodies	Supplier	Species	Dilution	Reference
eGFP	Roche	mouse	1:1000	11814460001
α-Tubulin	Santa Cruz	rat	1:500	sc-53029
α-Tubulin	Acris	rat	1:500	SM 568P
γ-Tubulin	Sigma-Aldrich	mouse	1:500	T6557
Pericentrin	Abcam	rabbit	1:1000	ab4448
Pericentrin	Abcam	mouse	1:1000	ab28144
SIRT4	Santa Cruz	rabbit	1:500	sc-135053
SIRT4	Sigma Aldrich	mouse	1:500	SAB1407208
TUBGCP2	Santa Cruz	mouse	1:500	sc-377117
TUBGCP3	Santa Cruz	mouse	1:500	sc-373758
SIRT3	Cell Signaling Technology	rabbit	1:500	5490
MTC02	Abcam	mouse	1:500	ab3298

Secondary antibodies	Supplier	Species	Species reactivity	Dilution	Reference
Alexa Fluor 633	Invitrogen - Thermo Fisher Scientific	goat IgG	anti-rat	1:1000	A-21094
Alexa Fluor 488	Invitrogen - Thermo Fisher Scientific	goat IgG	anti-rabbit	1:1000	A-11034
Alexa Fluor 546	Invitrogen - Thermo Fisher Scientific	goat IgG	anti-rabbit	1:1000	A-11035
Alexa Fluor 488	Invitrogen - Thermo Fisher Scientific	goat IgG	anti-mouse	1:1000	A-11029
Alexa Fluor 546	Invitrogen - Thermo Fisher Scientific	goat IgG	anti-mouse	1:1000	A-11003
