# Supplementary material for: Subcellular Localization and Mitotic Interactome Analyses Identify SIRT4 as a Centrosomally Localized and Microtubule Associated Protein
Source: Cells. 2020 Aug 24;9(9):1950. doi: 10.3390/cells9091950 (PMC7564595; doi:10.3390/cells9091950)
Supplement: Supplementary file 1 [file cells-09-01950-s001.zip › cells-830061-supplementary/suppl/western blot images_originals_revised.pdf]

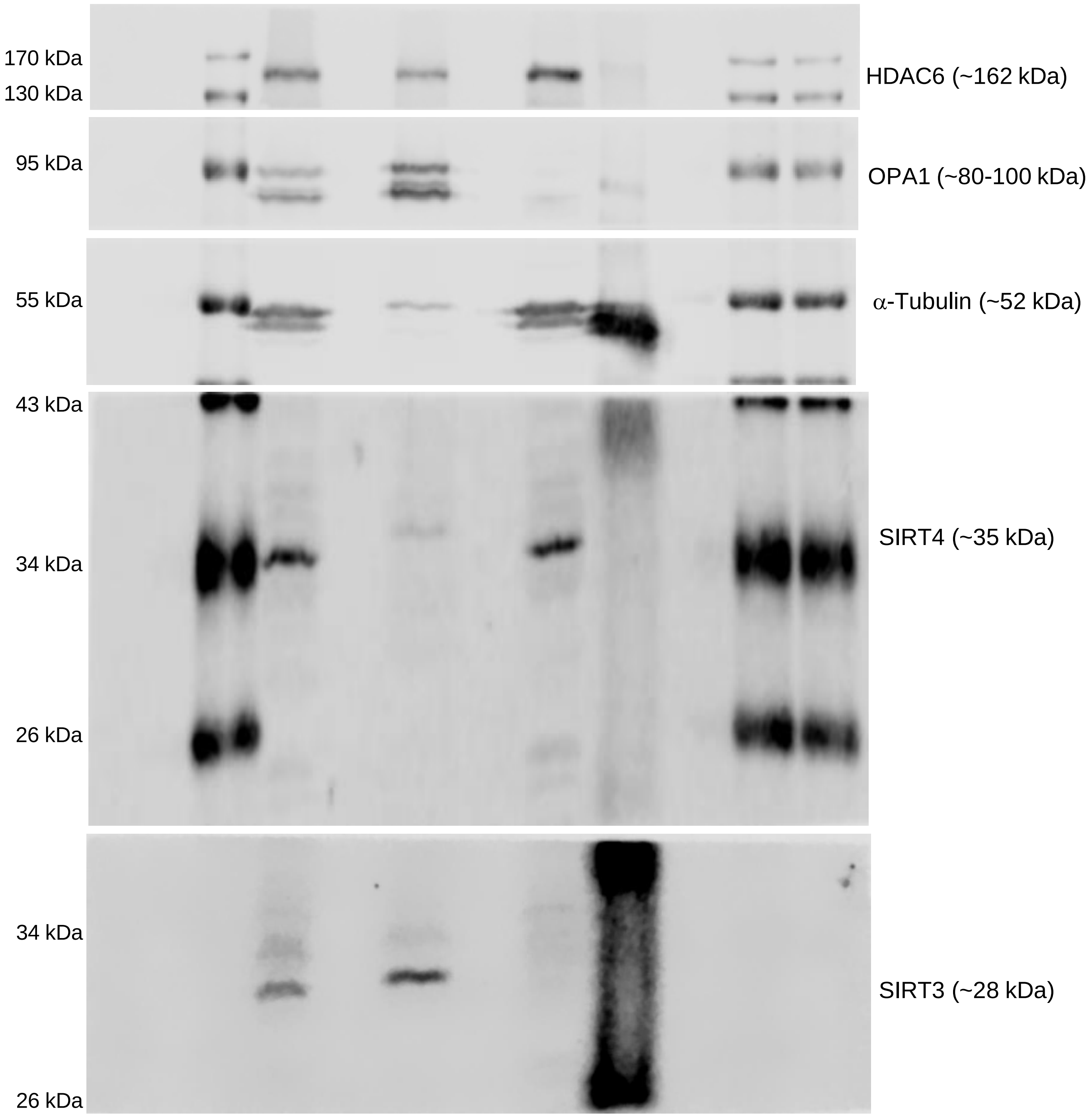
170 kDa
130 kDa
HDAC6 (~162 kDa)
95 kDa
OPA1 (~80-100 kDa)
55 kDa
α-Tubulin (~52 kDa)
43 kDa
34 kDa
26 kDa
SIRT4 (~35 kDa)
34 kDa
26 kDa
SIRT3 (~28 kDa)

Fig. 3

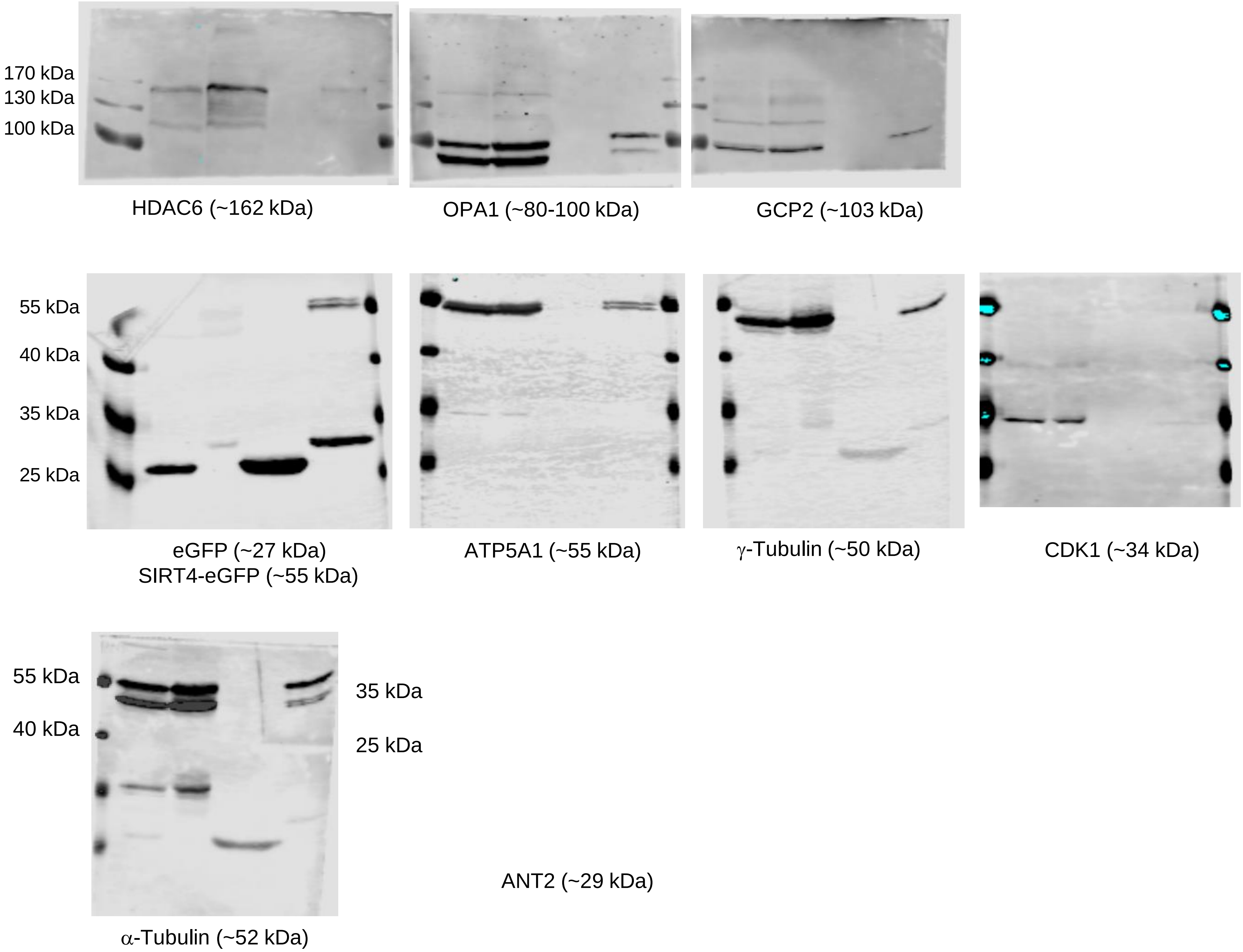
170 kDa
130 kDa
100 kDa
HDAC6 (~162 kDa)
OPA1 (~80-100 kDa)
GCP2 (~103 kDa)
55 kDa
40 kDa
35 kDa
25 kDa
eGFP (~27 kDa)
SIRT4-eGFP (~55 kDa)
ATP5A1 (~55 kDa)
γ-Tubulin (~50 kDa)
CDK1 (~34 kDa)
55 kDa
40 kDa
35 kDa
25 kDa
α-Tubulin (~52 kDa)
ANT2 (~29 kDa)

Fig. 5

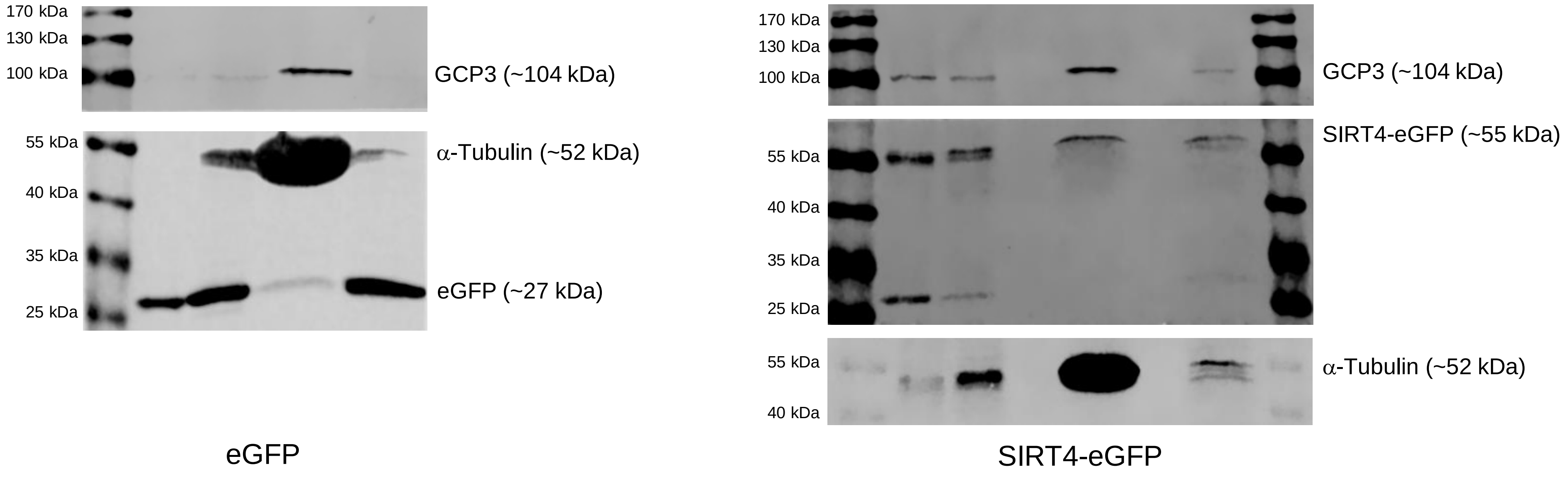
170 kDa
130 kDa
100 kDa
GCP3 (~104 kDa)
55 kDa
α-Tubulin (~52 kDa)
40 kDa
35 kDa
eGFP (~27 kDa)
25 kDa
eGFP
170 kDa
130 kDa
100 kDa
GCP3 (~104 kDa)
SIRT4-eGFP (~55 kDa)
55 kDa
40 kDa
35 kDa
25 kDa
55 kDa
α-Tubulin (~52 kDa)
40 kDa
SIRT4-eGFP

Fig. 6a

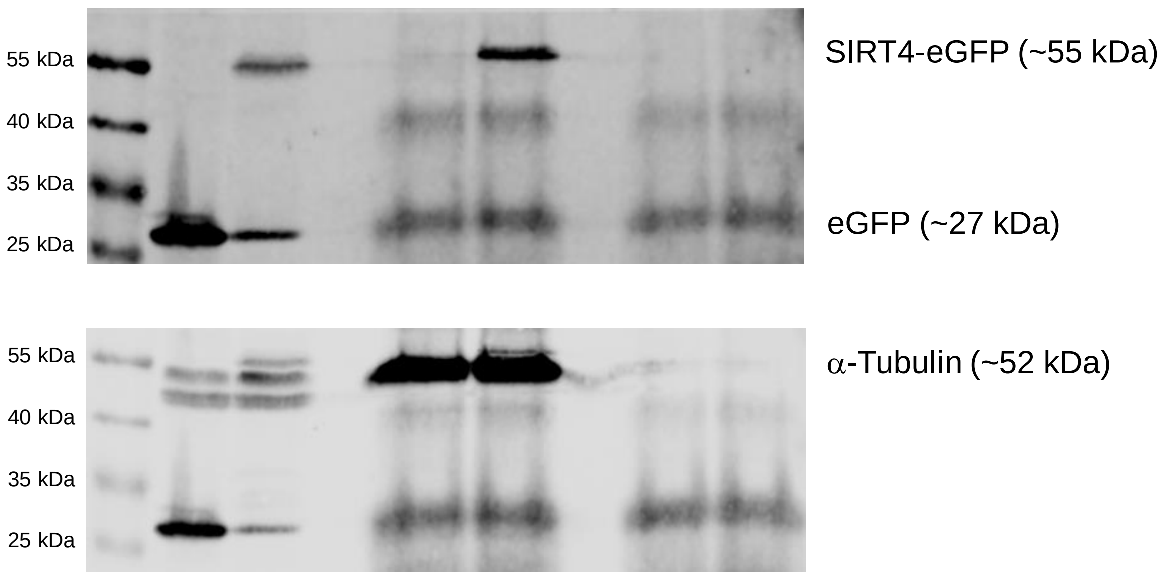
55 kDa
40 kDa
35 kDa
25 kDa
SIRT4-eGFP (~55 kDa)
eGFP (~27 kDa)
55 kDa
40 kDa
35 kDa
25 kDa
α-Tubulin (~52 kDa)

Fig. 6b

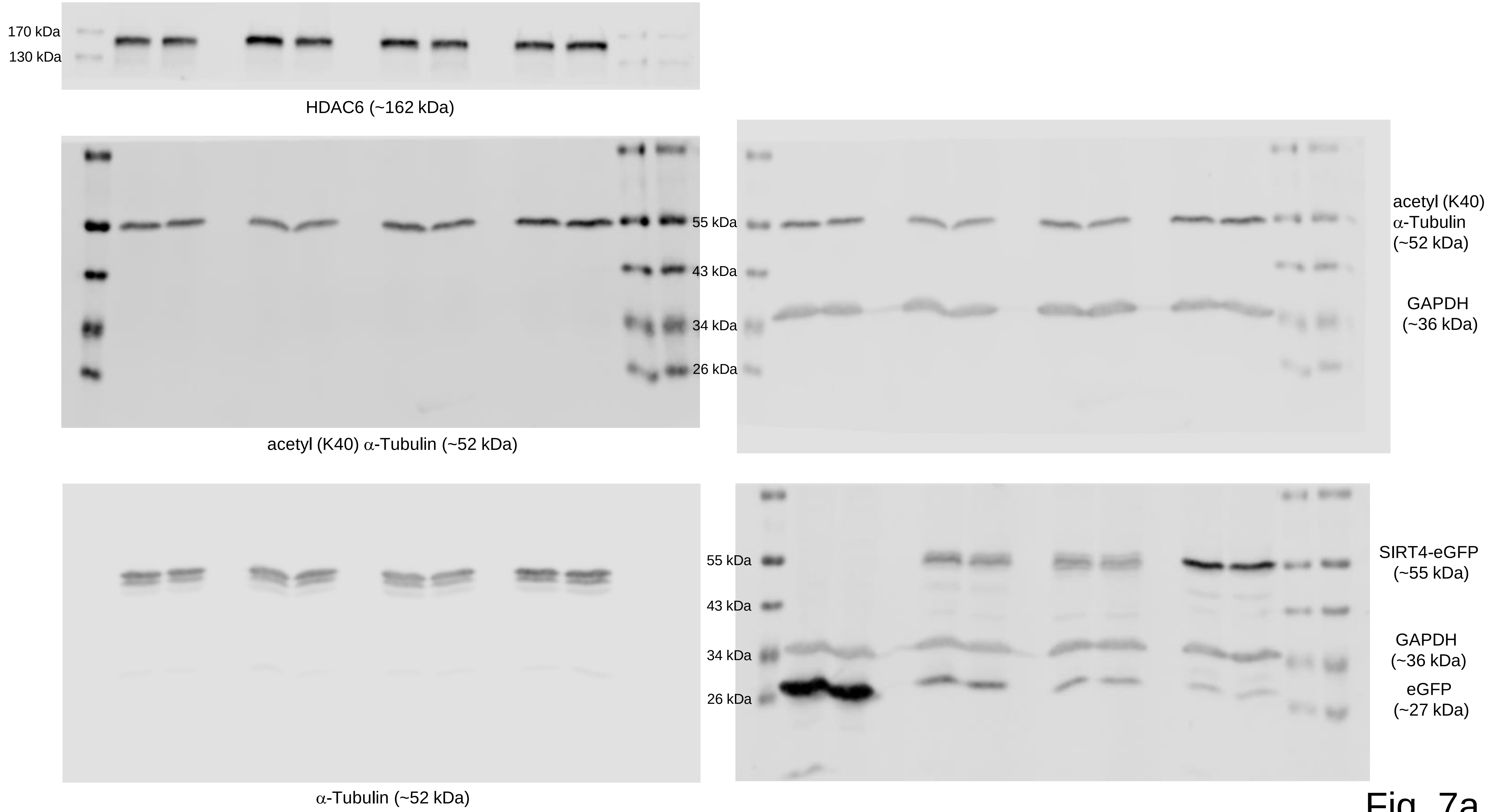

170 kDa
130 kDa
HDAC6 (~162 kDa)
55 kDa
43 kDa
34 kDa
26 kDa
acetyl (K40) α-Tubulin (~52 kDa)
acetyl (K40) α-Tubulin (~52 kDa)
GAPDH (~36 kDa)
α-Tubulin (~52 kDa)
55 kDa
43 kDa
34 kDa
26 kDa
SIRT4-eGFP (~55 kDa)
GAPDH (~36 kDa)
eGFP (~27 kDa)

Fig. 7a

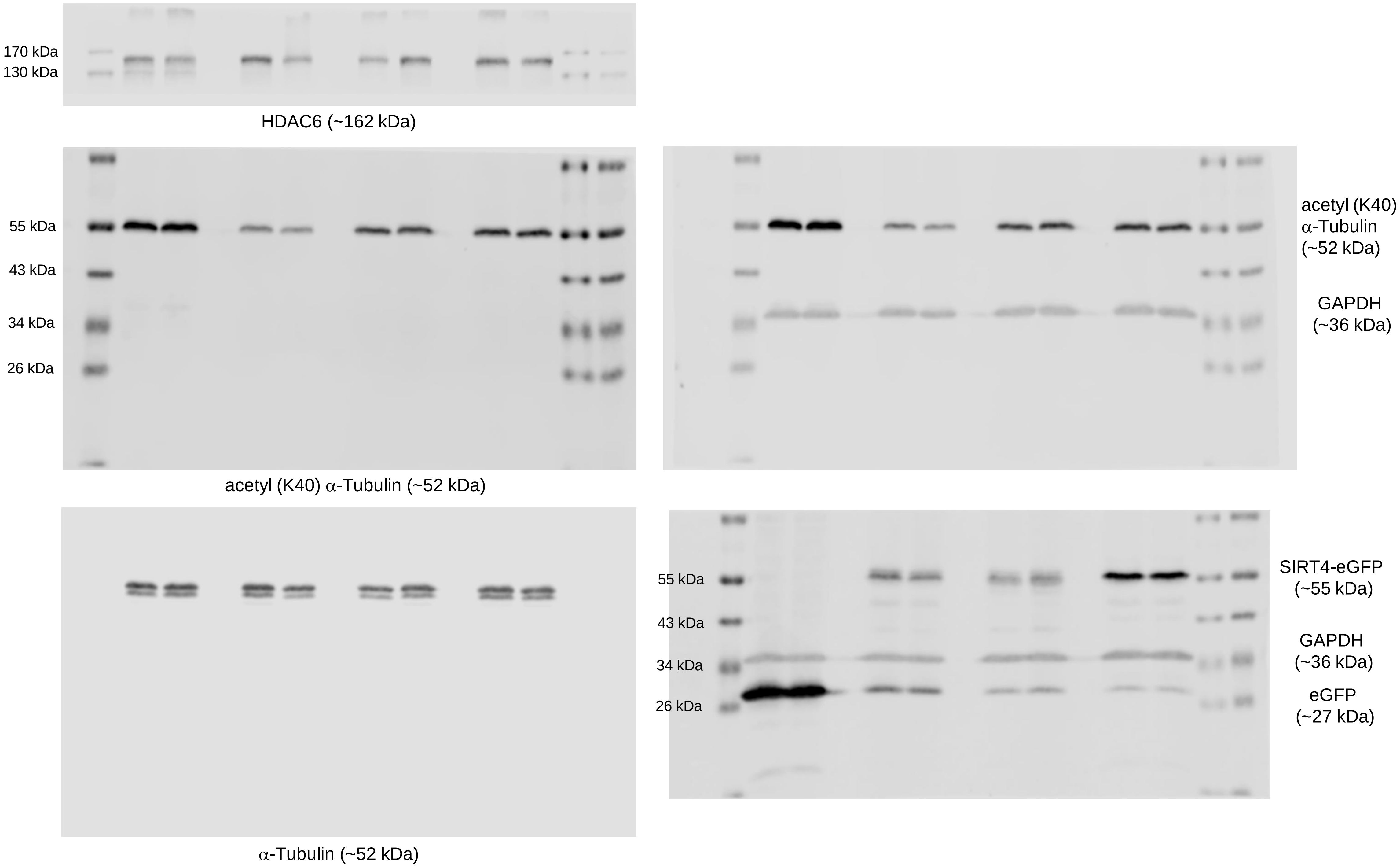
170 kDa
130 kDa
HDAC6 (~162 kDa)
55 kDa
43 kDa
34 kDa
26 kDa
acetyl (K40) α-Tubulin (~52 kDa)
acetyl (K40)
α-Tubulin
(~52 kDa)
GAPDH
(~36 kDa)
α-Tubulin (~52 kDa)
55 kDa
43 kDa
34 kDa
26 kDa
SIRT4-eGFP
(~55 kDa)
GAPDH
(~36 kDa)
eGFP
(~27 kDa)

Fig. 7b

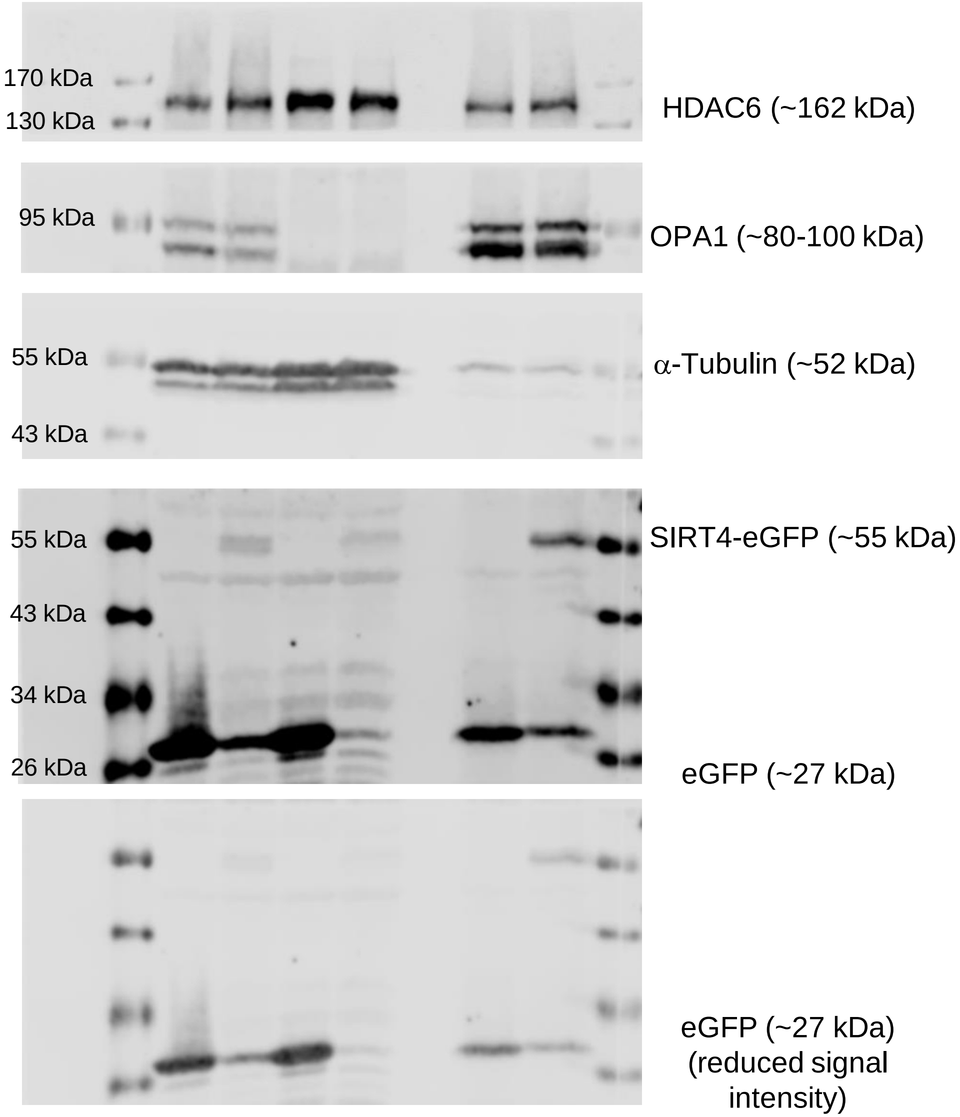
170 kDa
130 kDa
HDAC6 (~162 kDa)
95 kDa
OPA1 (~80-100 kDa)
55 kDa
43 kDa
α-Tubulin (~52 kDa)
55 kDa
43 kDa
34 kDa
26 kDa
SIRT4-eGFP (~55 kDa)
eGFP (~27 kDa)
eGFP (~27 kDa)
(reduced signal
intensity)

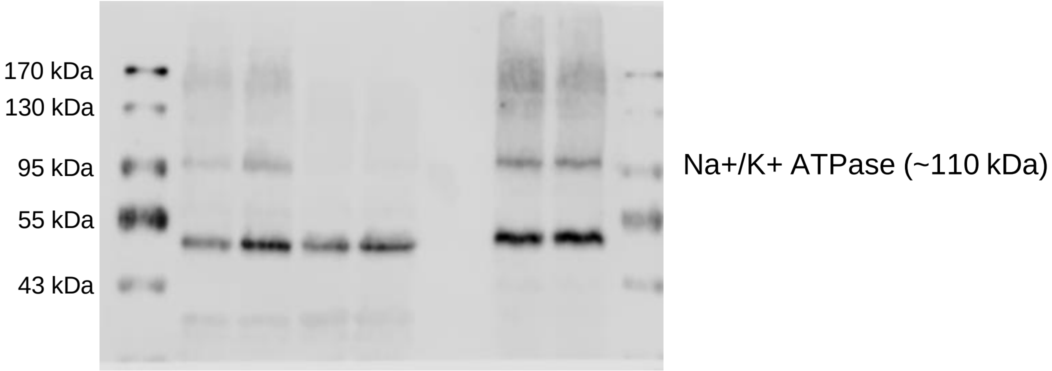
170 kDa
130 kDa
95 kDa
55 kDa
43 kDa
Na+/K+ ATPase (~110 kDa)

Fig. S7

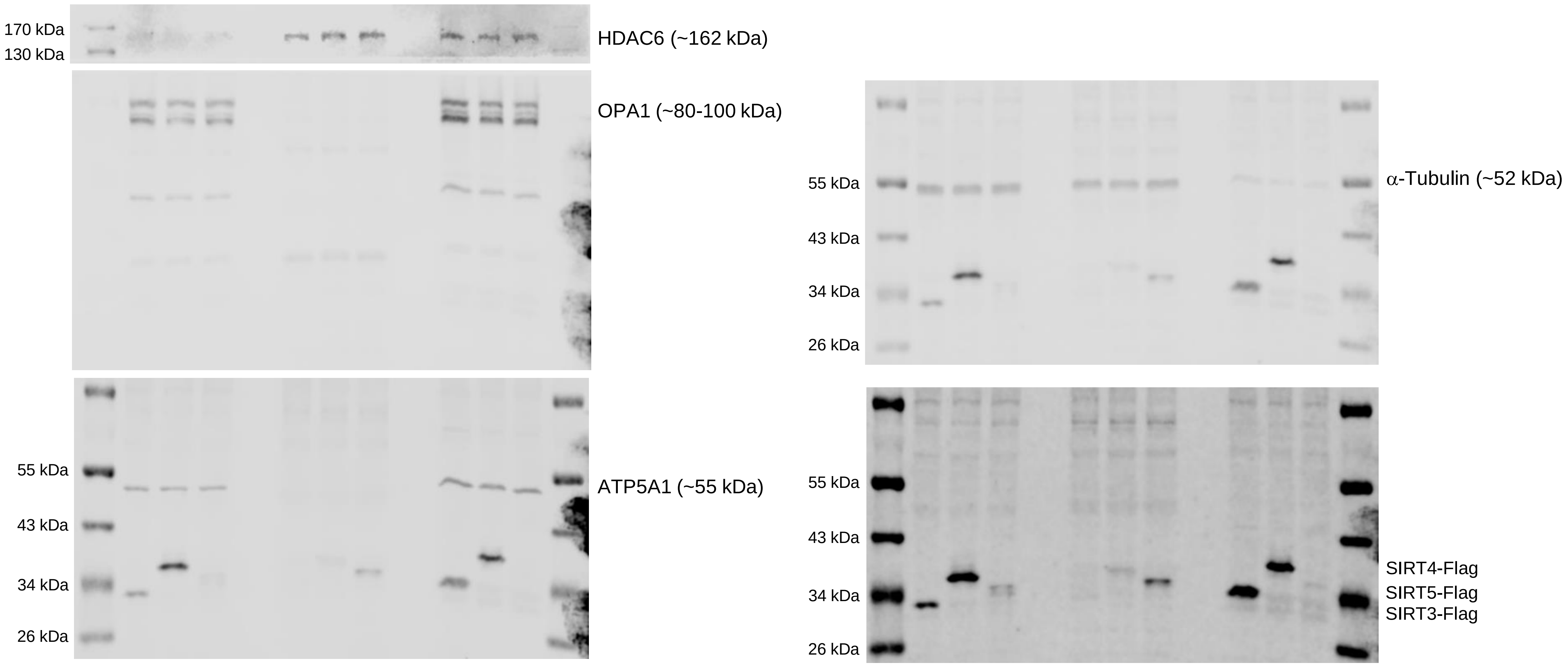
170 kDa
130 kDa
HDAC6 (~162 kDa)
OPA1 (~80-100 kDa)
55 kDa
43 kDa
34 kDa
26 kDa
ATP5A1 (~55 kDa)
55 kDa
43 kDa
34 kDa
26 kDa
α-Tubulin (~52 kDa)
55 kDa
43 kDa
34 kDa
26 kDa
SIRT4-Flag
SIRT5-Flag
SIRT3-Flag

Fig. S8

a

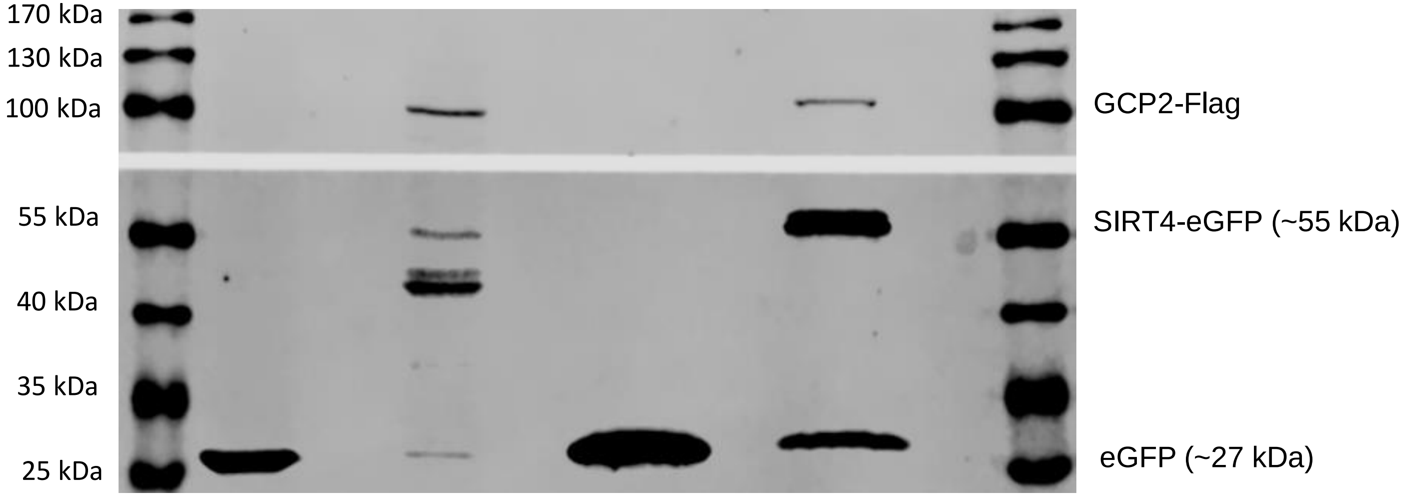
170 kDa
130 kDa
100 kDa
55 kDa
40 kDa
35 kDa
25 kDa
GCP2-Flag
SIRT4-eGFP (~55 kDa)
eGFP (~27 kDa)

b

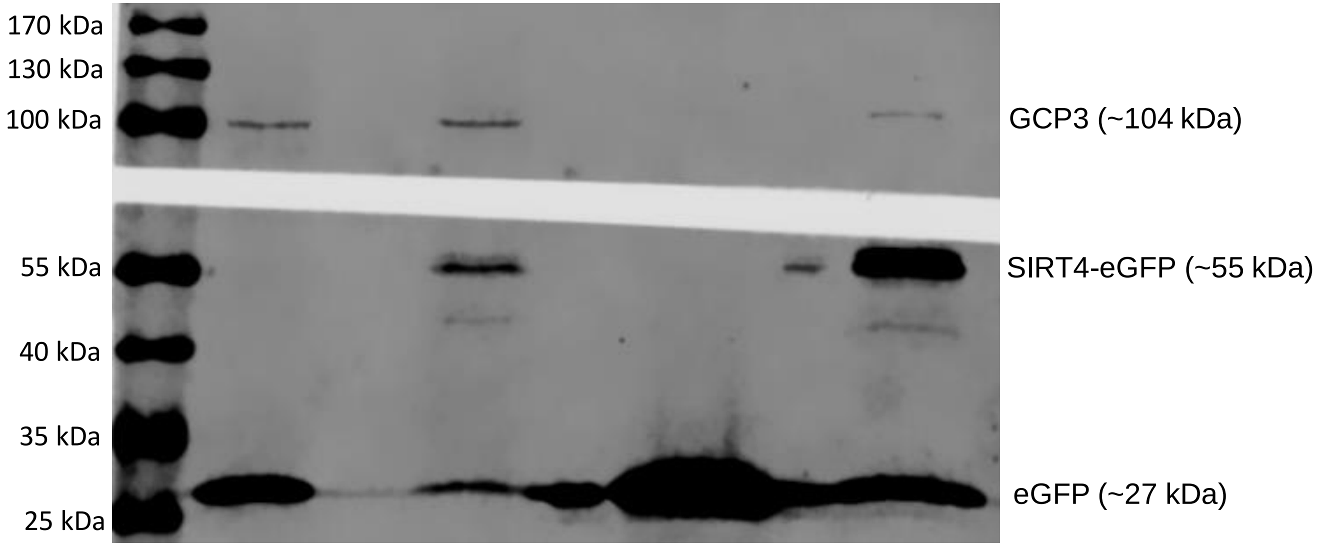
170 kDa
130 kDa
100 kDa
55 kDa
40 kDa
35 kDa
25 kDa
GCP3 (~104 kDa)
SIRT4-eGFP (~55 kDa)
eGFP (~27 kDa)

Fig. S11

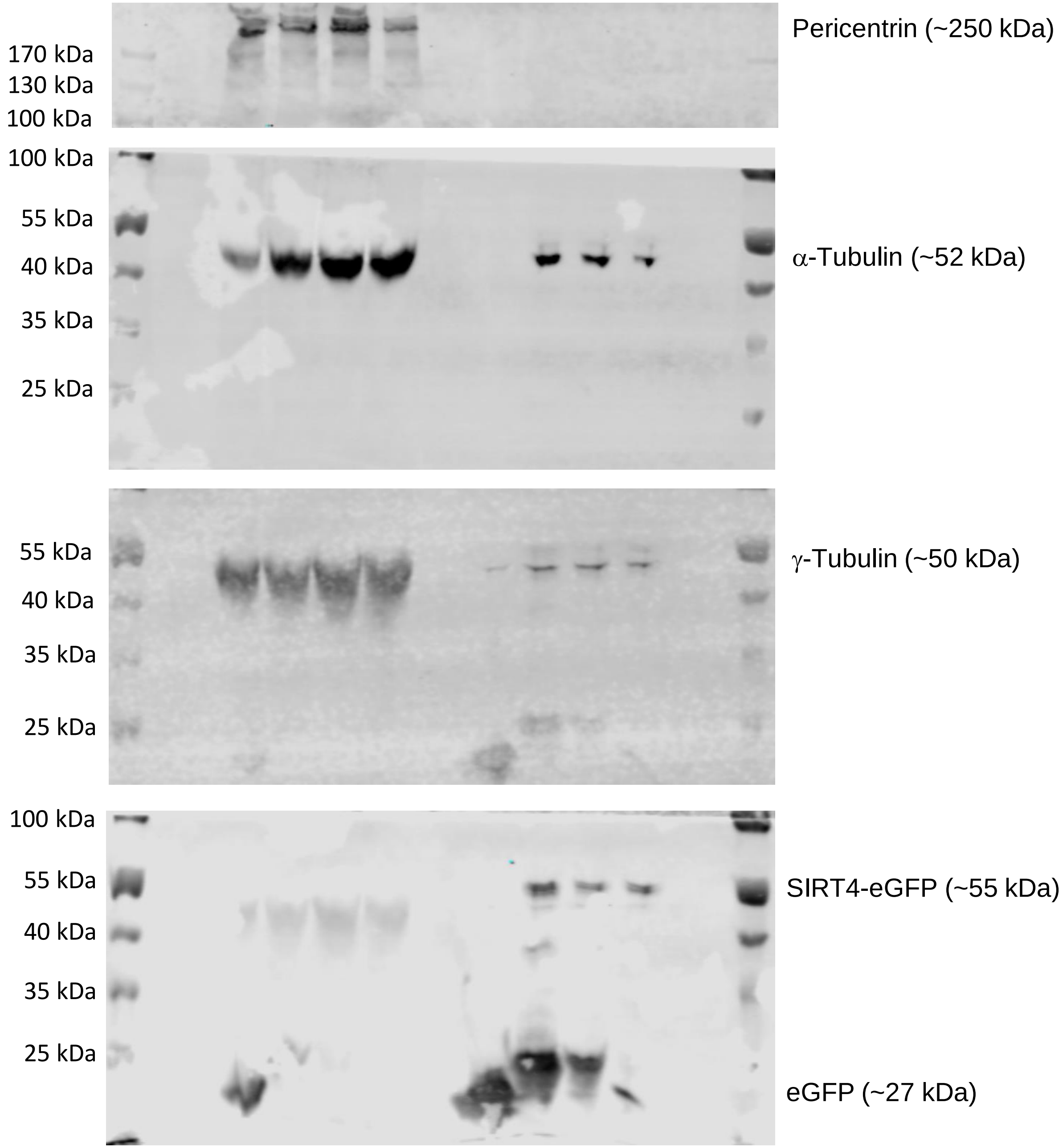
Pericentrin (~250 kDa)
170 kDa
130 kDa
100 kDa
100 kDa
55 kDa
40 kDa
35 kDa
25 kDa
α-Tubulin (~52 kDa)
55 kDa
40 kDa
35 kDa
25 kDa
γ-Tubulin (~50 kDa)
100 kDa
55 kDa
40 kDa
35 kDa
25 kDa
SIRT4-eGFP (~55 kDa)
eGFP (~27 kDa)

Fig. S12

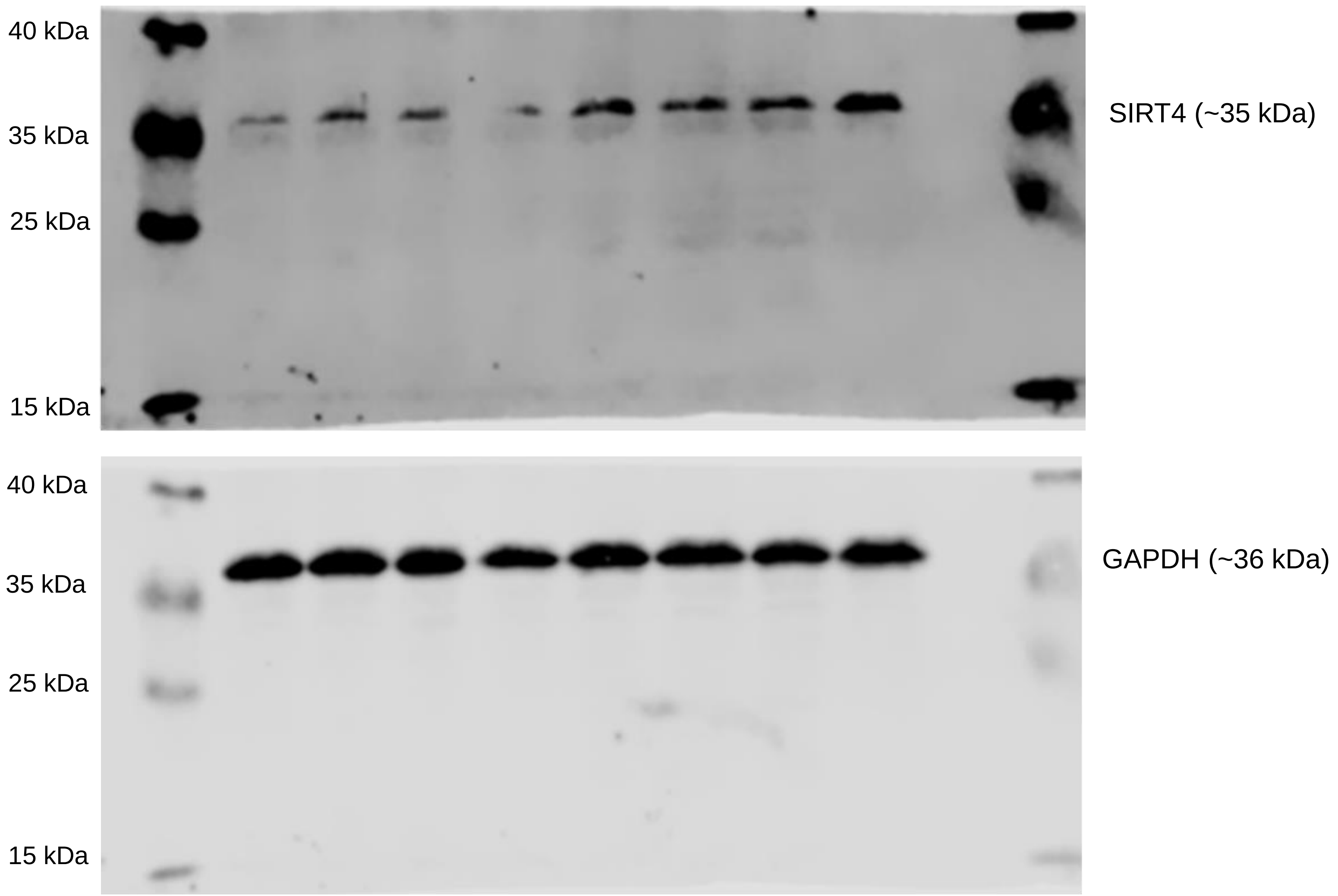
40 kDa
35 kDa
25 kDa
15 kDa
SIRT4 (~35 kDa)
40 kDa
35 kDa
25 kDa
15 kDa
GAPDH (~36 kDa)

Fig. S13
