# Supplementary figures and images for: Subcellular Localization and Mitotic Interactome Analyses Identify SIRT4 as a Centrosomally Localized and Microtubule Associated Protein
Source: Cells. 2020 Aug 24;9(9):1950. doi: 10.3390/cells9091950 (PMC7564595; doi:10.3390/cells9091950)

## Slide 1
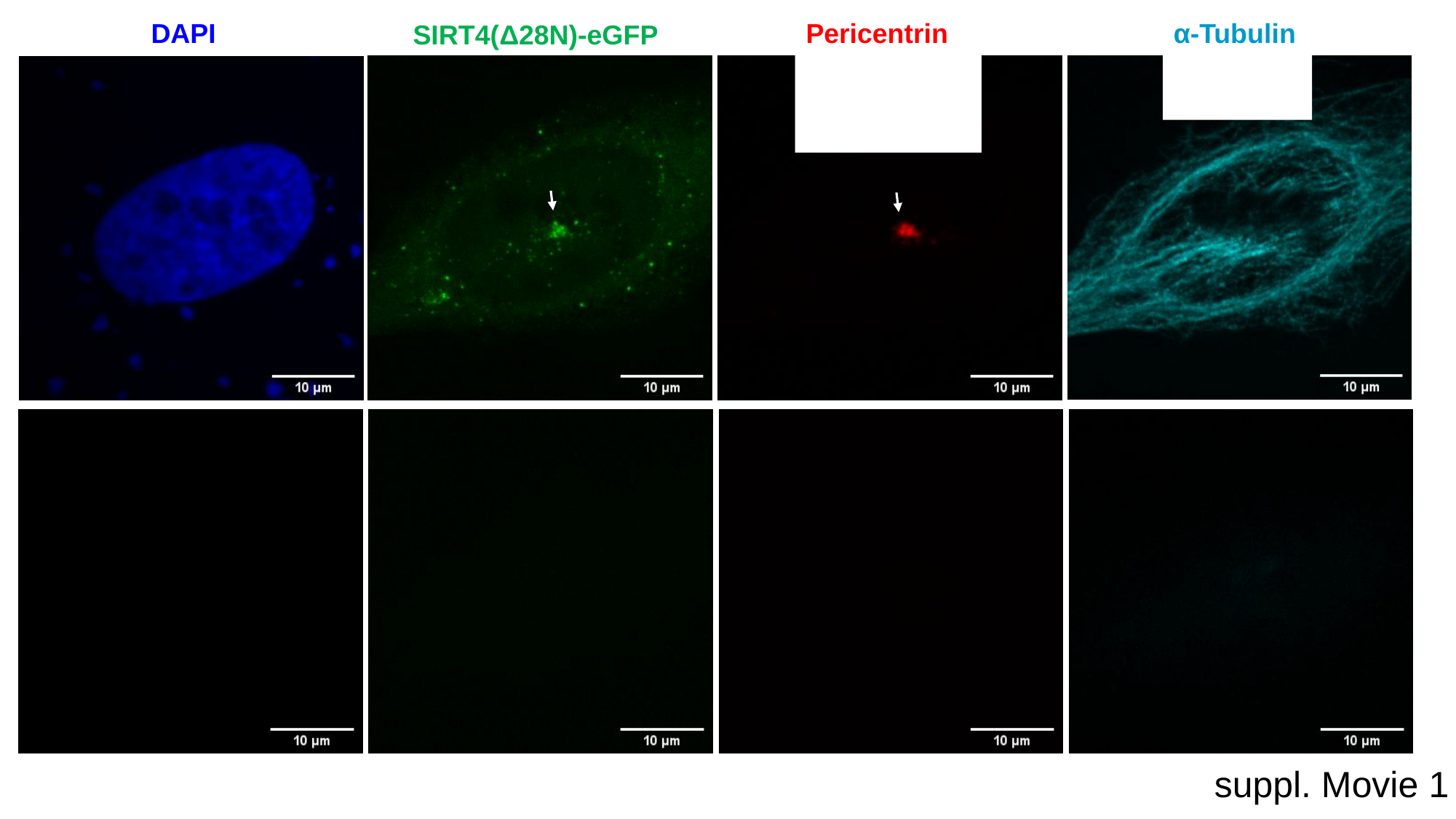

DAPI
Pericentrin
α-Tubulin
SIRT4(Δ28N)-eGFP
suppl. Movie 1

Supplement: Supplementary file 1 [file cells-09-01950-s001.zip › cells-830061-supplementary/suppl/Video_S1.pptx]

## Slide 1
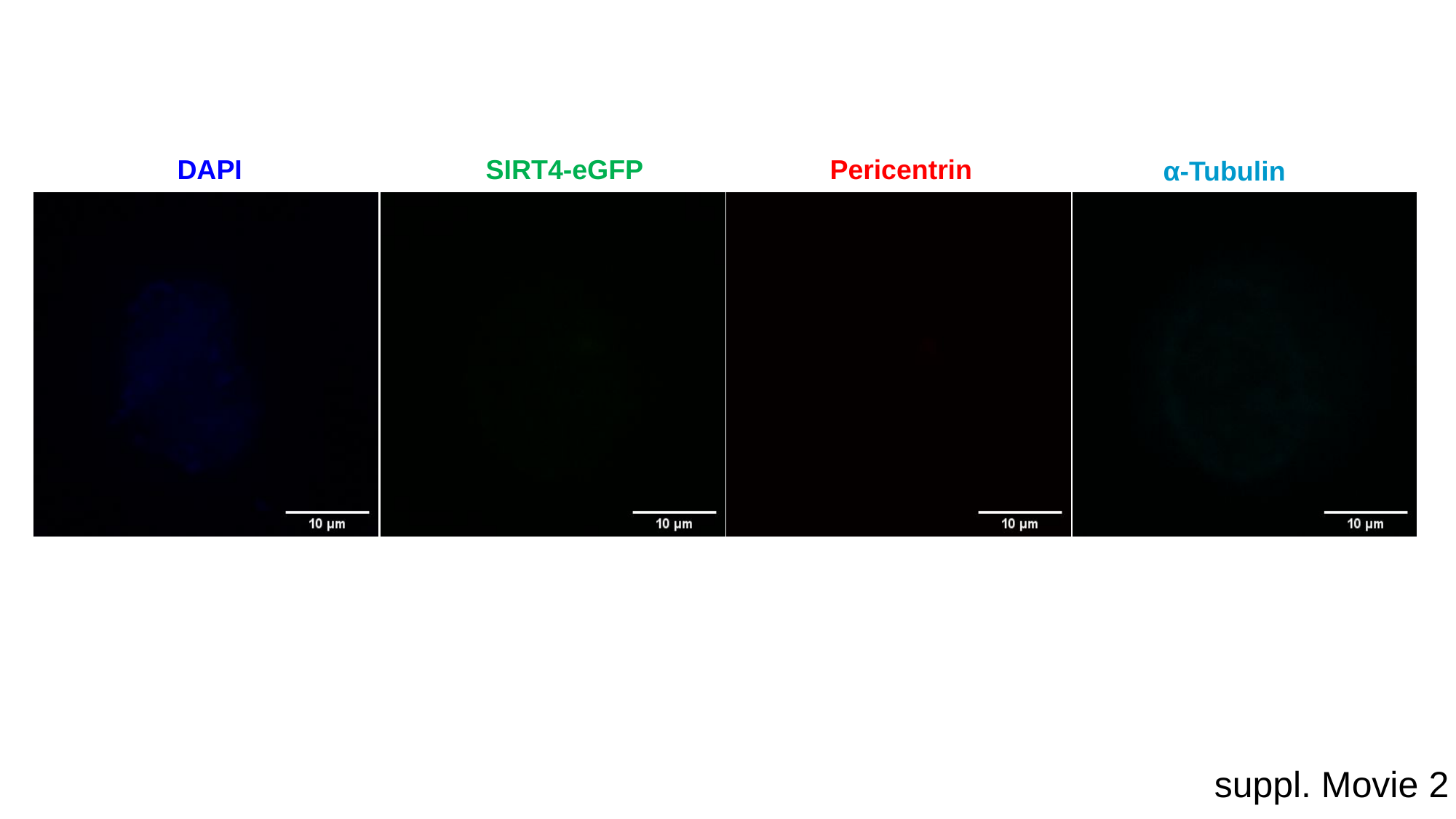

SIRT4-eGFP
Pericentrin
DAPI
α-Tubulin
suppl. Movie 2

Supplement: Supplementary file 1 [file cells-09-01950-s001.zip › cells-830061-supplementary/suppl/Video_S2.pptx]

## Slide 1
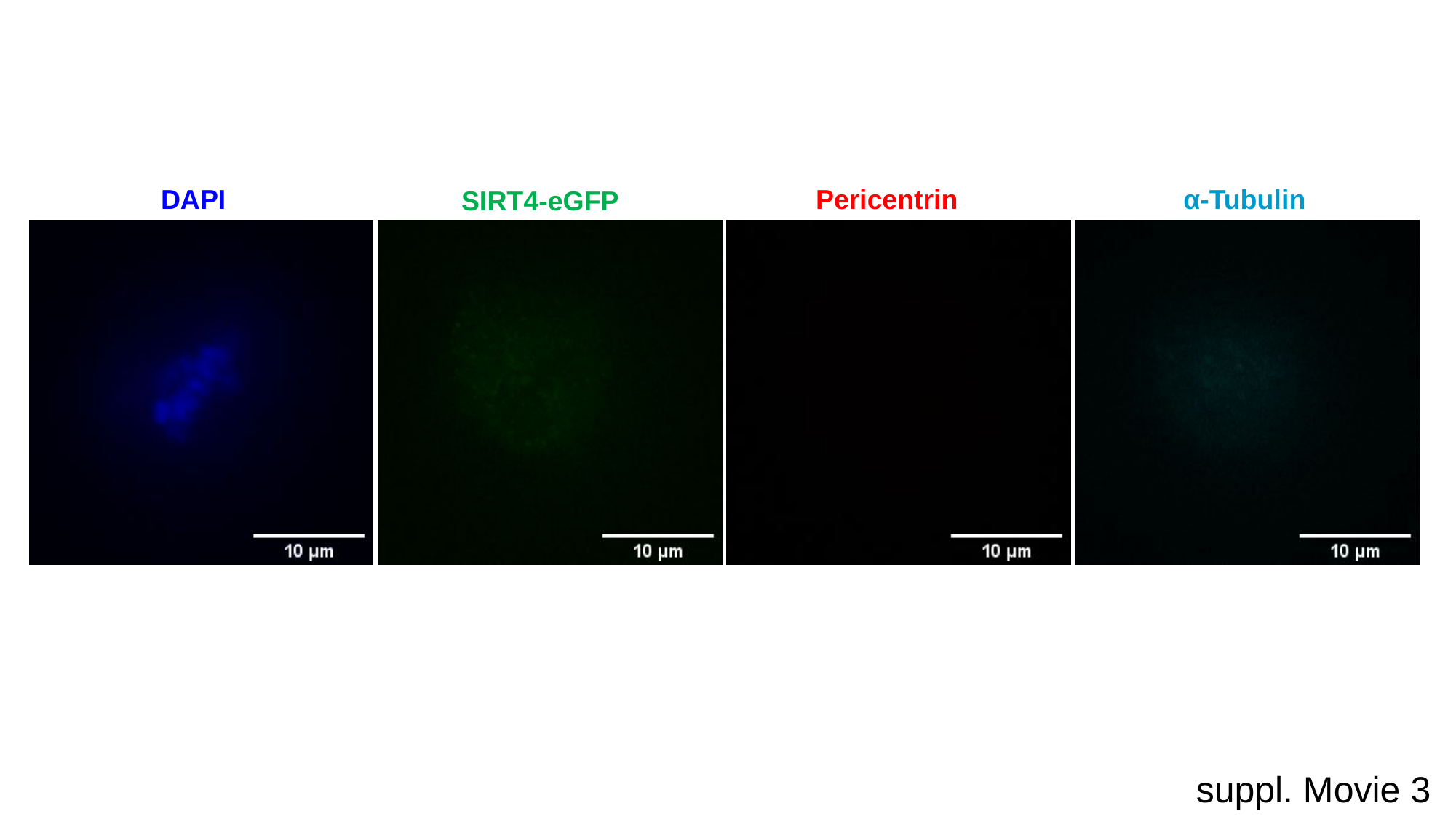

DAPI
Pericentrin
α-Tubulin
SIRT4-eGFP
suppl. Movie 3

Supplement: Supplementary file 1 [file cells-09-01950-s001.zip › cells-830061-supplementary/suppl/Video_S3.pptx]

## Slide 1
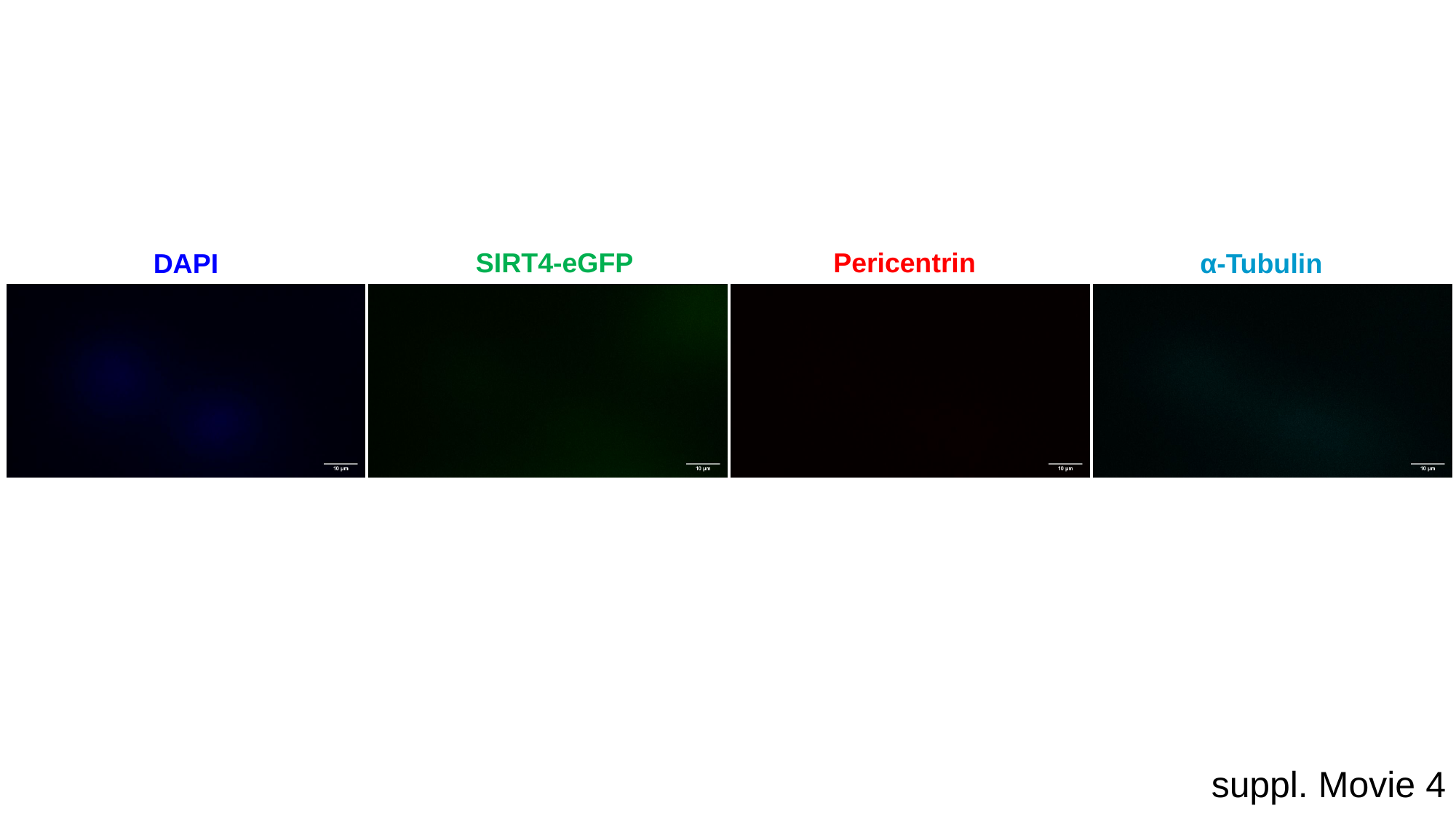

SIRT4-eGFP
Pericentrin
α-Tubulin
DAPI
suppl. Movie 4

Supplement: Supplementary file 1 [file cells-09-01950-s001.zip › cells-830061-supplementary/suppl/Video_S4.pptx]

## Slide 1
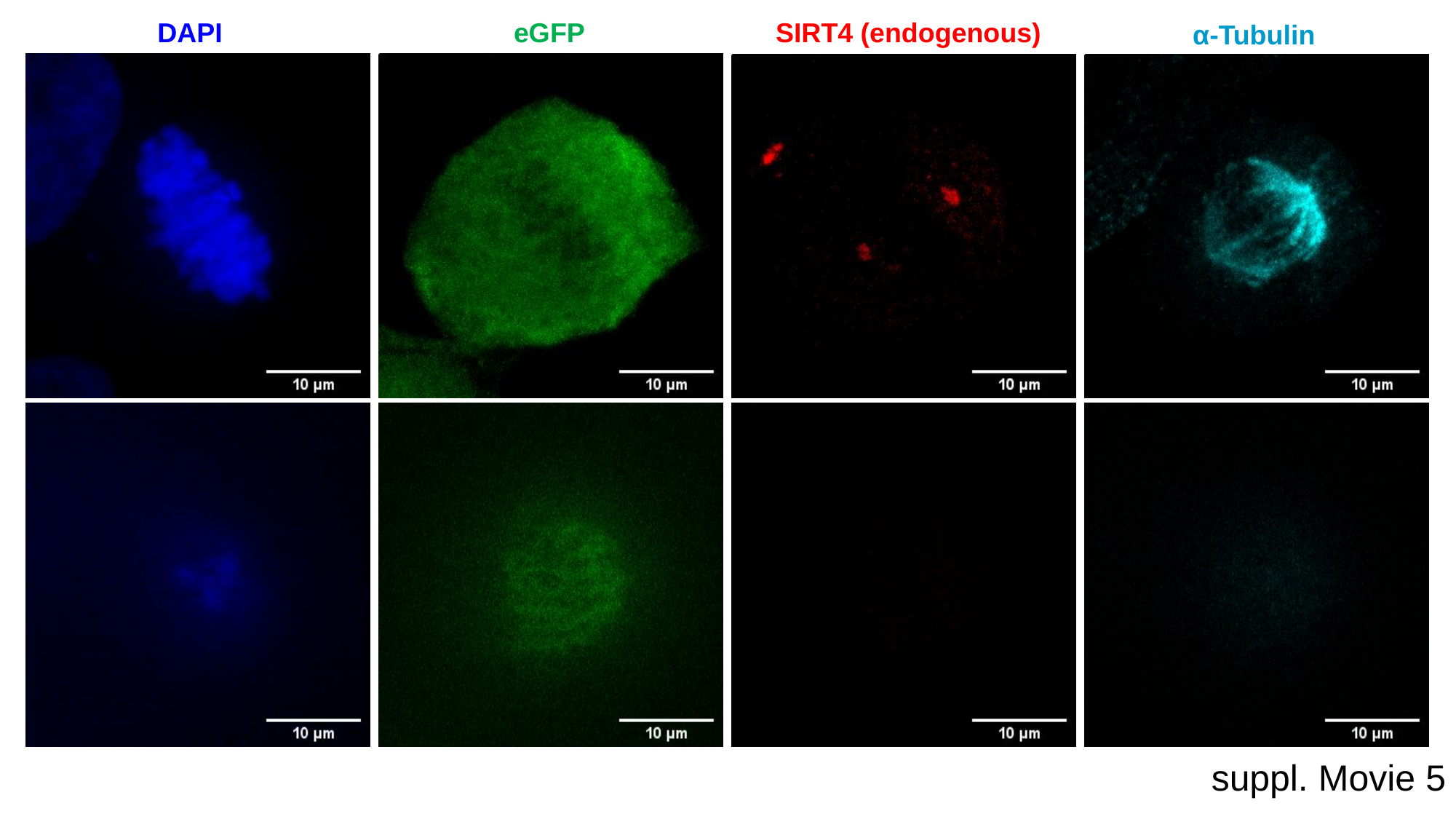

DAPI
eGFP
SIRT4 (endogenous)
α-Tubulin
suppl. Movie 5

Supplement: Supplementary file 1 [file cells-09-01950-s001.zip › cells-830061-supplementary/suppl/Video_S5.pptx]
